# Argonaute2 and LaminB modulate gene expression by controlling chromatin topology

**DOI:** 10.1371/journal.pgen.1007276

**Published:** 2018-03-12

**Authors:** Ezequiel Nazer, Ryan K. Dale, Madoka Chinen, Behram Radmanesh, Elissa P. Lei

**Affiliations:** 1 Nuclear Organization and Gene Expression Section, National Institute of Diabetes and Digestive and Kidney Diseases, National Institutes of Health, Rockville Pike, Bethesda, MD, United States of America; 2 Laboratory of Cellular and Developmental Biology, National Institute of Diabetes and Digestive and Kidney Diseases, National Institutes of Health, Rockville Pike, Bethesda, MD, United States of America; University of Cambridge, UNITED KINGDOM

## Abstract

*Drosophila* Argonaute2 (AGO2) has been shown to regulate expression of certain loci in an RNA interference (RNAi)-independent manner, but its genome-wide function on chromatin remains unknown. Here, we identified the nuclear scaffolding protein LaminB as a novel interactor of AGO2. When either AGO2 or LaminB are depleted in Kc cells, similar transcription changes are observed genome-wide. In particular, changes in expression occur mainly in active or potentially active chromatin, both inside and outside LaminB-associated domains (LADs). Furthermore, we identified a somatic target of AGO2 transcriptional repression, *no hitter* (*nht*), which is immersed in a LAD located within a repressive topologically-associated domain (TAD). Null mutation but not catalytic inactivation of *AGO2* leads to ectopic expression of *nht* and downstream spermatogenesis genes. Depletion of either AGO2 or LaminB results in reduced looping interactions within the *nht* TAD as well as ectopic inter-TAD interactions, as detected by 4C-seq analysis. Overall, our findings reveal coordination of AGO2 and LaminB function to dictate genome architecture and thereby regulate gene expression.

## Author summary

Argonaute proteins are an evolutionarily conserved protein family engaged in gene silencing. The key RNA interference (RNAi) pathway protein AGO2 interacts with small RNAs to regulate gene silencing in the cytoplasm. In addition, AGO2 has been shown to regulate gene expression by functioning in the nucleus. In this study, we determined that AGO2 forms a nuclear complex with LaminB, a nuclear scaffolding protein, as well as the transcription machinery. Together, AGO2 and LaminB limit transcription in active or potentially active regions that either do or do not interact directly with the nuclear lamina. We focused on *nht*, a master control gene of the sperm developmental program, which is up-regulated in the absence of AGO2 or LaminB. The *nht* gene interacts with the nuclear lamina in somatic cells, and we determined that AGO2 and LaminB control the three-dimensional configuration of the chromatin region in which *nht* is located. We conclude that AGO2 and LaminB work in concert to regulate how genes are turned on or off by controlling how the genome is folded within the nucleus, and therefore can affect key developmental processes such as the production of sperm.

## Introduction

Argonaute proteins correspond to an evolutionarily conserved protein family engaged in gene silencing. The well-studied RNA interference (RNAi) pathway effector protein Argonaute2 (AGO2) interacts with microRNAs (miRNAs) or short interfering RNAs (siRNAs) to regulate post-transcriptional gene silencing in the cytoplasm. In addition, several reports have shown that AGO2 is not restricted to the cytoplasm and can also function in the nucleus. In *Drosophila*, ChIP-seq analysis of AGO2 revealed association with active promoters, enhancers, and chromatin insulator sites [1]. At the Hox gene *Abd-B*, AGO2 interacts with insulator proteins and exerts a positive role in gene expression [1]. Consistent with a role in transcription, AGO2 was found to interact with the RNA Polymerase II (Pol II) core complex as well as Negative elongation factor (NELF) [2], a key factor involved in transcriptional pausing. Finally, transcriptional profiling of *AGO2* null but not catalytic mutants suggested that AGO2 functions primarily in transcriptional repression [3]. These results suggest that AGO2 may harbor both positive and negative roles in transcriptional regulation.

AGO2 has also been shown to affect chromatin topology and has been implicated in chromosome pairing. At *Abd-B*, AGO2 is required for proper looping between the promoter and its enhancer, resulting in activation of transcription [1,3]. Moreover, *AGO2* promotes long-range insulator-dependent pairing interactions within the nucleus [4]. In addition to the aforementioned transcriptional profiling [3], all three studies showed that the function of AGO2 in controlling chromatin topology and transcription occurs independently of the RNAi pathway.

Transcription is not only regulated through interactions between promoter and *cis*-regulatory factors, but also through chromatin accessibility and interactions with distant regulatory elements. High-throughput chromosome conformation capture techniques have revealed that chromatin contacts take place within functional and structural domains termed topologically-associated domains (TADs) [5,6]. These structures consist of highly self-interacting genomic regions within the domain, each separated by adjacent genomic regions called domain partition sites (DPSs) [7]. TADs can be sub-classified as active or inactive depending on their protein content and transcriptional activity [8]. A subset of inactive TADs are associated with the nuclear lamina, and these regions are referred to as Lamin-associated domains (LADs). LADs are broad regions defined by their interaction with the LaminB protein (associated with the BLACK chromatin state [9]), and on average, they are 90 kb in length and tend to be gene poor [10]. Less transcriptionally active in nature, LADs are enriched in repressive chromatin marks, such as Polycomb Group (BLUE) and heterochromatin factors (GREEN). Nevertheless, some hallmarks of active chromatin (RED and YELLOW) are also present within LADs [11].

In this study, we elucidate genome-wide mechanisms of AGO2 transcriptional control in concert with LaminB. First, we identified LaminB and Poll II as AGO2-associated factors in nuclear extracts. Nascent euRNA-seq (neuRNA-seq) experiments showed that depletion of AGO2 or LaminB produce highly similar transcriptome profiles, with both factors affecting transcription of genes located in active chromatin both inside and outside LADs. First revealed by transcriptional profiling of *AGO2* mutants, we found that both AGO2 and LaminB prevent transcription of *nht*, a master regulator of the spermatogenesis gene program, in somatic cells. Finally, 4C-seq analysis showed that AGO2 and LaminB modulate the chromatin topology of the TAD/LAD in which *nht* is located, thus contributing to transcriptional silencing of this key developmental regulator.

## Results

### AGO2 forms a nuclear complex with Pol II and LaminB

In order to identify factors that associate with AGO2 in the nucleus, we performed high stringency immunoaffinity purification from embryonic nuclear extracts. We used IgG as a negative control or a monoclonal antibody against AGO2 that has been previously validated to specifically purify AGO2 [12], and immunoprecipitates were analyzed by mass spectrometry. Consistent with previous studies [1,2], we found that the majority of the top-enriched proteins correspond to factors related to Pol II transcription, such as the largest Pol II subunit RPB1 as well as subunits of Mediator (S1 Table). In addition to numerous components of the transcription machinery, a variety of chromatin-associated proteins known to regulate transcription were also identified. Intriguingly, we also identified LaminB as a top-AGO2 interacting protein and selected it for further study because of its known role in nuclear scaffolding; additional proteins identified in this exploratory study remain to be investigated.

**Fig 1:**
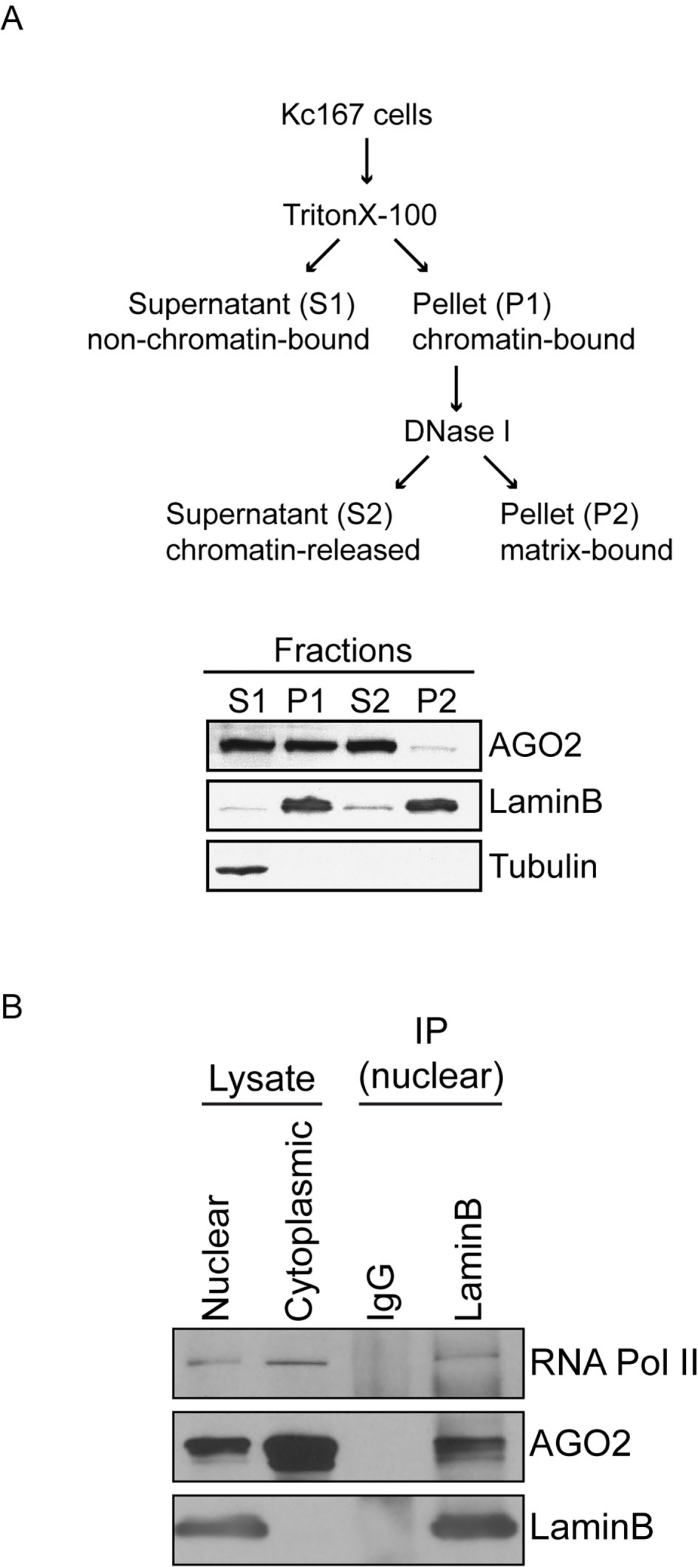
AGO2 forms a nuclear complex with Pol II and LaminB. A) Schematic representation of chromatin fractionation procedure. The S2 fraction consists of soluble chromatin-bound proteins. B) Western blot showing co-immunoprecipitation of endogenous AGO2 and Pol II from Kc nuclear extracts using monoclonal anti-LaminB antibody. Mouse IgG is used for control immunoprecipitations. Nuclear and cytoplasmic lysates are also shown.

In order to verify the interaction between AGO2 and LaminB, we performed biochemical fractionation of chromatin in the embryonic cell line Kc167 (Kc). Both proteins are found in the chromatin fraction (Fig 1A, fraction S2) in contrast to the cytoplasmic control Tubulin (Fig 1A, fraction S1). We immunoprecipitated LaminB from Kc nuclear extracts using anti-LaminB monoclonal antibody, and Western blotting of immunoprecipitates verified the interaction between LaminB and AGO2, in addition to Pol II (Fig 1B). We conclude from these experiments that AGO2, LaminB, and Pol II interact within the nucleus, although not necessarily in a single complex.

### AGO2 and LaminB attenuate transcription genome-wide

**Fig 2:**
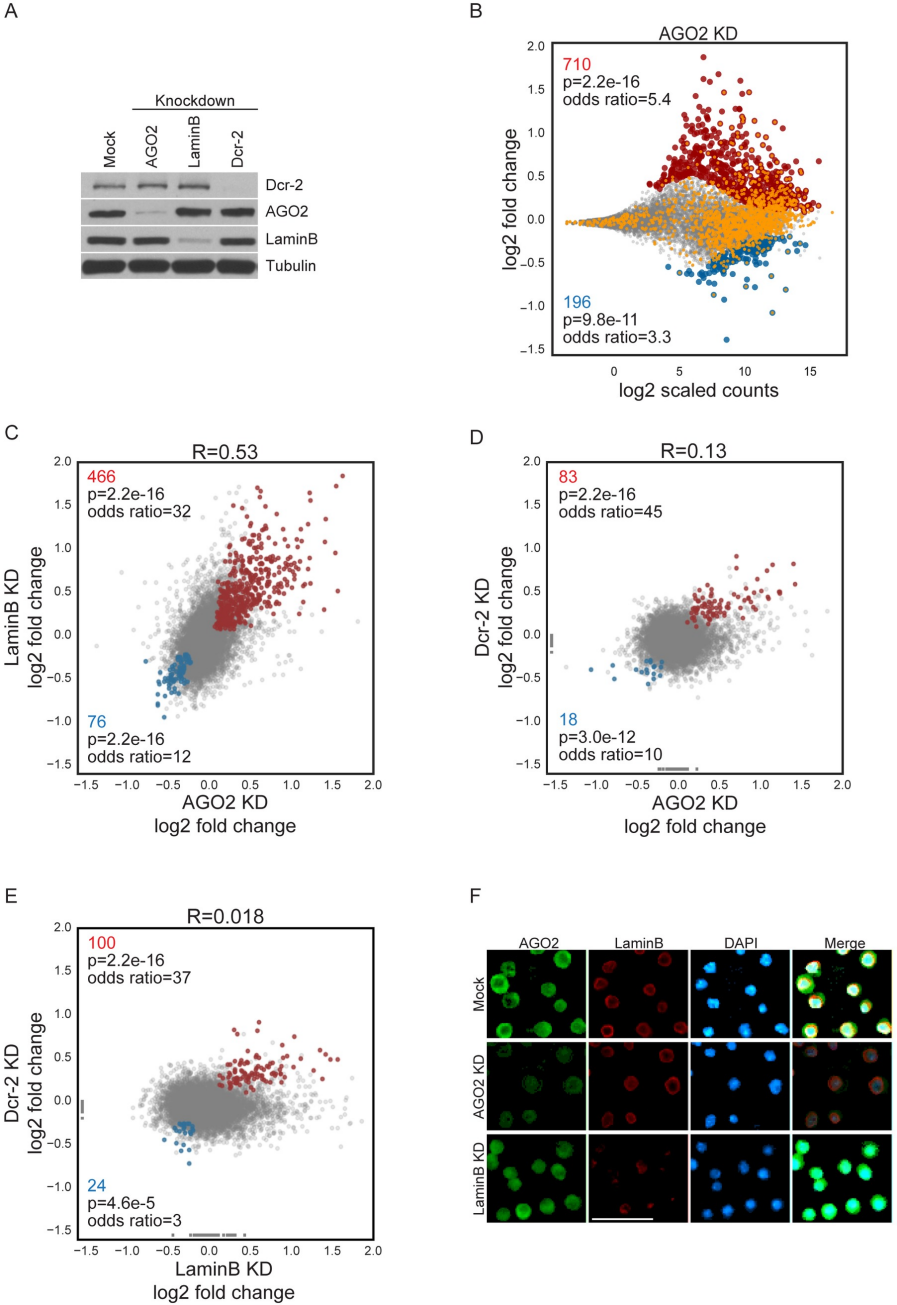
AGO2 and LaminB attenuate transcription genome-wide. A) Western blot analysis showing knockdown efficiencies for each protein analyzed. Tubulin is included as loading control. B) Changes in neuRNA levels upon depletion of AGO2. Statistically significant changes include 710 up-regulated genes (red) and 195 down-regulated genes (blue). (For AGO2 rescue experiments see S1 Fig). Genes containing an AGO2 peak at their promoter are additionally colored orange. FET *p*-values and odds ratios indicate significance of association between affected genes and presence of AGO2 peak at promoters. C) Scatterplot comparing neuRNA-seq profiles from AGO2 and LaminB knockdowns. Pearson’s R corresponds to correlation coefficient of the two profiles. FET *p*-values and odds ratios indicate significance of the association between coordinately up-regulated (466, red) or down-regulated (76, blue) genes in both knockdowns. D) Scatterplot comparing neuRNA-seq profiles from Dcr-2 and AGO2 knockdowns. Genes with undetermined log2 fold change (due to zero counts in one condition) are fixed to a minimal value of -1.5 so they can be visually represented on the plots. Genes with zero counts in both conditions are not displayed. E) Scatterplot comparing neuRNA-seq profiles from Dcr-2 and LaminB knockdowns. F) Immunofluorescence analysis for AGO2 and LaminB in mock, AGO2- and LaminB-depleted cells. AGO2 (green, Liu antibody), LaminB (red), and DAPI staining (blue) are shown. Scale bar represents 14 μm.

Previous work concluded that AGO2 affects gene expression based on assays that measure steady state levels of mRNAs. However, these analyses [1–3] cannot discern effects on transcription from post-transcriptional steps related to RNA metabolism. To assess directly the role of AGO2 in transcription regulation genome-wide, we performed neuRNA-seq in mock versus AGO2 siRNA-treated Kc cells. In brief, this assay relies on the incorporation of 5-ethynyl uridine (EU) into nascent RNA by feeding live cells for 1 h. Next, total RNA is isolated, and biotinylation of EU-labeled RNAs is performed, allowing their selective purification. We found that depletion of AGO2 results in up-regulated nascent transcription of 710 genes and down-regulation of 196 genes (Fig 2A and 2B), suggesting that AGO2 exerts predominantly a negative effect on transcription on a genome-wide level. Previous ChIP-seq analyses performed by two independent groups using the same AGO2 monoclonal antibody (9D6) in different cell lines/tissues showed that AGO2 associates with chromatin preferentially at transcription start sites of active promoters [1,3]. Importantly, we found that 32% of up-regulated genes and 24% of down-regulated genes contain an AGO2 binding site at the promoter in Kc cells. Only 8% of unchanged genes harbor an AGO2 peak (of 4201 total AGO2 peaks); therefore, the up- and down-regulated genes are significantly enriched for AGO2 binding compared to unchanged genes (Fig 2B, Fisher’s exact test (FET), odds ratio = 5.4, p<2.2e-16; odds ratio = 3.3, p<9.8e-11 respectively). These results suggest that observed changes in transcription can be caused by direct transcriptional effects of AGO2, although it remains possible that some changes are a consequence of secondary effects. Furthermore, depletion of AGO2 with an alternative siRNA designed against its 5’UTR showed an 85% correspondence of up-regulated genes (122 of 136 up-regulated genes, FET, odds ratio = 251, p<2.2e-16). Finally, transcriptional effects observed upon depletion of AGO2 are almost completely rescued by the expression of an siRNA-resistant wildtype version of AGO2 (S1 Fig).

**Fig 3:**
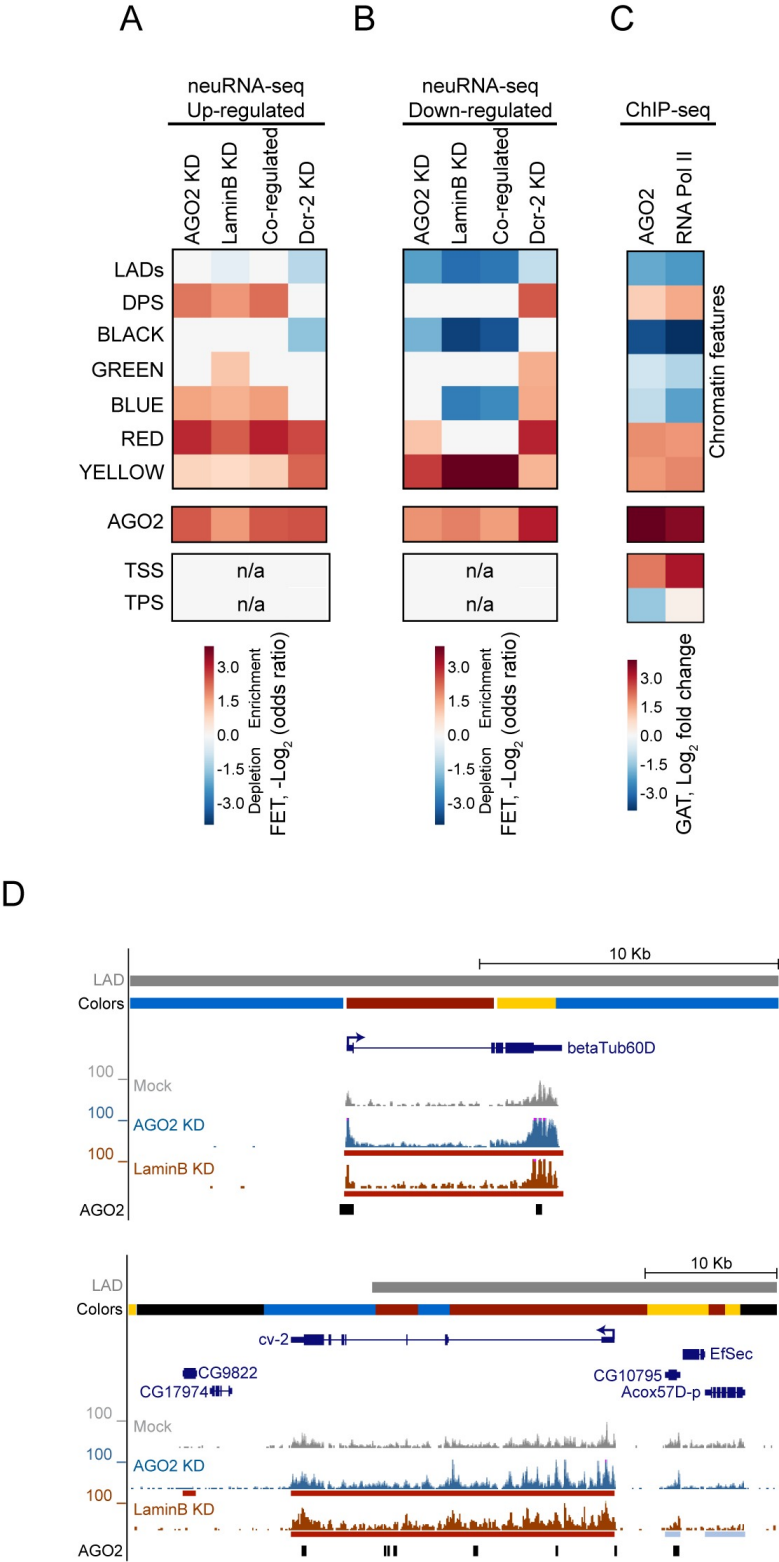
AGO2 and LaminB attenuate transcription in active chromatin and additional sites across the genome. A) Heatmap showing enrichment and depletion of neuRNA-seq up-regulated genes in AGO2-, LaminB, both- or Dcr-2-depleted cells across different chromatin features such as chromatin domains (LADs and DPSs), chromatin colors, transcription start sites (TSS), and transcription polyadenylation site (TPS). The color scale indicates the log_2_ odds ratio of the Fisher's exact tests, where negative (blue) indicates depletion and positive (red) indicates enrichment. B) Heatmap showing enrichment and depletion of neuRNA-seq down-regulated genes in AGO2-, LaminB, both- or Dcr-2-depleted cells with respect to chromatin features. C) Heatmap showing enrichment and depletion of AGO2 and Pol II ChIP-seq peaks with respect to chromatin features. Colormap represents the log_2_ fold change as reported by the Genomic Association Test (GAT). D) Representative screenshot of co-up-regulated genes in AGO2 KD and LaminB KD embedded within a LAD with TSS located in active RED chromatin. The red and blue bars below the neuRNA-seq signals correspond to significant differences (up and down, respectively) relative to mock-treated cells. Black bars at the bottom of each screenshot correspond to AGO2 peaks. See S2 Fig.

Given that AGO2 and LaminB interact with Pol II, we next examined whether LaminB may modulate transcription similarly to AGO2. Therefore, we performed neuRNA-seq upon depletion of LaminB in Kc cells (Fig 2A) and compared AGO2 and LaminB nascent transcriptome profiles. Strikingly, we found that depletion of either of these proteins produced exceedingly similar patterns of gene expression changes (Pearson’s R = 0.53). We observed 466 genes up-regulated in both knockdowns (66% of 710 in AGO2 KD and 33% of 1413 in LaminB KD, FET, odds ratio = 32, p<2.2e-16) and 76 genes down-regulated in both knockdowns (39% of 196 in AGO2 KD and 8% of 926 in LaminB KD, FET, odds ratio = 12, p<2.2e-16, Fig 2C). Genes co-up-regulated by AGO2 and LaminB also show a statistically significant enrichment for AGO2 binding at the transcription start site (TSS) (250 of 466 co-up-regulated genes, FET, odds ratio = 5.6, FDR-corrected p<2.2e-16 Fig 3A) suggesting a direct effect of AGO2. Reciprocal knockdowns of AGO2 and LaminB did not show altered subcellular localization changes for either factor (Fig 2F), indicating that similarities in transcriptional effects are not a result of protein mislocalization.

Since a major function of AGO2 is as an effector of the RNAi pathway, we tested whether changes in transcription due to depletion of AGO2 are related to defects in the RNAi pathway. To this end, we performed neuRNA-seq analysis upon Dcr-2 endonuclease depletion, but we did not observe similar transcriptome changes in comparison with either AGO2 (Fig 2D, R = 0.13) or LaminB knockdown (Fig 2E, R = 0.018). Furthermore, in AGO2-depleted cells expression of the AGO2^V966M^ catalytic slicing mutant defective for RNAi [13] still achieved extensive transcriptional rescue similar to wildtype AGO2 (S1 Fig). Collectively, these results indicate that AGO2 and LaminB attenuate transcription throughout the genome, independently of the RNAi pathway.

### AGO2 and LaminB attenuate transcription predominantly in active chromatin domains genome-wide

In order to characterize genome-wide transcriptional changes dependent on AGO2 and LaminB, we compared neuRNA-seq profiles from AGO2- and LaminB-depleted cells to several functional chromatin features. We first examined LADs, DPSs, and chromatin types as previously classified by color based on chromatin marks in Kc cells [9]. Interestingly, we found that the transcriptional de-repression observed upon depletion of AGO2 takes place mainly in RED (active developmentally regulated) and to a lesser extent, within YELLOW (active housekeeping) and BLUE (Polycomb) chromatin (Fig 3A). Depletion of LaminB shows a similar pattern to AGO2 with an additional mild enrichment of GREEN (heterochromatic) chromatin. We initially expected that AGO2 and LaminB co-up-regulated genes would be enriched in LADs and/or BLACK chromatin, which typically exhibit low levels of transcription. However, we did not find this to be the case (Fig 3A). Accordingly, AGO2 chromatin association itself is not significantly enriched within LADs genome-wide (Fig 3C).

Instead, like AGO2-dependent up-regulated genes, AGO2 and LaminB co-up-regulated genes are most enriched in active RED and to a lesser degree YELLOW and BLUE chromatin. Upon closer visual inspection, we determined that 44 of 94 co-up-regulated gene promoters correspond to RED chromatin yet are positioned inside a LAD (Fig 3D). In fact, the overall enrichment of co-up-regulated genes within RED chromatin occurs both inside and outside LADs (S2A Fig), indicating that this is not a LAD-specific phenomenon. Interestingly, genes whose promoters are in RED chromatin that are also inside a LAD are still expressed under normal conditions but at lower levels than RED chromatin outside of LADs (S2C Fig). Similarly, co-up-regulated genes both inside and outside LADs are associated with AGO2 binding (S2A Fig). In contrast, co-down-regulated genes harboring AGO2 binding at the TSS or located in YELLOW or RED chromatin are positioned outside of LADs (S2B Fig), consistent with LADs being less transcriptionally inactive. In other words, genes that are already silent cannot be further down-regulated. Our analyses indicate that RED chromatin, although typically considered active or at least potentially active, can reside within larger repressive domains such as LADs. We conclude that genes within these regions can then become more active after depletion of either AGO2 or LaminB.

Finally, we tested whether the co-regulated genes are associated with a topology-related chromatin feature such as DPS. We found that genes inhibited by AGO2 or LaminB are highly enriched in DPS, indicating preferential TAD border localization of these genes (Fig 3A and 3B). In contrast, genome-wide patterns of nascent transcription changes due to knockdown of Dcr-2 contrast substantially with that of both AGO2 and LaminB depleted cells (Fig 3A and 3B).

### AGO2 represses transcription of the spermatogenesis gene program

**Fig 4:**
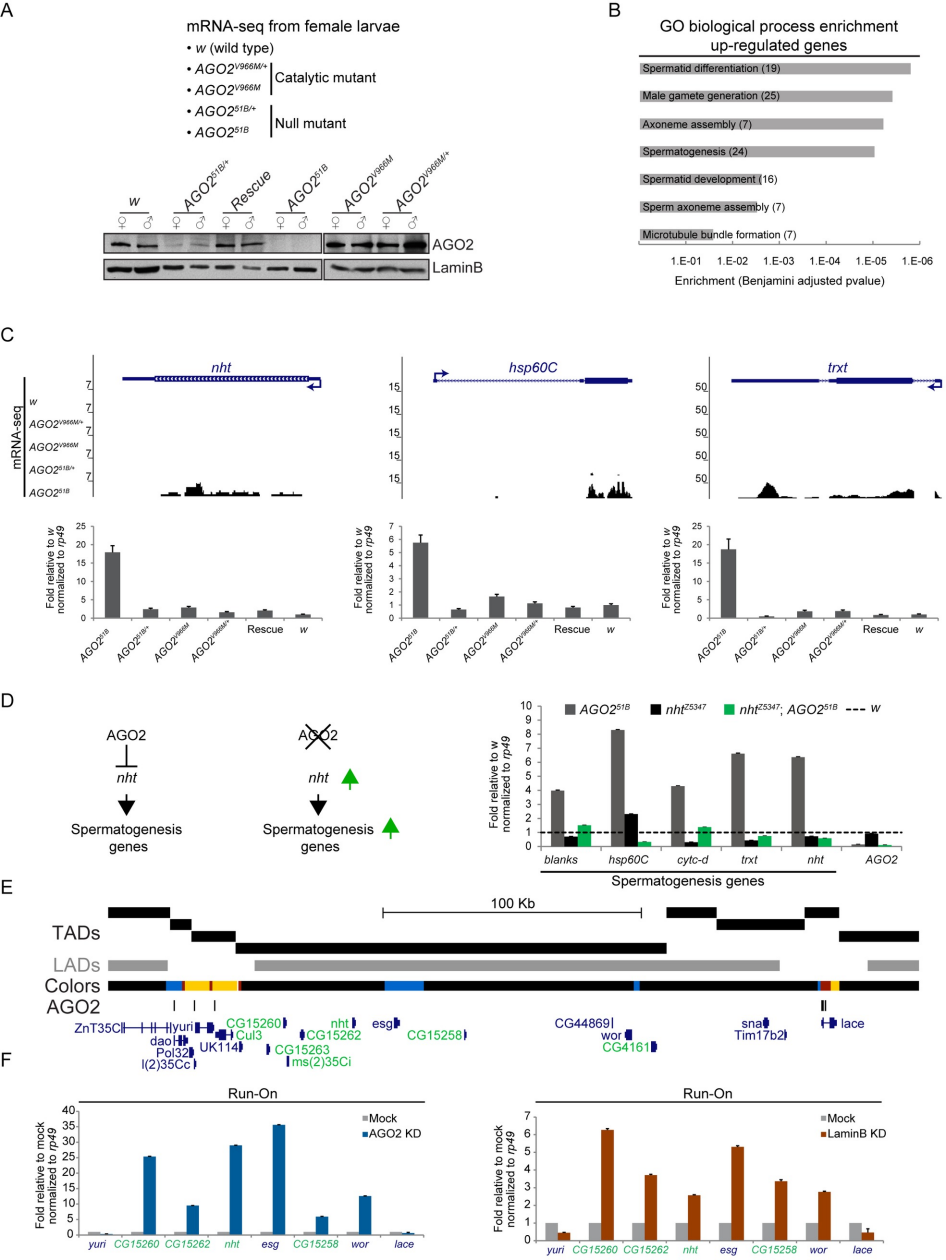
AGO2 represses spermatogenesis gene transcription in somatic cells dependent on *nht*. A) Female third instar larval strains profiled by mRNA-seq. Western blot analysis for AGO2 and LaminB in extracts of females or males of the indicated genotype. Rescue corresponds to a transgenic strain that expresses a genomic copy of wildtype AGO2 in the *AGO2*^*51B*^ null mutant background. See S3A Fig. B) Gene ontology (GO) analysis for up-regulated genes that depend on the presence of AGO2 independently of its catalytic activity (number of annotated genes per category are indicated). C) Examples of genes showing increased mRNA-seq signal (top panels) specifically in *AGO2*^*51B*^ null mutants. Validation by qRT-PCR is shown. Values are relative to *w* strain and normalized to *rp49* as loading control (bottom panels). See S3B, S3C, S3D Fig, and S3 Table. D) Genetic model for *AGO2* repression of spermatogenesis gene expression through *nht* regulation (left panel). Female larvae of indicated genotypes were subjected to qRT-PCR for different spermatogenesis genes. Values are relative to *w* (dashed line) and normalized to *rp49* as loading control (right panel). E) Genome browser view of LADs (grey bar) [11], TADs [8,16], chromatin colors [9] and AGO2 ChIP peaks (black bars) depicting chromatin context in which *nht* is located in Kc cells. Testis-expressed genes are highlighted in green. F) Transcription run-on assay followed by qRT-PCR from isolated permeabilized nuclei upon depletion of AGO2 (left panel) or LaminB (right panel) in Kc cells. Values are relative to mock-treated cells and normalized to *rp49* as loading control. Testis-expressed genes are highlighted in green. Error bars correspond to standard deviation of four technical replicates.

In order to address the physiological role of AGO2-mediated transcriptional regulation, we carried out mRNA-seq from *AGO2* mutant whole female larvae. To discard potential transcriptional effects due to the RNAi pathway, we compared wild type, *AGO2*^*51B*^ null, and *AGO2*^*V966M*^ mutant strains and filtered out effects dependent on AGO2 catalytic activity (Figs 4A and S3). As a result, we identified 396 de-repressed genes dependent on the presence of AGO2 but independent of its catalytic function (S3 Fig). Intriguingly, gene ontology analysis revealed that these up-regulated genes are statistically enriched exclusively for spermatogenesis and related processes (Fig 4B). A large number of genes with unknown function were also up-regulated but could not be assigned to a functional category. We verified up-regulation of several spermatogenesis genes by qRT-PCR analysis (Fig 4C and S3 Table). Similar results were obtained upon AGO2 knockdown of two different somatic cell lines, Kc and D8 (S3 Fig), suggesting that up-regulation of spermatogenesis genes is not due to female-to-male germline transformation of *AGO2* mutants. These results support a role for AGO2 in repression of the spermatogenesis gene expression program in somatic cells derived from a diverse set of tissues and stages of development.

### Up-regulation of spermatogenesis gene expression in *AGO2* mutants is dependent on *nht*

We hypothesized that the up-regulation of spermatogenesis gene expression in *AGO2* null mutants may be due to de-repression of a key upstream transcription factor that is sufficient to activate this developmentally regulated program. The *nht* gene is up-regulated in the *AGO2*^*51B*^ null mutant and expresses a testis specific TBP-associated factor, which activates spermatogenesis gene expression in primary spermatocytes [14]. To test if AGO2 represses spermatogenesis primarily through repression of *nht*, we compared spermatogenesis gene expression by qRT-PCR in female *AGO2*^*51B*^ and *nht*^*z5347*^ single null mutants versus *nht*^*z5347*^; *AGO2*^*51B*^ double null mutants. As expected, *nht*^*z5347*^ single mutants do not ectopically express spermatogenesis genes (Fig 4D). Importantly, up-regulation of spermatogenesis genes caused by the *AGO2*^*51B*^ null mutation is suppressed by the *nht*^*z5347*^ mutation, consistent with the notion that *AGO2* acts as an upstream repressor of *nht* to prevent spermatogenesis gene expression in somatic tissue.

### *nht* is located in a repressed spermatogenesis gene cluster within a LAD

We next examined the chromatin context of *nht* and found that it is embedded within a LAD that is also defined mainly as BLACK chromatin. Furthermore, *nht* is surrounded by several testis-specific genes (Fig 4E, highlighted in green), most of which are up-regulated in the *AGO2*^*51B*^ null mutant but not in the catalytic *AGO2*^*V966M*^ strain (S3 Table). We confirmed that AGO2 and LaminB are required to restrict transcription of *nht* and surrounding genes specifically within the LAD by performing run-on experiments in Kc cells followed by qRT-PCR upon depletion either of AGO2 or LaminB (Fig 4F). We also validated this finding by standard qRT-PCR to examine steady state levels (S3E Fig). Previous studies in *Drosophila* showed that certain testis-specific genes are also organized as clusters and enriched within LADs, and their repression in somatic cells is dependent on LaminB [15]. However, significant changes in radial positioning of the *nht* locus were not observed in AGO2 or LaminB knockdown Kc cells (S4 Fig).

### AGO2 and LaminB modulate chromatin topology of the TAD in which *nht* is immersed

**Fig 5:**
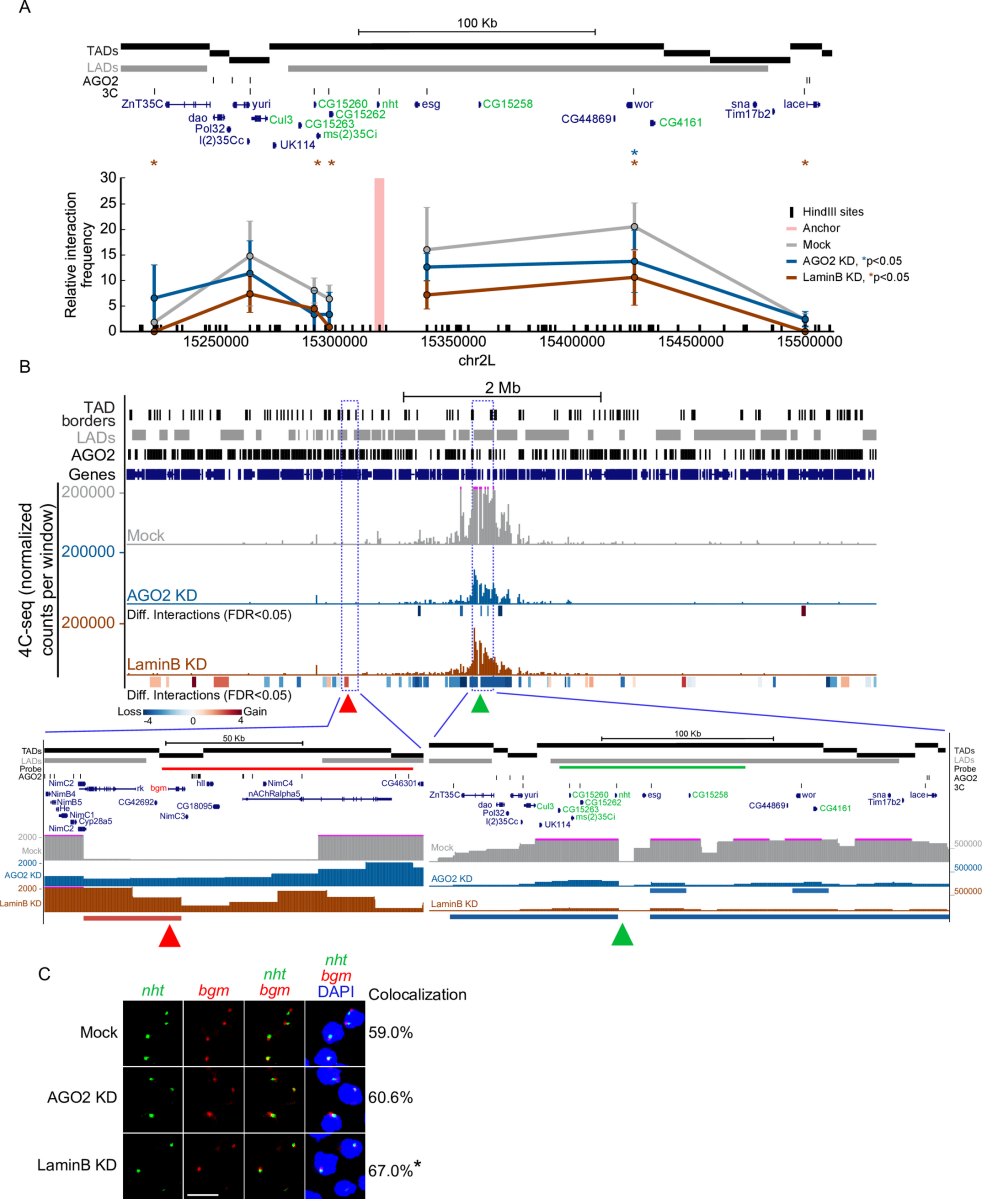
AGO2 and LaminB control chromatin topology of the *nht* TAD as well as surrounding regions. A) 3C analysis between the *nht* promoter as anchor (salmon bar) and different sites located within or at LAD/TAD borders. The *y*-axis represents the relative interaction frequency, and the *x*-axis shows genomic coordinates. LADs are shown in grey. TADs, AGO2 peaks, and 3C primers are shown as black bars. Testis-expressed genes are highlighted in green. Error bars correspond to standard deviation of four experiments. Significant comparisons relative to mock are highlighted with asterisks (Student’s t-test). B) Chromosome-wide view of 4C-seq signal using the *nht* promoter as anchor (green triangle), LADs (grey bars), TAD borders, and AGO2 peaks (black bars). The y-axis corresponds to normalized counts per window. Sites showing statistically significant differences in interaction (FDR<0.05) relative to mock-treated cells upon AGO2 or LaminB depletion are shown as rectangles under the corresponding track with decreased (blue) and increased (red) interaction indicated. Red triangle highlights a distant TAD containing the *bgm* gene that significantly increases its interaction with the *nht* promoter upon depletion of LaminB. Blue lines indicate zoomed-in regions. C) Representative maximal projections of images for dual DNA-FISH using a probe that recognizes the *nht* TAD (green) and an adjacent TAD (termed *bgm*, red) in mock, AGO2- and LaminB-depleted cells. Percentage of colocalization between both probes is indicated on right. Statistically significant value relative to mock is highlighted with an asterisk (LaminB KD, p<2.2e-16 Chi-squared test). Nuclei were stained with DAPI. Scale bar represents 16 μm. See S4 Fig.

Because AGO2 has been shown to promote gene looping in order to activate transcription, we sought to determine whether AGO2 may control overall chromatin topology of the *nht* LAD in concert with LaminB. We observed AGO2 at the borders of the *nht* LAD, which at this genomic region also generally align with TAD borders [8,16] (Fig 5A). To investigate if AGO2 or LaminB controls overall chromatin topology at this region, we performed chromosome conformation capture assays followed by qPCR (3C) using as a bait the *nht* promoter in either mock-treated or Kc cells depleted for AGO2 or LaminB. In mock treated cells, we observed a high level of interaction within the LAD as distant as 100 kb, confirming the highly self-interacting nature of this region. Importantly, we observed a substantial decrease in interaction frequency between the *nht* promoter and different sites tested within the LAD upon depletion of AGO2 or LaminB (Fig 5A). In order to obtain higher resolution, we also performed circular chromosome conformation capture assays (4C-seq) using the *nht* promoter as bait. Consistent with our 3C data, the majority of interactions observed in mock-treated cells are constrained within the LAD in which *nht* is located (Fig 5B). We applied the 4C-ker algorithm [17], which utilizes biological replicates to determine statistically significant differences in interaction frequencies between treatments. We found that depletion of AGO2 or LaminB leads to strong decreases in the frequency of interactions within the *nht* LAD (Fig 5B, p<0.05 for all indicated regions). Strikingly, we also observed increases in interaction between the *nht* promoter with various TADs beyond 1 Mb away, particularly in the LaminB knockdown. We validated by dual DNA FISH the increased colocalization between *nht* and one distant TAD in both knockdowns (referred to as *bgm*, Fig 5C). Collectively, these results support a concerted role for AGO2 and LaminB to transcriptionally repress *nht* by maintaining the LAD in a “locked” repressive structure that may be less conducive to transcriptional activity.

## Discussion

In this study, we employ proteomics, neuRNA-seq, ChIP-seq, and 4C-seq to elucidate a novel genome-wide relationship between *Drosophila* AGO2 and LaminB to function in genome organization and thereby affect gene regulation. By examining *AGO2* null mutants, we were able to focus on a single somatic target of AGO2 repression, *nht*, which is a key activator of spermatogenesis genes. The *nht* locus is immersed in a LAD located within a repressive TAD, which is flanked by AGO2 binding sites. Finally, we found that depletion of either AGO2 or LaminB results in a significant decrease in frequency of interactions within the TAD as well as increases in inter-TAD interactions. We conclude that AGO2 and LaminB can work in concert to regulate gene expression by orchestrating overall genome organization.

### LaminB and AGO2 preferentially modulate transcription of active domains

We found that the majority of genes for which transcription is altered in AGO2- or LaminB-knockdowns are normally actively transcribed, but we observed specificity of this effect. In particular, up-regulated genes are enriched in RED compared to YELLOW chromatin, while down-regulated genes show the opposite pattern. Although AGO2 is present in both types of active chromatin, one major difference between these two active chromatin states is the absence of histone H3K36me3, a hallmark of elongation, in RED chromatin [9]. Therefore, depletion of AGO2 may preferentially relieve attenuation of RED versus YELLOW chromatin. Furthermore, YELLOW chromatin generally corresponds to constitutively expressed genes, whereas RED is characteristic of developmentally regulated genes. To our surprise, we found that a substantial number of co-up-regulated genes within RED chromatin were distributed equally between LADs and non-LADs. Perhaps these RED domains are located within a subclass of LADs that allow gene expression at a certain level, likely to more efficiently respond to stimulus or developmental signals. Further analysis would be required in order to establish sub-classification of LADs in *Drosophila* and analyze their potential function, as has been recently performed for the mouse genome [18]. Overall, the presence of RED chromatin within a LAD could correspond to an additional layer of regulation of developmentally controlled gene expression.

Another non-exclusive possibility is that topological constraints imposed by AGO2 differentially affect the two chromatin types. For example, restriction of tissue-specific enhancer-promoter interactions present in RED chromatin might be relaxed by changes in topology while housekeeping genes could become ectopically subject to repression by surrounding chromatin from which it is normally insulated. Our results suggest that AGO2 can exert either positive or negative effects on transcription depending on the chromatin context.

### Spermatogenesis genes are repressed in somatic tissue by AGO2 and LaminB

While transcription changes observed in AGO2 and LaminB knockdowns genome-wide are most enriched for actively transcribed regions, some normally inactive or potentially active regions including within LADs, are also up-regulated. One such gene that is subject to tight tissue-specific regulation and repressed by AGO2 and LaminB is *nht*, which normally activates spermatogenesis specifically in primary spermatocytes. Since mRNA-seq profiling of *AGO2* mutants was performed on whole female larvae, detection of up-regulation of normally silent genes was favored. Previous work showed that certain spermatogenesis gene clusters are associated with the nuclear periphery in somatic cells and become de-repressed and repositioned toward the interior upon depletion of LaminB [15]. However, we did not observe a significant detachment of *nht* from the nuclear periphery upon depletion of AGO2 or LaminB. These results are in agreement with recent work showing that LaminB is not required to a large extent for LAD structure in mES cells [19]. In AGO2- or LaminB-depleted somatic cells, *nht* partially escapes from repression, although it is not fully activated likely due to its presence in a silent BLACK domain and the absence of other required factors. It is important to note that despite being able to detect higher levels of a variety of spermatogenesis transcripts in AGO2- and LaminB-depleted somatic cells, we did not observe increased protein levels. Strict regulation of spermatogenesis gene expression also occurs on the post-transcriptional level [20] and helps ensure no phenotypic consequences when transcription becomes dysregulated.

### Gene expression changes in AGO2- and LaminB-depleted cells may be caused by changes in chromatin topology

**Fig 6:**
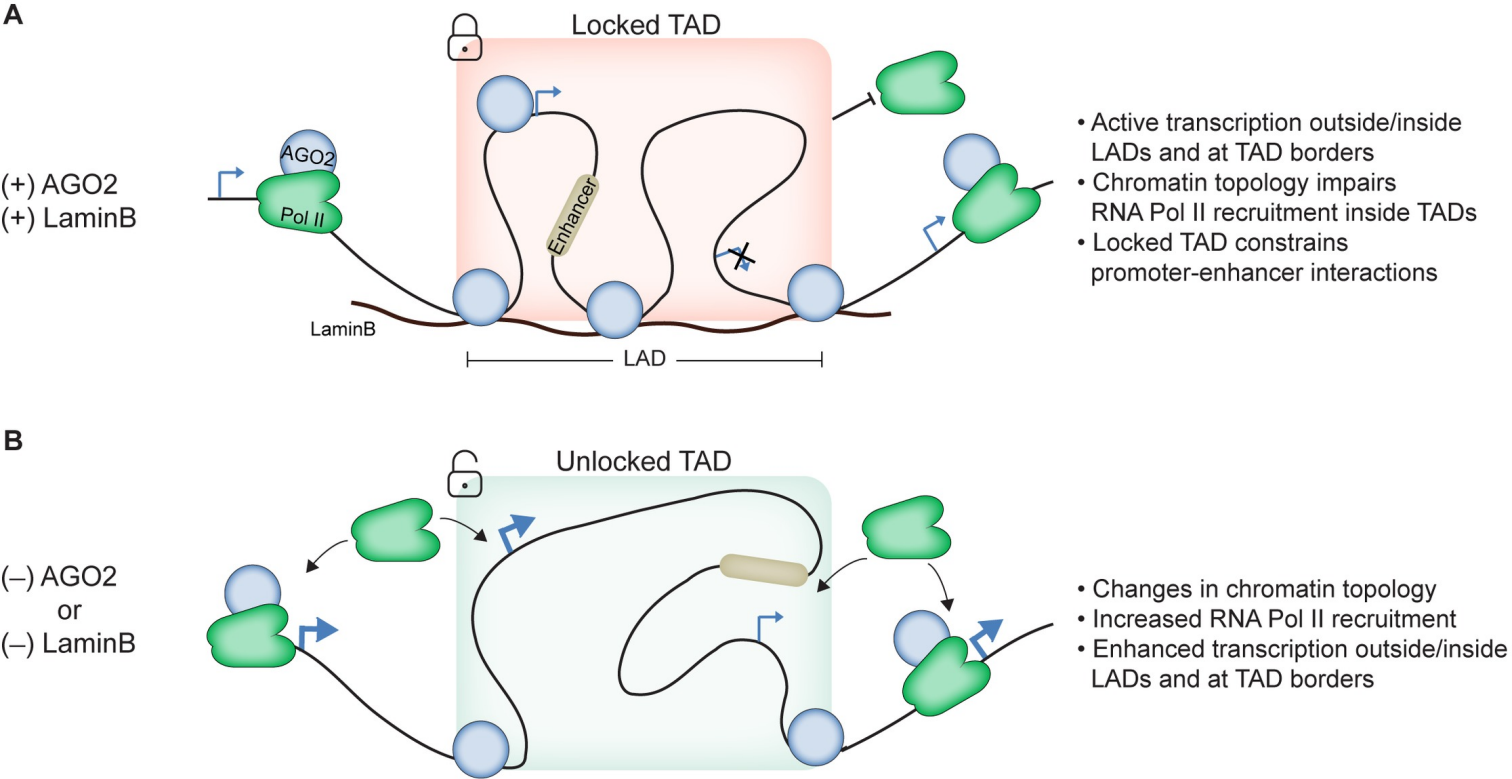
A model for enhanced transcription upon depletion of LaminB or AGO2. A) AGO2 associates with genes both outside and inside the LAD/TAD. LaminB scaffolding helps maintain the TAD in a locked structure, likely impairing Pol II recruitment and preventing promoter-enhancer interactions across the TAD border. B) Upon depletion of either AGO2 or LaminB the chromatin topology from the LAD/TAD switches from a locked to a more accessible unlocked structure, thus permitting inter-TAD interactions. As a result, Pol II recruitment and promoter-enhancer interaction may be increased.

We found that AGO2 and LaminB control the overall chromatin topology of the LAD and TAD in which *nht* is located, and these changes could be a key driver of observed transcriptional effects in AGO2- and LaminB-depleted cells. In the case of *Abd-B*, which is located outside of a LAD, AGO2 functions in concert with the insulator proteins CTCF and CP190 to promote or stabilize looping between the *Abd-B* promoter and *iab-8* enhancer region, thus stimulating transcription [1,20]. However, AGO2 itself does not associate with the *nht* promoter, so it is unlikely that AGO2 directly prevents the *nht* promoter from looping to nearby enhancers. Rather, AGO2 localizes to borders of the LAD/TAD encompassing *nht*, suggesting that it may play a larger role in constraining topology of the entire region. In fact, our 3C and 4C-seq analyses show that interactions between *nht* and other sites within the TAD decrease in interaction frequency in both AGO2- and LaminB-depleted cells. At the same time, increased interactions are observed between the *nht* promoter and sequences located in other chromatin domains beyond 1 Mb away. In this structural view, AGO2 appears to function in a manner hypothesized for insulator proteins at TAD borders [7]; however, no single insulator protein flanks this LAD/TAD. Large alterations in topology in AGO2- and LaminB-depleted cells could explain the resultant transcriptional increase of *nht* by allowing interaction with inappropriate enhancers or otherwise creating a more permissive transcriptional environment (Fig 6). Our results are in agreement with recent work performed in mouse showing that the absence of lamins decreases inter-TAD interactions within constitutive LADs and increases inter-TAD interactions between TADs inside LADs and TADs outside LADs [21]. Importantly, this topology remodelling correlates with changes in gene expression, overall suggesting an evolutionary conserved role of lamins to regulate transcription by controlling chromatin topology.

Contrary to the expectation that LaminB simply represses transcription within LADs, we found that its depletion also substantially affects transcription of active chromatin outside of the LAD. In line with our findings, microarray and RNA-seq analysis from mouse LaminB knockout embryonic stem cells and trophectoderm cells showed low correlation between the genes changed in expression and LaminB-bound genes, indicating that lamins do not repress transcription within LADs genome-wide [19,22]. Traditionally, LADs were viewed as chromatin platforms enriched in transcriptional repressors and repressive histone marks [23], thus creating an inactive chromatin environment impairing access to transcriptional activators [24]. However, we observed that AGO2 and LaminB co-repress active or potentially active promoters within LADs. In many cases the TSS of these genes are found within annotated RED chromatin inside the LAD, suggesting that LaminB does not serve as a simple barrier to the transcription machinery. Our results are more consistent with the interpretation that AGO2 and LaminB help prevent spurious interactions between active or potentially active regions. Such changes in topology could be achieved by disruption of LAD borders and/or decrease of the interaction frequency of sites within the LAD itself in favor of inter-LAD interactions. Alternatively, LaminB could still be present in inter-LAD boundaries in a sparse binding pattern that would not be defined as a classic LAD. This pool of LaminB would be affected by, and perhaps be more susceptible to, LaminB depletion which would result in the inter-LAD transcription effects we observe. Overall, our findings reveal a coordinated function for AGO2 and LaminB in regulating gene expression by controlling genome architecture.

## Materials and methods

### Fly stocks

Flies were maintained on standard cornmeal medium at room temperature or 25°C. Larvae for qRT-PCR and western blotting were raised at 25°C. *AGO2*^*51B*^ and *AGO2*^*V966M*^ mutants were described in [1,13], respectively. *AGO2* wild-type genomic rescue was previously described in [25].

### Cell lines

Kc167 cells were grown in CCM3 media (Thermo Scientific HyClone). Cells were maintained at 25°C. To produce a similar analysis of the larval mutants but in Kc cells, we attempted to generate *AGO2* mutants by CRISPR technology with no successful results perhaps because of the tetraploid nature of the Kc cell line and/or its sensitivity to the clonal dilution procedure.

### dsRNA and siRNA knockdowns

Amplicons used for dsRNA knockdowns were designed based on recommendations from the *Drosophila* RNAi Screening Center. Templates were PCR-amplified from genomic DNA using primers containing the T7 promoter sequence. In vitro transcription of PCR templates using the MEGAscript T7 kit (Ambion) was used to produce dsRNAs, and these were purified by phenol-chloroform extraction. Transfections using 2 μg of dsRNA or 100 pmol of siRNA, or no dsRNA/siRNA as mock treatment, were performed using Cell Line Nucleofector kit V (Amaxa Biosystems) transfection reagent using the G-30 program. Three days after transfection, cells were collected and knockdown efficiency was confirmed by Western blotting.

### AGO2 mutant rescues

To generate AGO2-rescues, AGO2 cDNA was cloned into p-ENTR/D-TOPO gateway vector (Invitrogen). Site-directed mutagenesis was carried out to obtain the AGO2^V966M^ mutant. These constructs were recombined into the pAFHW (Carnegie vectors) expression vector to generate N-terminal 3xFlag, 3XHA fusion constructs. Each construct was co-transfected with an siRNA designed against the 5’UTR of AGO2.

### AGO2 immunoaffinity purification and mass spectrometry

Nuclear extracts from 11 g of 0–24 h OR embryos were prepared as previously described [26] and lysed in 2.5 mL HBSMT-0.3% + 1 M KCl (50 mM HEPES, 150 m MNaCl, 1 M KCl, 3 mM MgCl_2_, 0.3% Triton X-100 [v/v] at pH 7) including 1 mM PMSF, and Complete protease inhibitor cocktail (Roche). For AGO2 immunoaffinity purification, 6 mL of 9D6 tissue culture supernatant was covalently crosslinked to rProtA sepharose beads using 20 mM dimethylpimelimidate in 0.2 M sodium borate, pH 9.0 quenched with 0.2 M ethanolamine, pH 7.9. For control sample, 2.4 μg normal mouse IgG (Santa Cruz) was used. Crosslinked beads were incubated with 5.5 mg nuclear lysate overnight at 4°C and washed as described previously [1]). Samples were eluted in formic acid, trypsin digested, subjected to ultraperformance liquid chromatography on a NanoAcquity system (Waters), and analyzed on an LTQ-Orbitrap-Velos system (Thermo) at the NIDDK Mass Spectrometry Facility. Results were analyzed by Mascot algorithm (Matrix Science).

### Chromatin fractionation

Chromatin fractionation was performed according to [2,27] with minor modifications. In brief, approximately 1–2 X 10^7^ Kc cells were washed twice with cold PBS. Cells were lysed for 15 min on ice in cold CSKI buffer (10 mM PIPES pH 6.8, 100 mM NaCl, 1 mM EDTA, 300 mM sucrose, 1 mM MgCl_2_, 0.5% (v/v) Triton X-100 supplemented with 1 Mini Complete tablet (Roche) per 10 mL lysis buffer. Cell lysate was divided into two portions, which were centrifuged at 500 x*g* at 4°C for 5 min. The supernatants (S1 fraction), which contain Triton-soluble proteins, were further analyzed. One of the pellets (P1 fraction) was washed twice in CSKI buffer and then resuspended in RIPA buffer (150 mM Tris-HCl pH 8, 150 mM NaCl, 0.5% sodium deoxycholate, 0.1% (w/v) SDS, 1% (v/v) NP-40). The second pellet, after washing in CSKI buffer, was resuspended in CSKII buffer (10 mM PIPES pH 6.8, 50 mM NaCl, 300 mM sucrose, 6 mM MgCl_2_), then treated with 10 U DNase I (Roche) for 30 min and extracted with 250 mM NH_2_SO_4_ for 10 min at 25°C. The sample treated with DNase I and salt was then centrifuged at 1,200 x*g* for 5 min at 4°C, and the supernatant (S2 fraction) and pellet (P2 fraction) were collected. The P2 fraction was also resuspended in RIPA buffer. All fractions were analyzed by western blotting.

### Co-immunoprecipitation

Approximately 1 X 10^8^ cells were harvested and washed twice with cold PBS. Cells were lysed for 5 min on ice in lysis buffer (Tris-HCl pH 7.4 30 mM, 100 mM NaCl, 10 mM MgCl_2_, 0.1% Triton X-100) supplemented with Complete EDTA free protease inhibitors (Roche) and centrifuged for 5 min at 500 x*g* at 4°C. The supernatant was removed and pellets were washed once with lysis buffer, resuspended in IP buffer (50 mM Tris-HCl pH 7.4, 150 mM NaCl, 0.3 mM MgCl_2_, 0.3% Triton X-100), incubated 10 min on ice and sonicated 1 cycle for 10 sec, to shear DNA (nuclear fraction). Nuclear extracts were incubated with normal mouse serum or a monoclonal antibody against LaminB (ADL67.10) from Developmental Studies Hybridoma Bank (DSHB), and allowed to bind for 1 h at 4°C. Next, 50 μL of prewashed Protein G beads 50% slurry was added. After incubation for 2 h, unbound supernatant was removed, and the beads were washed once in IP buffer and twice in TBS (50 mM Tris-HCl pH 7.4, 150 mM NaCl). The bound protein was eluted in sample buffer by boiling, separated by using SDS-PAGE, transferred to nitrocellulose in 10 mM CAPS, pH 11 and detected by western blotting. Proteins were detected with the SuperSignal substrate (Pierce).

### neuRNA labelling and library preparation

neuRNA labeling and capture were performed with Click-iT Nascent RNA Capture Kit (Thermo Fisher Scientific) according to manufacturer’s protocol with minor modifications. Cells were incubated with EU at 0.2 mM for 1 h and RNA was extracted with TRizol (Thermo Fisher Scientific). The Click-iT reaction was performed with 0.25 mM biotin azide, and biotinylated RNA was captured with 12 μL T1 beads. Nascent RNA was used to generate RNA-seq libraries with Ovation RNA-seq Systems 1–16 for Model Organisms (Nugen). All samples were sequenced with HiSeq2500 (Illumina) at the NIDDK Genomics Core Facility by 50 bp single-end sequencing.

### mRNA-seq from larvae

Whole female larvae were collected and total RNA extracted with TRizol (Invitrogen). Polyadenylated RNA and ribosomal (rRNA)-depleted RNA was purified from total RNA using the MicroPoly(A)Purist Kit (Ambion) and the RiboMinus Eukaryote Kit for RNA-seq (Invitrogen), respectively. Sequencing libraries were prepared from Poly(A)^+^ and rRNA-depleted RNA samples according to the manufacturer’s protocol (Illumina). All samples were sequenced with HiSeq2500 (Illumina) at the NIDDK Genomics Core Facility by 50 bp single-end sequencing.

### ChIP

Approximately 1–2 X 10^7^ cells were fixed by addition of 1% formaldehyde to cell media for 10 min at RT with gentle agitation. Formaldehyde was quenched by addition of glycine to 0.125 M with gentle agitation for 5 min at RT. Cells were pelleted at 2000 x*g*, washed twice in PBS, and resuspended in 0.8 mL ice–cold cell lysis buffer (5 mM PIPES pH 8, 85 mM KCl, 0.5% NP-40) supplemented with Complete protease inhibitors (Roche), incubated on ice 10 min pelleted by centrifugation at 2000 x*g* for 5 min at 4°C. Next, the supernatant was removed and pellets were resuspended in 1 mL nuclear lysis buffer (50 mM Tris-HCl pH 8, 10 mM EDTA.Na2, 1% SDS) and incubated for 10 min at 4°C. Afterwards, 0.5 mL of IP dilution buffer was added (16.7 mM Tris-HCl pH 8, 1.2 mM EDTA, 167 mM NaCl, 1.1% Triton X-100, 0.01% SDS) and chromatin was fragmented to an average size of 300 bp by using PicoBioruptor (Diagenode) using 10 cycles of 30 s on plus 30 s off, maximum output. Samples were centrifuged at max speed for 10 min at 4°C, and the supernatant (sheared chromatin) was saved at -80°C. Chromatin was diluted to 1:5 with IP buffer, and the assayed antibody in addition to 50 μL of prewashed protein A/G 50% slurry was added and rotated overnight at 4°C. The next day, beads were washed as follows:

- 3X Low salt IP dilution buffer (20 mM Tris-HCl pH 8, 2 mM EDTA.Na2, 150 mM NaCl, 1% Triton X-100, 0.1% SDS).
- 3X High salt IP dilution buffer (20 mM Tris-HCl pH 8, 2 mM EDTA.Na2, 500 mM NaCl, 1% Triton X-100, 0.1% SDS).
- 2X times LiCl buffer (10 mM Tris-HCl pH 8, 1 mM EDTA.Na2, 250 mM LiCl, 1% NP-40, 1% DOC).

Chromatin was eluted twice with 200 μL of elution buffer (500 μL of 1M NaHCO_3_, 250 μL of 20% SDS, 4.25 mL dH_2_O) for 30 min at 65°C each and further incubated overnight at 65°C with 38 μL of decrosslinking solution (20 μL of 5M NaCl, 8 μL of 0.5M EDTA, 10 μL of 1M Tris-HCl pH 8). After de-crosslinking, samples were treated with Proteinase K for 2 h at 50°C and then combined with 1 vol of phenol/chloroform/isoamyl alcohol (25:24:1), vortexed 15 s, and centrifuged for 5 min at 10,000 x*g*. The top layer was transferred to a new tube, and the procedure was repeated using 1 vol chloroform. The top layer was collected and subsequently precipitated with 0.1 vol of 3M NaOAc pH 5.2 and 2.5 vol of 100% ethanol supplemented with 2 μL of Glycoblue (Ambion). After incubating 30 min at -80°C, samples were centrifuged 20 min at 4°C at 10,000 x*g*. Pellets were washed with 70% ethanol and centrifuged 5 min at 4°C at 10,000 x*g*. Pellets were air dried at RT prior to resuspension in 10 μL of dH2O. Samples for ChIP-seq were prepared according to the manufacturer’s protocol with Clontech or TruSeq adapters (Illumina). All samples were sequenced with HiSeq2500 (Illumina) at the NIDDK Genomics Core Facility by 50 bp single-end sequencing.

### qRT–PCR

Total RNA was isolated from cells using TRizol reagent (Invitrogen) following the manufacturer's protocol. Reverse transcription of 0.5–1 μg of total RNA was performed using oligo(dT) or random hexamer as primers and SuperScript III reverse transcriptase (Invitrogen) using the manufacturer's protocol. Transcript levels were quantified in the linear amplification range by real-time PCR using HotStart-IT SYBR green qPCR Master Mix (USB Corporation) by calibration to a standard curve of genomic DNA to account for differences in primer efficiencies.

### Nuclear Run-On

Run-On was performed according to [27] with minor modifications. Nuclei were prepared as follows: cells were centrifuged and washed twice with PBS. The pellet was resuspended in cell lysis buffer (10 mM Tris-HCl pH 7.4, 3 mM MgCl_2_, 10 mM NaCl, 150 mM sucrose, and 0.5% Nonidet P-40 (NP-40)), and a 5 min incubation on ice followed. Nuclei were then collected by centrifugation (4°C, 500 x*g*) and gently washed with cell lysis buffer devoid of NP-40. After centrifugation, the pellet was resuspended in freezing buffer (50 mM Tris-HCl pH 8, 40% glycerol, 5 mM MgCl_2_ and 0.1 mM EDTA). One volume of transcription buffer (200 mM KCl, 20 mM Tris-HCl pH 8, 5 mM MgCl_2_, 4 mM dithiothreitol (DTT), 4 mM each of rATP, rGTP and rCTP, 200 mM sucrose and 20% glycerol) was gently added to nuclei in ice, 8 μL 10 mM biotin-16-UTP (Roche) was supplied to the mixture, which was incubated for 30 min at 29°C. Reaction was stopped by adding 250 mM CaCl_2_, 6 μL RNase-free DNase I (10 U/mL; Roche) and incubating for 15 min at 29°C. RNA purification of nuclear Run-On RNA was performed with TRizol reagent (LifeTechnologies) according to manufacturer’s instructions. Dynabeads M-280 (50 μL; Dynal, A.S., Oslo, Norway) resuspended in binding buffer (10 mM Tris-HCl, pH 7.5, 1 mM EDTA and 2 M NaCl) were mixed to an equal volume of Run-On RNA and incubated 20 min at 42°C and 2 h at room temperature. Beads were separated by the magnetic apparatus and then washed for 15 min in 500 μL 15% formamide and twice with 2X standard saline citrate (SSC), followed by a 5 min wash in 1 mL 2X SSC. Beads were then resuspended in DEPC-treated water and processed for qRT-PCR using random hexamers.

### Chromosome conformation capture (3C)

Crosslinking was performed by adding formaldehyde directly to the media at a final concentration of 1% and incubating for 10 min at RT. Reactions were quenched by adding glycine to a final concentration of 0.125 M and incubating for 5 min at RT. Reactions were incubated on ice 5 min followed by centrifugation at 1200 x*g* at 4°C for 5 min. Cells were then washed with 5 mL of cold PBS and centrifuged at 1200 x*g* at 4°C for 5 min. Lysis was performed by incubating cells in 1 mL of Lysis buffer (10 mM NaCl, 0.2% NP-40, 10 mM Tris pH8, 1 Mini Complete tablet (Roche) per 10 mL lysis buffer) at 37°C for 20 min. Samples were then centrifuged at 4200 x*g* at 4°C for 5 min, and the lysis step was repeated once. 800 μL of Lysis buffer was added, and samples were incubated at 37°C for 20 min. Samples were then centrifuged at 4200 x*g* at 4°C for 5 min, and resuspended in 1 mL Lysis buffer and incubated another 20 min at 37°C. Pellets from cell samples were then washed with 0.8 mL of digestion buffer [(0.2% NP-40, 1X NEBuffer 3 (NEB)] and centrifuged at 4200 x*g* at 4°C for 5 min. Nuclei were resuspended in 1.6 mL of digestion buffer with SDS added to a final concentration of 0.1% and incubated at 65°C for 30 min. Triton X-100 was added to a final concentration of 1% and samples incubated at 37°C for 15 min. A 40 μL aliquot of the sample was taken and used as the undigested control. The remaining sample was digested with 1600 U of *HindIII* (NEB) at 37°C overnight. Samples were incubated 20 min at 65°C to inactivate *HindIII*. A 40 μL aliquot of the sample was taken here and used as the digested control. The remaining sample was then diluted to 4 mL with ligation buffer [final concentrations were 1% Triton X-100, 1X T4 DNA Ligase Reaction Buffer (NEB)] and incubated at 37°C for 30 min. After the addition of 4800 U of T4 DNA Ligase (NEB), each sample was incubated at 16°C overnight. Proteinase K was added to all samples including the controls. Samples were then incubated at 65°C overnight to reverse crosslinking. After de-crosslinking, samples were combined with 1 vol of phenol/chloroform/isoamyl alcohol (25:24:1), vortexed 15 s, and centrifuged for 4 min at 10,000 x*g*. The top layer was transferred to a new tube, and the procedure was repeated using 1 vol chloroform. The top layer was collected and subsequently diluted with 1 vol of water. The sample was combined with 0.1 vol of 3M NaOAc pH 5.2 and 2.5 vol of 100% ethanol, supplemented with 2 μL of Glycoblue (Ambion). After incubating 1 h at -80°C, samples were centrifuged 20 min at 4°C at 10,000 x*g*. Pellets were washed with 75% ethanol and centrifuged 5 min at 4°C at 10,000 x*g*. Pellets were air dried at RT prior to resuspension in 350 μL water. Loading adjustment was performed by SYBR green quantitative PCR to the *blanks* locus, and samples were adjusted accordingly before TaqMan quantitative PCR for 3C [28]. The values from the qPCR were quantified relative to a standard curve generated using BACs covering the analyzed region (CH321-63B06, CH321-76H10, CH321-88N10, CH321-76C01). BACs were obtained from BACKPACK.

### Circular chromosome conformation capture (4C)

In brief, the first steps correspond exactly to the 3C protocol described above. After resuspending the final pellet with 350 μL of dH2O, 300 μL of the 3C library was digested with 5 μL of DpnII (50 U, NEB) overnight at 37°C. After the enzyme was inactivated, digested chromatin was re-ligated using 20 μL of ligase (100 U, NEB) in a total volume of 8 mL ligation reaction. Finally purification of the library was carried out by adding 1 vol of phenol/chloroform/isoamyl alcohol (25:24:1), vortexed 15 s, and centrifuged for 4 min at 10,000 x*g*. The top layer was transferred to a new tube, and the procedure was repeated using 1 vol chloroform. The top layer was collected and precipitated with 0.1 vol of 3M NaOAc, pH 5.2 and 2.5 vol of 100% ethanol supplemented with 7 μL of Glycoblue. Inverse PCR was carried out using primers containing a 6 nucleotide barcode and one universal forward primer to produce Illumina-4C-seq libraries. PCR amplification was carried out using Phusion High Fidelity DNA Polymerase (NEB) with 60–70 ng of DNA and 1 μL of forward primer (10mM) and 1 μL of reverse primer (10mM). Amplification program: 95°C for 3 min, 30 cycles of 98°C for 20 s, 50°C for 1 min and 72°C for 2 min; final elongation step was 72°C for 5 min. After pooling together 3 PCR reactions, samples were purified using Agencourt AMPure XP beads (Beckman Coulter). Finally, 4C-PCR products were sequenced with HiSeq2500 (Illumina) at the NIDDK Genomics Core Facility by 50 bp single-end sequencing.

### Immunofluorescence

Cells were centrifuged 1 min at 2000 xg, washed twice in PBS, allowed to settle on poly-L-lysine-coated slides and fixed in 4% paraformaldehyde (PFA) in PBS at RT for 10 min. Cells were washed twice with PBS, permeabilized and blocked with 0.5% Triton X-100, 1% Bovine serum albumin, 2% goat normal serum in PBS for 1 h at RT. After blocking, cells were first incubated with primary antibody diluted in 0.1% Triton X-100 and 1% BSA in PBS for 1 h at RT and then washed 3 times with PBS. Afterwards, slides were incubated with secondary antibodies diluted in 0.1% Triton X-100 and 1% BSA in PBS for 1 h at RT and washed three times with PBS. Primary antibodies were rabbit anti-AGO2 1:1000 (Liu) or mouse anti-LaminB 1:1000 (ADL67.10, DSHB). Secondary goat antibodies labeled with AlexaFluor 488 or AlexaFluor 594 (Molecular Probes) were used at 1∶1000. Finally, cells were stained in 1 μg/mL DAPI (Molecular Probes) prepared in PBS and mounted using ProLong Gold (Life Technologies).

### DNA-FISH

Cells were fixed in 4% PFA in PBS for 10 min, washed twice with PBS, permeabilized in 0.5% Saponin (Sigma Aldrich)/0.5% Triton X-100/PBS for 20 min at RT and incubated in 0.1 N HCl for 15 min at RT. Afterwards, cells were washed twice with 2X SSC and treated with RNase A (100 ug/mL) in 2x SSC 1 h at RT. After washing twice with 2X SSC, cells were kept in 50% formamide/2X SSC for at least 30 min at RT. BAC DNAs were purchased from BACPAC (CHORI: CH321-76H10 and CH321-28E07). DNA was used directly as a template in a nick translation reaction including fluorescently labeled dUTPs (ARES kit, Molecular Probes). A probe mix containing 200–400 ng of each fluorescently labeled probe, 100 μg/mL yeast tRNA (Ambion), 100 μg/mL salmon sperm in 10 μL of hybridization buffer (10% dextran sulfate, 50% formamide, 2x SSC, 1% Tween 20) was first incubated 10 min at 80°C and put on ice, then added to cells incubated at 78°C for 3 min and left to hybridize at 37°C overnight. Excess probe was washed three times with each: 1X SSC and 0.1x SSC at 42°C for 5 min. Finally, cells were stained in 1 μg/mL DAPI (Molecular Probes) prepared in PBS and mounted using ProLong Gold (Life Technologies).

### Image analysis

Cells were imaged on a Zeiss LSM 780 confocal microscope using the 63X oil objective. Image stacks of 8–10 images at steps of 1 μm were acquired. First, images from the same field of view and channel were maximally projected. Colocalization and radial measurements to nuclear periphery were performed using CellProfiler software and the pipelines for “colocalization” and “speckle” analysis, respectively. For radial analysis, nuclei were subdivided starting from the center in concentric rings using the DAPI channel and radial distance between objects (nucleus and probe, centroid distances), normalized to nucleus diameter, was calculated. The normalized radial position of the FISH signal was calculated at the spot center.

### RNA-seq (mapping and read counting)

For neuRNA-seq and mRNA-seq, raw reads were trimmed with cutadapt [29] v 1.10 to remove any adapters and poly-A tails while performing light quality trimming using parameters "—quality-cutoff = 20—minimum-length = 25—overlap = 10 -a AAAAAAAAAAAAAAAAAAAAAAAAAAAAAAAAAAAAAAAAAAAAAAAAAAAAAAA -a TTTTTTTTTTTTTTTTTTTTTTTTTTTTTTTTTTTTTTTTTTTTTTTTTTTTTTT -a AGATCGGAAGAGC". Trimmed reads were mapped to the primary chromosomes (2L, 2R, 3L, 3R, 4, X, Y, and mitochondrion_genome) of the FlyBase release 6.11 dm6 assembly with HISAT2 2.0.4 [30] using default parameters. Reads were counted in annotated genes in FlyBase release 6.11 using featureCounts v1.5.0-p3 [31]. Since neuRNA-seq enriches for nascent RNA and the libraries are stranded, we counted reads in full gene bodies using featureCounts parameters "-s 1 -t gene -g gene_id -f". For the unstranded mRNA-seq libraries, we counted reads in exons using parameters "-t exon -g gene_id".

### RNA-seq (differential expression)

For neuRNA-seq, counts tables were loaded into DESeq2 v1.10.1 [32]. Counts tables from independent neuRNA-seq experiments (siAGO2/dsLaminB/mock; dsDcr-2/mock; rescue constructs) were independently imported and normalized using simple design "~treatment" and using otherwise default parameters. Differentially expressed genes were those with an adjusted p-value < 0.1.

For mRNA-seq, the older libraries were originally analyzed with an older set of programs and algorithms (specifically DESeq v1 [33] instead of DESeq2). A major difference is that DESeq2 shrinks the log2 fold change estimates for genes with low information (i.e., low number of counts or high variance across replicates). Notably, *nht* is expressed at very low levels in both WT and AGO2 mutant flies. DESeq v1 detects *nht* as statistically significantly up-regulated in *AGO2* mutants. The AGO2 catalytic-independent gene list, GO analysis and follow-up experiments were based on this original observation. However, we found that *nht* is not significantly differentially expressed using DESeq2. Since follow-up RT-PCR confirmed that *nht* is in fact up-regulated in AGO2 mutants, we conclude that DESeq2 gives a false negative for *nht* likely because of its low expression, consistent with the known conservative behavior of DESeq2 in calling differentially expressed genes especially at low expression levels.

### RNA-seq (expression estimates)

We estimated gene expression by running Salmon v0.8.1 [34] on an index built from the transcriptome FASTA file from FlyBase (Release 6.11) using a *k* of 25 bp for our data containing 50 bp reads, and using default parameters in addition to the parameter "-l SF". For each gene, the reported transcript-level expression estimates in transcripts per million (TPM) were summed to give a total TPM value for each gene. Gene TPMs were averaged within replicates of each experiment to give a single value for each treatment for each experiment.

### Gene Ontology (GO)

Genes up-regulated in *AGO2*^*51B*^ null mutant but not in *AGO2*^*V966M*^ catalytic mutant female larva mRNA-seq libraries were analyzed for Biological Process GO-term enrichment in FlyMine http://www.flymine.org, an integrated database for *Drosophila* genomics [35]. The same gene can appear in more than 1 category. The actual source for GO analysis is the Gene Ontology Consortium http://www.geneontology.org/ [36].

### ChIP-seq

Trimming reads for adapters and light quality trimming was performed with cutadapt v1.10 using the parameters "—quality-cutoff 20 -a AGATCGGAAGAGC—minimum-length = 25—overlap = 10". Reads were aligned to the same reference genome described above for RNA-seq using bowtie2 v2.2.8 [37] with default parameters. Multimapping reads were removed using the "view" program of samtools v1.3.1 [38] with parameter "-q 20". The Picard tools v2.5.0 (http://broadinstitute.github.io/picard/) program MarkDuplicates was used to remove PCR duplicates from mapped reads.

Peak calling methods depended on whether the binding of the protein is punctate (AGO2) or broad (Pol II). In both cases we first used the MACS2 v2.1.1.20160309 callpeak program [39] and https://github.com/taoliu/MACS) to call peaks on each replicate independently. For both AGO2 and Pol II we used the non-default parameters "—gsize dm—bdg—SPMR". For Pol II we additionally included the parameter "—broad". We then ran the same peak-calling steps but on the pooled set of replicates for each target protein, by providing all input and IP replicates to MACS2. The signal bedGraph outputs from the pooled peak-calling runs were used in screenshots for figures.

For the punctate AGO2 peaks, we used the Irreproducible Discovery Rate (IDR) method using IDR v2.0.3 [40], https://github.com/nboley/idr, and following recommendations from https://sites.google.com/site/anshulkundaje/projects/idr). Initial lenient peaks were called with MACS2 with the lenient parameters "-p 1e-3—nomodel—to-large" on four versions of the IP data: replicates, pooled pseudoreplicates (BAM files generated by pooling all replicates, shuffling the result, and then splitting back out an equal number of reads into separate pseudoreplicate BAM files), self-pseudoreplicates (shuffle-and-split each replicate individually) and pooled replicates. Pooled input was always used as the control for these peak-calling runs. On each pair of replicate, self-pseudoreplicate, and pooled-pseudoreplicate called peaks, we ran IDR with parameters "—use-best-multisummit-IDR—soft-idr-threshold 0.05—rank p.value—input-file-type narrowPeak—peak-list $POOLED" where "$POOLED" is the peaks called on all replicates pooled together. From the pooled peaks ranked by increasing p-value, we chose the final AGO2 peaks as the top N peaks were N was the number of peaks in the pooled-pseudoreplicate comparison passing an IDR threshold of 0.05, as recommended by the IDR authors.

The IDR method has not been tested on broad peaks, and as such the authors do not recommend using it in such cases. We therefore selected final peaks for Pol II as those peaks in the pooled peak-calling run that were also found in at least one of the individual replicates. Specifically, using BEDTools v2.25.0 and pybedtools v0.7.9 [41,42] we used "pooled.intersect([replicates], u = True).sort().merge()" where "pooled" points to the pooled peak calls and "[replicates]" is the list of individual peak calls for each replicate.

When comparing AGO2 ChIP-seq peaks to genes changed upon AGO2 depletion, we considered a gene bound by AGO2 if it had an AGO2 peak overlapping at least one of the gene TSSs by at least 1 bp. In all other comparisons, we considered a gene bound by a protein if the protein overlapped anywhere in the gene body by at least 1 bp.

### 4C-seq

We used the 4c-ker algorithm [17] to analyze 4C-seq data following the authors' recommendations documented in https://github.com/rr1859/R.4Cker. The reduced genome was created around HindIII sites with the fragment size of 13 bp on either side. The primer sequence was ACCGTTTTTGACAACAGCAGCTGTA and was flanked by a HindIII site starting at genomic position chr2L:15318918 in the dm6 assembly. Results from the "near-bait" analysis mode (defined by 4c-ker to be 5 Mb on either side of the bait) are presented here, and used k = 5, and pval = 0.05. Post-processing of the 4c-ker results files were required to identify the direction of interaction changes (gain/loss). Specifically, the bedGraph signal output, which contains values for overlapping windows of different sizes, was intersected with each differential region for the each treatment and control replicate using BEDTools and pybedtools. The gain/loss fold change was calculated as the weighted average of treatment window signals divided by the weighted average of control window signals intersecting each differential region.

### External data

Chromatin colors from [9] were downloaded from GEO accession GSE22069 and lifted over to the dm6 assembly using liftOver from the UCSC tools [43]. TADs from [8] were downloaded from http://chorogenome.ie-freiburg.mpg.de [8] and lifted over to the dm6 assembly. LADs from [11] were downloaded from supplemental data. Original LAD data were in the dm2 assembly, so we performed a 2-stage liftover first from dm2 to dm3 and then from dm3 to dm6.

### Fisher's exact tests

All FETs used the implementation in the Python scipy package (scipy.stats.fisher_exact). When comparing intervals (peaks, TADs) with genes, we considered the entire gene body. Reported p-values are two-tailed. When multiple tests were performed (e.g., for heatmaps in Fig 3), we applied the Benjamini-Hochberg multiple test correction (FDR) as implemented in the statsmodels Python package (statsmodels.stats.multitest.fdrcorrection). Where FDR is displayed in heatmaps, if the odds ratio was <1 then the sign of the corresponding FDR was flipped such that negative values indicate depletion and positive values indicate enrichment. Where log-odds ratio is displayed in heatmaps, if the FDR was > 0.05 then the log odds was set to zero.

### Colocalization

For computing colocalization between sets of intervals, we used the Genomic Association Test v1.2.2 framework [44] using the dm6 assembly as the domain or workspace and otherwise default parameters. In the heatmaps showing log2 fold change enrichment, any comparisons where the q-value was <0.05 was reset to have a log2 fold change of zero, and any self-self comparisons were set to zero.

### Deposited data

Data are available from the following GEO accessions: GSE101365 (ChIP-seq), GSE95844 (mRNA-seq), GSE95845 (neuRNA-seq), and GSE95847 (4C-seq). The following link contains the setup, code, and documentation to reproduce the analysis and generate all figures: https://doi.org/10.6084/m9.figshare.5829318.

## Supporting information

S1 Fig Characterization of AGO2 and AGO2^V966M^ rescue constructs and neuRNA-seq analysis. A) AGO2 gene models and position of two independent siRNAs used. The short interfering RNA siAGO2 recognizes an exon common to both AGO2 transcripts whereas si5UTR recognizes the 5'UTR of both transcripts. B) Schematic diagram representing AGO2 and AGO2^V966M^ mutant. Constructs lack the AGO2 UTRs and therefore are resistant to degradation by si5UTR. N-terminal domain (NTD), PAZ domain, and PIWI domain are indicated. Asterisk indicates the position of the single PIWI domain point mutation V966M, which renders the protein catalytically inactive. C) Immunofluorescence (IF) analysis of transfected cells with the indicated constructs. IF was performed using anti-Flag antibody (green) and anti-CP190 (red) as nuclear marker. Nuclei were stained with DAPI (blue). Bar represents 14 μm. D) Western blot of transfected cells with the indicated constructs. Anti-AGO2 (Liu) recognizes both endogenous and ectopically expressed constructs. LaminB levels are also shown. E) Heatmap of up-regulated (red) and down-regulated (blue) genes corresponding to neuRNA-seq from si5UTR-depleted cells relative to mock transfected cells in addition to rescue with empty plasmid, *AGO2*, or *AGO2*^*V966M*^ constructs. Only genes changed in si5UTR-depleted cells relative to mock transfected cells are shown. White indicates restored expression of that gene (row) in rescue sample. Black arrow indicates the *AGO2* gene, which is reduced in expression in si5UTR-depleted cells but up-regulated in *AGO2* and *AGO2*^*V966M*^ rescues.
(TIF)

S2 Fig AGO2 and LaminB attenuate transcription in active RED chromatin inside and outside LADs. FET barplots testing association between the TSS of co-up-regulated (A) or co-down-regulated (B) genes in AGO2 KD and LaminB KD compared to chromatin colors, AGO2 chromatin association, inside LADs (black) or outside (grey). Association between affected genes with chromatin colors and LADs is expressed as log_2_ odds ratio. Asterisks indicate significant associations for log_2_ odds ratios >1 or <-1. (C) Violin plot showing a comparison of the expression levels in mock samples from the AGO2 KD and LaminB KD experiment across the following different classes of genes: in LAD and RED, LAD but not RED, RED but not LAD, and all other genes. Genes were sub-classified into whether or not the gene was co-upregulated in both knockdowns.
(TIF)

S3 Fig Strategy followed to analyze mRNA-seq libraries of *AGO2* mutants and validation of affected genes in cell lines. A) Strategy used to identify genes that depend on AGO2 independently of its catalytic activity. Data correspond to up-regulated genes from third instar larval strains profiled by mRNA-seq. B) Western blot showing knockdown efficiency of AGO2 in Kc167 and D8 female cell lines. LaminB levels are also shown. C) Validation by qRT-PCR for a set of spermatogenesis genes up-regulated upon depletion of AGO2 in Kc167 cells. Error bars correspond to standard deviation of four experiments. D) Validation in D8 cells. E) Validation by qRT-PCR for a set of genes located within a repressive TAD/LAD upon depletion of AGO2 or LaminB in Kc cells.
(TIF)

S4 Fig Radial position analysis for *nht* locus in either AGO2 or LaminB knockdown cells. A) Genome browser view of LADs, TADs, and AGO2 ChIP peaks (grey and black bars, respectively) depicting chromatin context in which *nht* is located in Kc cells. The probe used for DNA-FISH against *nht* is shown as a green bar. Testis-expressed genes are highlighted in green. B) Representative maximal projections of images using a probe against *nht* in mock, AGO2-, and LaminB- depleted cells. Nuclei were stained with DAPI. Scale bar represents 16 μm. C) Cumulative histograms of normalized radial distance distributions for *nht* from nuclear periphery. The horizontal axis represents the radial distance expressed as 250 concentric bins. Distance from nuclear periphery was determined using two biological replicates. FISH signals for mock (n = 1721), AGO2 KD (n = 1664), and LaminB KD (n = 2668). Reported *p*-values correspond to Kolmogorov-Smirnov test.
(TIF)

S1 Table Full list of AGO2-associated nuclear proteins identified by mass spec. (PDF)

S2 Table Full lists of affected genes in neuRNA-seq and mRNA-seq analyses. (PDF)

S3 Table Spermatogenesis genes located in the *nht* cluster are also up-regulated in *AGO2^51B^* null mutant female larvae. A) Analysis of mRNA-seq libraries from female larvae showing that several spermatogenesis genes close to *nht* are specifically up-regulated in *AGO2*^*51B*^ but not in *AGO2*^*V966M*^ catalytic mutants. Values are expressed as fold change (Log_2_) relative to expression in *w* strain (as control) and significance is provided as adjusted *p*-values (p_adj_). Note that the expression level for several genes in the control is below the sensitivity of the assay; thus, the fold change in the *AGO2*^*51B*^ mutant is reported as infinite. In addition, since several genes are also not expressed in the *AGO2*^*V966M*^ mutant, the fold change is reported as undefined in these cases. B) qRT-PCR adjusted Ct values for three spermatogenesis genes and *rp49* across the AGO2 female larvae mutants analyzed. Ct values were converted to arbitrary values relative to genomic DNA standard curves generated for each gene to account for differences in primer efficiencies.
(PDF)

S4 Table Antibodies used in this study. (PDF)

S5 Table Oligos used in this study. (PDF)
