# Supplementary figures and images for: Argonaute2 and LaminB modulate gene expression by controlling chromatin topology
Source: PLoS Genet. 2018 Mar 12;14(3):e1007276. doi: 10.1371/journal.pgen.1007276 (PMC5864089; doi:10.1371/journal.pgen.1007276)

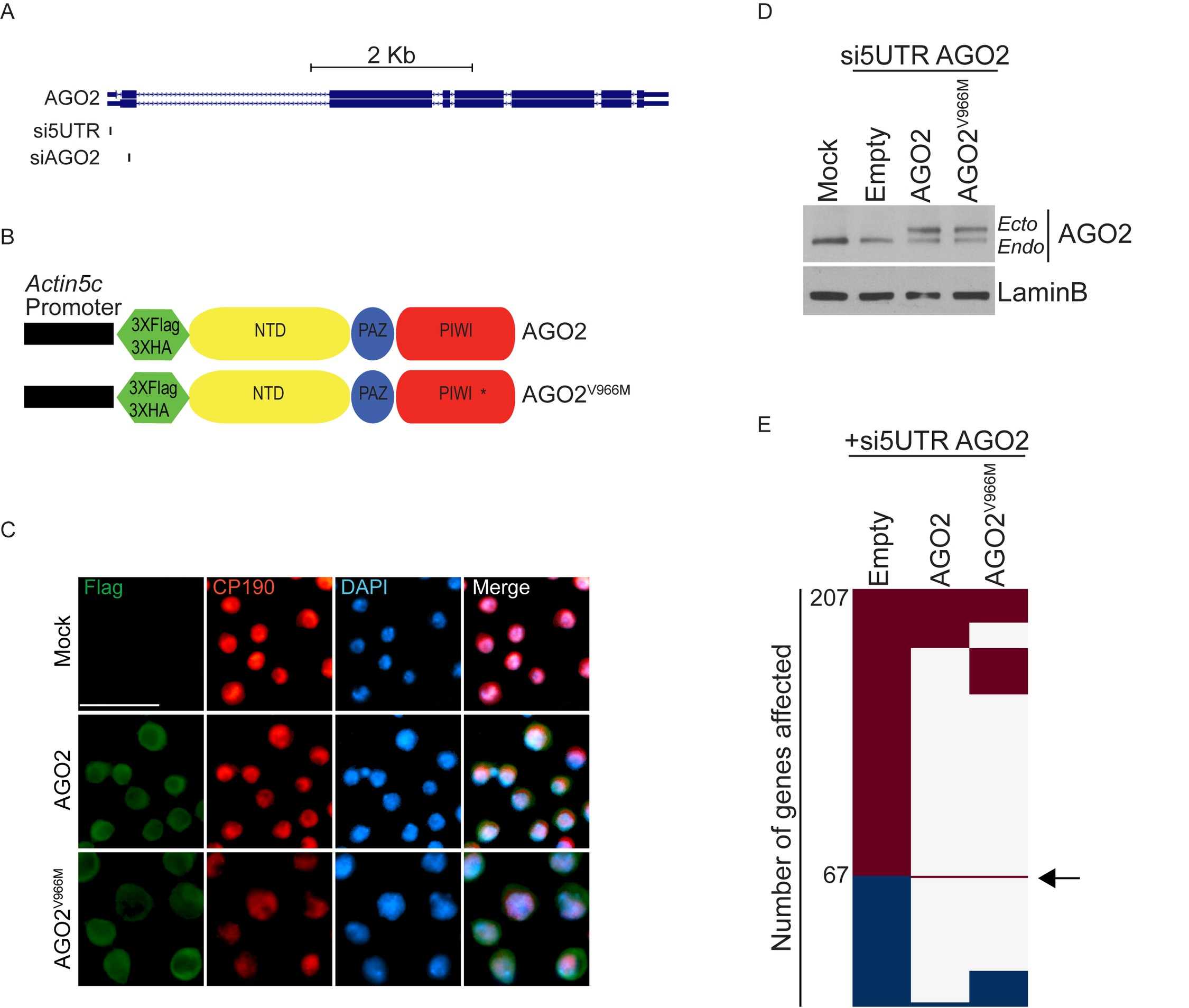

Supplement: S1 Fig — A) AGO2 gene models and position of two independent siRNAs used. The short interfering RNA siAGO2 recognizes an exon common to both AGO2 transcripts whereas si5UTR recognizes the 5'UTR of both transcripts. B) Schematic diagram representing AGO2 and AGO2V966M mutant. Constructs lack the AGO2 UTRs and therefore are resistant to degradation by si5UTR. N-terminal domain (NTD), PAZ domain, and PIWI domain are indicated. Asterisk indicates the position of the single PIWI domain point mutation V966M, which renders the protein catalytically inactive. C) Immunofluorescence (IF) analysis of transfected cells with the indicated constructs. IF was performed using anti-Flag antibody (green) and anti-CP190 (red) as nuclear marker. Nuclei were stained with DAPI (blue). Bar represents 14 μm. D) Western blot of transfected cells with the indicated constructs. Anti-AGO2 (Liu) recognizes both endogenous and ectopically expressed constructs. LaminB levels are also shown. E) Heatmap of up-regulated (red) and down-regulated (blue) genes corresponding to neuRNA-seq from si5UTR-depleted cells relative to mock transfected cells in addition to rescue with empty plasmid, AGO2, or AGO2V966M constructs. Only genes changed in si5UTR-depleted cells relative to mock transfected cells are shown. White indicates restored expression of that gene (row) in rescue sample. Black arrow indicates the AGO2 gene, which is reduced in expression in si5UTR-depleted cells but up-regulated in AGO2 and AGO2V966M rescues. (TIF) [file pgen.1007276.s001.tif]

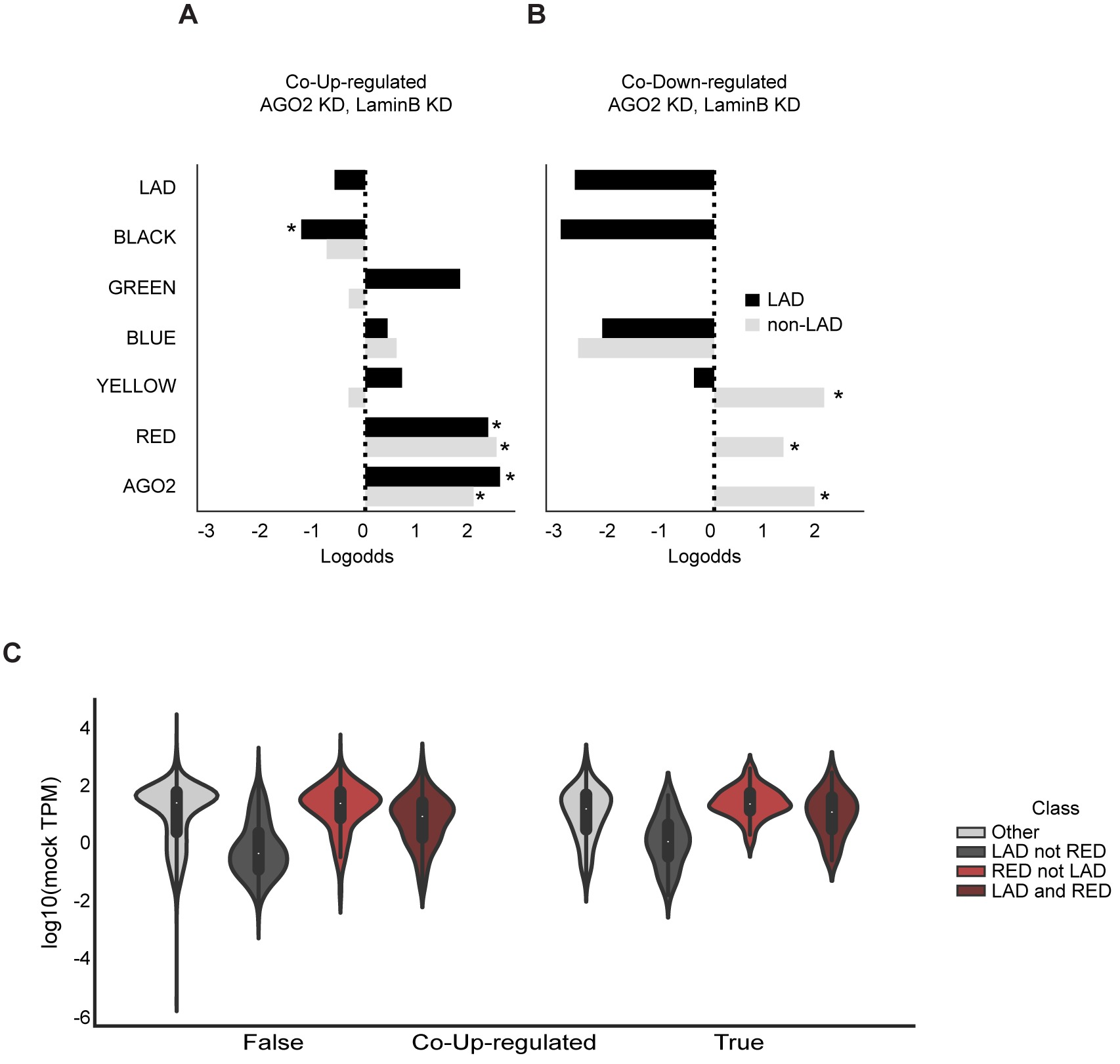

Supplement: S2 Fig — FET barplots testing association between the TSS of co-up-regulated (A) or co-down-regulated (B) genes in AGO2 KD and LaminB KD compared to chromatin colors, AGO2 chromatin association, inside LADs (black) or outside (grey). Association between affected genes with chromatin colors and LADs is expressed as log2 odds ratio. Asterisks indicate significant associations for log2 odds ratios >1 or <-1. (C) Violin plot showing a comparison of the expression levels in mock samples from the AGO2 KD and LaminB KD experiment across the following different classes of genes: in LAD and RED, LAD but not RED, RED but not LAD, and all other genes. Genes were sub-classified into whether or not the gene was co-upregulated in both knockdowns. (TIF) [file pgen.1007276.s002.tif]

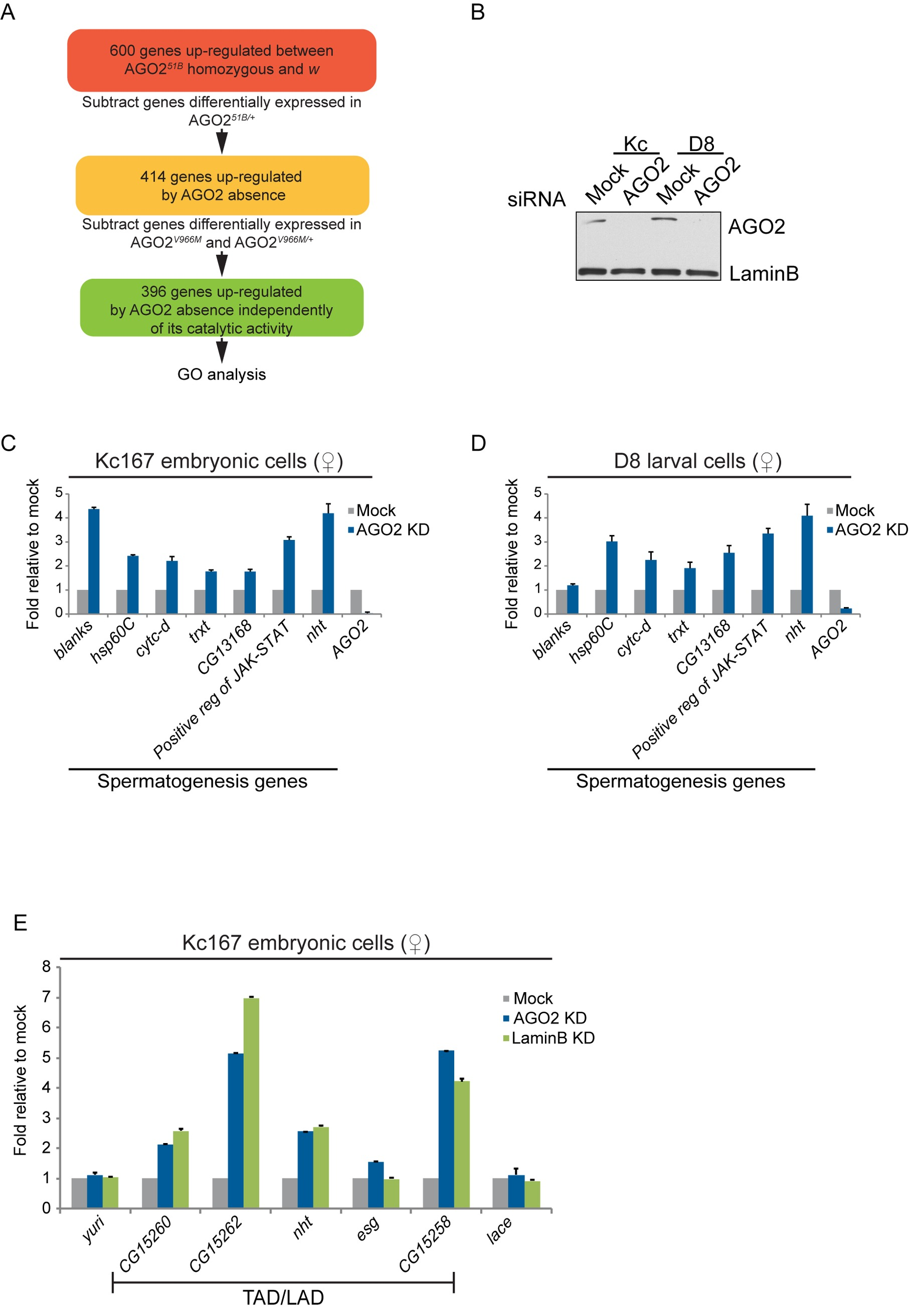

Supplement: S3 Fig — A) Strategy used to identify genes that depend on AGO2 independently of its catalytic activity. Data correspond to up-regulated genes from third instar larval strains profiled by mRNA-seq. B) Western blot showing knockdown efficiency of AGO2 in Kc167 and D8 female cell lines. LaminB levels are also shown. C) Validation by qRT-PCR for a set of spermatogenesis genes up-regulated upon depletion of AGO2 in Kc167 cells. Error bars correspond to standard deviation of four experiments. D) Validation in D8 cells. E) Validation by qRT-PCR for a set of genes located within a repressive TAD/LAD upon depletion of AGO2 or LaminB in Kc cells. (TIF) [file pgen.1007276.s003.tif]

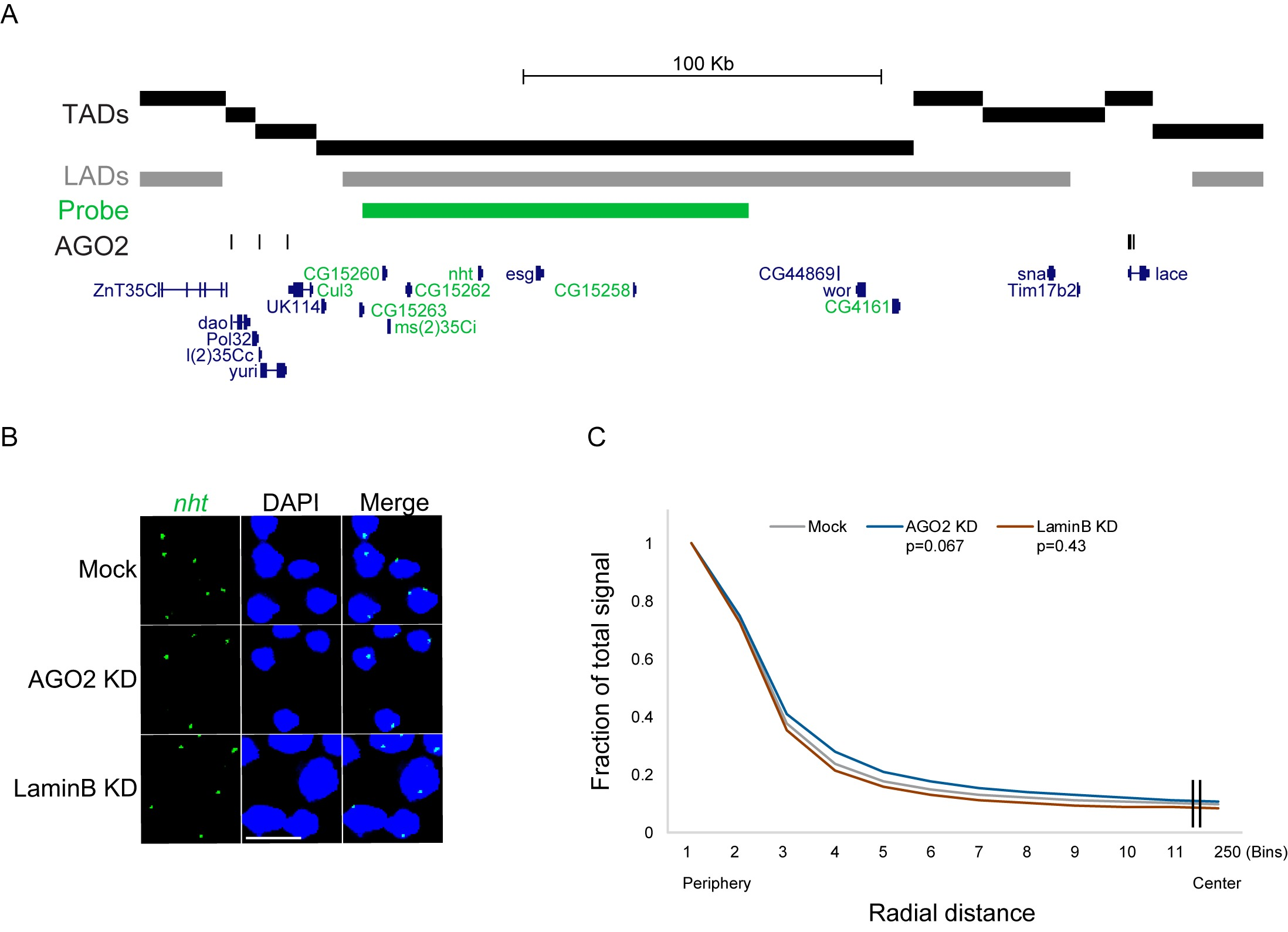

Supplement: S4 Fig — A) Genome browser view of LADs, TADs, and AGO2 ChIP peaks (grey and black bars, respectively) depicting chromatin context in which nht is located in Kc cells. The probe used for DNA-FISH against nht is shown as a green bar. Testis-expressed genes are highlighted in green. B) Representative maximal projections of images using a probe against nht in mock, AGO2-, and LaminB- depleted cells. Nuclei were stained with DAPI. Scale bar represents 16 μm. C) Cumulative histograms of normalized radial distance distributions for nht from nuclear periphery. The horizontal axis represents the radial distance expressed as 250 concentric bins. Distance from nuclear periphery was determined using two biological replicates. FISH signals for mock (n = 1721), AGO2 KD (n = 1664), and LaminB KD (n = 2668). Reported p-values correspond to Kolmogorov-Smirnov test. (TIF) [file pgen.1007276.s004.tif]
