# Supplementary material for: Argonaute2 and LaminB modulate gene expression by controlling chromatin topology
Source: PLoS Genet. 2018 Mar 12;14(3):e1007276. doi: 10.1371/journal.pgen.1007276 (PMC5864089; doi:10.1371/journal.pgen.1007276)
Supplement: S1 Table — (PDF) [file pgen.1007276.s005.pdf]

Proteins identified by mass spec	Score
AGO2	2399
Host cell factor	1669
ATP-dependent RNA helicase bel	1548
Histone-lysine N-methyltransferase trr	1477
Nucleosome-remodeling factor subunit NURF301	1361
Polyadenylate-binding protein	1356
Mediator of RNA polymerase II transcription subunit 13	1127
Pyruvate kinase	1072
Dynein light chain 1	986
Helicase domino	982
Ataxin-2 homolog	979
Mediator of RNA polymerase II transcription subunit 12	908
DNA topoisomerase 2	885
T-complex protein 1 subunit alpha	844
Putative oxidoreductase GLYR1 homolog	839
T-complex protein 1 subunit gamma	744
Histone deacetylase Mi2	712
Replication factor C subunit 1	674
Grainyhead	674
Lingerer	673
Neurogenic protein mastermind	649
E3 ubiquitin-protein ligase hyd	643
RNA polymerase II subunit RPB1	605
LaminB	604
Importin subunit alpha	575
Fragile X mental retardation syndrome-related protein 1	563
Chromatin-remodeling complex ATPase chain Iswi	561
Lysine-specific demethylase lid	551
ATP-dependent helicase brm	542
Transcription initiation factor TFIID subunit 4	539
Transcription elongation factor SPT6	473
Lamin-B receptor	468
DNA polymerase delta catalytic subunit	454
Otefin	450
ADP,ATP carrier protein	447
Heterogeneous nuclear ribonucleoprotein 27C	444
RNA-binding protein squid	439
DNA replication licensing factor Mcm7	431
Nipped-B protein	427
Transcription factor GAGA	400
F-box-like/WD repeat-containing protein ebi	398
Longitudinals lacking protein, isoform G	388
Serine/threonine-protein phosphatase PP2A 65 kDa regulatory subunit	387
Poly [ADP-ribose] polymerase	379
DNA ligase 1	377
HEAT repeat-containing protein 1 homolog	376

60S ribosomal protein L3	363
RuvB-like helicase 1	350
Modifier of mdg4	347
Protein BCL9 homolog	342
Transcription initiation factor TFIID subunit 1	340
Putative U5 small nuclear ribonucleoprotein 200 kDa helicase	339
Heat shock protein 83	337
Heterochromatin protein 1	336
Zinc finger protein on ecdysone puffs	334
rRNA 2'-O-methyltransferase fibrillarin	329
Chromosomal serine/threonine-protein kinase JIL-1	326
Tubulin alpha-4 chain	325
60S ribosomal protein L10a-2	323
Heterogeneous nuclear ribonucleoprotein 87F	323
Heterogeneous nuclear ribonucleoprotein A1	310
60S ribosomal protein L23	308
60S ribosomal protein L18	307
Calcium-transporting ATPase sarcoplasmic/endoplasmic reticulum type	304
60S ribosomal protein L7a	300
RNA-binding protein fusilli	278
Nuclear pore complex protein Nup160 homolog	274
40S ribosomal protein S2	258
Transitional endoplasmic reticulum ATPase TER94	254
DNA repair protein RAD50	249
40S ribosomal protein S3a	246
Histone H3	246
Myosin heavy chain, muscle	242
DNA replication licensing factor MCM4	237
Protein wech	237
Transcription elongation factor SPT5	236
Polycomb protein Sfmbt	236
DNA mismatch repair protein spellchecker 1	232
Integrator complex subunit 3 homolog	232
60S ribosomal protein L8	231
Profilin	228
60S ribosomal protein L14	224
FACT complex subunit spt16	224
Elongation factor 2	224
Polyhomeotic-proximal chromatin protein	221
Cyclin-dependent kinase 1	220
Kinesin-like protein subito	219
Males-absent on the first protein	218
40S ribosomal protein S3	217
Protein ELYS homolog	215
60S ribosomal protein L22	213
Serine/threonine-protein kinase polo	211
Translationally-controlled tumor protein homolog	210

Probable histone-binding protein Caf1	210
Protein suppressor of variegation 3-7	206
Ribonuclease H2 subunit A	206
Origin recognition complex subunit 1	205
Box A-binding factor	204
Ribosomal RNA processing protein 1 homolog	204
High mobility group protein DSP1	203
Transcription initiation factor TFIID subunit 6	202
Mediator of RNA polymerase II transcription subunit 14	201
40S ribosomal protein S4	200
Zinc finger protein CG2199	200
40S ribosomal protein S6	199
Histone-lysine N-methyltransferase, H3 lysine-79 specific	199
Transcription-associated protein 1	196
Ran GTPase-activating protein	195
Peptidyl-prolyl cis-trans isomerase	194
Dynamin	190
Mediator of RNA polymerase II transcription subunit 26	189
Vitellogenin-2	188
Guanylate kinase-associated protein mars	187
Heat shock factor protein	185
Polycomb protein PHO	180
Ubiquitin-40S ribosomal protein S27a	177
Inorganic pyrophosphatase	177
Transcription factor Dp	176
60S ribosomal protein L18a	175
Vitellogenin-3	173
DNA-directed RNA polymerase II subunit RPB2	170
Enhancer of mRNA-decapping protein 4 homolog	168
Histone deacetylase Rpd3	165
Ankyrin repeat and KH domain-containing protein mask	165
Cathepsin L	163
Protein tramtrack, beta isoform	162
Protein tramtrack, alpha isoform	159
14-3-3 protein epsilon	155
60S ribosomal protein L13a	154
Guanine nucleotide-binding protein subunit beta-like protein	154
Ubiquitin carboxyl-terminal hydrolase 36	151
Proteasome subunit alpha type-5	151
40S ribosomal protein S23	150
Polycomb protein l(1)G0020	146
40S ribosomal protein SA	146
LIM and SH3 domain protein Lasp	145
Lamin-C	145
40S ribosomal protein S17	145
Probable ATP-dependent RNA helicase kurz	144
Mediator of RNA polymerase II transcription subunit 17	143

60S ribosomal protein L7	142
Protein strawberry notch	141
Uncharacterized protein CG7065	140
Mediator of RNA polymerase II transcription subunit 25	139
Nucleolar complex protein 3 homolog	139
40S ribosomal protein S18	137
Actin-5C	137
Probable ATP-dependent RNA helicase CG8611	137
Supporter of activation of yellow protein	136
Protein suppressor of hairy wing	136
RNA-binding protein pno1	136
Probable ribosome biogenesis protein RLP24	135
Putative peptidyl-prolyl cis-trans isomerase dodo	135
Transcription termination factor 2	133
C-terminal-binding protein	133
Regulator of nonsense transcripts 1 homolog	131
THO complex subunit 5	131
Protein phosphatase PP2A 55 kDa regulatory subunit	131
Eukaryotic initiation factor 4A	131
Serine/threonine-protein phosphatase Pgam5, mitochondrial	130
Replication protein A 70 kDa DNA-binding subunit	130
40S ribosomal protein S7	129
60S ribosomal protein L19	128
Protein slender lobes	128
60S ribosomal protein L5	126
Vitellogenin-1	123
GATA-binding factor A	123
Histone H1	123
RRP12-like protein	121
Nucleoplasmin-like protein	120
Protein daughterless	120
ATP-dependent RNA helicase abstrakt	120
Importin subunit beta	119
Polycomb group protein Psc	119
Histone-lysine N-methyltransferase eggless	117
Serine/threonine-protein phosphatase alpha-2 isoform	117
GATA-binding factor C	117
Transcriptional regulator ATRX homolog	116
RNA-binding protein lark	116
Signal transducer and transcription activator	116
Apoptosis inhibitor 5 homolog	116
Enhancer of mRNA-decapping protein 3	115
40S ribosomal protein S11	115
5'-3' exoribonuclease 2 homolog	114
Pescadillo homolog	114
Pescadillo homolog	114
Hormone receptor 4	114

60S ribosomal protein L32	114
Bloom syndrome protein homolog	114
Protein NASP homolog	113
Homeotic protein ultrabithorax	113
Kinesin-like protein Klp10A	113
Protein suppressor of underreplication	113
Protein no-on-transient A	112
Mini-chromosome maintenance complex-binding protein	111
Protein encore	111
Puff-specific protein Bx42	109
Protein LST8 homolog	108
60S ribosomal protein L15	108
Probable histone-lysine N-methyltransferase Mes-4	107
Protein dead ringer	106
Probable nucleolar GTP-binding protein 1	105
Probable small nuclear ribonucleoprotein Sm D1	105
Protein claret segregational	103
RuvB-like helicase 2	103
Cell division control protein 2 cognate	103
Probable small nuclear ribonucleoprotein G	102
Probable nucleoporin Nup54	102
Protein lin-54 homolog	101
Transcription initiation factor TFIID subunit 5	101
Casein kinase I isoform alpha	101
U3 small nucleolar RNA-associated protein 18 homolog	101
Lethal(2) giant larvae protein	99
Protein lethal(2)denticleless	99
Histone-lysine N-methyltransferase trithorax	99
Nucleosomal histone kinase 1	99
DNA replication licensing factor Mcm2	98
Probable ATP-dependent RNA helicase pitchoune	98
60S ribosomal protein L37a	98
Histone-lysine N-methyltransferase E(z)	97
Protein pangolin, isoforms A/H/I	96
40S ribosomal protein S16	96
Protein anoxia up-regulated	95
Protein KRI1 homolog	94
60S ribosomal protein L38	94
26S protease regulatory subunit 4	93
Pre-mRNA-splicing factor Slu7	92
Probable protein phosphatase CG10417	92
Heat shock protein 23	92
SelT-like protein	91
Longitudinals lacking protein-like	91
Mediator of RNA polymerase II transcription subunit 23	90
Mediator of RNA polymerase II transcription subunit 1	90
IGHM protein	90

PERQ amino acid-rich with GYF domain-containing protein CG11148	90
DNA replication licensing factor Mcm5	90
Histone-lysine N-methyltransferase ash1	89
Peroxiredoxin 1	89
Pair-rule protein odd-paired	89
ATP synthase subunit gamma, mitochondrial	87
Protein groucho	87
Transcription initiation factor TFIID subunit 2	86
Homeotic protein ocelliless	86
Nucleoporin Ndc1	86
GTP-binding nuclear protein Ran	84
DNA replication licensing factor Mcm6	84
Polynucleotide 5'-hydroxyl-kinase NOL9	83
Zinc finger matrin-type protein CG9776	82
Nuclear hormone receptor FTZ-F1	82
JmjC domain-containing histone demethylation protein 1	81
DNA polymerase alpha catalytic subunit	81
Homeobox protein abdominal-A	80
Replication factor C subunit 2	80
Ribosome production factor 2 homolog	79
Histone H3.3	78
Transcriptional activator cubitus interruptus	78
Protein rigor mortis	78
60S ribosomal protein L17	78
Protein held out wings	78
Putative DNA helicase Ino80	78
Uncharacterized protein CG4951	77
Chorion peroxidase	77
Large subunit GTPase 1 homolog	77
Clathrin heavy chain	76
60S ribosomal protein L31	76
40S ribosomal protein S26	76
Protein will die slowly	75
60S ribosomal protein L10	75
La-related protein	75
Protein bric-a-brac 2	75
40S ribosomal protein S9	75
Putative cysteine proteinase CG12163	74
Protein suppressor of sable	74
Putative ATP-dependent RNA helicase me31b	73
Putative hydroxypyruvate isomerase	72
Actin-binding protein anillin	72
Ran-binding protein 16	71
Serine/threonine-protein kinase PITSLRE	71
Caspase-1	71
Zinc finger protein hangover	71
WD repeat-containing protein 55 homolog	71

DNA-binding protein modulo	71
N-acetyltransferase eco	71
Exportin-1	70
Probable dimethyladenosine transferase	70
Cyclin-dependent kinase 8	70
Lysine-specific demethylase NO66	69
Protein teflon	69
Uncharacterized protein CG9548	68
RAC serine/threonine-protein kinase	68
Protein fork head	67
Protein HIRA homolog	67
Negative elongation factor A	67
Ets DNA-binding protein pokkuri	67
40S ribosomal protein S13	66
Polycomb protein Asx	66
Protein SDA1 homolog	66
Protein jagunal	66
FACT complex subunit Ssrp1	65
Coatomer subunit beta	65
Transcription initiation factor TFIID subunit 9	65
Mediator of RNA polymerase II transcription subunit 16	64
Ribosomal L1 domain-containing protein CG13096	64
SAGA-associated factor 11 homolog	64
DNA damage-binding protein 1	64
ATP-dependent RNA helicase WM6	63
Interleukin enhancer-binding factor 2 homolog	63
Tubulin gamma-2 chain	62
Eukaryotic translation initiation factor 3 subunit H	61
Eukaryotic translation initiation factor 3 subunit L	61
Myc protein	61
UPF0474 protein CG13624	61
Surfeit locus protein 4 homolog	60
Protein Kr-h2	60
Chromodomain-helicase-DNA-binding protein 1	60
DNA-directed RNA polymerase I subunit RPA2	60
Polycomb group protein Pc	60
Probable medium-chain specific acyl-CoA dehydrogenase, mitochondrial	59
Borealin	58
MPN domain-containing protein CG4751	58
SOSS complex subunit B homolog	58
Mediator of RNA polymerase II transcription subunit 24	58
Acidic leucine-rich nuclear phosphoprotein 32 family member A	58
Brain tumor protein	58
Mitogen-activated protein kinase 14B	57
Fidgetin-like protein 1	57
Eukaryotic translation initiation factor 3 subunit K	57
NHP2-like protein 1 homolog	57

DNA primase large subunit	57
Enolase	56
Protein MCM10 homolog	56
60S ribosomal protein L11	56
Triosephosphate isomerase	56
GTP-binding protein CG1354	56
RING finger protein unkempt	55
Protein ariadne-1	55
Elongation factor 1-gamma	55
Protein cup	55
40S ribosomal protein S12	54
Serendipity locus protein H-1	54
E3 ubiquitin-protein ligase UBR1	54
REST corepressor	54
40S ribosomal protein S15Aa	53
Homeobox protein abdominal-B	53
Zinc finger CCCH-type with G patch domain-containing protein	53
Signal recognition particle receptor subunit alpha homolog	53
Claspin homolog	53
26S proteasome non-ATPase regulatory subunit 4	53
Titin	53
40S ribosomal protein S10b	53
DNA primase small subunit	52
Protein shuttle craft	52
Calcium/calmodulin-dependent protein kinase type II alpha chain	52
Dolichyl-diphosphooligosaccharide--protein glycosyltransferase 48 kDa subunit	52
ATR-interacting protein mus304	51
Serine/threonine-protein phosphatase PP2A	51
Slo-interacting protein 1	51
Protein crooked neck	50
Centrosome-associated zinc finger protein CP190	50
Coatomer subunit gamma	49
Transcription initiation factor TFIID subunit 12	49
Eukaryotic translation initiation factor 2 subunit 1	49
Probable N6-adenosine-methyltransferase MT-A70-like protein	49
Zinc finger protein ush	49
E3 ubiquitin-protein ligase Bre1	48
Origin recognition complex subunit 5	48
Mediator of RNA polymerase II transcription subunit 19	48
40S ribosomal protein S25	48
60S ribosomal protein L13	48
Origin recognition complex subunit 2	48
Nuclear cap-binding protein subunit 1	47
Amidophosphoribosyltransferase	47
MAU2 chromatid cohesion factor homolog	47
Putative elongator complex protein 4	46
V-type proton ATPase subunit B	46

Myb protein	46
Paramyosin, long form	46
ATP synthase subunit beta, mitochondrial	46
CAD protein	46
Trithorax group protein osa	46
Discs overgrown protein kinase	45
Yemanuclein-alpha	45
H/ACA ribonucleoprotein complex subunit 3	44
NEDD8-conjugating enzyme Ubc12	44
F-box/WD repeat-containing protein 7	44
Splicing factor U2af 38 kDa subunit	44
60S ribosomal protein L27a	43
Retinoblastoma family protein	43
Gastrulation defective protein 1 homolog	43
Signal peptidase complex subunit 2	43
Zinc finger protein 2	43
60S ribosomal protein L36	43
Protein abrupt	42
Negative elongation factor B	42
Eukaryotic translation initiation factor 2 subunit 3	42
Transmembrane protein 120 homolog	41
Zinc finger protein jing	40
Probable H/ACA ribonucleoprotein complex subunit 1	40
V-type proton ATPase subunit D 1	40
Protein aurora borealis	40
Protein maelstrom 2	40
Developmental protein eyes absent	40
Mediator of RNA polymerase II transcription subunit 15	39
40S ribosomal protein S29	39
DEAD-box helicase Dbp80	39
Cullin homolog 1	39
40S ribosomal protein S19a	39
Vacuolar protein sorting-associated protein 72 homolog	38
Protein twist	38
COP9 signalosome complex subunit 7	37
Microtubule-associated protein Jupiter	37
Eukaryotic translation initiation factor 3 subunit D-1	37
Tyrosine-protein kinase Btk29A	37
Splicing factor U2AF 50 kDa subunit	37
DNA repair protein complementing XP-C cells homolog	37
Probable isocitrate dehydrogenase [NAD] subunit alpha, mitochondrial	36
Nuclear pore complex protein Nup214	35
RNA polymerase-associated protein Rtf1	35
Transcription initiation factor TFIID subunit 10b	35
Exportin-2	35
NuA4 complex subunit EAF3 homolog	35
Actin-related protein 5	35

Asparagine synthetase domain-containing protein CG17486	35
Serendipity locus protein delta	34
Aryl hydrocarbon receptor nuclear translocator homolog	34
PHD finger protein rhinoceros	34
E3 ubiquitin-protein ligase RING1	33
Dimethyladenosine transferase 1, mitochondrial	33
Dolichyl-diphosphooligosaccharide--protein glycosyltransferase subunit DAD1	33
Guanine nucleotide-binding protein-like 3 homolog	33
Maternal protein pumilio	32
ATPase ASNA1 homolog	32
Protein hu-li tai shao	32
Eukaryotic translation initiation factor 3 subunit M	32
Ecdysone receptor	31
Protein charlatan	31
Regulator of telomere elongation helicase 1 homolog	31
Mediator of RNA polymerase II transcription subunit 11	31
Cytochrome c oxidase subunit 2	31
Probable polyprenol reductase	30
Myeloid leukemia factor	30
PHD finger and CXXC domain-containing protein CG17446	30
Congested-like trachea protein	29
Serine/threonine-protein kinase CG17528	29
NEDD8	28
ETS-like protein pointed, isoform P2/D	28
Band 7 protein CG42540	28
40S ribosomal protein S20	28
Probable aminoacyl tRNA synthase complex-interacting multifunctional protein 2	28
DNA-binding protein Ets97D	28
Probable 28S ribosomal protein S26, mitochondrial	27
Ovarian-specific serine/threonine-protein kinase Lok	27
Septin-1	27
Possible lysine-specific histone demethylase 1	27
Zinc finger CCHC domain-containing protein 8 homolog	27
Ribonucleoprotein RB97D	27
Probable histone-lysine N-methyltransferase CG1716	27
Probable N-acetyltransferase san	26
DNA repair protein complementing XP-A cells homolog	26
Ubiquitin carboxyl-terminal hydrolase 7	26
Bipolar kinesin KRP-130	26
Paired box protein Pax-6	26
Homeotic protein female sterile	25
Tetratricopeptide repeat protein 14 homolog	25
Succinate dehydrogenase [ubiquinone] flavoprotein subunit, mitochondrial	25
Cytosolic Fe-S cluster assembly factor NUBP1 homolog	25
Polycomb protein Su(z)12	24
cGMP-dependent protein kinase, isozyme 2 forms cD4/T1/T3A/T3B	24
Protein teflon	24

Histone deacetylase complex subunit SAP30 homolog	23
E3 ubiquitin-protein ligase Topors	22
Protein germ cell-less	22
DNA-binding protein Ewg	22
DDB1- and CUL4-associated factor-like 1	20
Ribosomal RNA small subunit methyltransferase NEP1	19
Moesin/ezrin/radixin homolog 2	18
