# Supplementary material for: Argonaute2 and LaminB modulate gene expression by controlling chromatin topology
Source: PLoS Genet. 2018 Mar 12;14(3):e1007276. doi: 10.1371/journal.pgen.1007276 (PMC5864089; doi:10.1371/journal.pgen.1007276)
Supplement: S3 Table — A) Analysis of mRNA-seq libraries from female larvae showing that several spermatogenesis genes close to nht are specifically up-regulated in AGO251B but not in AGO2V966M catalytic mutants. Values are expressed as fold change (Log2) relative to expression in w strain (as control) and significance is provided as adjusted p-values (padj). Note that the expression level for several genes in the control is below the sensitivity of the assay; thus, the fold change in the AGO251B mutant is reported as infinite. In addition, since several genes are also not expressed in the AGO2V966M mutant, the fold change is reported as undefined in these cases. B) qRT-PCR adjusted Ct values for three spermatogenesis genes and rp49 across the AGO2 female larvae mutants analyzed. Ct values were converted to arbitrary values relative to genomic DNA standard curves generated for each gene to account for differences in primer efficiencies. (PDF) [file pgen.1007276.s007.pdf]

Table S3 (related to figure 4). Spermatogenesis genes located in the *nht* cluster are also up-regulated in $AGO2^{51B}$ null mutant female larvae

A

$AGO2^{51B}$ relative to *w*

Gene	Fold Change (Log2)	p_{adj}
nht	Infinite	0.011
CG4161	Infinite	0.030
CG15260	Infinite	0.038
CG15262	Infinite	0.014
ms(2)35Ci	Infinite	0.17
CG15258	3.6	0.40

$AGO2^{V966M}$ relative to *w*

Gene	Fold Change (Log2)	p_{adj}
nht	Infinite	1.0
CG4161	Undefined	N/A
CG15260	Undefined	N/A
CG15262	Undefined	N/A
ms(2)35Ci	Undefined	N/A
CG15258	1.5	1.0

B

qRT-PCR Ct adjusted values

	nht	*hsp60C*	*trxt*	*rp49*
$AGO2^{51B}$	0.070	0.116	0.091	0.314
$AGO2^{51B/+}$	0.003	0.007	0.003	0.170
$AGO2^{V966M}$	0.004	0.019	0.005	0.183
$AGO2^{V966M/+}$	0.003	0.016	0.007	0.228
Rescue	0.006	0.013	0.003	0.248
w	0.004	0.020	0.005	0.320
