# Supplementary material for: Argonaute2 and LaminB modulate gene expression by controlling chromatin topology
Source: PLoS Genet. 2018 Mar 12;14(3):e1007276. doi: 10.1371/journal.pgen.1007276 (PMC5864089; doi:10.1371/journal.pgen.1007276)
Supplement: S4 Table — (PDF) [file pgen.1007276.s008.pdf]

Table S4 (related to Materials and Methods). Antibodies Used in this Study

Name	Aplication	Lab/company	Catalog
AGO2 9D6	Mass spec	Siomi	N/A
AGO2 Mueller	ChIP-seq	Mueller	N/A
AGO2 Liu	Wb; IFI	Liu	N/A
RNA Pol II 8WG16	Wb	Biolegend	920102
Tubulin	Wb; IFI	Sigma	T6074
LaminB	IP; Wb; IFI	DHSB	ADL67.10
Dcr-2	Wb	Abcam	ab4732
Flag	IFI	Sigma	F3165
Cp190	IFI	Corces	HL6040

Wb: western blot
IFI: immunofluorescence
IP: immunoprecipitation
