# Supplementary material for: Argonaute2 and LaminB modulate gene expression by controlling chromatin topology
Source: PLoS Genet. 2018 Mar 12;14(3):e1007276. doi: 10.1371/journal.pgen.1007276 (PMC5864089; doi:10.1371/journal.pgen.1007276)
Supplement: S5 Table — (PDF) [file pgen.1007276.s009.pdf]

Table S5 (related to Materials and Methods). Oligos used in this study

Primer	Gene	Application	Sequence
Blancks FOR		qRT-PCR	ACCAATGGCCGTAAGAAGCA
Blancks REV	CG10630	qRT-PCR	GTCGACTGTCACGCCCTTTA
Hsp60c FOR		qRT-PCR	CCTAAAGGTGGGCCTCCAAG
Hsp60c REV	CG7235	qRT-PCR	TCGAGCCGTACCATATTGGC
CytC-d FOR		qRT-PCR	AGTGCCACACCTACGAAGTG
CytC-d REV	CG13263	qRT-PCR	CCCGCTCCTCAGCCTTTTTA
Thrt FOR		qRT-PCR	CAAGGTGAACGTGGACGAGA
Thrt REV	CG3315	qRT-PCR	TGGGCGGACACCTCATTAAC
AGO2 FOR		qRT-PCR	GTGGTTTACACGCCTCCTCA
AGO2 REV	CG7439	qRT-PCR	GGGTAGTTGCGACTGTGGAA
RP49 FOR		qRT-PCR	ATGACCATCCGCCCAGCATAC
RP49 REV	CG7939	qRT-PCR	CTGCATGAGCAGGACCTCCAG
Nht FOR		qRT-PCR	ACGATCTCGAGATTGCCGAC
Nht REV	CG15259	qRT-PCR	TTCCAGACGAGCCACCTTTC
CG13168 FOR		qRT-PCR	ATCTGGCTGCCAAGAAGGTC
CG13168 REV	CG13168	qRT-PCR	TTTTTGAATCTTGCCCGCCG
Positive ref of JAK-STAT FOR		qRT-PCR	AAATTTGCCGTGTGGTTCCG
Positive ref of JAK-STAT REV	CG15306	qRT-PCR	TTAATCGTCGCCTGGCAAGT
CG15260 FOR		qRT-PCR	GCTCGGACCTGAGACTCATC
CG15260 REV	CG15260	qRT-PCR	CGCTGGTTGAGAAACAGTGA
CG15262 FOR		qRT-PCR	CGGATGGATATCCCATTTTG
CG15262 REV	CG15262	qRT-PCR	GGCTCGATGTCGTTGGTAAT
Wor FOR		qRT-PCR	ACAGCATCTGCCTCTTCGAT
Wor REV	CG4158	qRT-PCR	TTCATGTTTTCGCTCAGCAC
Esg FOR		qRT-PCR	TCAGCTGCAAGGATTGTGAC
Esg REV	CG3758	qRT-PCR	GAAGGTCTTCGAGCAACTGG
Yuri FOR		qRT-PCR	AATCGACGCTAAAAGCCTCA
Yuri REV	CG31732	qRT-PCR	GAAATCCACCCACTCCTGAA
CG15258 FOR		qRT-PCR	GGAAGTCACCGAAGCAGAAG
CG15258 REV	CG15258	qRT-PCR	GGGAGTTACACCGTTGGAGA
Lace FOR		qRT-PCR	CTGGGACCCAACAGTCTTGT
Lace REV	CG4162	qRT-PCR	GCCCTCGACCAAGATCATAA
Nht FOR		3C	ATGAGCAGCTGACCAGCATA
Nht REV	CG15259	3C	GCTGAAAGGCAGTAACCCTAAA
Esg FOR		3C	CCACGTGATATCCCTCCACT
Esg REV	CG3758	3C	AGGCTCCTCGGCTTGTTTAT
Wor FOR		3C	CGCTCTTGTTCCAACTGTCA
Wor REV	CG4158	3C	CACACTCTCCGATCAGCAAA
Lace FOR		3C	GGCTACGACAATTTGGCAAT
Lace REV	CG4162	3C	GCATCCCAACTGTGGGTAAA
CG15262 FOR		3C	TCCCTGCTTAACGAGTTGCT
CG15262 REV	CG15262	3C	GCAGAGATCGCTTTGGTTTG
CG15260 FOR		3C	GCCCTAACCGCAGATAGATG
CG15260 REV	CG15260	3C	CAAGAATGTGCGTCGTCAGT
Yuri FOR		3C	GTTCTCCTCGCCTTCAACTG
Yuri REV	CG31732	3C	CGCCCATTTTTGTGTTGTTT
ZnT35c FOR		3C	AGCTCGGATCGAAACAAAAA
ZnT35c REV	CG3994	3C	CGATCCGATACGGTGTAAGC
Probe nht promoter	CG15259	3C TaqMan probe	TTTCCCTTGAAGTTCCTATTCAGCCG

AGO2 B P-entry FOR		Cloning in P-entry TOPO	CACCATGGGAAAAAAAGATAAGAACAAGAA
AGO2 B P-entry REV	CG7439	Cloning in P-entry TOPO	TCAGACAAAGTACATGGGGTTTT
AGO2 V966M g2896a FOR		for making AGO2^{V966M} mutant	GAGAGGGATGGGTCATATCGGCTCCAATGTACA
AGO2 V966M g2896a REV	CG7439	for making AGO2^{V966M} mutant	TGTACATTGGAGCCGATATGACCCATCCCTCTC
siAGO2		siRNA knockdowns	CAACCACAGCAGCUGCAACdTdT
siAGO2	CG7439	siRNA knockdowns	GUUGCAGCUGCUGUGGUUGdTdT
si5UTR		siRNA knockdowns	GUCGUCAGUUAACUCGAAAdTdT
si5UTR	CG7439	siRNA knockdowns	UUUCGAGUUAACUGACGACdTdT
T7LaminB FOR		dsRNA knockdowns	TAATACGACTCACTATAGGGAGAGGAGAACACCATTCAGAGTC
T7LaminB REV	CG6944	dsRNA knockdowns	TAATACGACTCACTATAGGGAGACCATAAGGTCCTGGTACTCC
T7Dcr-2 FOR		dsRNA knockdowns	TAATACGACTCACTATAGGGGTTCCGCTTTGGTCAACAAT
T7Dcr-2 REV	CG6493	dsRNA knockdowns	TAATACGACTCACTATAGGGTGATCGTCTTTTCCATGCAG
T7Mi-2 FOR		dsRNA knockdowns	TAATACGACTCACTATAGGGATTTGCGTGGTAAATCGGAG
T7Mi-2 REV	CG8103	dsRNA knockdowns	TAATACGACTCACTATAGGGGTGGCTGGAGTGACGCTATT
P5_Nht promoter read bait_index4 FOR	CG15259	4C	*AATGATACGGCGACCACCGAGATCTACACTCTTTCCCTACACGACGCTCTTCCGATCT* TGACCA**ACCGTTTTTGACAACAGCA**
P5_Nht promoter read bait_index5 FOR	CG15259	4C	*AATGATACGGCGACCACCGAGATCTACACTCTTTCCCTACACGACGCTCTTCCGATCT* ACAGTG**ACCGTTTTTGACAACAGCA**
P5_Nht promoter read bait_index6 FOR	CG15259	4C	*AATGATACGGCGACCACCGAGATCTACACTCTTTCCCTACACGACGCTCTTCCGATCT* GCCAAT**ACCGTTTTTGACAACAGCA**
P5_Nht promoter read bait_index7 FOR	CG15259	4C	*AATGATACGGCGACCACCGAGATCTACACTCTTTCCCTACACGACGCTCTTCCGATCT* CAGATC**ACCGTTTTTGACAACAGCA**
P5_Nht promoter read bait_index8 FOR	CG15259	4C	*AATGATACGGCGACCACCGAGATCTACACTCTTTCCCTACACGACGCTCTTCCGATCT* ACTTGA**ACCGTTTTTGACAACAGCA**
P5_Nht promoter read bait_index9 FOR	CG15259	4C	*AATGATACGGCGACCACCGAGATCTACACTCTTTCCCTACACGACGCTCTTCCGATCT* GATCAG**ACCGTTTTTGACAACAGCA**
P7_Nht promoter non_reading REV	CG15259	4C	*CAAGCAGAAGACGGCATACGAGAT* **CCCTCTATGGCCCACTAACTC**

Underline: 4C primer index
Bold: 4C primer hybridization region
Italic: 4C primer P5 region
Italic and underline: 4C primer P7 region
