# Supplementary material for: Argonaute2 and LaminB modulate gene expression by controlling chromatin topology
Source: PLoS Genet. 2018 Mar 12;14(3):e1007276. doi: 10.1371/journal.pgen.1007276 (PMC5864089; doi:10.1371/journal.pgen.1007276)
Supplement: S2 Table — (PDF) [file pgen.1007276.s006.pdf]

Table S2 (related to figure 2, 4 and S1). Full list of affected genes in mRNA-seq and neuRNA-seq analyses

mRNA-Seq libraries_DESeq v2

mRNA-Seq libraries_DESeq v1

name	name	AGO2^{51B}_vs_wt_baseMean	AGO2^{51B}_vs_wt_log2FoldChange	AGO2^{51B}_vs_wt_padj	AGO2$^{51B/+}$_vs_wt_baseMean	AGO2$^{51B/+}$_vs_wt_log2FoldChange	AGO2$^{51B/+}$_vs_wt_padj	AGO2^{V966M}_vs_AGO2^{51B}_baseMean	AGO2^{V966M}_vs_AGO2^{51B}_log2FoldChange	AGO2^{V966M}_vs_AGO2^{51B}_padj
FBgn0031081	Neprilysin 3	714.5760679	-0.208533803	0.954321242	714.5760679	-0.232757236	0.852559484	714.5760679	0.552036286	0.251955746
FBgn0052826	transfer RNA:CR32826	0.488940935	0.117882697		0.488940935	0.089946065		0.488940935	-0.118713695	
FBgn0031085	CG9570 gene product from transcript	4.790823807	0.82546518	0.06241768	4.790823807	0.017704781		4.790823807	-0.847170419	0.028463796
FBgn0062565	Odorant receptor 19b	0.582111176	-0.025481858		0.582111176	0.013777447		0.582111176	0.037797928	
FBgn0031088	CG15322 gene product from transcrip	5.308032276	0.044143187	0.99764662	5.308032276	-0.045988555		5.308032276	0.022268244	0.988374361
FBgn0041626	Odorant receptor 19a	2.638028746	-0.11162003	0.994497358	2.638028746	0.065743299		2.638028746	0.350854027	0.663606104
FBgn0040784	CG15323 gene product from transcrip	1.541909247	0.259683763		1.541909247	0.109149548		1.541909247	-0.260753173	0.499824993
FBgn0031086	forkhead domain 19B	0.15702934	0		0.15702934	0		0.15702934	0.090208055	
FBgn0031082	CG17003 gene product from transcrip	1.374755308	0.24439092		1.374755308	0.05771442		1.374755308	-0.183204838	0.6608034
FBgn0031080	CG12655 gene product from transcrip	653.0921995	-0.126019632	0.993127879	653.0921995	-0.19446627	0.920812352	653.0921995	0.417958444	0.57402801
FBgn0052821	pseudo	2.876718977	-0.008883893	0.998322595	2.876718977	-0.096697874		2.876718977	-0.06929109	0.946532758
FBgn0053217	CG33217 gene product from transcrip	171.84702	-0.330567205	0.862079032	171.84702	0.356876389	0.752867275	171.84702	0.252714719	0.748198489
FBgn0052350	CG32350 gene product from transcrip	241.4622473	-0.070087753	0.9945211	241.4622473	0.407581883	0.701460763	241.4622473	0.586956806	0.314064035
FBgn0024733	Ribosomal protein L10	77182.93308	0.018738295	0.998322595	77182.93308	-0.030033102	0.990025502	77182.93308	-0.452032818	0.330235899
FBgn0040372	CG2995 gene product from transcript	897.8394411	-0.005322536	0.998322595	897.8394411	0.146483923	0.937166646	897.8394411	0.153576033	0.855940948
FBgn0000316	cinnamon	1377.856486	-0.160076287	0.959208615	1377.856486	0.042598837	0.984488479	1377.856486	0.260312345	0.589436819
FBgn0024989	CG3777 gene product from transcript	3068.216844	0.320847195	0.830069043	3068.216844	0.917286728	0.039140942	3068.216844	-0.653692768	0.15179619
FBgn0040371	CG12470 gene product from transcrip	10.01683409	1.065488465	0.110493717	10.01683409	0.234496635	0.914737519	10.01683409	-0.79913279	0.224917546
FBgn0029521	Odorant receptor 1a	2.223570457	0.213050361	0.958499814	2.223570457	0.02834362		2.223570457	-0.345705266	0.588766089
FBgn0004034	yellow	9.642013039	0.517422815	0.728029857	9.642013039	0.127483836	0.961113238	9.642013039	-0.557684096	0.451317304
FBgn0000022	achaete	42.32249192	-0.020997286	0.998322595	42.32249192	-0.159410775	0.944894567	42.32249192	0.002739131	0.998595283
FBgn0004170	scute	81.5427939	0.094284849	0.994497358	81.5427939	0.063535276	0.985550158	81.5427939	0.325591519	0.688482518
FBgn0002561	lethal of scute	12.98570235	-0.345489696	0.944197659	12.98570235	-0.240323562	0.919078655	12.98570235	0.049488766	0.974483245
FBgn0000137	asense	75.59319218	-0.02971384	0.998322595	75.59319218	-0.029203318	0.992952621	75.59319218	-0.137801483	0.872209701

name	AGO2^{V966M}_vs_wt_baseMean	AGO2^{V966M}_vs_wt_log2FoldChange	AGO2^{V966M}_vs_wt_padj	AGO2$^{V966M/+}$_vs_AGO2$^{51B/+}$_baseMean	AGO2$^{V966M/+}$_vs_AGO2$^{51B/+}$_log2FoldChange	AGO2$^{V966M/+}$_vs_AGO2$^{51B/+}$_padj	AGO2$^{V966M/+}$_vs_wt_baseMean	AGO2$^{V966M/+}$_vs_wt_log2FoldChange	AGO2$^{V966M/+}$_vs_wt_padj
FBgn0031081	714.5760679	0.343504694	0.775390444	714.5760679	-0.005532009	1	714.5760679	-0.238289245	1
FBgn0052826	0.488940935	0		0.488940935	-0.089850413		0.488940935	0	1
FBgn0031085	4.790823807	-0.021731898	1	4.790823807	-0.018480752	1	4.790823807	-0.000775962	1
FBgn0062565	0.582111176	0.012317291		0.582111176	-0.04933017		0.582111176	-0.035552713	1
FBgn0031088	5.308032276	0.066411973	0.993437727	5.308032276	0.183681018	1	5.308032276	0.13769243	1
FBgn0041626	2.638028746	0.239243637		2.638028746	0.198465107		2.638028746	0.264208369	1
FBgn0040784	1.541909247	0		1.541909247	-0.075734267		1.541909247	0.033415286	1
FBgn0031086	0.15702934	0.091533495		0.15702934	0.058704339		0.15702934	0.062803064	1
FBgn0031082	1.374755308	0.06118036		1.374755308	-0.057581633		1.374755308	0	1
FBgn0031080	653.0921995	0.291941676	0.907324809	653.0921995	-0.209704797	1	653.0921995	-0.404171055	1
FBgn0052821	2.876718977	-0.078176893		2.876718977	0.010736301	1	2.876718977	-0.085961576	1
FBgn0053217	171.84702	-0.077851017	0.986545988	171.84702	-0.428213783	1	171.84702	-0.071337377	1
FBgn0052350	241.4622473	0.516872495	0.590891726	241.4622473	-0.313080687	1	241.4622473	0.09450121	1
FBgn0024733	77182.93308	-0.433296073	0.537377049	77182.93308	0.062521039	1	77182.93308	0.032487936	1
FBgn0040372	897.8394411	0.148254254	0.957925315	897.8394411	-0.295282125	1	897.8394411	-0.148798191	1
FBgn0000316	1377.856486	0.100236715	0.962302558	1377.856486	0.064990746	1	1377.856486	0.107589582	1
FBgn0024989	3068.216844	-0.332848521	0.800787665	3068.216844	-0.497735323	0.952841016	3068.216844	0.419551421	1
FBgn0040371	10.01683409	0.266339867	0.933355004	10.01683409	-0.070531176	1	10.01683409	0.163965472	1
FBgn0029521	2.223570457	-0.132664746		2.223570457	-0.1961387		2.223570457	-0.167795039	1
FBgn0004034	9.642013039	-0.040274038	0.999894934	9.642013039	-0.093640158	1	9.642013039	0.033843697	1
FBgn0000022	42.32249192	-0.018258155	1	42.32249192	-0.234846255	1	42.32249192	-0.394257013	1
FBgn0004170	81.5427939	0.419878497	0.78671182	81.5427939	-0.393521487	1	81.5427939	-0.329986188	1
FBgn0002561	12.98570235	-0.29600007	0.932912729	12.98570235	0.065734431	1	12.98570235	-0.174589139	1
FBgn0000137	75.59319218	-0.167515972	0.949813094	75.59319218	-0.443500788	1	75.59319218	-0.47270409	1

name	AGO2^{51B}_vs_wt_baseMean	AGO2^{51B}_vs_wt_log2FoldChange	AGO2^{51B}_vs_wt_padj	AGO2$^{51B/+}$_vs_wt_baseMean	AGO2$^{51B/+}$_vs_wt_log2FoldChange	AGO2$^{51B/+}$_vs_wt_padj	AGO2^{V966M}_vs_AGO2^{51B}_baseMean	AGO2^{V966M}_vs_AGO2^{51B}_log2FoldChange	AGO2^{V966M}_vs_AGO2^{51B}_padj
FBgn0031081	702.6943622	-0.246482324	0.958744831	633.3502413	-0.27361105	0.99958454	848.3863153	0.63644006	0.215326986
FBgn0052826	0.746744057	inf	1	0.46059623	inf	0.961728726	0.771610487	#NAME?	0.923518833
FBgn0031085	11.76470723	6.230096285	2.69E-05	0.371167882	0.709140581	1	11.99714121	#NAME?	5.01E-05
FBgn0062565	0.545843819	-0.937282063	1	0.715028396	0.262081989	1	0.575674516	0.981872437	1
FBgn0031088	5.124252862	0.273544631	1	4.044333986	-0.129761593	1	6.007618858	0.101933194	1
FBgn0041626	1.587128013	-0.872606811	1	2.01279222	0.213688995	1	2.568389014	1.779701085	0.855926354
FBgn0040784	2.621622766	inf	0.061490995	0.92119246	inf	0.579672959	2.709031885	#NAME?	0.098390684
FBgn0031086	0			0			0.269306418	inf	1
FBgn0031082	2.434363997	inf	0.076652508	0.46059623	inf	0.977158798	3.088787966	-2.13360478	0.601557042
FBgn0031080	671.0888376	-0.174274808	1	593.4036945	-0.259031458	1	800.7913631	0.543843832	0.626449208
FBgn0052821	3.63177222	-0.209639856	1	2.847312287	-0.708819704	1	3.077924022	-0.379703777	1
FBgn0053217	155.8704006	-0.403164591	0.940932383	188.5871202	0.420164766	0.957597231	154.7641073	0.300496176	0.825927076
FBgn0052350	200.688736	-0.094504019	1	226.8842128	0.493536598	0.803494855	265.9510949	0.723979493	0.338659124
FBgn0024733	84163.27507	0.009782291	1	75146.50115	-0.039866557	1	74655.0035	-0.491988974	0.800302582
FBgn0040372	893.6423393	-0.014223231	1	866.7320851	0.167046084	1	982.6570658	0.187570721	0.882597223
FBgn0000316	1310.818687	-0.183108406	1	1284.349249	0.038747373	1	1410.592753	0.291320927	0.811059765
FBgn0024989	2759.053311	0.358156811	1	3362.897647	1.041761798	0.008629496	2557.264107	-0.744780603	0.139157267
FBgn0040371	16.39381365	3.614660019	0.001804791	3.890535518	1.294103082	0.87403363	19.34483166	-2.088231763	0.070422902
FBgn0029521	3.514717881	1.167626388	1	2.226481503	0.331008595	1	3.051743821	-2.222165349	0.48029904
FBgn0004034	13.58609866	1.645763036	0.250524155	7.136323056	0.470892346	0.986767242	13.6271142	-1.832072264	0.231713589
FBgn0000022	47.52610764	-0.070115365	1	40.83673148	-0.239264635	1	48.40268233	0.028977043	1
FBgn0004170	81.7616447	0.123512468	1	73.60293153	0.096034613	1	102.91897	0.418356291	0.880781593
FBgn0002561	14.06929545	-0.753317894	1	13.74263912	-0.486694583	1	11.46766056	0.16148315	1
FBgn0000137	84.63003117	-0.041842307	1	77.13597091	-0.034065862	1	82.19762023	-0.139976699	1

name	AGO2^{V966M}_vs_wt_baseMean	AGO2^{V966M}_vs_wt_log2FoldChange	AGO2^{V966M}_vs_wt_padj	AGO2$^{V966M/+}$_vs_AGO2$^{51B/+}$_baseMean	AGO2$^{V966M/+}$_vs_AGO2$^{51B/+}$_log2FoldChange	AGO2$^{V966M/+}$_vs_AGO2$^{51B/+}$_padj	AGO2$^{V966M/+}$_vs_wt_baseMean	AGO2$^{V966M/+}$_vs_wt_log2FoldChange	AGO2$^{V966M/+}$_vs_wt_padj
FBgn0031081	961.9577171	0.404799915	1	602.9673426	-0.006066786	1	750.5026167	-0.258621008	1
FBgn0052826	0			0.485596418	#NAME?	1	0		
FBgn0031085	0.167536855	#NAME?	1	0.38117895	-0.811114608	1	0.322031865	-0.07486444	1
FBgn0062565	0.796685973	0.057153682	1	0.548848938	-1.568627278	1	0.543258696	-1.300882418	1
FBgn0031088	5.811808063	0.392480746	1	5.249545722	0.659670084	1	6.126008925	0.558039827	1
FBgn0041626	3.22942325	0.921282178	1	3.108054439	0.790009517	1	3.339790365	1.019558757	1
FBgn0040784	0			1.247954317	-1.811114608	1	0.313678288	inf	1
FBgn0031086	0.285396807	inf	1	0.138380741	inf	1	0.156839144	inf	1
FBgn0031082	0.609348473	inf	1	0.485596418	#NAME?	1	0		
FBgn0031080	890.3962773	0.382778609	1	521.3761519	-0.266303633	1	650.7100239	-0.506047836	1
FBgn0052821	3.541618245	-0.576199819	1	2.331029743	0.065992261	1	3.450832105	-0.631787435	1
FBgn0053217	187.0869668	-0.087785057	1	193.219534	-0.515749806	1	185.5727881	-0.075517105	1
FBgn0052350	288.3494393	0.64488474	0.814247352	246.7969173	-0.385049314	1	232.6198797	0.130299769	1
FBgn0024733	78498.46297	-0.469160242	1	80064.64787	0.071745508	1	91700.56834	0.050949932	1
FBgn0040372	1042.803409	0.188874938	1	862.4942401	-0.34761567	1	912.2473671	-0.158492505	1
FBgn0000316	1580.643239	0.12299723	1	1407.116285	0.0732692	1	1567.07627	0.133673491	1
FBgn0024989	2327.146565	-0.371065865	0.892859508	3996.743534	-0.565018719	0.643861589	3127.681052	0.499378511	1
FBgn0040371	5.248398554	1.543918358	1	5.383033335	-0.238599605	1	4.12745349	1.086247604	1
FBgn0029521	1.743551596	-1.03886422	1	1.583294294	-2.239028282	1	1.470027332	-1.882219362	1
FBgn0004034	6.745005689	-0.171102526	1	7.808487773	-0.346134779	1	7.431776588	0.14602705	1
FBgn0000022	52.38617781	-0.028441187	1	35.75019371	-0.30063563	1	44.35382132	-0.52138469	1
FBgn0004170	104.9536178	0.55657594	1	67.33868028	-0.5541416	1	72.92117933	-0.437466036	1
FBgn0002561	16.02577387	-0.578412993	1	12.77797612	0.162453578	1	17.1609245	-0.305840207	1
FBgn0000137	88.11496556	-0.167150305	1	68.21324774	-0.517175487	1	77.91677327	-0.529545395	1

name	Dcr-2 KD_baseMean	Dcr-2 KD_log2FoldChange	Dcr-2 KD_padj	LaminB KD_baseMean	LaminB KD_log2FoldChange	LaminB KD_padj	AGO2 si5UTR KD_baseMean	AGO2 si5UTR KD_log2FoldChange	AGO2 si5UTR KD_padj
FBgn0031081	5.167830619	-0.06658591		6.711259036	-0.00512418	0.982958257	5.545122463	-0.022595825	
FBgn0052826	3.043387557	-0.092635097		3.860751096	0.141761053	0.301102406	2.333320292	-0.015971015	
FBgn0031085	14.77932127	-0.013077271		9.341867045	0.134327431	0.601092964	7.764948006	0.037464178	
FBgn0062565	34.02224055	-0.270599858		91.73712737	0.459195634		18.80671045	-0.010367068	
FBgn0031088	12.85538509	-0.020414834		11.05854486	0.236486581	0.30969007	7.779043212	0.011364839	
FBgn0041626	1.353311605	0.05258666		1.321688705	-0.028183		0.23630229	0.009874257	
FBgn0040784	0.531573386	0		0			0		
FBgn0031086	7.091198171	-0.427009296		6.740457329	-0.014630199	0.957045486	3.115857345	-0.006220805	
FBgn0031082	0.76441312	0.008255282		0.09831319	0		0		
FBgn0031080	0			0			0.153593788	0	
FBgn0052821	0.096844376	0.031551369		0			0		
FBgn0053217	702.6882567	-0.401458159	0.022731577	848.2696154	0.008852023	0.971178934	1665.248799	0.202130902	0.585013541
FBgn0052350	485.6516313	-0.202304788	0.376638829	692.3009708	-0.016303559	0.950118653	693.7538342	0.052061777	0.973607068
FBgn0024733	1715.692883	-0.128426281	0.562945002	2432.944663	-0.095624195	0.558713002	4360.581906	-0.10116325	0.855817261
FBgn0040372	1584.456575	-0.006930732	0.980686242	2909.184097	0.124645895	0.177270144	3314.128999	0.002250144	0.997423732
FBgn0000316	366.2472538	-0.233619333	0.414458978	443.3945117	0.048367604	0.855149242	709.6667174	-0.014136861	0.992954883
FBgn0024989	312.9504244	-0.201105491		862.5671089	0.015260917	0.953266711	1102.138132	0.322123778	0.216760414
FBgn0040371	0			0			0		
FBgn0029521	0			0			0		
FBgn0004034	25.8483073	-0.118458597		4.751810637	-0.004100024	0.985416745	7.921405702	-0.012940547	
FBgn0000022	0			0.249671637	-0.036814375		0		
FBgn0004170	0			0.196626381	0		0		
FBgn0002561	0			0			0		
FBgn0000137	0.236120962	0		0			0		

name	AGO2 si5UTR KD + AGO2 rescue_baseMean	AGO2 si5UTR KD + AGO2 rescue_log2FoldChange	AGO2 si5UTR KD + AGO2 rescue_padj	AGO2 si5UTR KD + AGO2^{V966M} rescue_baseMean	AGO2 si5UTR KD + AGO2^{V966M} rescue_log2FoldChange	AGO2 si5UTR KD + AGO2^{V966M} rescue_padj	AGO2 siAGO2 KD_baseMean	AGO2 siAGO2 KD_log2FoldChange	AGO2 siAGO2 KD_padj
FBgn0031081	5.545122463	-0.012367562		5.545122463	0.018041567		6.711259036	-0.045255674	
FBgn0052826	2.333320292	-0.002405587		2.333320292	-0.016083062		3.860751096	0.065558314	
FBgn0031085	7.764948006	0.016558078		7.764948006	-0.013368072		9.341867045	0.241163454	
FBgn0062565	18.80671045	-0.041513758		18.80671045	-0.013839554		91.73712737	0.205087275	
FBgn0031088	7.779043212	0.021780708		7.779043212	-0.009506496		11.05854486	0.31148552	0.26132523
FBgn0041626	0.23630229	0		0.23630229	0.00814602		1.321688705	-0.08298988	
FBgn0040784	0			0			0		
FBgn0031086	3.115857345	0.035455253		3.115857345	-0.007465649		6.740457329	0.049763485	
FBgn0031082	0			0			0.09831319	0.026134006	
FBgn0031080	0.153593788	0.015175241		0.153593788	0		0		
FBgn0052821	0			0			0		
FBgn0053217	1665.248799	0.131038635		1665.248799	0.172326161	0.536383732	848.2696154	-0.319461782	0.074341794
FBgn0052350	693.7538342	0.005155258		693.7538342	0.216184905		692.3009708	0.105888149	0.720769109
FBgn0024733	4360.581906	-0.150857041	0.748208621	4360.581906	-0.070301867	0.82449356	2432.944663	0.193454218	0.268705185
FBgn0040372	3314.128999	0.124436861	0.791385614	3314.128999	-0.037417889	0.924889426	2909.184097	0.091853658	0.487102492
FBgn0000316	709.6667174	-0.017748568		709.6667174	0.24634163		443.3945117	0.054436269	0.887918901
FBgn0024989	1102.138132	0.367710514		1102.138132	0.313047632	0.19583665	862.5671089	0.525083265	0.000604251
FBgn0040371	0			0			0		
FBgn0029521	0			0			0		
FBgn0004034	7.921405702	0.009443731		7.921405702	0.006587941		4.751810637	0.029969786	
FBgn0000022	0			0			0.249671637	-0.037561038	
FBgn0004170	0			0			0.196626381	0.034578082	
FBgn0002561	0			0			0		
FBgn0000137	0			0			0		

FBgn0061196	Syntaxin Interacting Protein 3	1.16986396	0.196582398		1.16986396	-0.036347915		1.16986396	-0.192791501	
FBgn0029728	CG2861 gene product from transcript	7.089603747	0.91773378	0.200332711	7.089603747	-0.029774547	0.994998875	7.089603747	-0.755299991	0.244019922
FBgn0029726	CG15471 gene product from transcrip	0.246623758	0.110226517		0.246623758	0.072763218		0.246623758	-0.111232335	
FBgn0029725	CG2871 gene product from transcript	0.811750165	0.221487775		0.811750165	0		0.811750165	-0.222109149	
FBgn0025679	CG2932 gene product from transcript	6.961300795	-0.405352812	0.883797649	6.961300795	-0.372762383	0.825115861	6.961300795	0.235061298	0.829268771
FBgn0029724	CG15472 gene product from transcrip	1.817087019	0.537058267	0.301908942	1.817087019	-0.05395475		1.817087019	-0.603030819	0.116997986
FBgn0029745	Regulatory particle non-ATPase 13-rela	7.68604283	0.706458841	0.400863957	7.68604283	0.072248926	0.982932118	7.68604283	-0.538929884	0.440881287
FBgn0029746	CG15465 gene product from transcrip	0			0			0		
FBgn0003449	sans fille	1953.026241	-0.007963308	0.998322595	1953.026241	-0.108334444	0.920127339	1953.026241	0.042482818	0.945325089
FBgn0011761	deadhead	0.864996429	0.034043625		0.864996429	-0.164321189		0.864996429	-0.15083211	
FBgn0029753	CG4198 gene product from transcript	4.9132433	0.602842893	0.173517382	4.9132433	0		4.9132433	-0.604073577	0.101804951
FBgn0029755	Something about silencing 10 ortholog	994.6335512	-0.035228532	0.995120986	994.6335512	-0.127007936	0.906843805	994.6335512	0.065330405	0.916000079
FBgn0014024	RNA-binding protein 4F	1307.481599	0.128259674	0.921015845	1307.481599	-0.015074075	0.99193807	1307.481599	-0.087292901	0.824407549
FBgn0029754	CG15930 gene product from transcrip	4.012385468	0.381687405	0.875497214	4.012385468	0.201102243		4.012385468	-0.397484829	0.632529721
FBgn0029752	Thioredoxin T	17.61248684	1.261010263	0.006985035	17.61248684	-0.056483632	0.98613036	17.61248684	-1.345525492	0.001964612
FBgn0029751	CG17764 gene product from transcrip	5.273842872	0.504814898	0.792520678	5.273842872	0.230549638		5.273842872	-0.658787754	0.402632197
FBgn0029750	CG3323 gene product from transcript	66.6781016	0.016223891	0.998322595	66.6781016	-0.38542414	0.823613198	66.6781016	0.06503035	0.964965366
FBgn0029747	CG5062 gene product from transcript	6.545345464	0.82733799	0.216179522	6.545345464	0.307683734	0.830705318	6.545345464	-0.608252909	0.334243607
FBgn0052767	CG32767 gene product from transcrip	1381.123234	0.28258254	0.944197659	1381.123234	0.36996827	0.780760046	1381.123234	-0.071225442	0.951445241
FBgn0024332	Minichromosome maintenance 3	926.8796652	-0.098620917	0.994497358	926.8796652	-0.177262831	0.934529508	926.8796652	0.014695779	0.991032456
FBgn0026751	CG4208 gene product from transcript	694.5759117	-0.125856093	0.961442281	694.5759117	-0.331583268	0.455161017	694.5759117	0.146313462	0.741597812
FBgn0010014	Calcineurin B	247.205546	-0.165650449	0.9621418	247.205546	-0.10301848	0.945658579	247.205546	0.33187793	0.480058329
FBgn0029761	small conductance calcium-activated p	222.9503161	0.669201908	0.491837753	222.9503161	0.56397732	0.605242615	222.9503161	-0.747690448	0.244689113
FBgn0029763	CG4165 gene product from transcript	1780.162535	-0.005797683	0.998322595	1780.162535	0.01346415	0.995412313	1780.162535	0.13307586	0.774866133
FBgn0029765	CG16756 gene product from transcrip	257.6027067	-0.291293597	0.953755212	257.6027067	-0.587686195	0.597449784	257.6027067	-0.641958468	0.368421382
FBgn0029766	CG15784 gene product from transcrip	5473.382634	0.099863475	0.990957786	5473.382634	-0.03058668	0.990025502	5473.382634	-0.427871486	0.34586108
FBgn0029768	Sex peptide receptor	35.23828029	-0.081830682	0.9945211	35.23828029	0.03961032	0.991444605	35.23828029	0.234830528	0.81544658
FBgn0029770	CG4151 gene product from transcript	64.35387328	-0.687272444	0.522771287	64.35387328	-0.645791314	0.548609198	64.35387328	0.914340767	0.152526099
FBgn0029771	CG12730 gene product from transcrip	840.6305716	-0.125219239	0.988216049	840.6305716	-0.001192397	1	840.6305716	0.180075172	0.813022106
FBgn0026702	lethal (1) G0045	272.8284746	0.162140041	0.954654825	272.8284746	-0.148213669	0.902048244	272.8284746	-0.061689371	0.928437805
FBgn0004687	Myosin light chain cytoplasmic	6143.521362	-0.193667167	0.929926159	6143.521362	-0.165518357	0.881734127	6143.521362	0.310815207	0.499508344
FBgn0029778	Rho GTPase activating protein at 5A	586.6322581	-0.056069536	0.994497358	586.6322581	-0.162492063	0.878470454	586.6322581	0.177990219	0.740745928
FBgn0052762	CG32762 gene product from transcrip	21.38985385	-0.081995615	0.979231897	21.38985385	-0.081936568	0.928099148	21.38985385	0.120632466	0.755590939
FBgn0029769	fra mauro	45.09116988	0.176263489	0.989856236	45.09116988	0.16143354	0.951777831	45.09116988	-0.615433253	0.460239305
FBgn0029762	Nutrient Amino Acid Transporter 1	1022.178882	-0.203446759	0.925140457	1022.178882	-0.627025415	0.138631416	1022.178882	0.172272051	0.775729854
FBgn0029756	CG3309 gene product from transcript	275.0082631	-0.015440722	0.998322595	275.0082631	-0.0119223	0.997246866	275.0082631	0.127980767	0.82379504
FBgn0028474	CG4119 gene product from transcript	2535.303645	0.075793711	0.978641661	2535.303645	0.018152737	0.989553502	2535.303645	-0.070999994	0.85619666
FBgn0029785	Ribosomal protein L35	27585.329	0.058302827	0.993127879	27585.329	0.003890583	0.999078753	27585.329	-0.283634868	0.316213159
FBgn0003747	Gustatory receptor 5a	1.90064166	-0.40014538	0.755584388	1.90064166	-0.381256649		1.90064166	0.57732003	0.302729943
FBgn0000394	crossveinless	664.9188412	0.042495017	0.995584438	664.9188412	-0.031513293	0.990082242	664.9188412	-0.133183011	0.862114431
FBgn0029791	CG4096 gene product from transcript	1505.687151	-0.051379129	0.994876798	1505.687151	-0.206185892	0.858059696	1505.687151	-0.153861892	0.822679457
FBgn0029795	CG15773 gene product from transcrip	711.4231257	-1.304584803	0.038223032	711.4231257	-1.297704059	0.040174772	711.4231257	0.632018775	0.411272345
FBgn0029798	CG4078 gene product from transcript	362.2440412	0.210917675	0.872095585	362.2440412	0.278833681	0.660326519	362.2440412	-0.232503536	0.599020556
FBgn0029799	CG15772 gene product from transcrip	99.90530921	1.117546492	0.005284405	99.90530921	1.602931381	6.60E-06	99.90530921	-0.913560387	0.022550903
FBgn0029801	CG15771 gene product from transcrip	1355.020467	-0.012933264	0.998322595	1355.020467	-0.16070052	0.901194571	1355.020467	-0.171066571	0.780288004

FBgn0037128	CG14572 gene product from transcrip	800.5050029	-0.113631645	0.988216049	800.5050029	0.540725677	0.324338644
FBgn0037131	CG14564 gene product from transcrip	9.14127666	0.355948392	0.934448847	9.14127666	0.449089754	0.781198575
FBgn0037133	CG7370 gene product from transcript	0.344299739	-0.073610489		0.344299739	0.010030077	
FBgn0000560	eagle	16.18297807	-0.129703445	0.994497358	16.18297807	-0.276622912	0.904226848
FBgn0022936	Cyclin H	404.5530896	0.104695516	0.985645036	404.5530896	0.126387285	0.918711937
FBgn0037134	CG7407 gene product from transcript	364.5527905	0.290972231	0.630145904	364.5527905	0.131649449	0.894512522
FBgn0037135	CG7414 gene product from transcript	12198.79241	0.072842472	0.993127879	12198.79241	0.011827371	0.996958448
FBgn0037139	CG14563 gene product from transcrip	5.078661627	0.491398674	0.799062687	5.078661627	-0.234659075	
FBgn0037138	delta-1-Pyrroline-5-carboxylate dehydr	3777.061825	-0.344539007	0.547136884	3777.061825	0.212763299	0.788893834
FBgn0046301	CG7148 gene product from transcript	429.5517477	0.0596123	0.994497358	429.5517477	0.000728976	1
FBgn0052448	CG32448 gene product from transcrip	826.5439326	-0.130799832	0.985645036	826.5439326	-0.151940031	0.928209273
FBgn0037130	Syntrophin-like 1	406.2506193	-0.003985912	0.998322595	406.2506193	-0.231957779	0.853421559
FBgn0037129	CG14565 gene product from transcrip	10.00242473	-0.552667422	0.753198637	10.00242473	-0.194721591	0.941360428
FBgn0037127	CG14566 gene product from transcrip	1100.192809	-0.313945284	0.923470826	1100.192809	0.566955792	0.572501461
FBgn0037126	CG14567 gene product from transcrip	732.6955419	-0.649752686	0.416541104	732.6955419	-0.053946029	0.986877529
FBgn0037124	CG14568 gene product from transcrip	0.835149521	-0.105735887		0.835149521	-0.020685862	
FBgn0037123	CG14569 gene product from transcrip	15.06491164	-0.029035631	0.998322595	15.06491164	0.361215855	0.84787134
FBgn0037122	CG14570 gene product from transcrip	8.42572258	-0.070147603	0.995584438	8.42572258	0.084962188	0.983976136
FBgn0037120	CG11247 gene product from transcrip	650.0266881	-0.173710722	0.901320232	650.0266881	-0.209505035	0.758179274
FBgn0052447	CG32447 gene product from transcrip	475.1762193	0.199123031	0.967258404	475.1762193	-0.097146785	0.965276384
FBgn0037116	CG7158 gene product from transcript	908.3423232	-0.086556927	0.986913729	908.3423232	-0.270421846	0.645025488
FBgn0037114	Cuticular protein 78E	14761.29832	-0.021321285	0.998322595	14761.29832	0.097511757	0.965276384
FBgn0037109	Mediator complex subunit 1	1643.78483	0.205334511	0.833688398	1643.78483	-0.0239522	0.989197377
FBgn0037107	CG7166 gene product from transcript	61.51959976	0.267649572	0.931940294	61.51959976	0.340503913	0.776273613
FBgn0037105	CG7169 gene product from transcript	360.224858	-0.429523198	0.359156749	360.224858	-0.275025048	0.674722988
FBgn0037140	CG7442 gene product from transcript	268.2547101	-0.13945017	0.985645036	268.2547101	-0.531769005	0.461886218
FBgn0037142	CG14562 gene product from transcrip	482.3224341	0.291557687	0.589988601	482.3224341	0.206353012	0.757422387
FBgn0037143	CG7448 gene product from transcript	0.167676683	0		0.167676683	0.129953448	
FBgn0037144	CG7458 gene product from transcript	168.5625541	-0.521264758	0.476136719	168.5625541	-0.424886184	0.618704078
FBgn0029091	Chitin synthase 2	602.9567512	0.42916583	0.468371713	602.9567512	-0.053575757	0.980355578
FBgn0037146	CG7470 gene product from transcript	15080.59815	-0.364937427	0.763195434	15080.59815	-0.406428265	0.64241103
FBgn0000045	Actin 79B	32.00546359	-0.250884207	0.97064214	32.00546359	-0.053856212	0.988639252
FBgn0004514	Octopamine-Tyramine receptor	61.07995222	-0.251326768	0.954378726	61.07995222	-0.298468607	0.840710536
FBgn0037149	CG14561 gene product from transcrip	120.5278711	-0.019784234	0.998322595	120.5278711	-0.150477021	0.91558304
FBgn0000100	Ribosomal protein LP0	62941.73607	0.026417371	0.998322595	62941.73607	-0.135911818	0.941360428
FBgn0004414	male-specific opa containing gene	0			0		
FBgn0037151	CG7130 gene product from transcript	380.995026	-0.003208072	0.999629143	380.995026	0.14212331	0.948432738
FBgn0037150	CG7133 gene product from transcript	387.9718402	-0.185641885	0.923202573	387.9718402	0.11757303	0.920826993
FBgn0027532	CG7139 gene product from transcript	1270.487919	-0.241552244	0.6673632	1270.487919	-0.289404584	0.480967344
FBgn0037147	CG7140 gene product from transcript	1.522416278	0.265891302		1.522416278	-0.028425009	
FBgn0037141	CG7143 gene product from transcript	188.3963126	0.379467368	0.61314666	188.3963126	0.283285738	0.74209022
FBgn0052449	transfer RNA:CR32449	0			0		
FBgn0015075	Dead-box-1	1200.306989	-0.013972971	0.998322595	1200.306989	0.139231812	0.915954217
FBgn0004179	Cysteine string protein	2567.0975	-0.237154649	0.875484329	2567.0975	0.01857781	0.994998875

FBgn0031752	CG9044 gene product from transcript	1552.195538	-0.341250489	0.518399313	1552.195538	-0.334085197	0.514147235	1552.195538	0.524680093	0.075333399
FBgn0031753	CG13999 gene product from transcrip	11.3953066	0.116701589	0.994497358	11.3953066	-0.079035967	0.984721517	11.3953066	-0.269741452	0.812732685
FBgn0031751	CG9016 gene product from transcript	10.99183518	1.188815683	0.080281028	10.99183518	-0.052973783	0.988881869	10.99183518	-1.098389553	0.074537075
FBgn0031749	CG14000 gene product from transcrip	1.145313861	0.078083619		1.145313861	0.148179732		1.145313861	0.109027532	
FBgn0031747	CG9021 gene product from transcript	7480.535813	-0.094607806	0.994497358	7480.535813	-0.08536859	0.975563803	7480.535813	0.55714167	0.39537075
FBgn0002855	Accessory gland protein 26Aa	0			0			0		
FBgn0002856	Accessory gland protein 26Ab	0.147590939	0.153120924		0.147590939	0		0.147590939	-0.15413487	
FBgn0031745	CG8965 gene product from transcript	167.0368017	-0.159297829	0.965741401	167.0368017	-0.237476779	0.805951663	167.0368017	0.278333181	0.585555419
FBgn0051644	CG31644 gene product from transcrip	0.576719465	0.163379416		0.576719465	-0.022504916		0.576719465	-0.188791297	
FBgn0031739	CG14005 gene product from transcrip	231.3715258	-0.088144716	0.993466746	231.3715258	0.164279847	0.902690127	231.3715258	0.162439586	0.801980414
FBgn0031738	CG9171 gene product from transcript	632.2271325	-0.246626007	0.956325514	632.2271325	-0.075324213	0.980546945	632.2271325	0.396558284	0.584371038
FBgn0031737	obstructor-E	6763.763055	0.931245842	0.14968942	6763.763055	1.181023469	0.032594451	6763.763055	-2.322934685	2.04E-08
FBgn0031734	CG11147 gene product from transcrip	158.5323019	-0.105950783	0.990956945	158.5323019	0.113547437	0.945260084	158.5323019	-0.151248681	0.831073574
FBgn0031733	CG14006 gene product from transcrip	3.422367358	0.33494332	0.892718858	3.422367358	-0.044451376		3.422367358	-0.252240759	0.775729854
FBgn0031732	CG11149 gene product from transcrip	273.4790606	-1.207755639	0.039587844	273.4790606	-0.88719436	0.226113461	273.4790606	1.618297731	0.000746438
FBgn0031731	CG14007 gene product from transcrip	0			0			0		
FBgn0003980	Vitelline membrane 26Ab	0.429047604	-0.032460149		0.429047604	0.043069133		0.429047604	0.074525681	
FBgn0031757	CG9064 gene product from transcript	3.55477815	0.659129532	0.442952877	3.55477815	0.214400517		3.55477815	-0.282261183	0.752147832
FBgn0031758	Uncoupling protein 4B	0			0			0		
FBgn0001942	Eukaryotic initiation factor 4a	52991.32858	0.06722173	0.975999817	52991.32858	0.051841481	0.944730798	52991.32858	-0.125168765	0.632099554
FBgn0001941	infertile crescent	1391.413247	-0.217016464	0.804645589	1391.413247	0.164731766	0.828254838	1391.413247	-0.180582249	0.665000897
FBgn0031760	Tetraspanin 26A	367.3236562	-0.233674298	0.946165413	367.3236562	0.286247446	0.805951663	367.3236562	0.525059396	0.315212076
FBgn0031763	CG13996 gene product from transcrip	1.104786515	0.036410313		1.104786515	-0.040493824		1.104786515	-0.097697328	
FBgn0051643	CG31643 gene product from transcrip	191.4337227	-0.248953429	0.830069043	191.4337227	-0.111098108	0.933985422	191.4337227	0.335830154	0.425211365
FBgn0031768	CG12393 gene product from transcrip	581.6235852	-0.088673982	0.993127879	581.6235852	-0.23749605	0.808406364	581.6235852	0.065244384	0.932121811
FBgn0031769	CG9135 gene product from transcript	3201.306178	-0.207577175	0.792520678	3201.306178	-0.254796783	0.611001919	3201.306178	0.275502389	0.389473558
FBgn0031770	CG13995 gene product from transcrip	59.21348574	0.128219916	0.989856236	59.21348574	-0.295060684	0.825524805	59.21348574	-0.200552885	0.809452642
FBgn0031772	CG13994 gene product from transcrip	284.1229572	-0.160115421	0.958499814	284.1229572	-0.11854008	0.929364445	284.1229572	0.039704463	0.959053359
FBgn0031774	CG9147 gene product from transcript	322.4149525	-0.544410345	0.471493797	322.4149525	-0.25687569	0.845600278	322.4149525	0.023087852	0.984379213
FBgn0031776	CG13993 gene product from transcrip	1131.912982	0.162143084	0.961435662	1131.912982	0.246906668	0.776273613	1131.912982	-0.342695167	0.446236813
FBgn0051642	CG31642 gene product from transcrip	28.12927477	0.8213977	0.416434118	28.12927477	-0.114321879	0.972271065	28.12927477	-0.79586603	0.289746178
FBgn0031781	Actin-related protein 2/3 complex, sub	1673.721227	-0.157596081	0.975999817	1673.721227	-0.223055035	0.868380046	1673.721227	-0.040254272	0.968137957
FBgn0031784	CG9222 gene product from transcript	1.145301868	0.483099691		1.145301868	-0.0330316		1.145301868	-0.529748101	
FBgn0031785	CG13991 gene product from transcrip	2.810644473	0.628093874	0.162886844	2.810644473	0.039894821		2.810644473	-0.60453782	0.117419275
FBgn0040950	Mucin 26B	3034.078249	1.012170793	0.068079257	3034.078249	0.49511257	0.622434389	3034.078249	-0.52690098	0.413426185
FBgn0031786	CG13989 gene product from transcrip	1.695949852	0.342879239	0.44211428	1.695949852	0		1.695949852	-0.324886845	0.327314783
FBgn0051639	CG31639 gene product from transcrip	1.677667737	0.590822152	0.172567834	1.677667737	0		1.677667737	-0.593451655	0.10131577
FBgn0031782	WD repeat domain 79 homolog	512.4118378	-0.119482705	0.99157593	512.4118378	-0.335158605	0.776273613	512.4118378	0.103800116	0.917015308
FBgn0031779	CG9175 gene product from transcript	849.2354847	0.001772236	0.999629143	849.2354847	-0.005736438	1	849.2354847	-0.123984689	0.88049888
FBgn0031777	CG9154 gene product from transcript	322.9826387	-0.098147951	0.994151247	322.9826387	-0.068940006	0.975886897	322.9826387	0.055890412	0.95345605
FBgn0031775	CG9150 gene product from transcript	48.69441006	-0.510643414	0.718487731	48.69441006	-0.537476719	0.631464713	48.69441006	0.290092612	0.755220395
FBgn0031773	CG9144 gene product from transcript	412.9988711	-0.239886212	0.85659012	412.9988711	-0.040491417	0.985550158	412.9988711	-0.234164346	0.644035704
FBgn0031771	CG9140 gene product from transcript	5691.724209	-0.141241203	0.967901085	5691.724209	0.056636449	0.973807349	5691.724209	-0.395252952	0.31689515
FBgn0029161	slowmo	14.75139291	0.410887834	0.89316218	14.75139291	0.422400379	0.802601542	14.75139291	-0.426738798	0.657559242

[illegible]

FBgn0032126	CG13113 gene product from transcrip	0			0			0
FBgn0032127	CG13114 gene product from transcrip	0			0			0
FBgn0032131	CG3841 gene product from transcript	74.8623835	-1.094427652	0.158424264	74.8623835	-0.888000976	0.334627264	74.8623835
FBgn0019809	CG3858 gene product from transcript	16.06931337	-0.405654165	0.89770139	16.06931337	-0.323443584	0.872418896	16.06931337
FBgn0040070	thioredoxin-2	5287.512103	0.076454996	0.993127879	5287.512103	0.415463752	0.384916036	5287.512103
FBgn0032135	CG3881 gene product from transcript	363.0214612	0.139785997	0.97064214	363.0214612	0.043545827	0.984488479	363.0214612
FBgn0032136	Apolipoprotein lipid transfer particle	2146.164424	0.772182303	0.225440661	2146.164424	0.909246938	0.103538847	2146.164424
FBgn0053299	CG33299 gene product from transcrip	1.444361105	0.054822243		1.444361105	0.043859383		1.444361105
FBgn0015393	hoi-polloi	1354.540539	-0.096373413	0.994497358	1354.540539	-0.024256758	0.994998875	1354.540539
FBgn0011207	pelota	1550.411372	-0.064993792	0.993127879	1550.411372	0.041738118	0.978624719	1550.411372
FBgn0032138	CG4364 gene product from transcript	5831.781756	-0.041104656	0.994497358	5831.781756	-0.312173651	0.490926479	5831.781756
FBgn0051710	CG31710 gene product from transcrip	527.4449838	0.091078926	0.994497358	527.4449838	-0.190470861	0.894405628	527.4449838
FBgn0000273	cAMP-dependent protein kinase 1	6652.282113	0.412136031	0.484043024	6652.282113	0.686014096	0.069008875	6652.282113
FBgn0051882	CG31882 gene product from transcrip	1.183367019	0.320902084		1.183367019	0.070869371		1.183367019
FBgn0032132	CG4382 gene product from transcript	57.99234248	0.102341418	0.994497358	57.99234248	-0.260655356	0.886539694	57.99234248
FBgn0014179	glial cells missing	39.62193643	-0.068258465	0.995120986	39.62193643	-0.626801685	0.519316077	39.62193643
FBgn0051709	CG31709 gene product from transcrip	5.526864602	0.688888378	0.126078663	5.526864602	0.083371199		5.526864602
FBgn0028479	Mitochondrial trifunctional protein alp	8136.409863	0.101659633	0.972176266	8136.409863	0.18299724	0.770169252	8136.409863
FBgn0032129	junctophilin	5249.625265	0.267087346	0.747610941	5249.625265	0.50763559	0.182223667	5249.625265
FBgn0004868	GDP dissociation inhibitor	20908.4776	-0.0537958	0.99157593	20908.4776	0.048030564	0.960705837	20908.4776
FBgn0032139	CG13116 gene product from transcrip	58.00562298	0.779337012	0.407473733	58.00562298	-0.35940694	0.832797896	58.00562298
FBgn0032140	CG13117 gene product from transcrip	674.8647821	0.091886972	0.994497358	674.8647821	-0.0796129	0.975886897	674.8647821
FBgn0032143	CG4017 gene product from transcript	44.79130559	0.388756052	0.901865105	44.79130559	-0.042949288	0.99193807	44.79130559
FBgn0032144	CG17633 gene product from transcrip	20140.9743	-1.936201237	1.67E-08	20140.9743	-1.21075225	0.003238302	20140.9743
FBgn0032147	Inositol 1,4,5-triphosphate kinase 1	438.1607568	-0.313099269	0.496187114	438.1607568	-0.418428075	0.230263803	438.1607568
FBgn0032149	CG4036 gene product from transcript	280.350517	-0.12100447	0.985645036	280.350517	-0.1487967	0.919789045	280.350517
FBgn0032169	CG4709 gene product from transcript	466.3549137	-0.24959819	0.73499901	466.3549137	-0.302942332	0.551819009	466.3549137
FBgn0032170	CG4658 gene product from transcript	2164.856929	-0.312406288	0.704953107	2164.856929	-0.462511648	0.334244738	2164.856929
FBgn0032172	CG5850 gene product from transcript	1092.818575	0.013626297	0.998322595	1092.818575	0.056622174	0.972431711	1092.818575
FBgn0032171	CG5846 gene product from transcript	372.2714843	-0.263512475	0.949774689	372.2714843	-0.285509432	0.849954585	372.2714843
FBgn0032168	CG13126 gene product from transcrip	313.758133	0.112993416	0.979231897	313.758133	0.184756526	0.84759519	313.758133
FBgn0063449	U snoRNA host gene 2	240.0289375	-0.246303415	0.944197659	240.0289375	-0.196171983	0.907768258	240.0289375
FBgn0025881	ncRNA	14.52031404	0.028700895	0.998322595	14.52031404	0.046406595	0.991444605	14.52031404
FBgn0063389	ncRNA	3.25827567	0.06635048	0.994876798	3.25827567	-0.284097438		3.25827567
FBgn0063392	ncRNA	7.927737408	0.149235216	0.993466746	7.927737408	0.347633795	0.854378696	7.927737408
FBgn0063391	ncRNA	12.11723687	-0.167880514	0.991146172	12.11723687	0.125747245	0.968777026	12.11723687
FBgn0032150	CG13123 gene product from transcrip	95.40813013	0.022260978	0.998322595	95.40813013	0.069102958	0.980546945	95.40813013
FBgn0042174	pseudo	6014.336176	-0.143152731	0.985645036	6014.336176	-0.088695163	0.969441701	6014.336176
FBgn0011291	TBP-associated factor 11	679.0348808	-0.034350439	0.99564268	679.0348808	-0.383486573	0.538158573	679.0348808
FBgn0015035	Cytochrome P450-4e3	183.3867713	1.857022876	0.000316365	183.3867713	1.492794066	0.01095326	183.3867713
FBgn0028704	CG18660 gene product from transcrip	178.1690998	0.517030723	0.357503918	178.1690998	0.701357835	0.103120291	178.1690998
FBgn0000180	big brain	433.7614589	0.019691542	0.998322595	433.7614589	-0.197447419	0.870305939	433.7614589
FBgn0043456	CG4747 gene product from transcript	5383.306205	-0.164409185	0.755584388	5383.306205	0.027417316	0.980691994	5383.306205
FBgn0011584	Translocation protein 1	6900.454956	0.018929653	0.998322595	6900.454956	0.10398483	0.91558304	6900.454956
FBgn0032176	CG13127 gene product from transcrip	0.203911637	0.121748477		0.203911637	0		0.203911637
FBgn0027600	obstructor-B	2454.269898	0.427873354	0.443158719	2454.269898	0.610248391	0.131760469	2454.269898
FBgn0053302	Cuticular protein 31A	236.3225361	0.763021998	0.399353831	236.3225361	1.118957963	0.082608088	236.3225361
FBgn0032178	Serpin 31A	59.22770442	0.048639976	0.997117822	59.22770442	-0.160405738	0.945756244	59.22770442
FBgn0032187	CG4839 gene product from transcript	508.3191515	-0.271611366	0.86896518	508.3191515	-0.213538076	0.856635871	508.3191515
FBgn0032188	CG13137 gene product from transcrip	0.555414214	0.165729505		0.555414214	0		0.555414214
FBgn0005593	Ribosomal protein L7	47693.35073	-0.073093125	0.994497358	47693.35073	-0.198329011	0.878598552	47693.35073
FBgn0032194	CG4901 gene product from transcript	503.1987552	-0.116203088	0.966062204	503.1987552	-0.122374864	0.894512522	503.1987552
FBgn0032195	CG4908 gene product from transcript	233.6322082	-0.09156406	0.994497358	233.6322082	-0.122809043	0.946798903	233.6322082
FBgn0032198	CG4912 gene product from transcript	8230.424975	0.364183228	0.492420817	8230.424975	0.445634546	0.29117856	8230.424975

FBgn0032485	CG9426 gene product from transcript	1247.715241	-0.144555336	0.958499814	1247.715241	-0.084120021	0.948432738
FBgn0032484	kekkon4	11.01945125	0.037920649	0.998322595	11.01945125	-0.349299513	0.855089983
FBgn0004191	small nuclear RNA U2 at 34ABa	26.66445594	2.676933034	2.16E-08	26.66445594	2.713497555	3.39E-08
FBgn0032483	CG15482 gene product from transcrip	11.28009501	0.687335224	0.578761289	11.28009501	0.017318633	0.997246866
FBgn0032481	CG16972 gene product from transcrip	442.6174526	0.174375173	0.951477694	442.6174526	0.133818362	0.915954217
FBgn0032479	CG16974 gene product from transcrip	3556.955325	0.037304668	0.994876798	3556.955325	0.214005921	0.74624482
FBgn0051849	CG31849 gene product from transcrip	114.2547566	0.161129827	0.967957809	114.2547566	0.070405911	0.971833087
FBgn0032475	Scm-related gene containing four mbt c	1061.953497	0.034332828	0.9945211	1061.953497	0.046920012	0.968777026
FBgn0032474	DnaJ homolog	3001.487216	0.061391476	0.994497358	3001.487216	-0.516592489	0.376930898
FBgn0040972	CG16978 gene product from transcrip	0.275553816	0.137999561		0.275553816	0	
FBgn0032472	CG9928 gene product from transcript	97.41052241	-0.509124774	0.775393399	97.41052241	-0.467570347	0.750861134
FBgn0032517	CG7099 gene product from transcript	289.3869447	0.228930117	0.938791798	289.3869447	0.252289718	0.82823087
FBgn0032521	CG7110 gene product from transcript	485.4911301	-0.074789247	0.991146172	485.4911301	-0.008875995	0.997246866
FBgn0032522	CG16848 gene product from transcrip	0.147590939	0.153120924		0.147590939	0	
FBgn0026760	CG7121 gene product from transcript	73.27546428	-0.498718162	0.688566959	73.27546428	-0.189957445	0.922400604
FBgn0028942	CG16852 gene product from transcrip	1.541533203	0.251668695		1.541533203	-0.029175685	
FBgn0028941	CG16853 gene product from transcrip	1.779278252	0.581133866	0.382483187	1.779278252	0.162615358	
FBgn0028546	icarus	1931.177926	-0.264757623	0.496187114	1931.177926	0.297946468	0.368897813
FBgn0028513	CG9254 gene product from transcript	12.65727647	0.693583755	0.50090493	12.65727647	0.312020335	0.861074999
FBgn0023407	CG9239 gene product from transcript	1061.05461	-0.076824194	0.994497358	1061.05461	-0.007486165	0.99875141
FBgn0032530	CG9263 gene product from transcript	1.244198034	0.346673154		1.244198034	-0.051286362	
FBgn0028943	CG15638 gene product from transcrip	0.802037972	0.189467754		0.802037972	0.036006282	
FBgn0032525	CG16954 gene product from transcrip	1.940032977	0.397035023	0.475578063	1.940032977	0.101112995	
FBgn0032524	CG9267 gene product from transcript	1340.453585	-0.270119295	0.741912251	1340.453585	0.232685842	0.751958089
FBgn0032523	CG16956 gene product from transcrip	0.167676683	0		0.167676683	0.129953448	
FBgn0003983	Vitelline membrane 34Ca	4.266013287	0.485737924	0.798887624	4.266013287	0.198954145	
FBgn0032520	CG10859 gene product from transcrip	11.13391878	1.575957132	0.007753682	11.13391878	0.406954706	0.80742724
FBgn0032519	CG16957 gene product from transcrip	4.514171913	0.543061537	0.656359093	4.514171913	-0.136045481	
FBgn0032518	Ribosomal protein L24	29590.63447	-0.039334765	0.997459641	29590.63447	-0.000323798	1
FBgn0032516	CG9293 gene product from transcript	670.7396215	0.140181119	0.971565969	670.7396215	-0.277443729	0.723577978
FBgn0028527	CG18507 gene product from transcrip	1251.070846	0.342293926	0.491837753	1251.070846	-0.033603917	0.985707697
FBgn0028848	CG7311 gene product from transcript	9.766118794	0.738234376	0.42048989	9.766118794	-0.201180881	0.929089204
FBgn0051813	CG31813 gene product from transcrip	0.481108047	-0.010531644		0.481108047	-0.107495823	
FBgn0028541	CG7364 gene product from transcript	1680.631838	0.004983449	0.998322595	1680.631838	0.153627453	0.858958482
FBgn0051845	ncRNA	4.54983348	0.074112145	0.995120986	4.54983348	-0.165312168	
FBgn0024846	p38b MAP kinase	990.6970137	-0.322842718	0.736375381	990.6970137	-0.264884012	0.770169252
FBgn0028539	CG31731 gene product from transcrip	2891.044292	-0.333610603	0.595425293	2891.044292	-0.372802325	0.471717511
FBgn0028931	CG16863 gene product from transcrip	110.9028725	-0.22980254	0.936478824	110.9028725	-0.381014207	0.658774601
FBgn0028538	Sec71 ortholog (S. cerevisiae)	1788.515848	0.066039799	0.991865251	1788.515848	0.022773336	0.989553502
FBgn0027844	Carbonic anhydrase 1	1707.163651	-0.316945087	0.91488891	1707.163651	-0.045784648	0.989204991
FBgn0028919	CG16865 gene product from transcrip	404.0844264	0.112146392	0.978641661	404.0844264	-0.035029225	0.986232862
FBgn0001965	Son of sevenless	1371.91785	-0.060759712	0.994497358	1371.91785	-0.135362798	0.908085574
FBgn0015271	Origin recognition complex subunit 5	105.8975346	0.082515828	0.994497358	105.8975346	0.121471376	0.940118668
FBgn0026373	RNA polymerase II 33kD subunit	708.4192851	-0.095410549	0.994497358	708.4192851	-0.064263649	0.985227037

[illegible]

FBgn0032748	CG10492 gene product from transcript	898.2532214	0.040289798	0.995120986	898.2532214	-0.057262544	0.973213602	898.2532214	0.107773638	0.857489088	[illegible]
FBgn0032749	PH domain leucine-rich repeat protein	5.413376737	0.120815201	0.994497358	5.413376737	-0.276803334		5.413376737	-0.701395407	0.345811327	[illegible]
FBgn0032750	CG10495 gene product from transcript	179.4877402	-0.223875584	0.918693681	179.4877402	-0.087465062	0.959800155	179.4877402	0.077811374	0.923321566	[illegible]
FBgn0032751	CG17343 gene product from transcript	0			0			0			[illegible]
FBgn0032755	CG17344 gene product from transcript	0			0			0			[illegible]
FBgn0000075	alpha methyl dopa-resistant	212.8033284	0.148570536	0.979231897	212.8033284	0.45453341	0.59197336	212.8033284	-0.384746086	0.526397309	[illegible]
FBgn0002036	CG10561 gene product from transcript	224.728705	-0.036221899	0.995584438	224.728705	-0.000625296	1	224.728705	-0.246389803	0.64911504	[illegible]
FBgn0010300	brain tumor	3684.946793	0.026080743	0.998322595	3684.946793	0.029365915	0.993214629	3684.946793	0.217525914	0.787325583	[illegible]
FBgn0002031	lethal (2) 37Cc	4309.509667	0.098852224	0.971762817	4309.509667	0.313766175	0.405004019	4309.509667	-0.318390219	0.256214707	[illegible]
FBgn0000422	Dopa decarboxylase	11594.2341	0.68207449	0.466307248	11594.2341	-0.175100097	0.939574224	11594.2341	0.555108266	0.442153584	[illegible]
FBgn0002023	CG10699 gene product from transcript	195.852994	-0.225684426	0.899899871	195.852994	-0.377946894	0.58023242	195.852994	0.213952182	0.704647809	[illegible]
FBgn0032754	CG10700 gene product from transcript	0.491122759	0.060134873		0.491122759	0.08841693		0.491122759	-0.060959149		[illegible]
FBgn0053315	pseudo	0.120302357	-0.141479015		0.120302357	-0.1379702		0.120302357	0		[illegible]
FBgn0032753	CG17572 gene product from transcript	27.37590854	0.38469195	0.907005324	27.37590854	0.097958233	0.978014635	27.37590854	-0.929935199	0.172436444	[illegible]
FBgn0032752	CG10702 gene product from transcript	121.4413191	0.256179519	0.886446899	121.4413191	0.073710118	0.971833087	121.4413191	-0.242850794	0.685126471	[illegible]
FBgn0002021	lethal (2) 37Bb	839.1515124	0.005470011	0.998322595	839.1515124	-0.109041244	0.944289621	839.1515124	0.076766022	0.922496939	[illegible]
FBgn0032744	Tetratricopeptide repeat domain 19	305.2699014	0.17641735	0.966157244	305.2699014	0.211812648	0.867222024	305.2699014	-0.184565401	0.792164209	[illegible]
FBgn0051800	CG31800 gene product from transcript	457.1855756	-0.127471775	0.969368572	457.1855756	0.065808949	0.962732231	457.1855756	0.168943594	0.729193623	[illegible]
FBgn0001202	hook	1306.367189	-0.505117093	0.184775721	1306.367189	-0.410293747	0.363292428	1306.367189	0.531901367	0.084119744	[illegible]
FBgn0046302	CG10650 gene product from transcript	4092.253557	-0.855148308	0.108558434	4092.253557	-1.020831278	0.031971252	4092.253557	0.723893448	0.141137886	[illegible]
FBgn0032733	CG15170 gene product from transcript	327.6906371	-1.507502231	0.006212889	327.6906371	-1.67433281	0.001848894	327.6906371	1.278706852	0.020668017	[illegible]
FBgn0032731	Swiprosin-1	2189.495762	0.065827252	0.994497358	2189.495762	-0.318134878	0.71250717	2189.495762	0.238898779	0.693013553	[illegible]
FBgn0032729	CG10639 gene product from transcript	4710.973698	0.164131292	0.954654825	4710.973698	0.289153005	0.681177925	4710.973698	-0.978453104	0.000542182	[illegible]
FBgn0015772	Numb-associated kinase	1515.646599	-0.008125206	0.998322595	1515.646599	-0.115588218	0.911006433	1515.646599	-0.04726041	0.934827272	[illegible]
FBgn0032727	CG10623 gene product from transcript	4403.515206	0.059951537	0.994497358	4403.515206	-0.23547236	0.823870471	4403.515206	-0.151122993	0.824648713	[illegible]
FBgn0032726	CG10621 gene product from transcript	11708.56201	0.349394506	0.718487731	11708.56201	0.473549213	0.43745913	11708.56201	-0.659862217	0.086060492	[illegible]
FBgn0032725	CG10679 gene product from transcript	1493.370595	0.031085603	0.998322595	1493.370595	0.112333294	0.950726652	1493.370595	-0.065664338	0.942325187	[illegible]
FBgn0032723	short spindle 3	484.9277107	-0.057694629	0.9945211	484.9277107	0.006994584	0.999078753	484.9277107	0.307904405	0.612855827	[illegible]
FBgn0010309	CG10739 gene product from transcript	282.6266143	-0.085950343	0.994497358	282.6266143	0.150946067	0.915954217	282.6266143	-0.012234455	0.990410953	[illegible]
FBgn0015380	derailed	745.2466734	-0.034810704	0.995584438	745.2466734	-0.236699654	0.796573058	745.2466734	0.333556276	0.462119576	[illegible]
FBgn0032769	CG10750 gene product from transcript	1.395922787	0.40696677		1.395922787	0		1.395922787	-0.316755541	0.444862887	[illegible]
FBgn0032770	CG13086 gene product from transcript	244.6554999	-0.010475381	0.998322595	244.6554999	-0.540064655	0.577622102	244.6554999	-0.095678546	0.92985706	[illegible]
FBgn0032771	CG17349 gene product from transcript	6.58377631	0.070995625	0.994876798	6.58377631	-0.403416548	0.759743592	6.58377631	-0.118458171	0.917992921	[illegible]
FBgn0032772	CG17350 gene product from transcript	346.3032618	0.285262949	0.815249619	346.3032618	-0.017521614	0.995503607	346.3032618	-0.8010712	0.018362521	[illegible]
FBgn0051798	CG31798 gene product from transcript	8.881716699	-0.110026181	0.994497358	8.881716699	-0.496435107	0.729968898	8.881716699	0.13335363	0.920266072	[illegible]
FBgn0016675	Galactose-specific C-type lectin	859.2786426	0.550140993	0.131995514	859.2786426	0.255500295	0.709337076	859.2786426	-0.852924342	0.001407799	[illegible]
FBgn0032780	CG13085 gene product from transcript	89.542578	0.297315236	0.861736263	89.542578	0.475996692	0.522753428	89.542578	-0.327052551	0.589736185	[illegible]
FBgn0032779	CG16771 gene product from transcript	867.6987003	-0.732670039	0.096107085	867.6987003	-0.495420127	0.420604516	867.6987003	0.094676345	0.906818333	[illegible]
FBgn0032781	CG9987 gene product from transcript	1429.469196	-0.044557218	0.995584438	1429.469196	-0.146983283	0.920812352	1429.469196	-0.057030798	0.946532758	[illegible]
FBgn0032782	CG9994 gene product from transcript	345.1090421	-0.14459091	0.987458415	345.1090421	-0.261388482	0.858370075	345.1090421	-0.584942621	0.311564729	[illegible]
FBgn0032783	CG10237 gene product from transcript	1258.939138	0.561004115	0.082693937	1258.939138	0.170608298	0.837830666	1258.939138	0.250125728	0.526499451	[illegible]
FBgn0032775	CG17544 gene product from transcript	1106.189305	0.104208481	0.975999817	1106.189305	0.050893953	0.971833087	1106.189305	-0.377803064	0.235386786	[illegible]
FBgn0032774	CG17549 gene product from transcript	11013.7139	-0.147502423	0.923470826	11013.7139	0.0887627	0.930935455	11013.7139	-0.222123404	0.52112617	[illegible]
FBgn0032773	fondue	91133.43558	0.961763759	0.000370729	91133.43558	0.922332041	0.001070355	91133.43558	-1.579946795	2.22E-11	[illegible]
[illegible]	[illegible]	[illegible]	[illegible]	[illegible]	[illegible]	[illegible]	[illegible]	[illegible]	[illegible]	[illegible]	[illegible]

FBgn0037534	CG2781 gene product from transcript	1535.73763	0.18915338	0.953305045	1535.73763	0.469546495	0.433830131	1535.73763	-0.030721073	0.972953354
FBgn0037533	CD98 heavy chain	5787.861431	0.035262667	0.994497358	5787.861431	-0.186702874	0.657040735	5787.861431	-0.046626188	0.914421542
FBgn0014861	Minichromosome maintenance 2	689.9045568	-0.105646398	0.967901085	689.9045568	0.000399756	1	689.9045568	0.047380242	0.926618593
FBgn0010433	atonal	75.36190636	-0.090164171	0.994497358	75.36190636	0.073166561	0.973807349	75.36190636	0.092855112	0.917015308
FBgn0037562	CG11671 gene product from transcrip	84.49445196	-0.466484725	0.741912251	84.49445196	-0.629963317	0.479376545	84.49445196	0.096202837	0.931642474
FBgn0051464	CG31464 gene product from transcrip	495.457655	-0.035152055	0.997117822	495.457655	0.051336418	0.984488479	495.457655	0.304936116	0.598311685
FBgn0051463	CG31463 gene product from transcrip	299.0519314	-0.661964728	0.614907394	299.0519314	-0.69567174	0.532682942	299.0519314	-0.207910676	0.860566088
FBgn0037566	mitochondrial ribosomal protein L1	655.8134124	0.323788446	0.747413252	655.8134124	0.230399022	0.824086124	655.8134124	-0.347708293	0.477470482
FBgn0037572	CG11698 gene product from transcrip	0.586864518	0.225538349		0.586864518	0		0.586864518	-0.189939444	
FBgn0037573	CG7483 gene product from transcript	1621.476842	0.055222812	0.994497358	1621.476842	0.156919946	0.901886449	1621.476842	0.017747761	0.984938178
FBgn0062412	Copper transporter 1B	3753.22998	-0.275898976	0.967485396	3753.22998	-0.454973485	0.776273613	3753.22998	-0.296168023	0.787894032
FBgn0037576	Odorant receptor 85a	1.054924028	-0.103198796		1.054924028	-0.201244405		1.054924028	0.112102683	
FBgn0037577	CG7443 gene product from transcript	0.919424287	-0.075281761		0.919424287	0.021225507		0.919424287	0.007159849	
FBgn0004908	ADP ribosylation factor-like 2	278.6528233	0.122365099	0.97978208	278.6528233	0.02005204	0.994998875	278.6528233	0.06600394	0.932121811
FBgn0037579	CG18193 gene product from transcrip	2.55499714	0.693207242	0.316901578	2.55499714	0.344749485		2.55499714	-0.580567636	0.320424838
FBgn0037580	Dipeptidyl aminopeptidase III	5969.187661	-0.132089818	0.945259341	5969.187661	-0.053308419	0.963603221	5969.187661	0.176217001	0.627778334
FBgn0037581	CG7352 gene product from transcript	144.4182168	-0.093898375	0.993466746	144.4182168	-0.240803255	0.831996423	144.4182168	0.259410509	0.66826517
FBgn0020249	steamer duck	3013.298155	-0.013563266	0.998322595	3013.298155	0.035946348	0.98613036	3013.298155	-0.208355665	0.66826517
FBgn0037584	CG7963 gene product from transcript	0.139419282	-0.122978845		0.139419282	-0.119403538		0.139419282	0	
FBgn0037589	Odorant-binding protein 85a	1.838050781	0.104955174	0.994497358	1.838050781	0.265974114		1.838050781	-0.026594475	0.981764763
FBgn0037583	CG9684 gene product from transcript	342.8553392	-0.214513163	0.892718858	342.8553392	0.094693619	0.946243168	342.8553392	0.441925251	0.23904383
FBgn0037578	CG9601 gene product from transcript	367.3621867	-0.339237907	0.530023602	367.3621867	-0.278994723	0.64660916	367.3621867	-0.157205928	0.753516155
FBgn0040529	CG9603 gene product from transcript	6715.417617	0.20990937	0.952780524	6715.417617	0.378844846	0.653763819	6715.417617	-0.490551608	0.32244652
FBgn0003129	Pox meso	140.4486164	0.259427406	0.907005324	140.4486164	-0.39409227	0.656734573	140.4486164	0.057798581	0.950100544
FBgn0037574	Coenzyme Q biosynthesis protein 2	144.7440892	0.205176807	0.950976319	144.7440892	0.227895551	0.848588436	144.7440892	-0.287553192	0.623003385
FBgn0037571	CG11694 gene product from transcrip	1.015661802	0.22446327		1.015661802	0		1.015661802	-0.22505383	
FBgn0037570	CG11693 gene product from transcrip	3.938676664	1.136410185	0.072750638	3.938676664	0.353323619		3.938676664	-0.780891424	0.242519497
FBgn0037569	CG9615 gene product from transcript	326.7898185	-0.087693351	0.994497358	326.7898185	-0.13535959	0.949698854	326.7898185	-0.058364343	0.960959655
FBgn0037565	CG9626 gene product from transcript	382.0474515	0.225102849	0.89770139	382.0474515	-0.112352014	0.939574224	382.0474515	0.297422122	0.538681372
FBgn0037563	CG11672 gene product from transcrip	1012.2067	0.723338612	0.110493717	1012.2067	0.544433317	0.338892396	1012.2067	-0.765508264	0.04511649
FBgn0037561	CG9630 gene product from transcript	483.393754	-0.064290178	0.994497358	483.393754	-0.106255749	0.931783738	483.393754	0.058044308	0.926939932
FBgn0010173	Replication Protein A 70	1809.55514	-0.094772882	0.968528723	1809.55514	-0.114011865	0.871207708	1809.55514	0.016063343	0.976651192
FBgn0037592	CG11737 gene product from transcrip	2793.452431	-0.353286799	0.746362007	2793.452431	-0.624663831	0.237797141	2793.452431	0.892661541	0.016381639
FBgn0051454	CG31454 gene product from transcrip	35.70086437	-0.069074639	0.995120986	35.70086437	-0.158133835	0.949698854	35.70086437	0.257882135	0.799468289
FBgn0051259	CG31259 gene product from transcrip	99.69987966	-1.465547378	0.010222251	99.69987966	-1.051199618	0.142821625	99.69987966	0.240003675	0.824731176
FBgn0037594	Odorant receptor 85d	0.36423422	-0.066593239		0.36423422	-0.013298468		0.36423422	0.076860742	
FBgn0051460	CG31460 gene product from transcrip	6.507971501	0.431496425	0.85659012	6.507971501	0.451044142	0.761838381	6.507971501	-0.066926585	0.964274138
FBgn0037602	CG8021 gene product from transcript	150.0949127	0.258684188	0.936159146	150.0949127	0.104875691	0.961378425	150.0949127	-0.506964175	0.383973784
FBgn0037603	CG11753 gene product from transcrip	401.9757007	-0.267565952	0.908816464	401.9757007	-0.531066673	0.462059678	401.9757007	0.02855221	0.980064295
FBgn0037606	CG8032 gene product from transcript	650.3189159	-0.041604896	0.994876798	650.3189159	0.190335658	0.821630922	650.3189159	0.048488079	0.939643062
FBgn0037607	CG8036 gene product from transcript	10338.05298	0.325416229	0.75390685	10338.05298	0.616059392	0.194370373	10338.05298	-0.856743426	0.012810067
FBgn0012024	transfer RNA:tyr1:85Aa	0.100708642	0		0.100708642	0		0.100708642	0.114352451	
FBgn0012025	transfer RNA:tyr1:85Ab	0.626785086	-0.068840347		0.626785086	0.066685529		0.626785086	-0.056832794	
FBgn0012026	transfer RNA:tyr1:85Ac	0			0			0		
FBgn0000447	Dihydroorotate dehydrogenase	1700.376838	-0.332478156	0.434229377	1700.376838	-0.186558028	0.779527893	1700.376838	0.218520124	0.55108334
FBgn0024326	MAP kinase kinase 4	2327.388291	-0.198744814	0.891109763	2327.388291	-0.143682854	0.888454014	2327.388291	0.186815136	0.693408291
FBgn0037601	CG9716 gene product from transcript	1027.1522	-0.595945909	0.002600731	1027.1522	-0.188506016	0.686410293	1027.1522	-0.026219704	0.959516228
FBgn0012027	transfer RNA:tyr1:85Ad	1.638701072	-0.034410611	0.995682185	1.638701072	0.084425203		1.638701072	-0.039228442	0.9635783
FBgn0012028	transfer RNA:tyr1:85Ae	0.099032819	0		0.099032819	0.123176119		0.099032819	0	
FBgn0026399	Odorant receptor 85e	0			0			0		
FBgn0051450	mitochondrial ribosomal protein S18A	352.1962799	0.180913605	0.970858745	352.1962799	0.538424327	0.449407684	352.1962799	-0.436417428	0.442157025
FBgn0051453	CG31453 gene product from transcrip	897.5193221	-0.037151274	0.995584438	897.5193221	-0.206580247	0.823463209	897.5193221	0.432182358	0.25608297
FBgn0037591	Odorant receptor 85c	0.921607398	-0.058385935		0.921607398	-0.055040473		0.921607398	0.130576605	
FBgn0037590	Odorant receptor 85b	0.436413636	0.097176037		0.436413636	-0.043477069		0.436413636	-0.076099594	
FBgn0037608	mitochondrial ribosomal protein L19	743.5684212	-0.005878182	0.998322595	743.5684212	-0.127744466	0.915954217	743.5684212	0.027902691	0.97166259
FBgn0037610	CG8043 gene product from transcript	203.6241482	-0.125502406	0.988354972	203.6241482	-0.316990672	0.770169252	203.6241482	-0.129367788	0.877510679
FBgn0037611	CG11755 gene product from transcrip	78.09579634	0.161309962	0.981763935	78.09579634	0.017326032	0.997246866	78.09579634	0.326911332	0.657559242
FBgn0037612	CG8112 gene product from transcript	0			0			0		
FBgn0037614	CG8116 gene product from transcript	311.9826177	0.052435271	0.994497358	311.9826177	0.262821074	0.711535649	311.9826177	0.199200601	0.686993939
FBgn0037615	CG11760 gene product from transcrip	25.86061145	0.197406583	0.985645036	25.86061145	0.133498288	0.964908204	25.86061145	-0.193182965	0.872209701
FBgn0037616	CG8136 gene product from transcript	25.4704801	0.975119453	0.220445206	25.4704801	0.058699843	0.988368947	25.4704801	-1.219368214	0.038821014
FBgn0037617	CG8145 gene product from transcript	392.6092565	-0.16528282	0.89770139	392.6092565	0.183743899	0.776778093	392.6092565	0.485440186	0.053142145
FBgn0037618	CG11762 gene product from transcrip	27.55215113	0.18686182	0.985645036	27.55215113	-0.20404344	0.933048047	27.55215113	0.084609981	0.947573257

[illegible]

FBgn0003312	shadow	853.3239388	0.177829	0.953941411	853.3239388	-0.24547486	0.79351381	853.3239388	-0.122861872	0.85102175
FBgn0051368	CG31368 gene product from transcrip	656.78517	0.039130218	0.995584438	656.78517	0.144749551	0.918428126	656.78517	0.124854315	0.859683257
FBgn0037964	CG14731 gene product from transcrip	2.077861445	0.360075361	0.850533132	2.077861445	0.119696336		2.077861445	-0.09925256	0.926031333
FBgn0051211	CG31211 gene product from transcrip	2907.16853	-0.026763051	0.995584438	2907.16853	-0.243602546	0.660326519	2907.16853	0.22731186	0.54219193
FBgn0013276	Heat-shock-protein-70Ab	105.8223145	0.995876337	0.097366476	105.8223145	0.418729417	0.733651912	105.8223145	-0.748388176	0.203277991
FBgn0000147	aurora	191.4474997	0.190870545	0.963243134	191.4474997	0.041410593	0.9872372	191.4474997	-0.119126002	0.883461408
FBgn0037970	Glutamate Carrier 2	0			0			0		
FBgn0037976	Tachykinin	225.2913346	-0.293640171	0.72740743	225.2913346	-0.231675525	0.776273613	225.2913346	-0.458795649	0.208536494
FBgn0037975	CG3397 gene product from transcript	4020.601442	0.175624681	0.976370706	4020.601442	0.339399071	0.780760046	4020.601442	-0.149852386	0.87115812
FBgn0037974	CG12224 gene product from transcrip	241.3367529	0.562880276	0.433061354	241.3367529	0.303171284	0.796573058	241.3367529	-0.490617573	0.370994156
FBgn0037973	CG18547 gene product from transcrip	1049.851124	-0.050909493	0.994497358	1049.851124	-0.189260753	0.821086229	1049.851124	-0.405190928	0.238339412
FBgn0037972	CG10005 gene product from transcrip	335.3318924	-0.183948743	0.952780524	335.3318924	-0.188718229	0.867818569	335.3318924	0.555140337	0.145194904
FBgn0013275	Heat-shock-protein-70Aa	34.62679925	0.66016773	0.266870502	34.62679925	0.293451912	0.797974284	34.62679925	-0.581889067	0.24412536
FBgn0037963	Cadherin 87A	1078.03629	-0.029955699	0.99723561	1078.03629	0.115381254	0.936207405	1078.03629	0.186030039	0.753482617
FBgn0037962	CG6971 gene product from transcript	3.637220654	0.826098421	0.207352888	3.637220654	-0.101164561		3.637220654	-0.869166714	0.09827435
FBgn0037960	methuselah-like 5	339.3027106	-0.390850074	0.685317231	339.3027106	-0.368877322	0.656953918	339.3027106	0.466408006	0.341502488
FBgn0037958	CG6962 gene product from transcript	213.2394536	0.011524327	0.998322595	213.2394536	-0.05742899	0.972773419	213.2394536	0.064023266	0.923846636
FBgn0037956	CG6959 gene product from transcript	1282.244623	-0.157814408	0.964395018	1282.244623	0.10308535	0.944730798	1282.244623	0.343541906	0.451317304
FBgn0037955	CG6950 gene product from transcript	2218.965732	-0.225654411	0.917640938	2218.965732	-0.024702619	0.993019482	2218.965732	-0.446477021	0.333815173
FBgn0051116	Chloride channel-a	1422.80279	-0.306886509	0.410788544	1422.80279	-0.332909465	0.32573729	1422.80279	-0.027959054	0.95961159
FBgn0025802	SET domain binding factor	712.426965	0.068024997	0.994497358	712.426965	0.08440865	0.951701663	712.426965	0.351576383	0.392211153
FBgn0037949	CG17360 gene product from transcrip	488.8446502	-0.085956767	0.993466746	488.8446502	0.081268574	0.961457172	488.8446502	0.272724343	0.605977425
FBgn0017581	CG17342 gene product from transcrip	12911.82484	0.081157634	0.994497358	12911.82484	-0.036717264	0.986877529	12911.82484	-0.089427094	0.906818333
FBgn0037979	GCC185 ortholog (H. sapiens)	749.9991329	-0.148184078	0.961435662	749.9991329	7.83E-05	1	749.9991329	0.024750876	0.974417377
FBgn0037981	CG3169 gene product from transcript	304.1275378	-0.177270371	0.952791788	304.1275378	-0.0509009	0.980691994	304.1275378	0.172435482	0.771119459
FBgn0037985	short spindle 5	3.535823357	0.9332386	0.041532639	3.535823357	-0.018003756		3.535823357	-0.920918161	0.02629836
FBgn0051361	CG31361 gene product from transcrip	148.8622162	0.515614952	0.399353831	148.8622162	0.226525779	0.84759519	148.8622162	-0.055991366	0.945796962
FBgn0011905	transfer RNA:lys5:87BC	0			0			0		
FBgn0037995	CG3809 gene product from transcript	1.058373923	0.21971951		1.058373923	0.026598465		1.058373923	-0.220309672	
FBgn0037994	CG4810 gene product from transcript	2.009507656	0.449394983	0.436800036	2.009507656	-0.069482507		2.009507656	-0.52812875	0.174560095
FBgn0037993	CG10095 gene product from transcrip	20.58070928	-0.096905941	0.994497358	20.58070928	-0.120111938	0.96950421	20.58070928	0.264428927	0.804735008
FBgn0037992	CG4702 gene product from transcript	10.19400329	0.365949808	0.924674154	10.19400329	0.193923349	0.941360428	10.19400329	-0.084651504	0.952769953
FBgn0037989	CG14741 gene product from transcrip	561.1933657	0.062369608	0.994151247	561.1933657	0.021635292	0.990025502	561.1933657	0.021216887	0.974417377
FBgn0037988	CG14740 gene product from transcrip	1.585887425	0.408243493		1.585887425	0		1.585887425	-0.348439773	0.420900623
FBgn0037987	CG14739 gene product from transcrip	2.023151947	0.362459245	0.503881835	2.023151947	-0.033367105		2.023151947	-0.400510085	0.264199091
FBgn0051358	CG31358 gene product from transcrip	0.099032819	0		0.099032819	0.123176119		0.099032819	0	
FBgn0037980	CG3313 gene product from transcript	556.2143096	-0.375926329	0.639837507	556.2143096	-0.142071114	0.917887901	556.2143096	0.280963569	0.596159573
FBgn0037978	CG3571 gene product from transcript	1474.063064	-0.00895577	0.998322595	1474.063064	0.012565078	0.997246866	1474.063064	-0.177933228	0.812804388
FBgn0051356	transfer RNA:CR31356	0			0			0		
FBgn0038000	CG10014 gene product from transcrip	3.612033328	0.909777949	0.065210238	3.612033328	0.106867803		3.612033328	-0.732901021	0.126477674
FBgn0038003	CG3916 gene product from transcript	5.035196222	0.194795182	0.985645036	5.035196222	0.330220854		5.035196222	0.27145991	0.801065406
FBgn0038008	CG3942 gene product from transcript	1.21916436	0.215806651		1.21916436	-0.007697503		1.21916436	-0.264010561	
FBgn0003651	seven up	1305.151688	0.196898041	0.865739954	1305.151688	0.175513058	0.824638819	1305.151688	-0.293998159	0.414294021
FBgn0038009	CG17738 gene product from transcrip	12055.80345	0.407118499	0.72740743	12055.80345	0.127480927	0.946392283	12055.80345	-0.282731446	0.683077841
FBgn0038011	CG4066 gene product from transcript	0	[illegible]	[illegible]	0	[illegible]	[illegible]	0	[illegible]	[illegible]
[illegible]	[illegible]	[illegible]	[illegible]	[illegible]	[illegible]	[illegible]	[illegible]	[illegible]	[illegible]	[illegible]

[illegible]

FBgn0038299	Serpin 88Eb	1175.040614	-0.329435843	0.704953107	1175.040614	0.26475899	0.755583988	1175.040614	[illegible]
FBgn0028984	Serpin 88Ea	2206.971898	-0.612810696	0.003841504	2206.971898	-0.323252955	0.332530839	2206.971898	[illegible]
FBgn0000047	Actin 88F	115.8942071	-0.080324932	0.995100492	115.8942071	0.105769238	0.974682104	115.8942071	[illegible]
FBgn0038342	CG14870 gene product from transcrip	5.158723043	0.407347423	0.68934315	5.158723043	-0.046668944		5.158723043	[illegible]
FBgn0019940	Rhodopsin 6	143.8100167	-0.530837992	0.690062146	143.8100167	-0.821005241	0.277551005	143.8100167	[illegible]
FBgn0038344	CG5205 gene product from transcript	822.8399442	-0.115442087	0.985645036	822.8399442	0.527778807	0.308582737	822.8399442	[illegible]
FBgn0038345	CG5213 gene product from transcript	2.114518159	0.350464199	0.540509922	2.114518159	0		2.114518159	[illegible]
FBgn0038350	CG18516 gene product from transcrip	40.88448212	-0.281861664	0.958499814	40.88448212	-0.092803641	0.977764842	40.88448212	[illegible]
FBgn0038349	CG6045 gene product from transcript	3338.290049	-0.016639827	0.998322595	3338.290049	0.199162756	0.670759547	3338.290049	[illegible]
FBgn0038348	CG18519 gene product from transcrip	1528.993486	0.636543501	0.50659044	1528.993486	0.951616347	0.14171344	1528.993486	[illegible]
FBgn0051296	CG31296 gene product from transcrip	1.260749077	0.179823914		1.260749077	0.169364358		1.260749077	[illegible]
FBgn0038343	CG14871 gene product from transcrip	14.33856423	0.005127361	0.998873611	14.33856423	-0.03532939	0.99467272	14.33856423	[illegible]
FBgn0038341	ADAM metallopeptidase with thrombo	1208.932879	0.127018792	0.985645036	1208.932879	-0.130415773	0.939440198	1208.932879	[illegible]
FBgn0053207	CG33207 gene product from transcrip	181.0436762	0.088197877	0.994497358	181.0436762	0.110417792	0.953310894	181.0436762	[illegible]
FBgn0051446	CG31446 gene product from transcrip	183.7349771	-0.547493987	0.732187889	183.7349771	-0.369803961	0.827267758	183.7349771	[illegible]
FBgn0038353	CG5399 gene product from transcript	3372.832921	-1.092518898	0.010545702	3372.832921	-0.80144139	0.130896806	3372.832921	[illegible]
FBgn0038354	CG5404 gene product from transcript	174.3025741	0.464405287	0.708898746	174.3025741	0.219660011	0.904193924	174.3025741	[illegible]
FBgn0038355	CG4520 gene product from transcript	2.509045964	0.380381976	0.372292686	2.509045964	0		2.509045964	[illegible]
FBgn0038356	CG12784 gene product from transcrip	0.295181878	0.186873145		0.295181878	0		0.295181878	[illegible]
FBgn0038358	CG4525 gene product from transcript	6.667032762	0.405944608	0.886446899	6.667032762	-0.061151185	0.986877529	6.667032762	[illegible]
FBgn0038359	CG5614 gene product from transcript	3.054977609	0.716075085	0.145149986	3.054977609	-0.071434708		3.054977609	[illegible]
FBgn0038360	CG9590 gene product from transcript	1652.489733	0.02443545	0.995584438	1652.489733	0.093583396	0.917910585	1652.489733	[illegible]
FBgn0038361	CG9589 gene product from transcript	3.150449795	-0.025970027	0.998322595	3.150449795	0.049095099		3.150449795	[illegible]
FBgn0038363	Acylphosphatase 2	400.8982495	-0.053998328	0.994497358	400.8982495	-0.244461389	0.707086576	400.8982495	[illegible]
FBgn0038365	CG9593 gene product from transcript	43.24847519	0.531276572	0.678655521	43.24847519	0.521493486	0.638266824	43.24847519	[illegible]
FBgn0026616	CG4606 gene product from transcript	215.6616298	-0.15116232	0.979231897	215.6616298	-0.050218608	0.98613036	215.6616298	[illegible]
FBgn0038366	CG4576 gene product from transcript	3359.966071	0.055510191	0.994497358	3359.966071	-0.080456345	0.956864988	3359.966071	[illegible]
FBgn0038369	Actin-related protein 2/3 complex, sub	606.252456	0.140392733	0.943722455	606.252456	0.287273399	0.52933786	606.252456	[illegible]
FBgn0003483	spindle E	71.25272664	-0.20839609	0.966157244	71.25272664	0.071138985	0.980546945	71.25272664	[illegible]
FBgn0038373	CG4546 gene product from transcript	28.87534929	0.823921257	0.414892658	28.87534929	-0.483625486	0.755539431	28.87534929	[illegible]
FBgn0038377	CG9632 gene product from transcript	7.211875419	0.769019093	0.424609919	7.211875419	0.373416345	0.821634324	7.211875419	[illegible]
FBgn0051183	CG31183 gene product from transcrip	691.9649987	-0.127534397	0.975861786	691.9649987	0.294108612	0.669909079	691.9649987	[illegible]
FBgn0038376	Heavy metal tolerance factor 1	1764.025774	-0.123627761	0.926642056	1764.025774	-0.065989639	0.941482517	1764.025774	[illegible]
FBgn0038371	PSEA-binding protein 45kD	106.8308318	0.225656226	0.910739253	106.8308318	0.103266892	0.948432738	106.8308318	[illegible]
FBgn0017567	NADH:ubiquinone reductase 23kD sub	1173.27323	0.179291754	0.961031911	1173.27323	0.468493267	0.44962986	1173.27323	[illegible]
FBgn0003227	recombination-defective	115.2732146	-0.380370785	0.751994093	115.2732146	-0.216143475	0.879032978	115.2732146	[illegible]
FBgn0000246	crossover suppressor on 3 of Gowen	64.42478126	-0.14482432	0.99157593	64.42478126	0.233968481	0.909872422	64.42478126	[illegible]
FBgn0038357	CG5623 gene product from transcript	0.918408231	0.129999678		0.918408231	-0.046282636		0.918408231	[illegible]
FBgn0051294	CG31294 gene product from transcrip	1.62350033	0.249195116	0.58318903	1.62350033	0		1.62350033	[illegible]
FBgn0051292	pseudo	0.38059894	0.007266469		0.38059894	0.019872848		0.38059894	[illegible]
FBgn0038381	CG3303 gene product from transcript	396.0103719	-0.027944482	0.995584438	396.0103719	-0.14121081	0.868925451	396.0103719	[illegible]
FBgn0038385	CG4221 gene product from transcript	483.7469949	0.098918319	0.979231897	483.7469949	-0.08237508	0.94728821	483.7469949	[illegible]
FBgn0038387	black pearl	339.1478196	0.230054833	0.88909528	339.1478196	-0.007038781	0.998730566	339.1478196	[illegible]
FBgn0013334	Synapse-associated protein 47kD	2927.04432	0.138393805	0.948663162	2927.04432	0.162679438	0.824248109	2927.04432	[illegible]
FBgn0038389	CG5516 gene product from transcript	459.1115742	-0.18269424	0.965076657	459.1115742	-0.138398433	0.933048047	459.1115742	[illegible]
[illegible]	[illegible]	[illegible]	[illegible]	[illegible]	[illegible]	[illegible]	[illegible]	[illegible]	[illegible]

FBgn0051216	Nicotinamide amidase	84.96251381	0.337045453	0.815846652	84.96251381	0.317506603	0.770169252	84.96251381	-0.344913611	0.570314255
FBgn0038732	CG11391 gene product from transcrip	0			0			0		
FBgn0038733	CG11407 gene product from transcrip	57.75388334	-1.230122848	0.005097334	57.75388334	-0.02069142	0.995690426	57.75388334	-1.618868428	0.000275911
FBgn0038734	CG11453 gene product from transcrip	716.6276471	-1.249312935	0.006498776	716.6276471	-0.897479762	0.117048743	716.6276471	0.11275556	0.914341744
FBgn0012004	transfer RNA:val3b:92Bb	0.462915628	0		0.462915628	0.074767772		0.462915628	0	
FBgn0051215	transfer RNA:CR31215	0			0			0		
FBgn0051471	transfer RNA:CR31471	0			0			0		
FBgn0038731	CG11659 gene product from transcrip	218.6071904	1.359109135	0.041870768	218.6071904	1.26962918	0.071030119	218.6071904	-1.045700126	0.116845431
FBgn0038730	CG6300 gene product from transcript	217.7769165	-0.542647282	0.734121216	217.7769165	0.540066329	0.666425735	217.7769165	-1.205294913	0.039989065
FBgn0012003	transfer RNA:val3b:92Ba	0.391045333	0.069946267		0.391045333	0		0.391045333	0.057065004	
FBgn0038725	CG6184 gene product from transcript	218.7509481	-0.274755676	0.917248088	218.7509481	-0.407024569	0.676826614	218.7509481	0.156560891	0.855963153
FBgn0038723	CG6195 gene product from transcript	1034.553705	-0.019931985	0.998322595	1034.553705	0.265169025	0.79038145	1034.553705	-0.219450982	0.722553655
FBgn0038721	CG16718 gene product from transcrip	1783.072435	-0.157030622	0.966988857	1783.072435	0.361943677	0.606973943	1783.072435	0.10012148	0.890025177
FBgn0038720	CG6231 gene product from transcript	1573.440559	-0.279473428	0.78070356	1573.440559	-0.230324644	0.796615544	1573.440559	0.360098757	0.399093695
FBgn0051459	CG31459 gene product from transcrip	1.475909391	0.233889205		1.475909391	0		1.475909391	-0.234296855	0.410339177
FBgn0038735	CG4662 gene product from transcript	914.684635	-0.15941406	0.953941411	914.684635	0.145208316	0.897821035	914.684635	-0.227276217	0.63203614
FBgn0038737	CG11447 gene product from transcrip	324.7760131	-0.22624815	0.924867928	324.7760131	-0.234874776	0.832797896	324.7760131	-0.048374423	0.957060064
FBgn0038739	CG4686 gene product from transcript	336.5809698	-0.842834867	0.238229165	336.5809698	-0.23298512	0.907409646	336.5809698	-0.058383666	0.96433474
FBgn0038742	CG4703 gene product from transcript	2111.566375	0.118231915	0.979742185	2111.566375	0.204275826	0.846310358	2111.566375	-0.283591354	0.574389429
FBgn0014006	Protein kinase at 92B	1670.621436	0.052552155	0.993466746	1670.621436	0.079056891	0.929364445	1670.621436	-0.033569763	0.943484233
FBgn0038744	CG4733 gene product from transcript	288.3228916	0.296642885	0.752889814	288.3228916	0.226378813	0.805797107	288.3228916	-0.309203833	0.501079945
FBgn0038747	Rho GTPase activating protein at 92B	2168.930733	-0.169962846	0.917248088	2168.930733	-0.141363863	0.878786989	2168.930733	0.261297833	0.496463886
FBgn0038751	CG4770 gene product from transcript	7.574537294	-0.389229837	0.907835199	7.574537294	-0.116774099	0.971833087	7.574537294	-0.313211334	0.771458576
FBgn0038756	CG4783 gene product from transcript	133.9024261	2.162061579	2.42E-06	133.9024261	0.274759964	0.889017886	133.9024261	0.106891556	0.928437805
FBgn0051213	CG31213 gene product from transcrip	6.61613871	0.312745355	0.952632367	6.61613871	0.201426291	0.934188786	6.61613871	-0.222819622	0.84118154
FBgn0038760	Mediator complex subunit 25	506.7516925	-0.166998194	0.902424733	506.7516925	-0.279266035	0.591766255	506.7516925	0.127530899	0.780567916
FBgn0038766	CG4854 gene product from transcript	46.96822843	0.025278201	0.998322595	46.96822843	0.08533068	0.973524774	46.96822843	0.212069272	0.801065406
FBgn0038768	CG4936 gene product from transcript	875.7686048	-0.077245423	0.990262271	875.7686048	-0.209592027	0.770169252	875.7686048	0.05885315	0.92280061
FBgn0038772	midlife crisis	371.3378891	-0.08438289	0.985645036	371.3378891	0.002997211	1	371.3378891	0.091947926	0.855940948
FBgn0038771	CG4390 gene product from transcript	4026.814625	0.009959352	0.998322595	4026.814625	0.07221868	0.959619247	4026.814625	-0.331861498	0.399624684
FBgn0040571	CG17193 gene product from transcrip	23.3140399	-0.033875717	0.998322595	23.3140399	0.210818361	0.93199833	23.3140399	0.19691797	0.862439265
FBgn0038769	CG10889 gene product from transcrip	242.8705026	-0.281569327	0.822495995	242.8705026	-0.373907527	0.598570044	242.8705026	0.385569959	0.403015416
FBgn0038767	trade embargo	273.6694257	-0.001528576	0.999629143	273.6694257	-0.547143957	0.179438235	273.6694257	-0.150573227	0.78476579
FBgn0038765	CG4424 gene product from transcript	94.41214779	0.446201389	0.58180865	94.41214779	0.22774763	0.856185454	94.41214779	0.082287861	0.923336238
FBgn0038763	CG4433 gene product from transcript	760.3304238	-0.317132981	0.65362448	760.3304238	-0.066968006	0.965276384	760.3304238	0.029583002	0.968137957
FBgn0038761	CG17190 gene product from transcrip	468.4508549	-0.068298618	0.994497358	468.4508549	0.248971659	0.816067304	468.4508549	-0.298565333	0.596263914
FBgn0038755	Heparan sulfate 6-O-sulfotransferase	828.737979	0.151530578	0.966157244	828.737979	0.095511642	0.948432738	828.737979	0.23932079	0.644843315
FBgn0021776	miranda	396.7385322	0.377507583	0.679755311	396.7385322	-0.007362536	0.99875141	396.7385322	-0.355058417	0.494028019
FBgn0038753	CG4459 gene product from transcript	91.92280413	-0.552381905	0.483527893	91.92280413	-0.489631875	0.567302715	91.92280413	-0.517667975	0.372780361
FBgn0038752	CG4462 gene product from transcript	809.8178412	-0.514377077	0.280684921	809.8178412	-0.175891125	0.872253128	809.8178412	0.241984374	0.635965558
FBgn0038750	CG4465 gene product from transcript	885.5074529	-0.543398593	0.339396406	885.5074529	-0.388068986	0.605242615	885.5074529	-0.015145143	0.988374361
FBgn0038749	exit protein of rhodopsin and TRP	0			0			0		
FBgn0038746	Surfeit 6	1001.242045	-0.338725706	0.716000519	1001.242045	-0.124507534	0.933048047	1001.242045	-0.064224247	0.933349818
FBgn0038745	CG4538 gene product from transcript	5459.299219	-0.40522031	0.383220123	5459.299219	-0.678495188	0.027443899	5459.299219	0.533546852	0.080636477
FBgn0002940	neither inactivation nor afterpotential E	2.720300527	0.287599468	0.952412688	2.720300527	0.06654577		2.720300527	-0.214499016	0.831960997
FBgn0038741	CG17186 gene product from transcrip	174.9086079	-0.143314167	0.98775218	174.9086079	-0.253802439	0.863983101	174.9086079	0.369002855	0.590045536
FBgn0038740	CG4562 gene product from transcript	713.8578247	-2.130373197	1.69E-08	713.8578247	-0.569812815	0.490270746	713.8578247	-0.220084171	0.799468289
FBgn0038738	CG4572 gene product from transcript	6005.79459	-0.33316225	0.339396406	6005.79459	-0.415427211	0.142821625	6005.79459	0.37104189	0.139927965
FBgn0038773	CG10887 gene product from transcrip	1.62350033	0.249195116	0.58318903	1.62350033	0		1.62350033	-0.249620796	0.38677458
FBgn0045471	Gustatory receptor 92a	0			0			0		
FBgn0038774	CG5023 gene product from transcript	9291.086906	0.177457455	0.973341033	9291.086906	0.307306173	0.789789955	9291.086906	-1.058828524	0.007770402
FBgn0038775	CG17199 gene product from transcrip	0.368977348	0.196066888		0.368977348	0		0.368977348	-0.196979264	
FBgn0051206	CG31206 gene product from transcrip	2.039086183	0.416106726	0.399353831	2.039086183	0.032546727		2.039086183	-0.417470506	0.252083567
FBgn0003249	Rhodopsin 3	0.174504111	0.073112477		0.174504111	0		0.174504111	0.014071434	
FBgn0038788	CG5085 gene product from transcript	441.7371369	-0.131654374	0.966865338	441.7371369	-0.140987298	0.894405628	441.7371369	0.018246927	0.98040309
FBgn0038789	Ionotropic receptor 92a	0.07379547	0.108851729		0.07379547	0		0.07379547	-0.111773044	
FBgn0038790	Metallothionein C	50.66456605	0.445867174	0.78070356	50.66456605	-0.832341106	0.269874997	50.66456605	-1.265306828	0.010883315
FBgn0038796	CG10881 gene product from transcrip	24.14977707	0.486161169	0.815455832	24.14977707	0.050315912	0.990025502	24.14977707	0.033197513	0.983201238
FBgn0038798	Odorant receptor 92a	0.404324036	0.121123811		0.404324036	0		0.404324036	-0.076265234	
FBgn0038799	CG4288 gene product from transcript	1842.357758	-0.430002722	0.433621973	1842.357758	-0.187213453	0.854243619	1842.357758	0.304023537	0.507731777

FBgn0028684	Regulatory particle triple-A ATPase 5	7713.489029	-0.23003765	0.845956885	7713.489029	-0.363578798	0.498665315	7713.489029	0.492310023
FBgn0039111	PTEN-like phosphatase	167.4912969	-0.165210692	0.97064214	167.4912969	0.109168863	0.948432738	167.4912969	-0.156766006
FBgn0039112	CG10219 gene product from transcript	2552.359981	-0.133930582	0.985645036	2552.359981	0.188822749	0.906537259	2552.359981	-0.600548801
FBgn0028475	CG10221 gene product from transcript	1754.939249	-0.075323808	0.99157593	1754.939249	0.177997358	0.826129098	1754.939249	-0.022891245
FBgn0039110	Ran binding protein 3	2409.047398	-0.100512335	0.994497358	2409.047398	-0.19041579	0.914502763	2409.047398	0.039903956
FBgn0028691	Regulatory particle non-ATPase 9	7465.827463	-0.228775933	0.845362246	7465.827463	-0.317651569	0.597449784	7465.827463	0.471812507
FBgn0039108	CG10232 gene product from transcript	27.30776671	0.354250553	0.910739253	27.30776671	0.249848851	0.909018935	27.30776671	-0.17930758
FBgn0002922	nautilus	19.64260684	-0.229027341	0.977442266	19.64260684	-0.062631022	0.9872372	19.64260684	-0.372140099
FBgn0047351	CG31468 gene product from transcript	3.295286354	0.889603359	0.058579545	3.295286354	0.029469659		3.295286354	-0.926501393
FBgn0043005	portabella	42.32865565	-0.157324935	0.989375441	42.32865565	-0.268922832	0.883421559	42.32865565	-0.108689545
FBgn0039104	CG10252 gene product from transcript	25.33889626	0.371294254	0.419743461	25.33889626	-0.010466159	0.997246866	25.33889626	-0.373755212
FBgn0027512	CG10254 gene product from transcript	706.8590784	0.059120184	0.994497358	706.8590784	0.036691132	0.98637934	706.8590784	-0.071075862
FBgn0051145	CG31145 gene product from transcript	1327.272323	0.133591387	0.949623474	1327.272323	0.023694812	0.988390106	1327.272323	-0.450308982
FBgn0039094	CG10184 gene product from transcript	2466.305336	0.610169581	0.568623357	2466.305336	1.000821419	0.124097763	2466.305336	-1.57904353
FBgn0051143	transfer RNA:CR31143	0.638569864	0.015327852		0.638569864	-0.063752622		0.638569864	-0.085092842
FBgn0039093	CG10183 gene product from transcript	1.35631495	0.187003651		1.35631495	0.205695084		1.35631495	-0.169753362
FBgn0039092	CG16723 gene product from transcript	63.88201932	-1.206719431	0.078254747	63.88201932	-1.765080471	0.001698745	63.88201932	1.674949065
FBgn0053337	CG33337 gene product from transcript	23.55245846	-1.380443668	0.033886908	23.55245846	-1.300118403	0.055303881	23.55245846	1.01015912
FBgn0039091	CG10182 gene product from transcript	1749.075477	-1.55885598	0.004121137	1749.075477	-1.409822229	0.015485563	1749.075477	1.380217022
FBgn0004187	small nuclear RNA U1 at 95Cc	2.857683811	0.406757567	0.818929998	2.857683811	0.337823639		2.857683811	-0.191950306
FBgn0004186	small nuclear RNA U1 at 95Cb	0.605699244	-0.208594291		0.605699244	-0.041780053		0.605699244	0
FBgn0051141	CG31141 gene product from transcript	0.664159226	0.216058883		0.664159226	0		0.664159226	-0.216758639
FBgn0039125	Ndc1 ortholog (S. cerevisiae)	483.664342	0.056330802	0.994497358	483.664342	0.213369972	0.741673255	483.664342	0.149785875
FBgn0047114	CG31142 gene product from transcript	185.2422568	-0.081801038	0.994497358	185.2422568	0.169395081	0.917887901	185.2422568	0.066040765
FBgn0039128	CG13599 gene product from transcript	114.2672268	0.146369219	0.978641661	114.2672268	0.245716421	0.831801071	114.2672268	0.02633786
FBgn0039130	CG5854 gene product from transcript	3626.438583	-0.006418096	0.998322595	3626.438583	-0.286187973	0.737092825	3626.438583	0.493984681
FBgn0039132	Adaptor Protein complex 1, sigma subu	1006.601798	-0.168785247	0.921015845	1006.601798	0.069455754	0.95758415	1006.601798	0.118563009
FBgn0051140	CG31140 gene product from transcript	795.5722777	0.157127328	0.953941411	795.5722777	0.217571827	0.792524256	795.5722777	-0.072890671
FBgn0051451	ncRNA	438.0868272	0.417538263	0.764963416	438.0868272	0.156624935	0.939033191	438.0868272	-0.191203487
FBgn0039136	CG5902 gene product from transcript	3216.609277	-0.173118729	0.859393667	3216.609277	-0.192999636	0.737481872	3216.609277	0.30912476
FBgn0015795	CG5915 gene product from transcript	7149.71611	-0.424798828	0.275894383	7149.71611	-0.559328056	0.075198315	7149.71611	0.524855728
FBgn0053108	CG33108 gene product from transcript	94.67149139	-0.241976322	0.936159146	94.67149139	-0.121729092	0.948432738	94.67149139	-0.004657186
FBgn0051184	CG31184 gene product from transcript	929.4385556	0.033264087	0.996983104	929.4385556	-0.114191446	0.940118668	929.4385556	-0.094643876
FBgn0020647	Krueppel target at 95D	1977.283322	0.090709889	0.989856236	1977.283322	-0.246231834	0.759743592	1977.283322	0.203912443
FBgn0039137	CG13604 gene product from transcript	389.7731013	-0.302904642	0.554448103	389.7731013	-0.111643031	0.914502763	389.7731013	0.301913741
FBgn0039135	CG13603 gene product from transcript	2428.923562	-0.230482693	0.518399313	2428.923562	-0.320596752	0.208714782	2428.923562	0.490538853
FBgn0005674	Glutamyl-prolyl-tRNA synthetase	2535.075374	0.256842857	0.786738826	2535.075374	0.38360243	0.455161017	2535.075374	-0.346012198
FBgn0039131	CG12268 gene product from transcript	229.2061157	-0.658038293	0.305215462	229.2061157	-0.417192451	0.652509677	229.2061157	0.004336555
FBgn0039129	Ribosomal protein S19b	19.6223631	0.697769747	0.569342223	19.6223631	0.24979354	0.915954217	19.6223631	-0.737460318
FBgn0015282	Regulatory particle triple-A ATPase 2	10242.24502	-0.133590963	0.971762817	10242.24502	-0.254814944	0.743255708	10242.24502	0.388190707
FBgn0039126	CG13601 gene product from transcript	550.9699218	-0.178163452	0.917248088	550.9699218	0.070198068	0.95914272	550.9699218	-0.080247146
FBgn0016754	six-banded	849.5496507	0.196300047	0.888834551	849.5496507	-0.12124087	0.91558304	849.5496507	0.201856227
FBgn0039124	testis-specifically expressed bromodon	5.450245183	1.134789234	0.043007838	5.450245183	-0.025934783		5.450245183	-1.075411208
FBgn0013347	Transcription-factor-IIA-S	1477.05662	-0.0736418	0.994497358	1477.05662	-0.248250983	0.815455785	1477.05662	0.068911028

[illegible]

FBgn0039467	CG14253 gene product from transcrip	590.8055312	0.250910964	0.859393667	590.8055312	0.06495099	0.971833087	590.8055312
FBgn0039469	TweedleC	4.597287465	0.368175915	0.862079032	4.597287465	0.373727417		4.597287465
FBgn0051077	CG31077 gene product from transcrip	0.667908784	0.099893966		0.667908784	0.039163291		0.667908784
FBgn0039471	CG6295 gene product from transcript	2042.133542	-0.989936963	0.20663283	2042.133542	-0.67078125	0.537391514	2042.133542
FBgn0039470	CG6296 gene product from transcript	3424.843723	-2.327343547	3.07E-07	3424.843723	-2.064778256	1.90E-05	3424.843723
FBgn0051076	CG31076 gene product from transcrip	1367.899151	-0.346760074	0.764963416	1367.899151	-0.646201553	0.226113461	1367.899151
FBgn0051075	CG31075 gene product from transcrip	50879.24387	0.290501135	0.872095585	50879.24387	0.481368458	0.511972821	50879.24387
FBgn0041224	Gustatory receptor 97a	3.169301366	-0.246226125	0.954626622	3.169301366	0.017937597		3.169301366
FBgn0039465	Tetraspanin 97E	876.6625332	-0.128789974	0.975999817	876.6625332	-0.144743182	0.912868368	876.6625332
FBgn0039464	CG6330 gene product from transcript	1587.621473	0.357132639	0.688262792	1587.621473	0.760995593	0.055730956	1587.621473
FBgn0051070	transfer RNA:CR31070	0.221414942	0.066564265		0.221414942	0		0.221414942
FBgn0051071	transfer RNA:CR31071	0			0			0
FBgn0004510	Ets at 97D	415.139665	-0.246441199	0.912494615	415.139665	-0.312727896	0.750344828	415.139665
FBgn0003267	rough	21.75349867	0.147371017	0.993127879	21.75349867	0.304635314	0.873881861	21.75349867
FBgn0026562	CG6378 gene product from transcript	7024.344635	0.258639179	0.939960319	7024.344635	0.211864326	0.899974378	7024.344635
FBgn0027889	ballchen	746.6369942	0.136623112	0.924867928	746.6369942	-0.00498677	0.998730566	746.6369942
FBgn0004842	RYamide receptor	11.58595522	0.98252252	0.231642292	11.58595522	0.605167199	0.627493242	11.58595522
FBgn0029170	TweedleT	88.21735725	0.685221306	0.498035621	88.21735725	0.555315049	0.637636361	88.21735725
FBgn0039479	CG14257 gene product from transcrip	60.03244479	0.71491798	0.330535239	60.03244479	0.25165565	0.878786989	60.03244479
FBgn0039482	CG14258 gene product from transcrip	7463.332059	0.257370537	0.949623474	7463.332059	0.668728274	0.351397063	7463.332059
FBgn0039483	CG14259 gene product from transcrip	33.00591507	0.256072001	0.972330876	33.00591507	0.21911676	0.932579238	33.00591507
FBgn0039485	CG17189 gene product from transcrip	0.570764227	0.240672956		0.570764227	0		0.570764227
FBgn0027574	CG5815 gene product from transcript	647.9405729	-0.146232065	0.954378726	647.9405729	-0.073428537	0.953470108	647.9405729
FBgn0039489	CG5880 gene product from transcript	126.3659378	-0.180887971	0.963043532	126.3659378	-0.154583506	0.915954217	126.3659378
FBgn0039490	CG5882 gene product from transcript	5.083517707	0.134255652	0.99157593	5.083517707	0.10936842		5.083517707
FBgn0029155	Malic enzyme b	3472.787899	0.095976358	0.994497358	3472.787899	0.507549084	0.510233305	3472.787899
FBgn0039494	Gram-positive Specific Serine protease	1490.935478	0.352000003	0.766289956	1490.935478	0.253577381	0.827267758	1490.935478
FBgn0039495	CG5909 gene product from transcript	21.35754867	0.437591387	0.840842244	21.35754867	0.611000292	0.59197336	21.35754867
FBgn0051065	pickpocket 31	1.763725771	0.353629635	0.833424348	1.763725771	0.482253573		1.763725771
FBgn0003482	spindle D	21.09299657	0.746212092	0.468843814	21.09299657	0.82418514	0.364538738	21.09299657
FBgn0051068	CG31068 gene product from transcrip	43.55257532	0.213625194	0.975999817	43.55257532	-0.01070787	0.998964492	43.55257532
FBgn0005696	DNA polymerase alpha 73kD	223.3542981	0.021755849	0.998322595	223.3542981	0.097374147	0.943911457	223.3542981
FBgn0039498	CG17991 gene product from transcrip	3.69935058	0.300556682	0.892718858	3.69935058	0.25119464		3.69935058
FBgn0042111	CG18766 gene product from transcrip	1018.038656	-0.097675262	0.975999817	1018.038656	-0.212435906	0.728212704	1018.038656
FBgn0051064	CG31064 gene product from transcrip	1115.196335	-0.018439738	0.99764662	1115.196335	-0.158883801	0.797865367	1115.196335
FBgn0004197	Serrate	1023.859626	-0.110275592	0.985645036	1023.859626	-0.200203141	0.84787134	1023.859626
FBgn0039492	CG6051 gene product from transcript	2284.96374	-0.0595389	0.9945211	2284.96374	-0.474449267	0.529547258	2284.96374
FBgn0039491	CG6059 gene product from transcript	8.221537363	0.833314415	0.407473733	8.221537363	-0.350418384	0.853093665	8.221537363
FBgn0039488	CG6066 gene product from transcript	353.6154073	-0.08860525	0.988354972	353.6154073	-0.104132612	0.933048047	353.6154073
FBgn0039487	genderblind	638.2965755	0.283333021	0.713985947	638.2965755	-0.008282454	0.997344995	638.2965755
FBgn0039486	CG6074 gene product from transcript	639.2364427	0.131128099	0.993466746	639.2364427	0.48588201	0.674722988	639.2364427
FBgn0039481	Cuticular protein 97Eb	190.1807062	0.636023122	0.443608781	190.1807062	0.782428216	0.245609857	190.1807062
FBgn0039480	Cuticular protein 97Ea	229.1026957	0.55123566	0.411204308	229.1026957	0.806279219	0.092739499	229.1026957
FBgn0003495	spatzle	765.8263748	0.541979743	0.221431479	765.8263748	0.336882191	0.618454741	765.8263748
FBgn0039478	Neprilysin 5	7.685439288	0.242150649	0.975532686	7.685439288	0.039514656	0.993214629	7.685439288
FBgn0039476	CG6271 gene product from transcript	59.13768923	-0.694727363	0.569140102	59.13768923	-0.977030698	0.239171466	59.13768923
FBgn0039475	CG6277 gene product from transcript	1182.391082	-0.733455596	0.508081622	1182.391082	-0.924594639	0.277551005	1182.391082
FBgn0039474	CG6283 gene product from transcript	3455.846245	-0.786068542	0.435744391	3455.846245	-0.735633385	0.480042936	3455.846245
FBgn0039473	CG17191 gene product from transcrip	41.05319938	0.894445084	0.303870338	41.05319938	1.446248759	0.016624333	41.05319938
FBgn0039472	CG17192 gene product from transcrip	3.675333897	-1.165475747	0.043707746	3.675333897	-0.912635949		3.675333897
FBgn0011289	Transcription factor IIA L	4181.918599	0.064164914	0.991146172	4181.918599	-0.190501215	0.73694932	4181.918599
FBgn0039504	CG14260 gene product from transcrip	6.01897951	-0.058867964	0.995682185	6.01897951	0.497449474		6.01897951
FBgn0039505	CG5934 gene product from transcript	384.6112924	-0.177419073	0.967901085	384.6112924	0.129232245	0.939697448	384.6112924
FBgn0046247	CG5938 gene product from transcript	1344.550515	-0.301858757	0.580354456	1344.550515	-0.391799384	0.326265697	1344.550515
FBgn0039508	CG3368 gene product from transcript	887.4910683	-0.050057594	0.994497358	887.4910683	-0.121237858	0.879953149	887.4910683
FBgn0015737	Hemomucin	6523.972826	0.337634995	0.74052988	6523.972826	0.398656602	0.579549857	6523.972826
FBgn0016061	sidestep	225.7282976	0.253218632	0.846035137	225.7282976	0.103976557	0.94426666	225.7282976
FBgn0039511	CG3330 gene product from transcript	4.705435279	0.715692326	0.086659627	4.705435279	0		4.705435279
FBgn0039510	CG3339 gene product from transcript	8.398017562	-0.250504567	0.974005319	8.398017562	-0.179835917	0.946634373	8.398017562
FBgn0040609	CG3348 gene product from transcript	3393.114429	0.164370811	0.982947464	3393.114429	0.146439487	0.944894567	3393.114429
FBgn0039509	CG3350 gene product from transcript	920.1279743	-0.11156103	0.99157593	920.1279743	-0.33008866	0.74945674	920.1279743
FBgn0039507	martik	1281.629308	-0.059696574	0.993127879	1281.629308	-0.078884623	0.936967609	1281.629308
FBgn0002441	lethal (3) malignant brain tumor	1181.254927	0.184493331	0.846500794	1181.254927	-0.021859658	0.989553502	1181.254927
FBgn0039503	CG14262 gene product from transcrip	10.10126438	0.554397184	0.751169575	10.10126438	-0.379934064	0.832797896	10.10126438
FBgn0010328	without children	3595.735129	-0.054000149	0.993127879	3595.735129	-0.095403657	0.906145662	3595.735129
FBgn0010441	pelle	819.0444135	-0.119119063	0.983550368	819.0444135	-0.187671222	0.871207708	819.0444135
FBgn0039501	CG5987 gene product from transcript	189.3775656	1.538236762	0.000409893	189.3775656	1.400175523	0.002654967	189.3775656
FBgn0039500	CG5984 gene product from transcript	67.2233326	0.302085348	0.933260463	67.2233326	0.259365503	0.883802014	67.2233326
FBgn0039518	CG13978 gene product from transcrip	1.74544484	0.344151457	0.526670516	1.74544484	-0.036161423		1.74544484
FBgn0039519	CG13977 gene product from transcrip	438.2670506	1.865057604	0.000160149	438.2670506	2.031019836	3.77E-05	438.2670506
FBgn0039520	Gustatory receptor 98a	3.977975997	-0.284915404	0.945699553	3.977975997	0.079853655		3.977975997
FBgn0039523	CG12885 gene product from transcrip	11.42240251	0.33121545	0.950636559	11.42240251	-0.142125417	0.961378425	11.42240251
FBgn0046887	Gustatory receptor 98b	0.060151179	-0.097832662		0.060151179	-0.093256207		0.060151179
FBgn0046886	Gustatory receptor 98c	0.15702934	0		0.15702934	0		0.15702934
FBgn0046885	Gustatory receptor 98d	0.07379547	0.108851729		0.07379547	0		0.07379547
FBgn0040080	rapsynoid	902.6160189	0.069288643	0.99157593	902.6160189	0.150123311	0.848825646	902.6160189
FBgn0039528	distracted	816.0147642	0.042410882	0.996542676	816.0147642	0.022286155	0.995412313	816.0147642
FBgn0039527	CG5639 gene product from transcript	535.2857509	0.174754401	0.965247527	535.2857509	0.499440323	0.422781784	535.2857509
FBgn0027492	wiederborst	5053.774874	0.154562755	0.975999817	5053.774874	0.305057267	0.770169252	5053.774874
FBgn0039525	CG5646 gene product from transcript	163.6217601	-0.541166754	0.588018622	163.6217601	-0.427262218	0.692875202	163.6217601
FBgn0004387	Kinesin-like protein at 98A	1971.486632	0.184580931	0.950976319	1971.486632	0.025468164	0.991444605	1971.486632
FBgn0039522	CG13972 gene product from transcrip	1.170311736	-0.181812813		1.170311736	-0.033872669		1.170311736
FBgn0039521	CG5402 gene product from transcript	240.7693195	0.374704912	0.840842244	240.7693195	0.43934232	0.684188976	240.7693195
FBgn0005659	Ets at 98B	513.3472167	-0.006212036	0.998322595	513.3472167	0.162983629	0.911006433	513.3472167
FBgn0067407	Odorant receptor 98a pseudogene	0			0			0
FBgn0039551	Odorant receptor 98a	0.376262458	0.125088582		0.376262458	0		0.376262458
FBgn0039552	CG12426 gene product from transcrip	1.252529199	0.230150391		1.252529199	0.011543973		1.252529199
FBgn0039555	mitochondrial ribosomal protein S22	1437.614147	0.019356767	0.998322595	1437.614147	0.188249872	0.895573563	1437.614147
FBgn0003279	Ribosomal protein L4	39393.0172	0.198554114	0.807572364	39393.0172	0.219178018	0.677282764	39393.0172
FBgn0027579	minotaur	2813.226981	-0.38145417	0.410788544	2813.226981	-0.26059861	0.669327837	2813.226981
FBgn0039560	CG5514 gene product from transcript	1127.423664	0.061193192	0.994497358	1127.423664	-0.019564523	0.99460581	1127.423664
FBgn0039562	Glycoprotein 93	20959.02308	0.110004848	0.988216049	20959.02308	0.364507598	0.624517446	20959.02308
FBgn0039564	CG5527 gene product from transcript	138.5143924	0.63238626	0.316901578	138.5143924	0.662499403	0.265714407	138.5143924
FBgn0051054	pseudo	0.112641396	0		0.112641396	0		0.112641396
FBgn0039566	CG4849 gene product from transcript	831.8013565	-0.001656842	0.999629143	831.8013565	0.146014828	0.851566102	831.8013565
FBgn0039568	CG4815 gene product from transcript	0.683037394	0.205795168		0.683037394	0.032521441		0.683037394
FBgn0003890	beta-Tubulin at 97EF	1626.938147	0.18824273	0.961435662	1626.938147	0.170050661	0.910120848	1626.938147
FBgn0051131	CG31131 gene product from transcrip	0			0			0
FBgn0051253	CG31253 gene product from transcrip	0			0			0
FBgn0039565	CG4884 gene product from transcript	0			0			0
FBgn0039563	CG4951 gene product from transcript	373.5335399	-0.181415174	0.924143028	373.5335399	-0.261498406	0.706410494	373.5335399
FBgn0039561	mitoferrin	1267.008702	-0.097709691	0.973453438	1267.008702	0.048608255	0.968777026	1267.008702
FBgn0039559	CG4976 gene product from transcript	1090.667312	0.10434657	0.982498169	1090.667312	0.094196901	0.944289621	1090.667312
FBgn0051053	CG31053 gene product from transcrip	10.77975954	-0.165728015	0.991146172	10.77975954	-0.351618379	0.84759519	10.77975954
FBgn0039558	BCAS2 ortholog	662.4299038	0.180745572	0.921622016	662.4299038	0.007780556	0.997538821	662.4299038
FBgn0039557	CG12259 gene product from transcrip	493.9932865	-0.110241538	0.966157244	493.9932865	-0.042401657	0.973807349	493.9932865
FBgn0053213	CG33213 gene product from transcrip	87.90646867	-0.057349287	0.995120986	87.90646867	0.089141832	0.971833087	87.90646867
FBgn0039554	CG5003 gene product from transcript	593.7726547	0.03836013	0.9945211	593.7726547	0.058754409	0.962628311	593.7726547
FBgn0002865	Male-specific RNA 98Ca	5.243763471	0.561511146	0.278911227	5.243763471	-0.055700516		5.243763471
FBgn0004171	Male-specific RNA 98Cb	3.499654306	0.75976145	0.118417841	3.499654306	0.06746384		3.499654306
FBgn0039576	CG17856 gene product from transcrip	0.368977348	0.196066888		0.368977348	0		0.368977348
FBgn0039577	CG12516 gene product from transcrip	0.442772817	0.202930855		0.442772817	0		0.442772817
FBgn0039581	CG1866 gene product from transcript	2003.895695	-0.109315758	0.946165413	2003.895695	-0.173432127	0.696528331	2003.895695
FBgn0039584	CG14064 gene product from transcrip	105.8079799	0.290765006	0.918245666	105.8079799	0.254612169	0.870577842	105.8079799
FBgn0039585	CG1894 gene product from transcript	9.371100796	0.029045683	0.998322595	9.371100796	0.047459248	0.990025502	9.371100796
FBgn0013813	Dynein heavy chain at 89D	10.03019203	-0.008960412	0.998322595	10.03019203	-0.315968859	0.870965316	10.03019203
FBgn0039588	CG12413 gene product from transcrip	372.6771943	0.019485374	0.998322595	372.6771943	-0.060556267	0.971833087	372.6771943
FBgn0003137	Papilin	3714.424966	0.945100345	0.000826244	3714.424966	0.94263748	0.001138034	3714.424966
FBgn0051051	CG31051 gene product from transcrip	73.27923987	-0.216891005	0.958580291	73.27923987	-0.590617015	0.423277688	73.27923987
FBgn0039580	Glutamine:fructose-6-phosphate amin	2195.447962	0.037427959	0.995584438	2195.447962	-0.009119819	0.997246866	2195.447962
FBgn0001215	Heterogeneous nuclear ribonucleoprot	11015.77757	0.158789942	0.868854885	11015.77757	-0.079778368	0.932075796	11015.77757
FBgn0039589	CG9986 gene product from transcript	293.7874366	-0.303212865	0.7235766	293.7874366	-0.135662232	0.914578467	293.7874366
FBgn0039591	CG9988 gene product from transcript	342.281682	0.462641999	0.624737605	342.281682	0.362404045	0.717244271	342.281682
FBgn0053346	CG33346 gene product from transcrip	668.433484	-1.891492227	2.88E-06	668.433484	-1.950971978	2.42E-06	668.433484
FBgn0039593	CG9989 gene product from transcript	687.5716057	0.261026604	0.953305045	687.5716057	-0.010355446	0.998730566	687.5716057
FBgn0039594	CG9990 gene product from transcript	833.1000882	0.115749583	0.963057925	833.1000882	0.047958456	0.971533442	833.1000882
FBgn0027655	huntingtin	1218.007359	0.37210432	0.407834999	1218.007359	0.387898422	0.355624684	1218.007359
FBgn0039599	intrepid	0			0			0
FBgn0039598	aquarius	0.147619473	0		0.147619473	0		0.147619473
FBgn0039597	CG9997 gene product from transcript	0			0			0
FBgn0039596	CG10000 gene product from transcrip	15.08857701	-0.63223931	0.657834464	15.08857701	-0.684295014	0.551050621	15.08857701
FBgn0039595	Allatostatin A receptor 2	177.6637563	0.42099744	0.438169484	177.6637563	0.437604697	0.401080598	177.6637563
FBgn0051050	CG31050 gene product from transcrip	0.941866333	0.210118031		0.941866333	0		0.941866333
FBgn0039590	CG10011 gene product from transcrip	526.052498	0.043964082	0.995584438	526.052498	-0.255979908	0.805797107	526.052498
FBgn0026400	CG10009 gene product from transcrip	1958.081491	-0.129592656	0.967258404	1958.081491	-0.091929776	0.941360428	1958.081491
FBgn0000659	fork head	1217.454258	0.114727191	0.974005319	1217.454258	0.007521459	0.997522315	1217.454258
FBgn0039600	CG1646 gene product from transcript	2327.856833	0.026351959	0.996983104	2327.856833	-0.010780138	0.997246866	2327.856833
FBgn0039602	CG1647 gene product from transcript	417.4328469	-0.005558479	0.998322595	417.4328469	-0.08213538	0.971833087	417.4328469
FBgn0039609	CG14529 gene product from transcrip	194.8992053	0.306213883	0.750626842	194.8992053	0.028211099	0.990025502	194.8992053
FBgn0039611	CG14528 gene product from transcrip	1167.665508	0.344697182	0.244029996	1167.665508	0.336827814	0.259454048	1167.665508
FBgn0039613	CG14527 gene product from transcrip	577.8383182	0.390973138	0.747413252	577.8383182	0.34270993	0.750344828	577.8383182
FBgn0027578	CG14526 gene product from transcrip	4638.546644	0.317544632	0.732714402	4638.546644	-0.012710992	0.997246866	4638.546644
FBgn0039616	CG11828 gene product from transcrip	1.648814528	0.016395607	0.998322595	1.648814528	0.190534181		1.648814528
FBgn0039623	CG1951 gene product from transcript	482.4073627	-0.034176635	0.995584438	482.4073627	0.136820649	0.910950418	482.4073627
FBgn0041780	Strictosidine synthase-like 2	0.745628545	-0.106714295		0.745628545	-0.094557526		0.745628545
FBgn0039622	CG1442 gene product from transcript	6.088128988	1.018195651	0.178987886	6.088128988	0.396644664		6.088128988
FBgn0039620	waterproof	1140.505187	0.186728788	0.958580291	1140.505187	-0.040001623	0.986877529	1140.505187
FBgn0039617	CG14521 gene product from transcrip	42.01976117	0.274574735	0.949774689	42.01976117	0.14538905	0.948520713	42.01976117
FBgn0053203	CG33203 gene product from transcrip	50.97911708	-0.177900899	0.975999817	50.97911708	-0.300361595	0.824638819	50.97911708
FBgn0039612	CG14523 gene product from transcrip	185.966618	0.573324566	0.137548366	185.966618	0.031702593	0.988368947	185.966618
FBgn0015589	APC-like	965.69853	0.140398941	0.965741401	965.69853	0.158158021	0.876744307	965.69853
FBgn0024273	CG1520 gene product from transcript	1331.440116	-0.375832266	0.344087078	1331.440116	-0.432867559	0.211963045	1331.440116
FBgn0039601	CG1523 gene product from transcript	789.9247183	-0.093761446	0.975532686	789.9247183	-0.176087853	0.771990265	789.9247183
FBgn0005642	wings down	835.6164433	-0.136367105	0.971762817	835.6164433	-0.23562272	0.783255143	835.6164433
FBgn0027873	Cleavage and polyadenylation specificit	659.8514526	-0.087210792	0.981763935	659.8514526	-0.198595706	0.764601941	659.8514526
FBgn0003093	Protein C kinase 98E	1674.093452	0.303516696	0.652274434	1674.093452	0.226159241	0.761838381	1674.093452
FBgn0039627	CG11837 gene product from transcrip	148.8485441	-0.086622606	0.994497358	148.8485441	-0.04603358	0.986232862	148.8485441
FBgn0039628	CG11841 gene product from transcrip	22.03227732	-0.346332892	0.924143028	22.03227732	-0.266762342	0.901802431	22.03227732
FBgn0039629	CG11842 gene product from transcrip	16.99880645	-0.082871043	0.994876798	16.99880645	0.093514743	0.980691994	16.99880645
FBgn0039630	CG11843 gene product from transcrip	0.060151179	-0.097832662		0.060151179	-0.093256207		0.060151179
FBgn0039631	CG11305 gene product from transcrip	271.6468234	-0.095346277	0.989856236	271.6468234	-0.092128094	0.950635663	271.6468234
FBgn0039633	CG11873 gene product from transcrip	1166.615169	0.310272133	0.870991786	1166.615169	0.143478669	0.938654786	1166.615169
FBgn0039635	CG11876 gene product from transcrip	6040.75624	0.225959573	0.909440876	6040.75624	0.365917869	0.613066493	6040.75624
FBgn0039636	Autophagy-specific gene 14	677.292956	-0.247321507	0.758699653	677.292956	-0.319485359	0.537328086	677.292956
FBgn0039637	CG11880 gene product from transcrip	2206.043146	-0.499995128	0.688050284	2206.043146	-1.117743599	0.038384298	2206.043146
FBgn0028671	Vacuolar H[+] ATPase 100kD subunit 1	3735.211195	-0.181586466	0.764963416	3735.211195	-0.134698527	0.821630922	3735.211195
FBgn0039640	CG14516 gene product from transcrip	359.2732171	-0.156586124	0.948432591	359.2732171	0.024736854	0.990025502	359.2732171
FBgn0028692	Regulatory particle non-ATPase 2	16524.2867	-0.125170253	0.961435662	16524.2867	-0.321217046	0.465964027	16524.2867
FBgn0039644	CG11897 gene product from transcrip	622.1827141	-0.262288574	0.822885156	622.1827141	-0.200460122	0.838932786	622.1827141
FBgn0039645	CG11898 gene product from transcrip	64.58242811	-1.074026221	0.135717236	64.58242811	-1.397750424	0.022475628	64.58242811
FBgn0014427	CG11899 gene product from transcrip	2108.621992	-0.178831901	0.972479043	2108.621992	-0.798298803	0.125691939	2108.621992
FBgn0051044	ncRNA	171.3545088	0.303400301	0.886446899	171.3545088	-0.036528725	0.990025502	171.3545088
FBgn0029176	CG11901 gene product from transcrip	48148.20352	0.165783396	0.907444462	48148.20352	0.245729344	0.656953995	48148.20352
FBgn0039654	CG14514 gene product from transcrip	708.4495282	-0.092014857	0.99157593	708.4495282	-0.416834022	0.484683967	708.4495282
FBgn0028476	CG15817 gene product from transcrip	1099.480016	-0.03011212	0.995584438	1099.480016	0.080381337	0.940118668	1099.480016

FBgn0062411	Copper transporter 1C	0.368977348	0.196066888		0.368977348	0		0.368977348
FBgn0039811	CG15550 gene product from transcrip	17.42279933	0.93261574	0.269030267	17.42279933	0.001584531	1	17.42279933
FBgn0039810	CG15549 gene product from transcrip	1.045321365	0.238456412		1.045321365	-0.119341842		1.045321365
FBgn0013973	Guanylyl cyclase beta-subunit at 100B	82.49873044	-0.107735231	0.994497358	82.49873044	-0.12025298	0.95914272	82.49873044
FBgn0004389	bottleneck	6.157509839	0.416977022	0.883797649	6.157509839	0.203186622	0.935512889	6.157509839
FBgn0039827	CG1544 gene product from transcript	1872.676692	0.550850307	0.059845763	1872.676692	0.824284699	0.000550945	1872.676692
FBgn0039828	CG1542 gene product from transcript	709.0402247	-0.035912035	0.994876798	709.0402247	0.016188326	0.994142899	709.0402247
FBgn0039831	CG12054 gene product from transcrip	1697.064674	0.230238065	0.847707545	1697.064674	0.270281218	0.692194725	1697.064674
FBgn0039835	mitochondrial ribosomal protein L32	183.7024707	0.280018237	0.792520678	183.7024707	0.572658052	0.182688349	183.7024707
FBgn0039838	CG3669 gene product from transcript	0.144899044	0		0.144899044	0		0.144899044
FBgn0040628	CG18672 gene product from transcrip	0.245607685	0		0.245607685	0		0.245607685
FBgn0040629	CG18673 gene product from transcrip	0.823531639	0.05282395		0.823531639	-0.031330082		0.823531639
FBgn0039839	pickpocket 24	0.662897269	-0.071396439		0.662897269	-0.147035323		0.662897269
FBgn0039840	CG11340 gene product from transcrip	216.5827171	-1.068596904	0.002129887	216.5827171	-0.700451273	0.124097763	216.5827171
FBgn0039836	CG1750 gene product from transcript	62.11408429	0.419346479	0.72893315	62.11408429	0.520493697	0.523007999	62.11408429
FBgn0051005	CG31005 gene product from transcrip	189.9290062	-0.140104831	0.979231897	189.9290062	0.190995365	0.89758168	189.9290062
FBgn0039833	CG15564 gene product from transcrip	1.065113248	0.24766135		1.065113248	-0.034032292		1.065113248
FBgn0039832	CG15563 gene product from transcrip	0.888780595	0.058882239		0.888780595	0.244233892		0.888780595
FBgn0039830	CG1746 gene product from transcript	38241.93543	0.465554222	0.650039453	38241.93543	0.79502577	0.153446131	38241.93543
FBgn0046696	Ribonuclease P RNA	272.6601873	0.24164	0.886446899	272.6601873	0.098918629	0.949821247	272.6601873
FBgn0039829	CG15561 gene product from transcrip	226.2227685	-0.149671276	0.978641661	226.2227685	-0.287437232	0.794514253	226.2227685
FBgn0061476	CG18729 gene product from transcrip	203.4039171	-0.013272911	0.998322595	203.4039171	0.025835142	0.990025502	203.4039171
FBgn0051004	CG31004 gene product from transcrip	2395.284279	-0.714829654	0.206246096	2395.284279	-0.786770601	0.131972029	2395.284279
FBgn0046332	gasket	4.227195664	0.88289118	0.132496023	4.227195664	-0.013101009		4.227195664
FBgn0039844	CG1607 gene product from transcript	2935.32044	-0.14339184	0.979231897	2935.32044	-0.359408969	0.684745433	2935.32044
FBgn0019990	CG1609 gene product from transcript	849.5114441	0.136896697	0.960742862	849.5114441	0.093780466	0.937166646	849.5114441
FBgn0039850	CG11333 gene product from transcrip	10.12365223	1.213137202	0.092659882	10.12365223	0.873316838	0.347242268	10.12365223
FBgn0039851	morpheyus	184.5098642	-0.31898088	0.903108364	184.5098642	-0.484790149	0.645558524	184.5098642
FBgn0039852	nyobe	301.1331679	-0.086593716	0.994497358	301.1331679	-0.200251186	0.884956001	301.1331679
FBgn0028968	Coat Protein (coatomer) gamma	3806.154942	0.235669411	0.797866853	3806.154942	0.329376213	0.522024427	3806.154942
FBgn0003268	rough deal	474.2252002	0.106268136	0.979231897	474.2252002	0.006158994	0.998730566	474.2252002
FBgn0039854	CG1635 gene product from transcript	57.11154739	0.024349571	0.998322595	57.11154739	0.170389346	0.937682657	57.11154739
FBgn0039855	CG1638 gene product from transcript	8.580400712	0.46097259	0.851178593	8.580400712	0.232307064	0.922935459	8.580400712
FBgn0039856	CG1774 gene product from transcript	432.6440292	0.427226809	0.815249619	432.6440292	0.457750859	0.699222128	432.6440292
FBgn0039858	Cyclin G	27102.96061	0.174248776	0.953941411	27102.96061	0.321775283	0.651795237	27102.96061
FBgn0039857	Ribosomal protein L6	26878.78314	0.262864787	0.476136719	26878.78314	0.112738916	0.868380046	26878.78314
FBgn0043900	pygopus	727.1748238	0.128001184	0.966157244	727.1748238	0.235985665	0.728212704	727.1748238
FBgn0039849	CG11334 gene product from transcrip	3626.716752	-0.023114314	0.997459641	3626.716752	-0.055482048	0.96950421	3626.716752
FBgn0039848	lysyl oxidase-like	13.40975626	0.30788804	0.956325514	13.40975626	0.190492847	0.942409981	13.40975626
FBgn0039846	CG11337 gene product from transcrip	387.2521595	0.043051726	0.994497358	387.2521595	0.090267749	0.939574224	387.2521595
FBgn0051002	CG31002 gene product from transcrip	2.057899212	-0.157395701	0.979231897	2.057899212	0.094086656		2.057899212
FBgn0051204	CG31204 gene product from transcrip	2.371383783	0.480433388	0.497950998	2.371383783	0.032194257		2.371383783
FBgn0039860	CG1792 gene product from transcript	190.5546755	0.046358134	0.994876798	190.5546755	-0.06360585	0.971833087	190.5546755
FBgn0039861	partner of drosha	562.286919	-0.04953628	0.994497358	562.286919	-0.045172472	0.971833087	562.286919
FBgn0039862	CG1804 gene product from transcript	179.5804029	-0.164180281	0.965076657	179.5804029	-0.056686154	0.979303552	179.5804029
FBgn0039863	CG1815 gene product from transcript	1172.055835	-0.222977768	0.851249857	1172.055835	-0.051231453	0.974682104	1172.055835
FBgn0003870	tramtrack	1758.8928	0.022879237	0.998322595	1758.8928	0.053880053	0.973807349	1758.8928
FBgn0039868	CG11563 gene product from transcrip	383.0832459	-0.025105132	0.996983104	383.0832459	0.137038471	0.879348054	383.0832459
FBgn0000557	Elongation factor 1alpha100E	3271.272541	-0.257257414	0.813832472	3271.272541	-0.387830518	0.479376545	3271.272541
FBgn0039869	CG1890 gene product from transcript	1467.995078	-0.20815677	0.765575472	1467.995078	-0.143438009	0.840932206	1467.995078
FBgn0039870	CG1896 gene product from transcript	695.4976715	-0.390831527	0.416434118	695.4976715	-0.06118776	0.967970176	695.4976715
FBgn0022349	CG1910 gene product from transcript	4704.465361	0.06155737	0.994497358	4704.465361	0.070247028	0.951701663	4704.465361
FBgn0039875	septin interacting protein 3	1771.67847	0.039431627	0.994497358	1771.67847	0.148487073	0.78038886	1771.67847
FBgn0005632	fat facets	4522.215941	-0.03490414	0.995584438	4522.215941	0.083140055	0.950970297	4522.215941

[illegible]

FBgn0027535	brother of tout-velu	598.0430745	-0.082926188	0.988354972	598.0430745	0.107623294	0.919333418	598.0430745
FBgn0034419	CG15111 gene product from transcrip	933.6980584	-0.0122325	0.998322595	933.6980584	-0.110363557	0.915954217	933.6980584
FBgn0034418	CG15118 gene product from transcrip	2444.362243	-0.342391474	0.495571928	2444.362243	-0.361808623	0.436545737	2444.362243
FBgn0050125	Ionotropic receptor 56a	0.759551347	0.054465033		0.759551347	0.042491132		0.759551347
FBgn0034413	CG15115 gene product from transcrip	23.09487126	-0.321694053	0.944197659	23.09487126	0.27007813	0.896716593	23.09487126
FBgn0041702	CG15107 gene product from transcrip	211.0135026	0.316690974	0.646532102	211.0135026	-0.14127601	0.904226848	211.0135026
FBgn0014269	proliferation disrupter	934.0418218	-0.137048618	0.938791798	934.0418218	-0.096115439	0.918067312	934.0418218
FBgn0028669	Vacuolar H[+] ATPase 100kD subunit 3	2.268394044	0.463078843	0.458324575	2.268394044	-0.067932219		2.268394044
FBgn0050227	transfer RNA:CR30227	0.100708642	0		0.100708642	0		0.100708642
FBgn0034377	CG15071 gene product from transcrip	2.096519952	0.224609567	0.963501097	2.096519952	0.339672857		2.096519952
FBgn0034379	CG15073 gene product from transcrip	187.7265955	-0.028355726	0.998150112	187.7265955	-0.234473992	0.823463209	187.7265955
FBgn0053147	Heparan sulfate 3-O sulfotransferase-A	109.1527728	-0.032702871	0.998322595	109.1527728	0.197115095	0.911006433	109.1527728
FBgn0034387	CG15077 gene product from transcrip	278.8913595	-0.288801184	0.68934315	278.8913595	0.118259924	0.915954217	278.8913595
FBgn0050229	transfer RNA:CR30229	0.056320698	0		0.056320698	0		0.056320698
FBgn0034391	CG15080 gene product from transcrip	15.8584289	0.259360355	0.969626359	15.8584289	0.318334942	0.870933294	15.8584289
FBgn0010551	Prohibitin 2	4281.963873	0.029746124	0.997450287	4281.963873	0.387988398	0.558960203	4281.963873
FBgn0034397	CG15082 gene product from transcrip	21.9665393	-0.357274058	0.908816464	21.9665393	-0.552394712	0.64616911	21.9665393
FBgn0034399	CG15083 gene product from transcrip	1361.145862	0.102590606	0.988216049	1361.145862	0.435147127	0.436418979	1361.145862
FBgn0034402	CG15084 gene product from transcrip	103.5082875	-0.226106611	0.934448847	103.5082875	-0.367079435	0.666545328	103.5082875
FBgn0004240	Diptericin	2744.732957	1.00335678	0.188886872	2744.732957	0.212700282	0.930935455	2744.732957
FBgn0034407	Diptericin B	986.3565888	1.018780949	0.188962069	986.3565888	0.487863638	0.736007486	986.3565888
FBgn0034406	Juvenile hormone epoxide hydrolase 3	3593.924139	-0.506363645	0.367097244	3593.924139	-0.613820468	0.183730674	3593.924139
FBgn0010053	Juvenile hormone epoxide hydrolase 1	1338.464923	-0.119927425	0.967901085	1338.464923	0.017684514	0.993214629	1338.464923
FBgn0034403	CG18190 gene product from transcrip	177.6139024	-0.154836567	0.985645036	177.6139024	-0.298839372	0.834775152	177.6139024
FBgn0034401	CG15100 gene product from transcrip	1283.951093	0.102641792	0.975999817	1283.951093	0.239580216	0.673945053	1283.951093
FBgn0034400	CG15099 gene product from transcrip	2030.895846	0.003990121	0.998322595	2030.895846	-0.232599382	0.816067304	2030.895846
FBgn0034398	CG15098 gene product from transcrip	20616.52179	-0.347078785	0.509274411	20616.52179	-0.51144993	0.15337121	20616.52179
FBgn0034396	CG15097 gene product from transcrip	430.6748783	0.045020866	0.997459641	430.6748783	-0.814603591	0.277551005	430.6748783
FBgn0034394	CG15096 gene product from transcrip	589.4151289	-0.500763521	0.556956355	589.4151289	-0.666115798	0.277551005	589.4151289
FBgn0010651	lethal (2) 08717	4136.845718	-0.526181242	0.169926019	4136.845718	-0.439895961	0.321040925	4136.845718
FBgn0034392	Major Facilitator Superfamily Transport	825.9594332	-0.250496621	0.819283673	825.9594332	-0.101587718	0.939574224	825.9594332
FBgn0034390	CG15093 gene product from transcrip	337.959264	0.576601867	0.623344971	337.959264	0.724967844	0.38976091	337.959264
FBgn0034383	CG17821 gene product from transcrip	0.221386409	0.173816599		0.221386409	0		0.221386409
FBgn0034382	CG18609 gene product from transcrip	15.99678386	0.519295085	0.784648266	15.99678386	0.602879569	0.639506025	15.99678386
FBgn0050121	ncRNA	0.130116168	0.082464995		0.130116168	0		0.130116168
FBgn0034381	Lithium-inducible SLC6 transporter	74.89042134	0.232271321	0.961435662	74.89042134	0.023924346	0.995503607	74.89042134
FBgn0034380	CG15087 gene product from transcrip	436.7215307	-0.36183254	0.353576216	436.7215307	-0.25337461	0.632657555	436.7215307
FBgn0023214	ETS-domain lacking	333.5401323	0.091292944	0.991146172	333.5401323	-0.420265467	0.457843388	333.5401323
FBgn0053136	CG33136 gene product from transcrip	2.348017116	0.385322458	0.7987068	2.348017116	-0.076076224		2.348017116
FBgn0034372	GDI interacting protein 3	0			0			0
FBgn0034371	CG5473 gene product from transcript	989.854962	-0.262313547	0.719948026	989.854962	-0.073502967	0.951701663	989.854962
FBgn0011868	transfer RNA:gly3:55E	0			0			0
FBgn0050122	CG30122 gene product from transcrip	5896.511294	0.202988685	0.826430371	5896.511294	0.157597241	0.836520481	5896.511294
FBgn0034368	CG5482 gene product from transcript	3460.71248	-0.036950129	0.995120986	3460.71248	0.12830461	0.914004486	3460.71248

[illegible]

FBgn0030108	Gustatory receptor 8a	0.755442356	0.013712941		0.755442356	-0.02761527		0.755442356
FBgn0010269	Downstream of raf1	1931.063978	-0.113821171	0.978233016	1931.063978	-0.22240718	0.780244249	1931.063978
FBgn0030114	CG17754 gene product from transcrip	3562.389066	-0.525582765	0.526670516	3562.389066	-0.983700418	0.040003082	3562.389066
FBgn0028292	CG15797 gene product from transcrip	523.7625155	-0.178013069	0.942692295	523.7625155	-0.111104654	0.933048047	523.7625155
FBgn0030122	CG16892 gene product from transcrip	749.0238454	-0.172613446	0.971711431	749.0238454	0.079120199	0.971833087	749.0238454
FBgn0002576	lozenge	124.6317465	0.338242127	0.755584388	124.6317465	0.063623423	0.977204596	124.6317465
FBgn0040234	CG12149 gene product from transcrip	680.1050276	0.33559014	0.753828842	680.1050276	0.260500013	0.798530085	680.1050276
FBgn0040235	CG12135 gene product from transcrip	659.5760837	-0.10980732	0.979231897	659.5760837	-0.095304377	0.94426666	659.5760837
FBgn0040236	CG12132 gene product from transcrip	2111.334329	-0.161530946	0.963501097	2111.334329	-0.119418562	0.933985422	2111.334329
FBgn0030121	CG17446 gene product from transcrip	388.1243892	0.058239208	0.994497358	388.1243892	-0.067345725	0.96327181	388.1243892
FBgn0030120	CG17440 gene product from transcrip	14.59961335	0.102207127	0.994497358	14.59961335	0.084088264	0.984041268	14.59961335
FBgn0052703	Extracellularly regulated kinase 7	5.757642891	1.060077507	0.018249065	5.757642891	0.043515137		5.757642891
FBgn0030116	pseudo	1.367164378	0.271984378		1.367164378	0		1.367164378
FBgn0000077	almondex	251.9826304	-0.195291584	0.949774689	251.9826304	-0.349567698	0.651045377	251.9826304
FBgn0052704	Ionotropic receptor 8a	0.38631066	0		0.38631066	0		0.38631066
FBgn0030109	CG12121 gene product from transcrip	1378.714961	0.069157803	0.988354972	1378.714961	0.02901942	0.985435073	1378.714961
FBgn0030102	CG12119 gene product from transcrip	2875.207479	0.107356288	0.987910537	2875.207479	-0.146005501	0.91558304	2875.207479
FBgn0030101	CG12118 gene product from transcrip	345.4194508	-0.181472872	0.934448847	345.4194508	-0.055857934	0.973807349	345.4194508
FBgn0030097	CG12115 gene product from transcrip	3523.242794	-0.738583295	0.039828605	3523.242794	-0.964527558	0.002165331	3523.242794
FBgn0030096	CG9060 gene product from transcript	591.1028971	0.012799465	0.998322595	591.1028971	0.076828081	0.961378425	591.1028971
FBgn0040931	CG9034 gene product from transcript	2166.105939	-0.084988771	0.994497358	2166.105939	0.076349437	0.980691994	2166.105939
FBgn0014868	Oligosaccharyltransferase 48kD subun	8207.523442	0.274032103	0.739002922	8207.523442	0.317439222	0.592565499	8207.523442
FBgn0030092	frataxin homolog	209.8379086	-0.242163749	0.886446899	209.8379086	-0.256274938	0.788893834	209.8379086
FBgn0030090	forked end	459.3019081	0.739367883	0.308402171	459.3019081	0.885077329	0.149581297	459.3019081
FBgn0004856	CG8264 gene product from transcript	2376.608764	-0.045085112	0.9945211	2376.608764	-0.188309578	0.830928836	2376.608764
FBgn0030087	CG7766 gene product from transcript	3662.395409	0.306782175	0.755584388	3662.395409	0.59558031	0.179847443	3662.395409
FBgn0030081	CG7246 gene product from transcript	315.8632714	0.012697833	0.998322595	315.8632714	-0.203271002	0.79038145	315.8632714
FBgn0030078	CG10970 gene product from transcrip	3.117586464	0.184583943	0.979231897	3.117586464	-0.096539718		3.117586464
FBgn0030077	CG15365 gene product from transcrip	426.9172102	0.068142076	0.989856236	426.9172102	0.081614341	0.936375202	426.9172102
FBgn0026149	CG17252 gene product from transcrip	728.0777447	0.028369127	0.995584438	728.0777447	-0.000708307	1	728.0777447
FBgn0030136	Ribosomal protein S28b	52673.92165	0.007627383	0.998322595	52673.92165	-0.18614347	0.850323429	52673.92165
FBgn0001186	Hexokinase A	4268.628191	0.465715078	0.548176313	4268.628191	0.389877596	0.645025488	4268.628191
FBgn0030141	Golgi-localized, gamma-adaptin ear cor	1610.986885	0.000498005	0.999781966	1610.986885	-0.148943955	0.828254838	1610.986885
FBgn0027084	Lysyl-tRNA synthetase	3217.841781	0.142137319	0.901924308	3217.841781	0.239924466	0.572501461	3217.841781
FBgn0052699	CG32699 gene product from transcrip	1375.71359	0.061276328	0.994497358	1375.71359	0.138463867	0.886539694	1375.71359
FBgn0052702	Cubilin ortholog	1215.449472	-0.284546771	0.882857781	1215.449472	0.151530275	0.927022063	1215.449472
FBgn0030137	CG15317 gene product from transcrip	78.59230065	-0.106497109	0.99157593	78.59230065	-0.140129635	0.933048047	78.59230065
FBgn0028327	lethal (1) G0320	17005.77573	0.453791378	0.225883877	17005.77573	0.67474213	0.017052116	17005.77573
FBgn0000233	buttonhead	119.694974	-0.039040242	0.995584438	119.694974	-0.324508267	0.709337076	119.694974
FBgn0020378	CG1343 gene product from transcript	918.8812765	0.175201227	0.856136229	918.8812765	-0.020133886	0.990025502	918.8812765
FBgn0052698	CG32698 gene product from transcrip	5143.963765	0.215968484	0.736375381	5143.963765	-0.021856366	0.988639252	5143.963765
FBgn0026415	Imaginal disc growth factor 4	21873.52842	0.502342968	0.022562863	21873.52842	0.524790807	0.015545949	21873.52842
FBgn0030148	CG3106 gene product from transcript	397.5483438	-1.806084106	0.000391715	397.5483438	-0.864754029	0.292131032	397.5483438
FBgn0030164	CG1889 gene product from transcript	0.254386337	0		0.254386337	0.136980718		0.254386337
FBgn0052694	CG32694 gene product from transcrip	682.5920494	0.755149324	0.416369183	682.5920494	1.13591197	0.080766617	682.5920494
FBgn0030170	CG2990 gene product from transcript	70.72412888	0.014598658	0.998322595	70.72412888	0.455196472	0.700947822	70.72412888
FBgn0004045	Yolk protein 1	2.864503328	0.155868844	0.988216049	2.864503328	0.53324893		2.864503328
FBgn0052693	Gustatory receptor 9a	0			0			0
FBgn0030174	CG15312 gene product from transcrip	95.10095515	-0.099947984	0.993466746	95.10095515	-0.056361857	0.984041268	95.10095515
FBgn0005391	Yolk protein 2	45.37753399	0.523427066	0.733719175	45.37753399	0.888476269	0.250111665	45.37753399
FBgn0030167	CG15252 gene product from transcrip	2.900488936	0.33492431	0.895960801	2.900488936	0.225420807		2.900488936
FBgn0030163	CG1791 gene product from transcript	0.241472153	0.06444487		0.241472153	0.110367284		0.241472153
FBgn0030177	CG2972 gene product from transcript	793.3863141	-0.116291011	0.975999817	793.3863141	-0.145547484	0.888777857	793.3863141
FBgn0030181	CG12645 gene product from transcrip	0.896825585	0.042752944		0.896825585	0.065184547		0.896825585
FBgn0052690	ncRNA	5.498651806	0.849479613	0.218094263	5.498651806	0.030708442		5.498651806
FBgn0028342	lethal (1) G0230	5112.214347	-0.003703747	0.998873611	5112.214347	0.382969228	0.705388028	5112.214347
FBgn0030189	CG2909 gene product from transcript	289.113613	-0.148652907	0.959208615	289.113613	-0.457302726	0.30423522	289.113613
FBgn0028480	CG17841 gene product from transcrip	1682.50791	-0.194880409	0.83696011	1682.50791	-0.052162429	0.968777026	1682.50791
FBgn0030195	CG2898 gene product from transcript	7.884933366	0.442260797	0.862079032	7.884933366	0.119992541	0.971533442	7.884933366
FBgn0000711	flapwing	1287.4546	0.100337845	0.988354972	1287.4546	0.321125874	0.651045377	1287.4546
FBgn0030200	CG2885 gene product from transcript	0.099032819	0		0.099032819	0.123176119		0.099032819
FBgn0030206	CG2889 gene product from transcript	303.5638059	-0.052159574	0.994497358	303.5638059	0.064356468	0.968777026	303.5638059
FBgn0030208	Protein phosphatase 4 regulatory subu	5388.13988	-0.190923331	0.793978838	5388.13988	-0.126208365	0.861074999	5388.13988
[illegible]	[illegible]	[illegible]	[illegible]	[illegible]	[illegible]	[illegible]	[illegible]	[illegible]

FBgn0046999	CG6429 gene product from transcript	9.727865564	-0.027665442	0.998322595	9.727865564	-0.708615793	0.520085824	9.727865564
FBgn0034166	CG6472 gene product from transcript	11.45312626	-0.304811121	0.954886241	11.45312626	0.132500472	0.964908204	11.45312626
FBgn0004784	inactivation no afterpotential C	20.66072239	-0.121251566	0.994497358	20.66072239	0.006225663	1	20.66072239
FBgn0003091	Protein C kinase 53E	172.7456324	0.687131529	0.350031487	172.7456324	0.393533696	0.735301845	172.7456324
FBgn0034168	CG15614 gene product from transcript	1.827937504	-0.012540532	0.998322595	1.827937504	0.163286817		1.827937504
FBgn0034172	CG6665 gene product from transcript	639.9132161	-0.051675325	0.994497358	639.9132161	-0.394992372	0.444619778	639.9132161
FBgn0011867	transfer RNA:gly3:53E	0			0			0
FBgn0004580	Calbindin 53E	230.362194	-0.21645517	0.949623474	230.362194	-0.104852488	0.951777831	230.362194
FBgn0034177	CG6796 gene product from transcript	116.8644605	-0.2143243	0.963501097	116.8644605	-0.04630857	0.9872372	116.8644605
FBgn0026402	Nuclear inhibitor of Protein phosphata	295.9451116	-0.145072069	0.974005319	295.9451116	-0.065743782	0.97230334	295.9451116
FBgn0034176	prenyl protease type I	1604.563187	-0.017168948	0.998322595	1604.563187	0.147702425	0.851566102	1604.563187
FBgn0034175	ste24b prenyl protease type I	20.86353576	-0.258017695	0.971565969	20.86353576	0.051486358	0.990025502	20.86353576
FBgn0034173	CG9010 gene product from transcript	1.080908486	0.218765076		1.080908486	0.013913114		1.080908486
FBgn0025833	CG8910 gene product from transcript	54.48606774	-0.19347132	0.975999817	54.48606774	-0.797535762	0.27078092	54.48606774
FBgn0014870	P-element somatic inhibitor	2946.514824	0.145817358	0.92740957	2946.514824	0.047653023	0.971833087	2946.514824
FBgn0025832	Flap endonuclease 1	421.402751	0.02835889	0.99764662	421.402751	0.047561865	0.984079364	421.402751
FBgn0026754	UDP-glycosyltransferase 37c1	42.56040138	1.993291209	0.000175148	42.56040138	2.284297689	1.23E-05	42.56040138
FBgn0034159	CG15615 gene product from transcrip	173.4106618	0.16189653	0.991146172	173.4106618	0.169028852	0.948432738	173.4106618
FBgn0010213	Superoxide dismutase 2 (Mn)	2383.769702	0.334818587	0.769876747	2383.769702	0.467994135	0.485692146	2383.769702
FBgn0015584	Accessory gland protein 53Ea	0.139419282	-0.122978845		0.139419282	-0.119403538		0.139419282
FBgn0034153	Accessory gland protein 53C14b	0.12877022	0		0.12877022	0		0.12877022
FBgn0034152	Accessory gland protein 53C14a	0			0			0
FBgn0034151	CG15617 gene product from transcrip	0.518843275	0		0.518843275	0.109304163		0.518843275
FBgn0053458	CG33458 gene product from transcrip	26.33714568	0.112532394	0.994497358	26.33714568	-0.243021889	0.918961524	26.33714568
FBgn0034132	Sperm-Leucylaminopeptidase 8	15.60974608	0.373843509	0.398005839	15.60974608	0	1	15.60974608
FBgn0011260	CG4700 gene product from transcript	1577.888892	-0.353454062	0.580861838	1577.888892	-0.125563399	0.917910585	1577.888892
FBgn0034135	Syntrophin-like 2	38.37160194	-0.423524074	0.842650983	38.37160194	-0.658284118	0.516257105	38.37160194
FBgn0034137	CG4945 gene product from transcript	191.2989894	-0.127319973	0.979231897	191.2989894	0.196675748	0.862031847	191.2989894
FBgn0016131	Cyclin-dependent kinase 4	617.1754902	0.029355419	0.998322595	617.1754902	0.176230441	0.901802431	617.1754902
FBgn0003274	Ribosomal protein LP2	103342.72	-0.149278304	0.988851516	103342.72	-0.272057494	0.869020803	103342.72
FBgn0034144	CG5089 gene product from transcript	11.0704508	1.186167778	0.008439781	11.0704508	0.107060335	0.960705837	11.0704508
FBgn0034145	CG5065 gene product from transcript	337.9044398	0.281764217	0.886446899	337.9044398	0.202907668	0.886645978	337.9044398
FBgn0024232	CG18471 gene product from transcrip	109.3877966	-0.015021335	0.998322595	109.3877966	0.339716539	0.658774601	109.3877966
FBgn0013763	CG5210 gene product from transcript	21936.03229	0.565990175	0.109693554	21936.03229	0.613913225	0.069034959	21936.03229
FBgn0050463	CG30463 gene product from transcrip	2934.037498	0.081985345	0.985645036	2934.037498	-0.123952198	0.885373767	2934.037498
FBgn0024315	CG8098 gene product from transcript	3154.653725	-0.19822487	0.958499814	3154.653725	-0.392087574	0.64241103	3154.653725
FBgn0024319	CG8178 gene product from transcript	2.122332291	-0.379711197	0.784023628	2.122332291	-0.203525465		2.122332291
FBgn0020506	CG8221 gene product from transcript	1745.146776	-0.803194526	0.242862418	1745.146776	-0.37560661	0.772058606	1745.146776
FBgn0040505	CG8250 gene product from transcript	734.2801908	0.372076129	0.728029857	734.2801908	0.385197418	0.645558524	734.2801908
FBgn0034143	CG8303 gene product from transcript	267.2178349	0.640320443	0.386851348	267.2178349	0.792456965	0.185133756	267.2178349
FBgn0034142	CG8306 gene product from transcript	2207.274421	0.010494752	0.998322595	2207.274421	0.24838412	0.703789098	2207.274421
FBgn0034141	CG8311 gene product from transcript	770.7408481	-0.374702673	0.573694932	770.7408481	-0.152540562	0.901802431	770.7408481
FBgn0034140	CG8317 gene product from transcript	3010.128006	-0.354993281	0.504383899	3010.128006	-0.506501326	0.176009909	3010.128006
FBgn0034138	Ribosomal protein S15	32202.86665	-0.005723367	0.998322595	32202.86665	-0.0438893	0.98613036	32202.86665
FBgn0034136	Dopamine transporter	26.3116959	0.047969202	0.997459641	26.3116959	-0.17414412	0.944894567	26.3116959
FBgn0034133	CG7813 gene product from transcript	5.297838775	0.826757927	0.091368185	5.297838775	-0.07126046		5.297838775
FBgn0034131	CG15712 gene product from transcrip	0.060151179	-0.097832662		0.060151179	-0.093256207		0.060151179
FBgn0050239	transfer RNA:CR30239	1.779694211	0.166396775	0.975999817	1.779694211	0.013115044		1.779694211
FBgn0050240	transfer RNA:CR30240	0.349349285	0.157015978		0.349349285	0		0.349349285
FBgn0034094	Transferrin 3	573.635316	-0.022972246	0.99764662	573.635316	0.226731181	0.750344828	573.635316
FBgn0034095	CG15701 gene product from transcrip	119.1447983	-0.34739681	0.817019193	119.1447983	-0.405081211	0.661343871	119.1447983
FBgn0034097	CG3687 gene product from transcript	0.590363756	0.212547667		0.590363756	0		0.590363756
FBgn0053017	CG33017 gene product from transcrip	1.047699013	0.253737978		1.047699013	0.061851465		1.047699013
FBgn0034103	CG15704 gene product from transcrip	38.54302977	0.536827612	0.693008237	38.54302977	0.145581195	0.951701663	38.54302977
FBgn0034104	CG15705 gene product from transcrip	0.586864518	0.225538349		0.586864518	0		0.586864518
FBgn0034106	CG9068 gene product from transcript	0.147619473	0		0.147619473	0		0.147619473
FBgn0028424	Juvenile hormone-inducible protein 26	205.1319983	-0.407001799	0.845308204	205.1319983	-0.399529865	0.775844637	205.1319983
FBgn0050100	CG30100 gene product from transcrip	0			0			0
FBgn0034110	Autophagy-specific gene 9	2337.534346	0.074484758	0.994497358	2337.534346	0.03702551	0.986877529	2337.534346
FBgn0034114	CG4282 gene product from transcript	343.1798191	-0.183907215	0.900180886	343.1798191	-0.309807637	0.573994985	343.1798191
FBgn0010611	HMG Coenzyme A synthase	1579.272505	-0.297472189	0.737534252	1579.272505	-0.276369747	0.708422272	1579.272505
FBgn0034118	Nucleoporin 62kD	956.8217127	-0.102131985	0.978169713	956.8217127	-0.293208079	0.586395408	956.8217127
FBgn0034126	CG4398 gene product from transcript	425.8403676	-0.582965263	0.079799667	425.8403676	-0.921368202	0.00059333	425.8403676

FBgn0033812	Peroxin 13	2502.202255	-0.260387755	0.569140102	2502.202255	-0.119939815	0.873100502	2502.202255
FBgn0043010	CG4643 gene product from transcript	776.4066165	-0.237022131	0.859393667	776.4066165	-0.245076649	0.771825396	776.4066165
FBgn0050058	CG30058 gene product from transcrip	0			0			0
FBgn0005654	latheo	0			0			0
FBgn0053182	Histone demethylase 4B	81.96676811	0.017652308	0.998322595	81.96676811	0.075314026	0.978624719	81.96676811
FBgn0033802	CG17724 gene product from transcrip	70.37810891	-0.007665635	0.998322595	70.37810891	0.364359095	0.797528394	70.37810891
FBgn0033799	Glial Lazarillo	130.4198206	-0.374113417	0.720774982	130.4198206	-0.159433363	0.915954217	130.4198206
FBgn0033798	CG15870 gene product from transcrip	275.632975	0.504130229	0.694459829	275.632975	-0.122516354	0.959800155	275.632975
FBgn0020370	tripeptidyl-peptidase II	6092.736921	-0.11396754	0.965741401	6092.736921	-0.304572821	0.479376545	6092.736921
FBgn0033794	CG13326 gene product from transcrip	2.115708652	0.30937922	0.814456364	2.115708652	-0.003870011		2.115708652
FBgn0033793	CG3955 gene product from transcript	63.00122644	-0.483524835	0.73499901	63.00122644	-0.611832145	0.522083033	63.00122644
FBgn0028963	Odorant receptor 49b	1.588964227	0.158159184		1.588964227	0.302676153		1.588964227
FBgn0000181	bicaudal	23069.6723	0.211459778	0.835962233	23069.6723	0.205592919	0.773294804	23069.6723
FBgn0002069	Aspartyl-tRNA synthetase	1420.34729	-0.087041026	0.994497358	1420.34729	-0.134637342	0.932579238	1420.34729
FBgn0003975	vestigial	333.3928054	0.001169186	0.999629143	333.3928054	0.044071439	0.980691994	333.3928054
FBgn0033782	sugarbabe	922.2453217	0.213435493	0.969368572	922.2453217	-0.617275021	0.455161017	922.2453217
FBgn0033785	Sans ortholog	395.0505744	0.091681029	0.994151247	395.0505744	-0.232497462	0.838932786	395.0505744
FBgn0033787	CG13321 gene product from transcrip	5031.402624	-0.43970934	0.756991008	5031.402624	-0.44124598	0.687397308	5031.402624
FBgn0033791	Derailed 2	89.32056231	0.038503028	0.998322595	89.32056231	0.020106097	0.997246866	89.32056231
FBgn0033789	CG13324 gene product from transcrip	665.4242489	-1.30639324	0.000203217	665.4242489	-0.651927963	0.229621519	665.4242489
FBgn0033788	CG13323 gene product from transcrip	2389.889154	-1.194144749	0.013185216	2389.889154	-0.871259794	0.145701698	2389.889154
FBgn0005624	Posterior sex combs	553.1264005	0.137857928	0.975999817	553.1264005	0.092671381	0.953470108	553.1264005
FBgn0004512	Multi drug resistance 49	2330.558014	-0.142706289	0.967901085	2330.558014	-0.188021688	0.848588436	2330.558014
FBgn0050487	CG30487 gene product from transcrip	7.832218009	0.169659039	0.988354972	7.832218009	-0.231593238	0.915890998	7.832218009
FBgn0033784	CG13322 gene product from transcrip	690.5093189	-0.236510473	0.826123977	690.5093189	-0.18148939	0.841278077	690.5093189
FBgn0033783	CG17019 gene product from transcrip	1502.643027	-0.36289032	0.411204308	1502.643027	-0.38834643	0.336746575	1502.643027
FBgn0025692	CG3814 gene product from transcript	0			0			0
FBgn0033778	CG3790 gene product from transcript	0			0			0
FBgn0033777	CG17574 gene product from transcrip	1388.595502	-0.534903907	0.396447053	1388.595502	-0.473734348	0.485933377	1388.595502
FBgn0050486	CG30486 gene product from transcrip	0			0			0
FBgn0050488	antares	7.478762051	0.825517441	0.400362196	7.478762051	0.579523343	0.650864719	7.478762051
FBgn0033775	CG17577 gene product from transcrip	680.9467918	-0.250730933	0.72740743	680.9467918	-0.095528121	0.932579238	680.9467918
FBgn0011592	frazzled	1281.498559	-0.159346643	0.972945531	1281.498559	-0.074207758	0.971833087	1281.498559
FBgn0033753	CG8587 gene product from transcript	121.6013084	0.222151612	0.949623474	121.6013084	0.356845207	0.699950039	121.6013084
FBgn0003527	stand still	7.265861122	0.352042625	0.898129226	7.265861122	-0.022579939	0.996470448	7.265861122
FBgn0033755	Chloride channel-b	1377.750328	-0.080261333	0.985645036	1377.750328	-0.002375837	1	1377.750328
FBgn0050055	ncRNA	215.8595353	-0.153998305	0.979231897	215.8595353	-0.129950242	0.944730798	215.8595353
FBgn0027356	Amphiphysin	2430.612569	0.226140118	0.944197659	2430.612569	0.575868211	0.344344471	2430.612569
FBgn0004435	G protein alpha q subunit	639.670054	0.246282205	0.960742862	639.670054	0.493264422	0.642743241	639.670054
FBgn0050054	CG30054 gene product from transcrip	37.71683909	0.16050208	0.987910537	37.71683909	0.102004846	0.971833087	37.71683909
FBgn0033756	CG17760 gene product from transcrip	78.81476761	-0.0188165	0.998322595	78.81476761	0.127545652	0.953470108	78.81476761
FBgn0028683	CG12372 gene product from transcrip	469.3654095	-0.008748838	0.998322595	469.3654095	0.255688755	0.719387902	469.3654095
FBgn0011604	Imitation SWI	1708.630644	0.025775699	0.99723561	1708.630644	-0.120696261	0.915954217	1708.630644
FBgn0033762	Zinc transporter 49B	1191.466252	-0.16238851	0.844755814	1191.466252	-0.088607567	0.915954217	1191.466252

FBgn0033449	CG1663 gene product from transcript	150.9396084	-0.061371027	0.994497358	150.9396084	-0.135274858	0.915954217	150.9396084	0.370005742	0.367736693
FBgn0033451	CG1665 gene product from transcript	751.144766	0.753248039	0.45251689	751.144766	0.681990435	0.522753428	751.144766	0.143719542	0.910955404
FBgn0023180	Origin recognition complex subunit 6	86.72585706	-0.078856953	0.994497358	86.72585706	-0.044306769	0.986877529	86.72585706	0.074776939	0.931254569
FBgn0033450	CG12924 gene product from transcript	171.7494108	-0.138513845	0.979231897	171.7494108	-0.145314173	0.929503482	171.7494108	0.562016063	0.218872285
FBgn0033448	CG1623 gene product from transcript	2548.152528	-0.442626621	0.386851348	2548.152528	-0.462484292	0.332530839	2548.152528	-0.682315468	0.030394598
FBgn0033447	dilatory	27.47671278	0.060621962	0.995682185	27.47671278	-0.170774468	0.948575578	27.47671278	0.049102112	0.974403296
FBgn0050001	CG30001 gene product from transcript	95.33280842	-0.057035117	0.994876798	95.33280842	0.061611827	0.980691994	95.33280842	0.263199303	0.688597865
FBgn0033446	CG1648 gene product from transcript	5586.776045	-0.153078502	0.975999817	5586.776045	-0.192975128	0.888777857	5586.776045	-0.356005475	0.525976881
FBgn0027589	CG1688 gene product from transcript	35.26336487	-0.0914204	0.994497358	35.26336487	-0.194332762	0.937211825	35.26336487	0.582645119	0.454317938
FBgn0033442	CG1690 gene product from transcript	4.709331663	0.606387306	0.165317004	4.709331663	0		4.709331663	-0.607655156	0.097063432
FBgn0023143	Ubiquitin activating enzyme 1	15625.19357	-0.124125213	0.946165413	15625.19357	-0.043435542	0.971833087	15625.19357	0.309553525	0.2473945
FBgn0033438	Matrix metalloproteinase 2	2073.900702	0.018712472	0.998322595	2073.900702	-0.465779816	0.401409705	2073.900702	0.408015757	0.352239126
FBgn0033437	CG12926 gene product from transcript	2492.95321	-0.572124967	0.213812507	2492.95321	-0.321256141	0.660844743	2492.95321	0.367746518	0.419444726
FBgn0016970	lethal (2) k10201	208.2477928	-0.542442137	0.3397435	208.2477928	-0.364307034	0.645025488	208.2477928	0.339475438	0.518156062
FBgn0041087	wunen-2	300.5903855	-0.151828424	0.975999817	300.5903855	-0.185487618	0.896716593	300.5903855	0.343397458	0.537838452
FBgn0033413	preli-like	2422.348479	-0.135552156	0.956083993	2422.348479	-0.132412399	0.886324007	2422.348479	-0.150371234	0.744009137
FBgn0017558	Pyruvate dehydrogenase kinase	6492.339564	-0.342948329	0.552093055	6492.339564	-0.045617572	0.980546945	6492.339564	0.032480031	0.964876035
FBgn0004360	Wnt oncogene analog 2	246.9922599	-0.155006539	0.978641661	246.9922599	-0.265839346	0.825788686	246.9922599	0.440109279	0.421665501
FBgn0033422	Odorant receptor 45b	0			0			0		
FBgn0033423	CG1809 gene product from transcript	0			0			0		
FBgn0033426	CG1814 gene product from transcript	651.3756559	-0.354150168	0.558559435	651.3756559	-0.167132434	0.867222024	651.3756559	-0.170529705	0.750427952
FBgn0033428	CG1818 gene product from transcript	1911.896743	-0.065928018	0.994497358	1911.896743	-0.159024924	0.907409646	1911.896743	0.077836307	0.921050636
FBgn0025286	Ribosomal protein L31	35825.63309	0.056272171	0.995120986	35825.63309	0.054841354	0.985550158	35825.63309	-0.386285934	0.541680107
FBgn0033431	CG1827 gene product from transcript	1234.073334	-0.484170353	0.327857108	1234.073334	-0.638651315	0.101734457	1234.073334	0.508337193	0.167276735
FBgn0010342	Microtubule-associated protein 60	1945.059429	-0.158013391	0.88370699	1945.059429	-0.138197484	0.838932786	1945.059429	0.213529838	0.500735201
FBgn0033434	CG1902 gene product from transcript	2060.416016	-0.211187094	0.823279465	2060.416016	-0.09866482	0.92774174	2060.416016	0.248232121	0.505640514
FBgn0050338	CG30338 gene product from transcript	111.4150983	0.454086486	0.583269985	111.4150983	0.01135913	0.997344995	111.4150983	-0.331068996	0.576125453
FBgn0050339	CG30339 gene product from transcript	304.3871711	-0.559244128	0.57599282	304.3871711	-0.397365561	0.745673628	304.3871711	-0.105964231	0.922829316
FBgn0050340	CG30340 gene product from transcript	9.707674238	-0.624633436	0.678344496	9.707674238	-0.54673708	0.687453403	9.707674238	0.575457353	0.503619102
FBgn0033429	CG12929 gene product from transcript	324.3801026	-0.092076081	0.994497358	324.3801026	-0.238753117	0.90250871	324.3801026	-0.242447827	0.795974014
FBgn0033427	CG1868 gene product from transcript	210.5620759	-0.019384583	0.998322595	210.5620759	-0.018183809	0.994998875	210.5620759	-0.029255836	0.970791391
FBgn0033421	CG1888 gene product from transcript	522.8353104	-0.147663329	0.979231897	522.8353104	-0.325753336	0.741063982	522.8353104	0.009746599	0.992480194
FBgn0029092	CG11804 gene product from transcript	9330.273696	-0.158312184	0.950976319	9330.273696	-0.287408682	0.645902731	9330.273696	0.377564857	0.299104401
FBgn0033412	CG13955 gene product from transcript	1.844230963	0.029814743	0.998322595	1.844230963	0.028223431		1.844230963	0.258101764	0.689610074
FBgn0016078	wunen	916.129224	-0.30436954	0.880088833	916.129224	-0.539372642	0.473746073	916.129224	0.481022176	0.39655054
FBgn0028473	Novel nucleolar protein 1	11884.70322	0.038521698	0.994497358	11884.70322	-0.33070685	0.296529859	11884.70322	0.099982567	0.802870111
FBgn0033408	CG8800 gene product from transcript	7.338187522	0.132824095	0.994497358	7.338187522	0.076466517	0.984721517	7.338187522	-0.241720844	0.827473121
FBgn0033405	CG13954 gene product from transcript	28.64632905	0.038896512	0.998322595	28.64632905	0.05088181	0.990025502	28.64632905	-0.365441252	0.699095569
FBgn0011746	anachronism	198.6153789	0.031472688	0.998322595	198.6153789	0.197216684	0.904246991	198.6153789	0.419969362	0.473526049
FBgn0025709	CG8083 gene product from transcript	274.1808149	-0.318776318	0.708898746	274.1808149	-0.426684084	0.440811166	274.1808149	0.116811163	0.851918021
FBgn0033371	Concentrative Nucleoside Transporter 1	2.841210467	0.474233986	0.693008237	2.841210467	0.392801201		2.841210467	-0.275041666	0.738186023
FBgn0010220	DEAD box protein 45A	427.0739241	-0.11780308	0.967901085	427.0739241	-0.10484154	0.918961524	427.0739241	0.055438764	0.923564636
FBgn0033376	CG8777 gene product from transcript	401.6698955	-0.374790528	0.33180341	401.6698955	-0.205015367	0.746258753	401.6698955	0.276886543	0.407563539
FBgn0015838	Van Gogh	1456.656761	-0.113545811	0.975999817	1456.656761	-0.062874823	0.966441311	1456.656761	0.302030246	0.446805064
FBgn0033378	tsunagi	1696.204333	-0.069950075	0.99157593	1696.204333	-0.050902807	0.971833087	1696.204333	0.061594005	0.915291908

[illegible]

FBgn0019985	metabotropic Glutamate Receptor	59.15988901	-0.11186128	0.994497358	59.15988901	0.211971485	0.914502763	59.15988901	[illegible]
FBgn0019650	twin of eyeless	275.7484819	-0.022108968	0.998322595	275.7484819	0.081882173	0.965276384	275.7484819	[illegible]
FBgn0010217	ATP synthase-beta	45441.36586	0.085927539	0.994497358	45441.36586	0.437572837	0.510930867	45441.36586	[illegible]
FBgn0013749	ADP ribosylation factor at 102F	2089.798636	0.175437033	0.927121587	2089.798636	0.553942579	0.124097763	2089.798636	[illegible]
FBgn0002521	pleiohomeotic	1343.373118	-0.225698499	0.917248088	1343.373118	0.123511623	0.936641868	1343.373118	[illegible]
FBgn0052017	CG32017 gene product from transcrip	281.9864454	-0.120891249	0.99157593	281.9864454	0.215965843	0.896524206	281.9864454	[illegible]
FBgn0039928	Calsyntenin-1	2081.592675	-0.070340336	0.994497358	2081.592675	0.337703382	0.773294804	2081.592675	[illegible]
FBgn0024913	Activin-beta	107.6438945	-0.379450685	0.77169846	107.6438945	-0.332429532	0.770169252	107.6438945	[illegible]
FBgn0011642	Zyxin	1190.748827	-0.133691081	0.956325514	1190.748827	0.024299262	0.988881869	1190.748827	[illegible]
FBgn0039929	CG11076 gene product from transcrip	1050.231182	-0.010559789	0.998322595	1050.231182	0.389668437	0.475620641	1050.231182	[illegible]
FBgn0039930	CG11077 gene product from transcrip	1.008368221	-0.062209049		1.008368221	0.055322171		1.008368221	[illegible]
FBgn0039932	fussel	19.17812186	0.150986921	0.993127879	19.17812186	0.479030397	0.740981826	19.17812186	[illegible]
FBgn0052016	eIF4E-Transporter	1399.982031	-0.07764439	0.98775218	1399.982031	0.089859251	0.932579238	1399.982031	[illegible]
FBgn0023213	eukaryotic translation initiation factor [illegible]	8352.428863	0.127186982	0.988852951	8352.428863	0.437810666	0.621010334	8352.428863	[illegible]
FBgn0039936	CG11148 gene product from transcrip	3083.905142	-0.068515756	0.994497358	3083.905142	0.0644102	0.973718552	3083.905142	[illegible]
FBgn0039937	forkhead domain 102C	18.66332234	-0.236219298	0.975861786	18.66332234	-0.112186445	0.971833087	18.66332234	[illegible]
FBgn0039938	CG11153 gene product from transcrip	322.2909926	-0.252160495	0.871091663	322.2909926	-0.135123177	0.922352014	322.2909926	[illegible]
FBgn0039923	Mediator complex subunit 26	1672.28604	0.002010594	0.999629143	1672.28604	0.284340521	0.74945674	1672.28604	[illegible]
FBgn0031208	CG11023 gene product from transcrip	14.15250195	-0.239638721	0.975999817	14.15250195	-0.261978392	0.914502763	14.15250195	[illegible]
FBgn0024698	Cleavage and polyadenylation specificit	480.5140248	-0.150857415	0.961435662	480.5140248	0.058679065	0.971833087	480.5140248	[illegible]
FBgn0030538	CG13402 gene product from transcrip	0.941866333	0.210118031		0.941866333	0		0.941866333	[illegible]
FBgn0010473	turtle	780.773526	0.036044654	0.994892516	780.773526	-0.094622225	0.934188786	780.773526	[illegible]
FBgn0003386	Shaker cognate w	98.74256515	-0.33428366	0.901513224	98.74256515	-0.365113811	0.796573058	98.74256515	[illegible]
FBgn0051481	proboscipedia	35.59723475	0.2821644	0.954886241	35.59723475	-0.008958819	0.999512309	35.59723475	[illegible]
FBgn0004577	Peroxidase	4844.484412	-0.00158025	0.999629143	4844.484412	0.436607013	0.22366388	4844.484412	[illegible]
FBgn0010453	Wnt oncogene analog 4	319.4976688	-0.118802349	0.994497358	319.4976688	-0.095924171	0.971833087	319.4976688	[illegible]
FBgn0010549	lethal (2) 03659	167.5428684	0.066143349	0.994497358	167.5428684	0.213179533	0.849115479	167.5428684	[illegible]
FBgn0012018	transfer RNA:tyr1:28C	0			0			0	[illegible]
FBgn0012016	transfer RNA:tyr1:22Fa	0.351559643	0		0.351559643	0		0.351559643	[illegible]
FBgn0012017	transfer RNA:tyr1:22Fb	0.360834224	-0.026426919		0.360834224	-0.06934085		0.360834224	[illegible]
FBgn0052525	transfer RNA:CR32525	0.186436866	0.066048487		0.186436866	0		0.186436866	[illegible]
FBgn0052520	transfer RNA:CR32520	0.072449522	0		0.072449522	0		0.072449522	[illegible]
FBgn0011910	transfer RNA:leu:49Fb	0.442858418	0		0.442858418	0		0.442858418	[illegible]
FBgn0011909	transfer RNA:leu:49Fa	0.100708642	0		0.100708642	0		0.100708642	[illegible]
FBgn0014342	meiosis I arrest	80.77812183	0.007882615	0.998322595	80.77812183	-0.150312473	0.930935455	80.77812183	[illegible]
FBgn0032721	CG10602 gene product from transcrip	3526.586185	-0.254560996	0.614332623	3526.586185	0.148883429	0.825700404	3526.586185	[illegible]
FBgn0032720	mitochondrial ribosomal protein L13	214.4459575	0.162454871	0.975861786	214.4459575	0.051210174	0.985550158	214.4459575	[illegible]
FBgn0036290	CG10638 gene product from transcrip	1528.932925	0.138644815	0.954378726	1528.932925	0.259915498	0.650864719	1528.932925	[illegible]
FBgn0036362	CG10725 gene product from transcrip	3423.542709	-0.026004998	0.998322595	3423.542709	0.001685112	1	3423.542709	[illegible]
FBgn0002565	Larval serum protein 2	54873.93462	0.145721604	0.979231897	54873.93462	0.322584832	0.747420655	54873.93462	[illegible]
FBgn0036255	Autophagy-specific gene 12	110.8979676	-0.05817253	0.9945211	110.8979676	-0.213465332	0.864515109	110.8979676	[illegible]
FBgn0017424		0.969228123	0.056333339		0.969228123	-0.074422475		0.969228123	[illegible]
FBgn0025885	CG11143 gene product from transcrip	5068.646374	0.387526481	0.396544824	5068.646374	0.491773645	0.174302958	5068.646374	[illegible]
FBgn0014018	Relish	2849.509135	0.307744358	0.753828842	2849.509135	0.041953252	0.985659903	2849.509135	[illegible]
[illegible]	[illegible]	[illegible]	[illegible]	[illegible]	[illegible]	[illegible]	[illegible]	[illegible]	[illegible]

FBgn0053425	5S ribosomal RNA	0			0			0
FBgn0053426	5S ribosomal RNA	0			0			0
FBgn0053427	5S ribosomal RNA	0			0			0
FBgn0053428	5S ribosomal RNA	0.519982508	-0.175572253		0.519982508	-0.17099359		0.519982508
FBgn0053429	5S ribosomal RNA	0			0			0
FBgn0053430	5S ribosomal RNA	0			0			0
FBgn0053431	5S ribosomal RNA	0			0			0
FBgn0053432	5S ribosomal RNA	0			0			0
FBgn0053433	5S ribosomal RNA	0			0			0
FBgn0053434	5S ribosomal RNA	0			0			0
FBgn0053435	5S ribosomal RNA	0			0			0
FBgn0053436	5S ribosomal RNA	0			0			0
FBgn0053437	5S ribosomal RNA	0			0			0
FBgn0053438	5S ribosomal RNA	0			0			0
FBgn0053439	5S ribosomal RNA	0			0			0
FBgn0053440	5S ribosomal RNA	0			0			0
FBgn0053441	5S ribosomal RNA	0			0			0
FBgn0053442	5S ribosomal RNA	0			0			0
FBgn0053443	5S ribosomal RNA	0			0			0
FBgn0053444	5S ribosomal RNA	0			0			0
FBgn0053445	5S ribosomal RNA	0			0			0
FBgn0053446	5S ribosomal RNA	0			0			0
FBgn0053447	5S ribosomal RNA	0			0			0
FBgn0053448	5S ribosomal RNA	0			0			0
FBgn0053449	5S ribosomal RNA	0			0			0
FBgn0053450	5S ribosomal RNA	0			0			0
FBgn0053451	5S ribosomal RNA	0.07379547	0.108851729		0.07379547	0		0.07379547
FBgn0053452	5S ribosomal RNA	0.147619473	0		0.147619473	0		0.147619473
FBgn0040849	Ionotropic receptor 41a	4.92596516	-0.121307031	0.994497358	4.92596516	-0.046539812		4.92596516
FBgn0033005	CG3107 gene product from transcript	2310.10301	0.143148591	0.975532686	2310.10301	0.585006229	0.212614568	2310.10301
FBgn0033010	CG3136 gene product from transcript	1237.870498	0.040371925	0.997459641	1237.870498	0.391764899	0.74117838	1237.870498
FBgn0026238	gustavus	1791.18242	-0.133590431	0.926642056	1791.18242	-0.009022887	0.997037674	1791.18242
FBgn0013348	Troponin C at 41C	34.98762476	0.673103727	0.606342502	34.98762476	0.897417698	0.321040925	34.98762476
FBgn0032949	CG3305 gene product from transcript	10367.53173	-0.271464915	0.805580095	10367.53173	-0.16252285	0.886324007	10367.53173
FBgn0030715	Odorant receptor 13a	0.450830923	0.002665209		0.450830923	-0.058333252		0.450830923
FBgn0030717	CG8931 gene product from transcript	404.659966	-0.191131795	0.920855571	404.659966	-0.144208412	0.904246991	404.659966
FBgn0000611	extradenticle	3033.699548	-0.107897736	0.973453438	3033.699548	0.099058156	0.922400604	3033.699548
FBgn0030720	CG8939 gene product from transcript	985.8810827	0.039973057	0.994497358	985.8810827	0.137158897	0.864607967	985.8810827
FBgn0030721	CG12698 gene product from transcrip	5.41878286	0.96704567	0.230092183	5.41878286	-0.171640074		5.41878286
FBgn0030723	CG14948 gene product from transcript	47.50888989	-0.267618072	0.954378726	47.50888989	-0.260610766	0.884342164	47.50888989
FBgn0030725	CG8958 gene product from transcript	4.344547822	-0.014230565	0.998322595	4.344547822	0.092542957		4.344547822
FBgn0028397	CG9214 gene product from transcript	164.2995059	0.441168356	0.807463547	164.2995059	0.571173744	0.592909786	164.2995059
FBgn0030724	CG9212 gene product from transcript	3722.891462	0.138531038	0.972945531	3722.891462	0.359689033	0.58226435	3722.891462
FBgn0030722	CG12395 gene product from transcrip	0.951169446	0.157785806		0.951169446	-0.032532096		0.951169446
FBgn0003969	vacuolar peduncle	2036.568051	-0.082362244	0.993127879	2036.568051	-0.127981613	0.918961524	2036.568051
FBgn0030719	CG9177 gene product from transcript	7516.391727	0.021868212	0.998322595	7516.391727	0.189122061	0.878786989	7516.391727
FBgn0030718	CG9172 gene product from transcript	2344.651371	0.17714906	0.948196716	2344.651371	0.313523026	0.645902731	2344.651371
FBgn0030716	CG9170 gene product from transcript	499.0268081	-0.26092523	0.72740743	499.0268081	-0.508900769	0.138631416	499.0268081
FBgn0053236	CG33236 gene product from transcrip	0			0			0
FBgn0053237	CG33237 gene product from transcrip	0			0			0
FBgn0053238	CG33238 gene product from transcrip	0			0			0
FBgn0053239	CG33239 gene product from transcrip	0			0			0
FBgn0053240	CG33240 gene product from transcrip	0			0			0
FBgn0053241	CG33241 gene product from transcrip	0			0			0
FBgn0053242	CG33242 gene product from transcrip	0			0			0
FBgn0053243	CG33243 gene product from transcrip	0			0			0
FBgn0053244	CG33244 gene product from transcrip	0			0			0
FBgn0053245	CG33245 gene product from transcrip	0			0			0
FBgn0053246	CG33246 gene product from transcrip	0			0			0
FBgn0053247	CG33247 gene product from transcrip	0			0			0
FBgn0029174	FK506-binding protein FKBP59	3950.699124	-0.013355999	0.998322595	3950.699124	0.031332844	0.986877529	3950.699124
FBgn0032153	CG4537 gene product from transcript	77.98338114	0.106333992	0.994497358	77.98338114	0.120428072	0.959367674	77.98338114
FBgn0010520	Bekka	232.5803494	-0.036645598	0.996536713	232.5803494	0.070712318	0.971833087	232.5803494
FBgn0024285	CG4602 gene product from transcript	1898.98333	-0.099850116	0.985645036	1898.98333	-0.042680589	0.983090698	1898.98333
FBgn0040064	yippee interacting protein 2	8988.159139	0.137655889	0.969368572	8988.159139	0.174113644	0.860946594	8988.159139
FBgn0032160	CG4598 gene product from transcript	1769.763646	0.126193937	0.963501097	1769.763646	0.239954392	0.684990219	1769.763646
FBgn0032161	CG4594 gene product from transcript	445.0651483	-0.170148803	0.97064214	445.0651483	0.024848052	0.993457973	445.0651483
FBgn0032162	CG4592 gene product from transcript	1187.536505	0.215181519	0.934448847	1187.536505	0.48833904	0.425684924	1187.536505
FBgn0032166	CG4619 gene product from transcript	329.8733732	0.092902709	0.987910537	329.8733732	0.184899732	0.836520481	329.8733732
FBgn0032167	CG5853 gene product from transcript	303.6562077	-0.458143064	0.777805509	303.6562077	-0.427919053	0.746380565	303.6562077
FBgn0051713	diadenosine tetraphosphate hydrolase	0			0			0
FBgn0051712	CG31712 gene product from transcrip	1325.688106	0.085568402	0.985161753	1325.688106	0.00790952	0.997246866	1325.688106
FBgn0051876	Cuticular protein 30F	3.194793757	0.898369733	0.18504247	3.194793757	0.238907493		3.194793757
FBgn0032163	CG13125 gene product from transcrip	1.926002413	0.14696869	0.988216049	1.926002413	0.156632745		1.926002413
FBgn0025700	CG5885 gene product from transcript	5258.302979	0.392505688	0.569019066	5258.302979	0.790174691	0.030964771	5258.302979
FBgn0032156	CG13124 gene product from transcrip	3406.583581	0.043662581	0.995120986	3406.583581	0.013066024	0.997246866	3406.583581
FBgn0053300	Mucin 30E	1.015980184	-0.087614527		1.015980184	-0.235320358		1.015980184

[illegible]

FBgn0051992	gawky	8023.22875	-0.063221138	0.994497358	8023.22875	-0.073392753	0.956742869
FBgn0028341	lethal (1) G0232	3640.830486	0.165838746	0.915675288	3640.830486	0.026467208	0.988117656
FBgn0032974	CG3651 gene product from transcript	724.2978543	-0.060905333	0.994497358	724.2978543	-0.024747685	0.989048077
FBgn0029870	Mitochondrial assembly regulatory fact	3229.03821	-0.271766765	0.387586645	3229.03821	-0.235612882	0.498106822
FBgn0035001	Sterile20-like kinase	3276.234757	0.124183031	0.96882922	3276.234757	0.066039672	0.961113238
FBgn0032666	CG5758 gene product from transcript	1064.224748	-0.275813997	0.838879663	1064.224748	-0.178566984	0.883802014
FBgn0026533	CG5935 gene product from transcript	4623.199411	-0.075338905	0.993127879	4623.199411	-0.046299682	0.980546945
FBgn0036534	Decapping protein 2	2370.410919	-0.170976584	0.973453438	2370.410919	0.114754356	0.950102927
FBgn0036144	CG6207 gene product from transcript	1290.473471	0.415490332	0.567039952	1290.473471	0.243899035	0.811972621
FBgn0033265	coilin	269.562148	-0.270090805	0.694459829	269.562148	-0.020061557	0.991444605
FBgn0038139	Pyrokinin 2 receptor 2	476.5285945	0.657539577	0.31925014	476.5285945	-0.052983685	0.98613036
FBgn0027529	CG8920 gene product from transcript	2264.683594	-0.126598794	0.94728993	2264.683594	-0.249807933	0.579112715
FBgn0031802	pickpocket 7	0.314400184	-0.098911827		0.314400184	-0.142618533	
FBgn0037234	CG9795 gene product from transcript	846.7956636	-0.124435211	0.949774689	846.7956636	-0.260618806	0.54969847
FBgn0030641	CG6299 gene product from transcript	46.5797476	-0.465816053	0.72893315	46.5797476	-0.314488295	0.82754465
FBgn0029736	CG4041 gene product from transcript	1214.183076	0.068123861	0.994497358	1214.183076	-0.083557787	0.949757912
FBgn0030662	CG9220 gene product from transcript	1482.330484	0.060275321	0.994497358	1482.330484	-0.056392127	0.976646329
FBgn0035978	CG4347 gene product from transcript	4068.733594	0.310268487	0.72740743	4068.733594	0.478852367	0.323737992
FBgn0031530	polypeptide GalNAc transferase 2	1117.817695	-0.00578369	0.998322595	1117.817695	0.330706396	0.645558524
FBgn0037100	Capability receptor	35.38353792	-0.264680792	0.955137318	35.38353792	0.121704735	0.961113238
FBgn0032957	CG2225 gene product from transcript	481.1520899	0.057590599	0.994497358	481.1520899	0.117223984	0.920812352
FBgn0034417	CG15117 gene product from transcrip	277.9154016	-0.33219287	0.717467024	277.9154016	-0.23539275	0.805951663
FBgn0031294	IA-2 ortholog	2699.553777	0.184205926	0.850206448	2699.553777	0.060988065	0.958134108
FBgn0000542	echinus	1102.512663	0.131769406	0.972176266	1102.512663	0.032040683	0.9872372
FBgn0050372	ArfGAP with SH3 domain, ankyrin repea	1949.457277	-0.140314347	0.938791798	1949.457277	-0.258838084	0.598986434
FBgn0030844	pickpocket 23	2.42868841	-0.020153866	0.998322595	2.42868841	-0.013029034	
FBgn0031536	CG3248 gene product from transcript	644.7532796	-0.089634339	0.993908998	644.7532796	-0.108645668	0.94714921
FBgn0039675	pickpocket 21	0.601008454	-0.135016371		0.601008454	-0.088798158	
FBgn0039676	pickpocket 20	0.418435974	0.049577855		0.418435974	0.064376946	
FBgn0020279	lingerer	11322.04754	0.176679712	0.823279465	11322.04754	0.096293966	0.912868368
FBgn0015777	nervana 2	1765.500802	-0.161939537	0.923470826	1765.500802	0.109806464	0.915954217
FBgn0020546	ncRNA	9.409252291	-0.337595471	0.947347039	9.409252291	-0.060107474	0.988378975
FBgn0011998	transfer RNA:val3b:84Da	0			0		
FBgn0052547	CG32547 gene product from transcrip	90.73249905	0.617388741	0.492281998	90.73249905	0.308908214	0.834775152
FBgn0037560	pseudo	271.6589453	-0.172929327	0.969626359	271.6589453	-0.183927886	0.901802431
FBgn0029105	alpha-catenin related	108.5365915	-0.065631636	0.994497358	108.5365915	-0.237342286	0.855882953
FBgn0038570	Peroxiredoxin 5	0			0		
FBgn0038571	CG7215 gene product from transcript	0			0		
FBgn0039308	CG11889 gene product from transcrip	0			0		
FBgn0039309	CG11891 gene product from transcrip	0			0		
FBgn0036170	CG11714 gene product from transcrip	0			0		
FBgn0036169	alpha-L-fucosidase	0			0		
FBgn0031039	CG14209 gene product from transcrip	0			0		
FBgn0031038	CG14208 gene product from transcrip	0			0		

[illegible]

FBgn0053908	CG33908 gene product from transcrip	1.53644416	-0.070096149		1.53644416	0.332475359		1.53644416	0.084978626	0.921857951
FBgn0051611	CG31611 gene product from transcrip	0			0			0		
FBgn0061209	CG17949 gene product from transcrip	0.395504545	-0.036545818		0.395504545	0.121065121		0.395504545	0	
FBgn0053909	CG33909 gene product from transcrip	0			0			0		
FBgn0053910	CG33910 gene product from transcrip	0			0			0		
FBgn0028991	sequoia	0			0			0		
FBgn0053912	CG33912 gene product from transcrip	0			0			0		
FBgn0053914	CG33914 gene product from transcrip	22.38183708	-0.358477018	0.918956451	22.38183708	-0.341617052	0.850323429	22.38183708	0.420213737	0.64250158
FBgn0065107	small non-messenger RNA 128	0			0			0		
FBgn0025455	Cyclin T	1801.082807	0.251075443	0.885470186	1801.082807	0.217006565	0.845042502	1801.082807	0.104557659	0.889440369
FBgn0065102	small non-messenger RNA 184	0			0			0		
FBgn0065094	small non-messenger RNA 419	0			0			0		
FBgn0038683	CG11779 gene product from transcrip	644.1497394	-0.074392723	0.988354972	644.1497394	0.283219946	0.527183686	644.1497394	-0.253072364	0.445542507
FBgn0038682	CG5835 gene product from transcript	805.3666741	-0.754788208	0.000689152	805.3666741	-0.687024174	0.003944762	805.3666741	0.25950792	0.432436472
FBgn0033628	CG13203 gene product from transcrip	31.06175458	-0.324553747	0.948242463	31.06175458	-0.267370808	0.906074835	31.06175458	0.02073434	0.990113525
FBgn0033739	Dystrobrevin-like	892.736566	0.133481259	0.963501097	892.736566	0.263442912	0.666695515	892.736566	-0.211541301	0.62697774
FBgn0034081	CG10731 gene product from transcrip	360.0247895	0.227583344	0.902352095	360.0247895	-0.065304948	0.973040341	360.0247895	-0.486004888	0.2473945
FBgn0029175	CG8433 gene product from transcript	327.4701011	-0.14791811	0.966157244	327.4701011	-0.059822755	0.973040341	327.4701011	0.363078706	0.384841052
FBgn0027358	Translocase of inner membrane 9b	0			0			0		
FBgn0031041	CG12788 gene product from transcrip	0			0			0		
FBgn0003435	smooth	917.2166141	0.293917055	0.919906743	917.2166141	0.125630559	0.952484385	917.2166141	0.091937605	0.930658001
FBgn0024251	bobby sox	11.80087906	-0.271436582	0.968528723	11.80087906	0.083047999	0.984079364	11.80087906	0.348722237	0.740745928
FBgn0024182	waclaw	0			0			0		
FBgn0031455	Snapin ortholog (H. sapiens)	0			0			0		
FBgn0031454	CG9960 gene product from transcript	0			0			0		
FBgn0053476	CG33476 gene product from transcrip	0.207742118	0.067675148		0.207742118	-0.049276071		0.207742118	-0.121504776	
FBgn0053919	CG33919 gene product from transcrip	0.985606768	0.226704011		0.985606768	0.395768996		0.985606768	-0.164772653	
FBgn0038032	CG10096 gene product from transcrip	0			0			0		
FBgn0038033	CG10097 gene product from transcrip	0			0			0		
FBgn0052652	CG32652 gene product from transcrip	3.896296087	0.627490329	0.15534653	3.896296087	0.043244589		3.896296087	-0.629149838	0.091991723
FBgn0053920	CG33920 gene product from transcrip	4.939021145	-0.104754066	0.994497358	4.939021145	-0.167400148		4.939021145	-0.179109801	0.86754205
FBgn0051224	CG31224 gene product from transcrip	2000.157084	0.308114528	0.863561469	2000.157084	0.138557243	0.939574224	2000.157084	0.198071228	0.796336067
FBgn0065048	ncRNA	4.492909077	0.172288075	0.987458415	4.492909077	0.16443392		4.492909077	-0.33731693	0.71100653
FBgn0011640	CG8597 gene product from transcript	3179.179923	0.005712293	0.998322595	3179.179923	-0.09851229	0.941360428	3179.179923	-0.012909954	0.988196052
FBgn0053922	CG33922 gene product from transcrip	0			0			0		
FBgn0053923	CG33923 gene product from transcrip	0.07379547	0.108851729		0.07379547	0		0.07379547	-0.111773044	
FBgn0065099	small nuclear RNA 7SK	638.2945376	-0.040608913	0.997459641	638.2945376	0.042109209	0.990025502	638.2945376	-0.198993061	0.828432542
FBgn0053926	CG33926 gene product from transcrip	761.2194726	-3.735976375	8.31E-21	761.2194726	-2.129587527	2.71E-06	761.2194726	0.123807638	0.917015308
FBgn0053927	CG33927 gene product from transcrip	0.639249431	-0.026064599		0.639249431	-0.011778251		0.639249431	0.110322576	
FBgn0053928	CG33928 gene product from transcrip	1.819175997	0.190647816	0.974745767	1.819175997	-0.096863753		1.819175997	-0.087615967	0.931972736
FBgn0067311	Chemosensory protein B 38b	0.678319441	-0.161364103		0.678319441	-0.157992049		0.678319441	0.149008093	
FBgn0024293	Serpin 43Ab	2859.529289	0.296664178	0.900374228	2859.529289	-0.054218266	0.98613036	2859.529289	-0.897302916	0.051768217
FBgn0038888	Chemosensory protein B 93a	0			0			0		
FBgn0053929	pseudo	0			0			0		
FBgn0065076	ncRNA	18.24998705	0.090905684	0.9945211	18.24998705	0.033654638	0.994998875	18.24998705	-0.279032263	0.799331856
FBgn0033564	Peroxin 6	431.9783069	0.046872387	0.994497358	431.9783069	0.196150488	0.765698719	431.9783069	-0.27173883	0.423364229
FBgn0061356	CG18003 gene product from transcrip	3298.663201	-0.079761157	0.994497358	3298.663201	-0.290064776	0.728676069	3298.663201	-0.710118875	0.042845919
FBgn0066304	CG33931 gene product from transcrip	0			0			0		
FBgn0066303	CG33932 gene product from transcrip	0			0			0		
FBgn0260466	I'm not dead yet 2	0			0			0		
FBgn0064119	CG33934 gene product from transcrip	0			0			0		
FBgn0002709	meiotic 218	11.50134745	0.039233212	0.998322595	11.50134745	0.167080323	0.950970297	11.50134745	0.256524549	0.823519204
FBgn0028581	CG33935 gene product from transcrip	0			0			0		
FBgn0063127	putative noncoding RNA 002:3R	1306.43148	0.005403603	0.998322595	1306.43148	-0.249494187	0.759743592	1306.43148	0.527706928	0.128907673
FBgn0047095	putative noncoding RNA 004:X	146.070126	-0.250966597	0.958580291	146.070126	-0.351324134	0.801423447	146.070126	0.414719295	0.578345412
FBgn0062928	putative noncoding RNA 009:3L	139.2605604	2.179688499	5.83E-12	139.2605604	1.881521609	2.71E-08	139.2605604	-2.209776647	2.41E-12
FBgn0062961	putative noncoding RNA 016:2R	73.43549487	-0.756640052	0.363120737	73.43549487	-0.088189137	0.976286705	73.43549487	-0.059457504	0.964377123
FBgn0047334	CG33943 gene product from transcrip	17.4067244	-0.394615408	0.897805725	17.4067244	-0.226989535	0.922400604	17.4067244	-0.400837525	0.676699921
FBgn0046756	putative noncoding RNA 017:3R	5.099350376	0.326793055	0.938791798	5.099350376	0.559343577		5.099350376	0.427769208	0.632529721
FBgn0047000	putative noncoding RNA 008:3L	75.97707287	0.473114163	0.649039738	75.97707287	0.196648988	0.913631634	75.97707287	-0.574033855	0.301582333
FBgn0083068	putative noncoding RNA 011:3L	64.56696076	-0.746049921	0.440031726	64.56696076	-0.664335817	0.523007999	64.56696076	0.481866436	0.565063875
FBgn0063083	putative noncoding RNA 015:3L	468.260642	-0.324532473	0.874352494	468.260642	-0.509922005	0.555968073	468.260642	0.227750651	0.778198379
FBgn0029153	Malic enzyme like-2	0			0			0		
FBgn0029154	Malic enzyme like-1	0			0			0		
FBgn0004507	Glycogen phosphorylase	34070.9036	0.137755328	0.991146172	34070.9036	0.478881933	0.652680566	34070.9036	-0.906520573	0.08561358
FBgn0053629	pseudo	0			0			0		
FBgn0053632	CG33632 gene product from transcrip	0.116471877	-0.071974357		0.116471877	-0.068468588		0.116471877	0	
FBgn0053721	CG33721 gene product from transcrip	0			0			0		
FBgn0040606	CG6503 gene product from transcript	128.3538834	0.35416346	0.918245666	128.3538834	-0.015798065	0.997354109	128.3538834	-0.531825245	0.511998096
FBgn0066084	Ribosomal protein L41	94630.16298	-0.21598344	0.971762817	94630.16298	-0.087035366	0.975048554	94630.16298	-0.371974903	0.633645925
FBgn0083167	CG15304 gene product from transcrip	4524.617526	0.229312045	0.949623474	4524.617526	0.510503646	0.487617023	4524.617526	-0.974900105	0.015322657
FBgn0031414	eyes shut	94.75443531	-0.006587015	0.998322595	94.75443531	-0.121981308	0.942477801	94.75443531	0.032219165	0.973661639
FBgn0034903	CG9850 gene product from transcript	6633.9516	0.270781243	0.965076657	6633.9516	-0.426395522	0.782634313	6633.9516	0.715417247	0.328692798
FBgn0001297	kayak	5002.225309	0.024004069	0.996542676	5002.225309	0.055272007	0.963914828	5002.225309	-0.064973373	0.902881685
FBgn0030117	pseudo	0.056320698	0		0.056320698	0		0.056320698	0	
[illegible]	[illegible]	[illegible]	[illegible]	[illegible]	[illegible]	[illegible]	[illegible]	[illegible]	[illegible]	[illegible]

FBgn0054015	CG34015 gene product from transcrip	109.0998082	-0.186973838	0.975999817	109.0998082	0.150972725	0.941360428	109.0998082
FBgn0054021	CG34021 gene product from transcrip	0.828403015	0.050275071		0.828403015	-0.015629888		0.828403015
FBgn0054024	ncRNA	0.056320698	0		0.056320698	0		0.056320698
FBgn0054025	CG34025 gene product from transcrip	0.843019889	0.036916706		0.843019889	0.109385242		0.843019889
FBgn0054026	CG34026 gene product from transcrip	535.5015291	-0.985769641	0.126645433	535.5015291	-1.076105611	0.079017755	535.5015291
FBgn0054027	CG34027 gene product from transcrip	2.843647778	0.390141938	0.851740804	2.843647778	0.206449579		2.843647778
FBgn0054028	CG34028 gene product from transcrip	0.543513016	0.028042429		0.543513016	-0.023040314		0.543513016
FBgn0054029	CG34029 gene product from transcrip	0.720479924	0.199628844		0.720479924	0		0.720479924
FBgn0054030	CG34030 gene product from transcrip	0.302908743	0.007945117		0.302908743	-0.035967726		0.302908743
FBgn0054031	CG34031 gene product from transcrip	24.14629708	-0.213439399	0.979231897	24.14629708	-0.046095662	0.991444605	24.14629708
FBgn0054033	CG34033 gene product from transcrip	0.07379547	0.108851729		0.07379547	0		0.07379547
FBgn0054034	CG34034 gene product from transcrip	40.46255683	-1.039313452	0.127291124	40.46255683	-0.373095162	0.810946239	40.46255683
FBgn0038156	CG14372 gene product from transcrip	45.87097328	0.191551674	0.979231897	45.87097328	0.141726175	0.951701663	45.87097328
FBgn0053635	CG33635 gene product from transcrip	143.6668591	-0.182987894	0.953305045	143.6668591	-0.101355309	0.948432738	143.6668591
FBgn0053465	CG33465 gene product from transcrip	344.2215742	0.047651997	0.995584438	344.2215742	0.409943613	0.621010334	344.2215742
FBgn0053798	CG33798 gene product from transcrip	0.100708642	0		0.100708642	0		0.100708642
FBgn0054038	CG34038 gene product from transcrip	71.41661037	0.019884205	0.998322595	71.41661037	0.124969204	0.959986388	71.41661037
FBgn0054039	CG34039 gene product from transcrip	163.1938003	-0.196484607	0.91488891	163.1938003	-0.273749619	0.708643066	163.1938003
FBgn0054040	CG34040 gene product from transcrip	0.120302357	-0.141479015		0.120302357	-0.1379702		0.120302357
FBgn0054041	CG34041 gene product from transcrip	0.203940171	0		0.203940171	0		0.203940171
FBgn0065110	pickpocket 10	6.455021834	0.26344606	0.969626359	6.455021834	-0.536598733	0.683435231	6.455021834
FBgn0054043	CG34043 gene product from transcrip	210.9100183	-0.392288594	0.836815114	210.9100183	0.112370041	0.964908204	210.9100183
FBgn0054044	ncRNA	9.193168714	-0.034412312	0.998322595	9.193168714	0.186467806	0.944894567	9.193168714
FBgn0054045	CG34045 gene product from transcrip	51.40689956	0.880368048	0.152110799	51.40689956	0.930975068	0.118987803	51.40689956
FBgn0054046	ncRNA	2.399409649	-0.17730645	0.979231897	2.399409649	0.010706945		2.399409649
FBgn0054047	ncRNA	0.07379547	0.108851729		0.07379547	0		0.07379547
FBgn0054049	CG34049 gene product from transcrip	0			0			0
FBgn0054051	CG34051 gene product from transcrip	1.109899157	-0.009790929		1.109899157	0.085738072		1.109899157
FBgn0054052	ncRNA	1.552238304	-0.005150482		1.552238304	-0.026782903		1.552238304
FBgn0054054	CG34054 gene product from transcrip	27.71186558	1.146255476	0.112312671	27.71186558	0.829675825	0.380866247	27.71186558
FBgn0065108	pickpocket 16	0			0			0
FBgn0065109	pickpocket 11	0			0			0
FBgn0054056	CG34056 gene product from transcrip	1073.603692	0.377826741	0.46210274	1073.603692	0.549703454	0.130315824	1073.603692
FBgn0054057	CG34057 gene product from transcrip	22.68691695	-0.682121022	0.52830041	22.68691695	-0.254601962	0.908103495	22.68691695
FBgn0260453	CG17140 gene product from transcrip	0			0			0
FBgn0260454	CG17139 gene product from transcrip	0			0			0
FBgn0052529	Histone gene-specific Epigenetic Repres	1349.326063	0.30409843	0.768250233	1349.326063	0.263831033	0.770169252	1349.326063
FBgn0013696	tRNA	39.96052014	-0.560330988	0.746362007	39.96052014	-0.390928001	0.826129098	39.96052014
FBgn0013703	tRNA	6.610706824	0.038299617	0.998322595	6.610706824	0.631934052	0.592909786	6.610706824
FBgn0013700	tRNA	0.324706023	0		0.324706023	0.090037752		0.324706023
FBgn0013680	NADH dehydrogenase subunit 2	5744.24069	0.134734419	0.993466746	5744.24069	1.803526207	0.000285651	5744.24069
FBgn0013709	tRNA	0.89031395	-0.115676786		0.89031395	0.020651627		0.89031395
FBgn0013690	tRNA	85.3431444	-0.131997247	0.991146172	85.3431444	0.122140187	0.955295577	85.3431444
FBgn0013710	tRNA	21.82508439	0.108254495	0.994497358	21.82508439	0.986563207	0.228054347	21.82508439
FBgn0013674	cytochrome c oxidase subunit I	241944.683	-0.001875662	0.999629143	241944.683	0.666658321	0.471717511	241944.683
FBgn0013699	tRNA	1.399257625	-0.049369384		1.399257625	0.465096327		1.399257625
FBgn0013675	cytochrome c oxidase subunit II	64679.88081	-0.138797859	0.989856236	64679.88081	0.759918744	0.246480896	64679.88081
FBgn0013697	tRNA	3.401608989	-0.110526309	0.99157593	3.401608989	0.102675581		3.401608989
FBgn0013691	tRNA	0.729021653	-0.124674725		0.729021653	-0.01072455		0.729021653
FBgn0013673	ATP synthase F0 subunit 8	9005.12462	0.170578454	0.989856236	9005.12462	2.457520447	1.09E-06	9005.12462
FBgn0013672	ATP synthase F0 subunit 6	41098.90911	-0.084820073	0.994497358	41098.90911	1.369394132	0.005211602	41098.90911
FBgn0013676	cytochrome c oxidase subunit III	109716.0169	0.14395829	0.989483958	109716.0169	0.878585662	0.154833987	109716.0169
FBgn0013694	tRNA	1.125847324	-0.108168641		1.125847324	0.128989393		1.125847324
FBgn0013681	NADH dehydrogenase subunit 3	4370.640083	-0.03378461	0.99723561	4370.640083	1.159397586	0.000956006	4370.640083

[illegible]

FBgn0085222	CG34193 gene product from transcrip	566.1974754	-0.244757614	0.956414448	566.1974754	-0.539689862	0.561930586
FBgn0085223	CG34194 gene product from transcrip	97.17714097	-0.510998267	0.727537036	97.17714097	-0.359476985	0.815824306
FBgn0085224	CG34195 gene product from transcrip	95.61867466	1.135659326	0.008608878	95.61867466	1.03420545	0.027950351
FBgn0085225	CG34196 gene product from transcrip	0.899574211	-0.156788758		0.899574211	-0.150106813	
FBgn0085227	CG34198 gene product from transcrip	149.9892239	-0.206284757	0.961649406	149.9892239	-0.011840901	0.997344995
FBgn0085228	CG34199 gene product from transcrip	1.87943275	-0.327777713	0.840842244	1.87943275	-0.281838221	
FBgn0085229	CG34200 gene product from transcrip	815.3067078	0.267959468	0.865273684	815.3067078	0.465464541	0.462332962
FBgn0085230	CG34201 gene product from transcrip	0.790274547	0.010825681		0.790274547	0.066134601	
FBgn0085231	CG34202 gene product from transcrip	3.120827398	-0.002927179	0.999629143	3.120827398	0.355124245	
FBgn0085232	CG34203 gene product from transcrip	3.225076277	0.058545904	0.995120986	3.225076277	-0.48194853	
FBgn0085233	CG34204 gene product from transcrip	0			0		
FBgn0085234	CG34205 gene product from transcrip	0.306739223	-0.028913986		0.306739223	-0.072246796	
FBgn0085235	CG34206 gene product from transcrip	143.7943557	-0.302693219	0.898912603	143.7943557	-0.491811588	0.589051643
FBgn0085236	CG34207 gene product from transcrip	7.483106408	0.272080707	0.963501097	7.483106408	0.33203438	0.850061293
FBgn0085237	CG34208 gene product from transcrip	0.488124445	0.048824009		0.488124445	0.144138265	
FBgn0085239	CG34210 gene product from transcrip	0.394214697	0.149665514		0.394214697	0.052812563	
FBgn0085240	CG34211 gene product from transcrip	0			0		
FBgn0085241	CG34212 gene product from transcrip	0.099032819	0		0.099032819	0.123176119	
FBgn0085242	CG34213 gene product from transcrip	119.1864681	-0.094778228	0.993127879	119.1864681	-0.19124371	0.884079416
FBgn0085243	CG34214 gene product from transcrip	1.700808629	0.06004617	0.994876798	1.700808629	0.343221929	
FBgn0085244	CG34215 gene product from transcrip	522.2254697	4.293895641	7.26E-38	522.2254697	3.56529204	2.87E-25
FBgn0085245	CG34216 gene product from transcrip	2.388335276	0.249067224	0.954378726	2.388335276	0.111390608	
FBgn0085246	CG34217 gene product from transcrip	0.429217369	-0.061126971		0.429217369	-0.059355243	
FBgn0085248	CG34219 gene product from transcrip	1.079244196	-0.121147513		1.079244196	-0.070947731	
FBgn0085249	CG34220 gene product from transcrip	103.9268109	0.298495829	0.961435662	103.9268109	0.014930299	0.998619505
FBgn0085250	CG34221 gene product from transcrip	5.505698315	-0.330206801	0.917248088	5.505698315	-0.060883431	
FBgn0085252	CG34223 gene product from transcrip	0.770369283	0.291066633		0.770369283	0	
FBgn0085253	CG34224 gene product from transcrip	0			0		
FBgn0085254	CG34225 gene product from transcrip	3.193008235	0.602965455	0.619579859	3.193008235	0.231205798	
FBgn0085256	CG34227 gene product from transcrip	160022.8376	0.134796422	0.988354972	160022.8376	0.097122239	0.966326291
FBgn0085257	CG34228 gene product from transcrip	252.2317618	0.247207481	0.956325514	252.2317618	0.252889008	0.878470454
FBgn0085259	CG34230 gene product from transcrip	0.448382105	0.173174747		0.448382105	0.066984067	
FBgn0085260	CG34231 gene product from transcrip	10.44980136	0.153958581	0.993127879	10.44980136	0.264693309	0.911006433
FBgn0085261	CG34232 gene product from transcrip	1418.126453	-0.041856675	0.995584438	1418.126453	-0.52493815	0.426812551
FBgn0085263	CG34234 gene product from transcrip	0.456976849	0.010839351		0.456976849	0.126442571	
FBgn0085264	CG34235 gene product from transcrip	0			0		
FBgn0085265	CG34236 gene product from transcrip	72.61096649	-0.39167056	0.782401957	72.61096649	-0.569585206	0.480471243
FBgn0085267	CG34238 gene product from transcrip	0.293028587	0.182888499		0.293028587	0	
FBgn0085271	CG34242 gene product from transcrip	311.3651254	-0.023771665	0.998322595	311.3651254	0.133841941	0.935991255
FBgn0085273	CG34244 gene product from transcrip	501.9662563	-0.441118145	0.638948409	501.9662563	-0.433205414	0.60377373
FBgn0085274	CG34245 gene product from transcrip	1.502428858	-0.017988821		1.502428858	-0.025029517	
FBgn0085276	CG34247 gene product from transcrip	4.507988165	0.132422639	0.991865251	4.507988165	0.18009152	
FBgn0085277	CG34248 gene product from transcrip	2.378463842	-0.23322847	0.963827624	2.378463842	-0.021403037	
FBgn0085279	CG34250 gene product from transcrip	142.0630067	0.17760607	0.977857422	142.0630067	0.026389516	0.994998875

[illegible]

FBgn0086679	pink	1367.739483	-0.156959785
FBgn0086680	ventral veins lacking	1636.98324	0.57892992
FBgn0086681	Male-specific transcript 36Fa	27.82380898	-0.308488778
FBgn0086683	CG17540 gene product from transcrip	1574.720816	-0.119994961
FBgn0085407	PDGF- and VEGF-related factor 3	216.8876641	0.34464104
FBgn0086686	lethal (3) L1231	1696.920424	0.112808285
FBgn0086687	Desaturase 1	21560.55236	0.214277294
FBgn0086690	CG33957 gene product from transcrip	1771.650388	0.141095205
FBgn0086691	CG15261 gene product from transcrip	4154.250861	0.451465251
FBgn0086693	inactive	36.74267635	0.017265268
FBgn0086694	CG10542 gene product from transcrip	1419.902886	0.089424172
FBgn0086695	humpty dumpty	172.4058507	-0.208218206
FBgn0086698	fritz	342.8234358	-0.295156172
FBgn0086704	slow termination of phototransductior	87.37192008	-0.064954473
FBgn0086706	pixie	9141.38828	-0.045843162
FBgn0086707	nucampholin	2064.330829	0.068053409
FBgn0086708	starvin	2380.733484	0.47032105
FBgn0086710	Ribosomal protein L30	16894.04277	-0.002958271
FBgn0086711	moladietz	396.4104003	0.087090552
FBgn0086712	Enigma	750.141875	0.040467462
FBgn0086757	centrosomin's beautiful sister	795.3536714	0.199816973
FBgn0086758	Chronologically inappropriate morphog	1344.643765	0.368870543
FBgn0086768	Protein-L-isoaspartate (D-aspartate) O-	1809.187678	-0.148001887
FBgn0086778	nicotinic Acetylcholine Receptor alpha7	125.373534	0.206179509
FBgn0086779	steppke	1137.113833	-0.130928956
FBgn0086782	amnesiac	21.21132573	0.344381122
FBgn0086784	stambha A	1001.614069	-0.14798947
FBgn0086785	Vacuolar protein sorting 36	669.8382047	-0.168584401
FBgn0086855	CG17078 gene product from transcrip	553.0847791	-0.104852222
FBgn0086856	CG11555 gene product from transcrip	280.8378957	-0.034481792
FBgn0086895	peanuts	537.7427767	0.00712205
FBgn0086898	diego	412.9879937	-0.015439055
FBgn0086900	Cuticular protein 65Ax1	0	
FBgn0086901	crossveinless c	2895.606936	0.193509818
FBgn0086904	Nascent polypeptide associated compl	25708.81776	0.1765914
FBgn0086906	sallimus	13381.63343	0.535442637
FBgn0086907	Cytochrome c distal	8.324920967	0.910304812
FBgn0086908	eggless	987.6722636	0.072075639
FBgn0086909	CG31751 gene product from transcrip	2233.295094	0.074281885
FBgn0086912	mushroom body miniature	701.8337481	0.030180232
FBgn0086913	CG34410 gene product from transcrip	85.02635845	-0.055930101
FBgn0086915	Male-specific transcript 77F	12.53731328	0.722312786
FBgn0086916	sneaky	0.794275394	0.203667065
FBgn0086917	spookier	310.2841896	-0.138131928
FBgn0087002	Retinoid- and fatty acid-binding glycop	106028.2718	0.614640153
FBgn0087005	retinophilin	12.37335743	-0.046628677
FBgn0087007	big bang	2930.694128	-0.153882818
FBgn0087008	enhancer of yellow 3	2244.598496	0.424901452
FBgn0087011	CG41520 gene product from transcrip	133.2636387	0.130923908
FBgn0087012	5-hydroxytryptamine (serotonin) recep	33.58890714	-0.043445541
FBgn0087013	Karyopherin beta 3	12665.05532	-0.233267534
FBgn0087021	CG7242 gene product from transcript	363.8177779	-0.002898605
FBgn0087035	Argonaute 2	4756.502566	-5.018405445
FBgn0087039	SECIS-binding protein 2	538.732746	-0.378861525
FBgn0087040	alpha-Tubulin at 67C	8.157611088	0.113514142
FBgn0087041	CG42231 gene product from transcrip	0	
FBgn0243486	reduced ocelli	299.7035115	0.328336197
FBgn0243511	phagocyte signaling impaired	1289.353412	0.072219677
FBgn0243512	puckered	1261.352851	-0.008321525
FBgn0243513	cornichon related	91.03046393	0.0058305
FBgn0243514	CG6124 gene product from transcript	694.1500711	0.507500722
FBgn0243516	Verprolin 1	1070.218935	-0.072267844
FBgn0243586	Tubby	1.345115036	0.199298597
FBgn0250731	5.8S ribosomal RNA	0	
FBgn0250732	GST-containing FLYWCH zinc-finger prc	1232.240515	0.276890957
FBgn0250746	Proteasome beta7 subunit	11068.27951	-0.06136843
FBgn0250753	krasavietz	11280.18947	0.027035382
FBgn0250754	CG42232 gene product from transcrip	1278.140737	0.291281592
FBgn0250755	CG42233 gene product from transcrip	201.8526841	-0.030824922
FBgn0250757	CG42235 gene product from transcrip	9420.874996	0.136778465
FBgn0250785	varicose	2522.890972	0.056772542
FBgn0250786	Chromodomain-helicase-DNA-binding	2160.078822	0.137302617
FBgn0250788	beta Spectrin	8069.023751	0.024251389
FBgn0250789	alpha Spectrin	6454.572121	0.160966481
FBgn0250791	alpha Soluble NSF attachment protein	2586.440381	-0.218364111
FBgn0250814	CG4169 gene product from transcript	10087.34442	0.02288623
FBgn0250815	Jonah 65Aiv	89088.13513	-2.004546646
FBgn0250816	Argonaute 3	43.3302094	-0.434809653
FBgn0250817	ncRNA	0	
FBgn0250819	CG33521 gene product from transcrip	3755.70333	0.201037651
FBgn0250820	medial glomeruli	1256.884983	0.127706522
FBgn0250821	CG14644 gene product from transcrip	7.552405017	0.426450603
FBgn0250822	CG34222 gene product from transcrip	2.162956834	0.511759915
FBgn0250823	gilgamesh	2333.402962	-0.118301526
FBgn0250824	CG33655 gene product from transcrip	27.28469605	-0.493965808
FBgn0250825	CG34241 gene product from transcrip	6.88299028	0.623718595
FBgn0250826	CG34160 gene product from transcrip	1.54084076	0.412096962
FBgn0250827	whipple	3.952116952	0.554463912
FBgn0250830	CG12547 gene product from transcrip	991.0562913	-0.023701779
FBgn0250831	CG34103 gene product from transcrip	0	
FBgn0250832	Ductus ejaculatorius peptide 99B	0.219335176	-0.098996051
FBgn0250833	CG34461 gene product from transcrip	1.262943638	0.084016193
FBgn0250834	CG33308 gene product from transcrip	1.014315854	0.273483753
FBgn0250835	CG15394 gene product from transcrip	18.04534022	0.340183774
FBgn0250836	CG8628 gene product from transcript	5385.310833	-0.217581176
FBgn0250837	Deoxyuridine triphosphatase	0	
FBgn0250838	CG30415 gene product from transcrip	7135.341793	0.45584529
FBgn0250839	CG2016 gene product from transcript	798.8508131	0.056497675
FBgn0250841	CG17242 gene product from transcrip	0	
FBgn0250842	CG17575 gene product from transcrip	0.47293639	-0.138570365
FBgn0250843	Proteasome alpha6 subunit	5240.28527	-0.210929345
FBgn0250844	CG4218 gene product from transcript	3.527786986	0.786700537
FBgn0250845	CG1288 gene product from transcript	4.768172608	0.762572141
FBgn0250846	globin 2	2.351295264	0.873390107
FBgn0250847	CG14034 gene product from transcrip	0.422634684	0.046875793
FBgn0250848	26-29kD-proteinase	9196.705524	0.214034259
FBgn0250849	CG32388 gene product from transcrip	0	
FBgn0250850	rigor mortis	691.5900576	-0.087926234
FBgn0250851	CG33981 gene product from transcrip	0	
FBgn0250862	CG42237 gene product from transcrip	43.16485736	0.437730263
FBgn0250867	CG42238 gene product from transcrip	487.2395732	-0.138827991
FBgn0250868	CG42239 gene product from transcrip	1089.793277	-0.017221201
FBgn0250869	CG42240 gene product from transcrip	1283.853271	0.398252956
FBgn0250871	papillote	3670.006283	0.035101865
FBgn0250874	tiny tim 50	1195.116814	0.129531775
FBgn0250876	Semaphorin-5c	743.8308671	-0.207857707
FBgn0250903	lemming A	0	
FBgn0250904	CG34434 gene product from transcrip	19.84195598	1.571401066
FBgn0250906	Phosphoglycerate kinase	14985.69419	0.084291733
FBgn0250907	Chitinase 3	11637.07475	-0.355686557
FBgn0250908	CG42242 gene product from transcrip	58.40267999	0.065555126
FBgn0250910	Octopamine beta3 receptor	42.7009658	0.473504149
FBgn0250911	CG42245 gene product from transcrip	0	
FBgn0259098	CG42246 gene product from transcrip	59.69579244	0.067526406
FBgn0259099	Doublecortin-domain-containing echin	23.73811613	0.16067865
FBgn0259101	CG42249 gene product from transcrip	1769.632235	-0.881883239
FBgn0259108	CG34387 gene product from transcrip	452.3900087	0.519010996
FBgn0259110	mind-meld	267.8666716	0.281829288
FBgn0259111	Na[+]-driven anion exchanger 1	2602.520852	0.319545823
FBgn0259112	CG42254 gene product from transcrip	36.41573982	-0.113065348
FBgn0259113	DNA polymerase alpha 180kD	443.0302634	0.135082777
FBgn0259139	glorund	8032.065264	0.168649642
FBgn0259140	CG42255 gene product from transcrip	525.0110716	-0.614468934
FBgn0259143	CG42258 gene product from transcrip	2956.650355	0.135432624
FBgn0259145	CG42260 gene product from transcrip	84.7221286	0.216317275
FBgn0259146	CG42261 gene product from transcrip	56.58546879	0.03571364
FBgn0259148	CG42263 gene product from transcrip	1.839603159	0.264179876
FBgn0259149	CG42264 gene product from transcrip	36.27764845	-0.682480408
FBgn0259150	CG42265 gene product from transcrip	153.0965294	0.173954827
FBgn0259151	CG42266 gene product from transcrip	12.4779537	0.042332046
FBgn0259152	Caliban	1263.708769	-0.083340897
FBgn0259162	CG42267 gene product from transcrip	13.87131811	0.17271479
FBgn0259163	CG42268 gene product from transcrip	1119.851749	-0.133126908
FBgn0259164	CG42269 gene product from transcrip	306.3050272	-0.819400177
FBgn0259166	CG42271 gene product from transcrip	1009.185902	0.03641446
FBgn0259167	CG42272 gene product from transcrip	68.59416432	-0.423626688
FBgn0259168	minibrain	2214.156966	0.012902353
FBgn0259170	alpha Mannosidase I	913.1645741	0.229670786
FBgn0259171	Phosphodiesterase 9	1205.800597	0.514779286
FBgn0259173	cornetto	2331.186204	-0.321740484
FBgn0259174	CG42279 gene product from transcrip	2201.300018	-0.25629436
FBgn0259175	omega	1568.554726	-0.213817017
FBgn0259176	bunched	6978.811952	0.099372788
FBgn0259178	CG42283 gene product from transcrip	214.4250784	-0.082801558
FBgn0259179	CG42284 gene product from transcrip	0.451835808	-0.02351098
FBgn0259183	CG42287 gene product from transcrip	2.95035182	0.170005675
FBgn0259184	CG42288 gene product from transcrip	9.869556714	1.10569687
FBgn0259185	Ionotropic receptor 60b	0.597705933	0.073803582
FBgn0259186	Ionotropic receptor 60c	0.515806902	-0.081278239
FBgn0259187	Ionotropic receptor 60d	0.07379547	0.108851729
FBgn0259188	Ionotropic receptor 7f	2.035990301	0.006573858
FBgn0259189	Ionotropic receptor 7e	0.07379547	0.108851729
FBgn0259190	Ionotropic receptor 7d	0.240127923	-0.093439558
FBgn0259192	CG42296 gene product from transcrip	21.3639975	0.284426251
FBgn0259193	Ionotropic receptor 94d	0.69714526	0.084358692
FBgn0259194	Ionotropic receptor 94e	0.607321659	-0.067745833
FBgn0259195	CG42299 gene product from transcrip	2.72468167	0.073811853
FBgn0259196	CG42300 gene product from transcrip	2.459184641	0.343063723
FBgn0259198	CG42302 gene product from transcrip	0.174504111	0.073112477
FBgn0259199	Snurportin	0	
FBgn0259200	CG42304 gene product from transcrip	0	
FBgn0259201	CG42305 gene product from transcrip	0	
FBgn0259202	CG42306 gene product from transcrip	0	
FBgn0259203	CG42307 gene product from transcrip	0.213214751	-0.032560156
FBgn0259204	CG42308 gene product from transcrip	0	
FBgn0259209	Muscle LIM protein at 60A	16382.62188	0.132111835
FBgn0259210	prominin	17.02566857	0.612792517
FBgn0259211	grainy head	2414.616594	0.110356051
FBgn0259212	canoe	1815.402522	0.009407478
FBgn0259213	CG42313 gene product from transcrip	73.0193774	0.235056248
FBgn0259214	plasma membrane calcium ATPase	6319.651486	-0.191036903
FBgn0259215	Ionotropic receptor 93a	6.614631022	0.08063666
FBgn0259216	Rho GTPase activating protein at 102A	725.6409514	0.183526048
FBgn0259219	CG42319 gene product from transcrip	1896.322967	-0.027962849
FBgn0259221	CG42321 gene product from transcrip	1483.376973	-0.031093162
FBgn0259222	CG42322 gene product from transcrip	82.52591186	0.217515451
FBgn0259223	CG42323 gene product from transcrip	6.620203571	-0.126406784
FBgn0259224	CG42324 gene product from transcrip	874.026201	0.422948923
FBgn0259226	CG42326 gene product from transcrip	93.2963164	0.309057472
FBgn0259227	CG42327 gene product from transcrip	1518.339582	-0.245601601
FBgn0259228	C3G ortholog (H. sapiens)	1335.008997	0.297926167
FBgn0259229	CG42329 gene product from transcrip	567.4672958	-0.232649026
FBgn0259230	CG42295 gene product from transcrip	545.7720056	0.606259193
FBgn0259231	Cholecystokinin-like receptor at 17D1	11.27333053	-0.00832902
FBgn0259233	CG42331 gene product from transcrip	28.26493196	0.177753855
FBgn0259234	Calmodulin-binding transcription activ	376.9268691	-0.105117834
FBgn0259236	CG42334 gene product from transcrip	919.8563972	0.060072901
FBgn0259237	CG42335 gene product from transcrip	160.6682852	-0.630751527
FBgn0259238	CG42336 gene product from transcrip	5911.244392	0.11615427
FBgn0259239	CG42337 gene product from transcrip	112.9971209	-0.033738624
FBgn0259241	CG42339 gene product from transcrip	70.84229115	0.057086128
FBgn0259242	CG42340 gene product from transcrip	116.0046861	0.063787275
FBgn0259243	cAMP-dependent protein kinase R1	9026.995583	0.070415256
FBgn0259244	CG42342 gene product from transcrip	1474.024905	0.440817777
FBgn0259245	CG42343 gene product from transcrip	45.9372821	-0.267376731
FBgn0259246	bruchpilot	326.7638542	0.175526221
FBgn0259247	laccase 2	1241.081825	0.215864995
FBgn0259481	CG11711 gene product from transcrip	3416.61484	0.05453598
FBgn0259482	CG4946 gene product from transcript	245.6175146	0.061554738
FBgn0259483	CG3403 gene product from transcript	2243.970589	-0.255187714
FBgn0259676	DNA polymerase alpha 60kD	209.8317781	-0.173197689
FBgn0259677	CG42346 gene product from transcrip	58.15854091	0.058433116
FBgn0259678	spaghetti-squash activator	1863.172765	-0.304205117
FBgn0259680	Protein kinase C delta	409.1019888	0.459734409
FBgn0259682	CG42351 gene product from transcrip	6312.348877	0.000967455
FBgn0259683	Ionotropic receptor 40a	15.33471101	0.835688566
FBgn0259685	crumbs	1464.083692	-0.094843551
FBgn0259697	neverland	76.39188806	-0.02858503
FBgn0259699	CG42353 gene product from transcrip	0	
FBgn0259700	CG42354 gene product from transcrip	13.4866176	-0.460998173
FBgn0259701	CG42355 gene product from transcrip	1.499323324	0.620160773
FBgn0259702	CG42356 gene product from transcrip	0	
FBgn0259703	CG42357 gene product from transcrip	0.072449522	0
FBgn0259704	CG42358 gene product from transcrip	290.9672479	-0.153306672
FBgn0259705	CG42359 gene product from transcrip	207.6760505	-0.490351762
FBgn0259707	CG42361 gene product from transcrip	111.9538186	-0.032380104
FBgn0259708	CG42362 gene product from transcrip	0	
FBgn0259709	CG42363 gene product from transcrip	0	
FBgn0259710	CG42364 gene product from transcrip	2.324085769	-0.132759804
FBgn0259711	CG42365 gene product from transcrip	184.6131505	0.707964477
FBgn0259712	CG42366 gene product from transcrip	6.14226616	0.324362313
FBgn0259713	CG42367 gene product from transcrip	0.413284369	0
FBgn0259714	CG42368 gene product from transcrip	171.1185042	-0.450905622
FBgn0259715	CG42369 gene product from transcrip	278.708917	0.225398291
FBgn0259716	CG42370 gene product from transcrip	4461.700419	0.38451766
FBgn0259717	CG42371 gene product from transcrip	0	
FBgn0259718	CG42372 gene product from transcrip	0	
FBgn0259720	CG42374 gene product from transcrip	0	
FBgn0259721	CG42375 gene product from transcrip	0	
FBgn0259722	CG42376 gene product from transcrip	474.8403602	0.112573105
FBgn0259725	CG42379 gene product from transcrip	0	
FBgn0259726	CG42380 gene product from transcrip	0	
FBgn0259727	CG42381 gene product from transcrip	0	
FBgn0259728	CG42382 gene product from transcrip	0.581499656	0.054510278
FBgn0259729	CG42383 gene product from transcrip	1.312788793	0.303538423
FBgn0259730	CG42384 gene product from transcrip	0	
FBgn0259731	CG42385 gene product from transcrip	0	
FBgn0259732	CG42386 gene product from transcrip	0	
FBgn0259733	CG42387 gene product from transcrip	0	
FBgn0259734	CG42388 gene product from transcrip	1283.353615	0.145703978
FBgn0259735	CG42389 gene product from transcrip	1983.64738	0.385366634
FBgn0259736	CG42390 gene product from transcrip	3208.77356	-0.144080928
FBgn0259737	CG42391 gene product from transcrip	4.050509583	0.364019289
FBgn0259738	CG42392 gene product from transcrip	1.894115088	-0.072631903
FBgn0259739	CG42393 gene product from transcrip	4.793329035	0.744461932
FBgn0259740	CG42394 gene product from transcrip	1314.121828	0.316874052
FBgn0259741	CG42395 gene product from transcrip	11.36511263	-0.138737659
FBgn0259742	CG42360 gene product from transcrip	231.9793633	-0.135491972
FBgn0259745	CG42396 gene product from transcrip	687.2913111	-0.026071507
FBgn0259748	CG42397 gene product from transcrip	3.876590092	-0.162346765
FBgn0259749	mummy	4877.428037	0.326467365
FBgn0259785	putzig	3444.866987	0.046894684
FBgn0259789	vielfaltig	1650.00055	0.341224894
FBgn0259791	aurora borealis	142.6689278	0.048937063
FBgn0259794	sina homologue	3.28017451	0.36577146
FBgn0259795	CG4750 gene product from transcript	23.13048944	0.675953486
FBgn0259818	CG42399 gene product from transcrip	87.28955874	0.094956037
FBgn0259821	CG42402 gene product from transcrip	81.41267851	-0.435033735
FBgn0259822	Ca2+-channel-protein-beta-subunit	307.3771749	0.704816669
FBgn0259823	CG42404 gene product from transcrip	127.030102	0.077691963
FBgn0259824	Huntingtin-interacting protein 14	1201.218231	0.098597047
FBgn0259831	CG34309 gene product from transcrip	2.637603546	0.058225814
FBgn0259832	CG34229 gene product from transcrip	188.4815317	0.220451233
FBgn0259834	outsiders	382.7591388	-0.38606515
FBgn0259838	ncRNA	0	
FBgn0259841	ncRNA	0	
FBgn0259843	ncRNA	0	
FBgn0259845	ncRNA	0.056320698	0
FBgn0259846	ncRNA	0	
FBgn0259847	ncRNA	0	
FBgn0259848	ncRNA	0.056320698	0
FBgn0259850	ncRNA	0.07379547	0.108851729
FBgn0259851	ncRNA	0	
FBgn0259852	ncRNA	0	
FBgn0259853	ncRNA	0	
FBgn0259855	ncRNA	0	
FBgn0259856	ncRNA	0.07379547	0.108851729
FBgn0259857	ncRNA	0	
FBgn0259858	ncRNA	0	
FBgn0259859	ncRNA	0	
FBgn0259860	ncRNA	0	
FBgn0259861	ncRNA	0.221386409	0.173816599
FBgn0259862	ncRNA	0	
FBgn0259863	ncRNA	0.147590939	0.153120924
FBgn0259869	ncRNA	0	
FBgn0259875	ncRNA	0	
FBgn0259876	CG34438 gene product from transcrip	1070.591436	-0.031128859
FBgn0259878	Follistatin	85.44180464	-0.015285987
FBgn0259896	Nimrod C1	672.8255336	0.739674949
FBgn0259916	CG42445 gene product from transcrip	400.3934297	0.27500314
FBgn0259917	CG42446 gene product from transcrip	0.735801404	0.231823787
FBgn0259918	CG42447 gene product from transcrip	0	
FBgn0259922	CG42448 gene product from transcrip	0.610449501	0.158393272
FBgn0259923	Septin 4	439.3634756	-0.135203423
FBgn0259926	CG42449 gene product from transcrip	2.1602564	-0.001956586
FBgn0259927	CG42450 gene product from transcrip	171.4959106	0.11084764
FBgn0259932	CG42455 gene product from transcrip	0	
FBgn0259933	CG42456 gene product from transcrip	0	
FBgn0259935	CG42458 gene product from transcrip	77.89644225	0.239396732
FBgn0259936	ncRNA	84.46800559	-0.195846797
FBgn0259937	Nucleolar protein at 60B	3191.25521	0.1661167
FBgn0259938	clockwork orange	1676.181473	0.092384212
FBgn0259949	Seminal fluid protein 23F	7.505116375	-0.03125893
FBgn0259950	CG42460 gene product from transcrip	3846.74767	0.724341899
FBgn0259951	Seminal fluid protein 24Ba	0.133946648	0.011640396
FBgn0259952	Seminal fluid protein 24Bb	0	
FBgn0259953	CG42463 gene product from transcrip	0	
FBgn0259954	CG42464 gene product from transcrip	0.737096681	0.11805999
FBgn0259955	CG42465 gene product from transcrip	0.297098458	0
FBgn0259956	Seminal fluid protein 24C1	0.112641396	0
FBgn0259957	CG42467 gene product from transcrip	0.233309342	-0.048685468
FBgn0259958	Seminal fluid protein 24F	0.198065639	0
FBgn0259959	Seminal fluid protein 26Ac	0	
FBgn0259960	CG42470 gene product from transcrip	0	
FBgn0259961	CG42471 gene product from transcrip	0	
FBgn0259962	Seminal fluid protein 33A1	0.07379547	0.108851729
FBgn0259963	Seminal fluid protein 33A2	0.07379547	0.108851729
FBgn0259964	Seminal fluid protein 33A3	0.330528566	0.076114052
FBgn0259965	Seminal fluid protein 35C	0	
FBgn0259966	Seminal fluid protein 51E	0.418568307	0
FBgn0259967	Seminal fluid protein 53D	0.201417284	0
FBgn0259968	Seminal fluid protein 60F	0.147619473	0
FBgn0259969	Seminal fluid protein 65A	0.296925412	-0.150029014
FBgn0259970	Seminal fluid protein 70A4	0.060151179	-0.097832662
FBgn0259971	CG42481 gene product from transcrip	0.155353517	0
FBgn0259972	Seminal fluid protein 77F	0	
FBgn0259973	Seminal fluid protein 79	0.24614629	0.097291802
FBgn0259974	Seminal fluid protein 84E	2.265264391	-0.156750784
FBgn0259975	Seminal fluid protein 87B	0.133946648	0.011640396
FBgn0259977	Tyrosine decarboxylase 1	266.9482291	0.134433026
FBgn0259978	vulcan	1170.329017	0.141242358
FBgn0259979	CG17337 gene product from transcrip	6079.342917	0.123525843
FBgn0259982	lethal (2) 35Cc	206.0095847	-0.313349076
FBgn0259984	kuzbanian	2582.882997	0.009930997
FBgn0259985	Metallophosphoesterase	235.85655	-0.216161345
FBgn0259986	CG33545 gene product from transcrip	260.2407953	-0.037825105
FBgn0259989	CG42486 gene product from transcrip	0.456339271	0.00278308
FBgn0259990	CG42487 gene product from transcrip	0	
FBgn0259991	CG42488 gene product from transcrip	0	
FBgn0259992	CG42489 gene product from transcrip	455.7527708	0.097436854
FBgn0259993	ncRNA	630.2199443	-0.090271276
FBgn0259994	CG42492 gene product from transcrip	787.4557014	0.074291339
FBgn0259996	ncRNA	0.201417284	0
FBgn0259998	CG17571 gene product from transcrip	35758.96449	-2.206171685
FBgn0260000	CG17570 gene product from transcrip	210.4665898	-0.358742363
FBgn0260002	ncRNA	8.468276301	-0.777335751
FBgn0260003	Dystrophin	2045.399179	-0.30837528
FBgn0260004	Sensory neuron membrane protein 1	54.13836052	0.023646139
FBgn0260005	water witch	326.8188311	-0.159557712
FBgn0260006	drop dead	589.6786704	-0.23535427
FBgn0260007	CG42493 gene product from transcrip	0.568959566	0.031264029
FBgn0260008	Ubiquinol-cytochrome C reductase cor	2927.578082	0.261353862
FBgn0260011	Nimrod C4	0.439537791	0.039608793
FBgn0260012	CG17509 gene product from transcrip	1137.47715	-0.11219511
FBgn0260026	CG42494 gene product from transcrip	224.3682136	0.461901978
FBgn0260027	CG42495 gene product from transcrip	60.08715004	-0.103347117
FBgn0260049	flare	8657.092321	0.115067576
FBgn0260222	CG42496 gene product from transcrip	0	
FBgn0260223	CG42497 gene product from transcrip	0	
FBgn0260224	CG42498 gene product from transcrip	0	
FBgn0260225	pseudo	0	
FBgn0260226	CG42500 gene product from transcrip	75258.83683	-0.064563934
FBgn0260228	CG42502 gene product from transcrip	0	
FBgn0260229	CG42503 gene product from transcrip	0	
FBgn0260230	CG42504 gene product from transcrip	0	
FBgn0260231	CG42505 gene product from transcrip	0	
FBgn0260233	CG42507 gene product from transcrip	0	
FBgn0260234	CG42508 gene product from transcrip	0	
FBgn0260237	CG42511 gene product from transcrip	0	
FBgn0260238	CG42512 gene product from transcrip	0.056320698	0
FBgn0260243	Enhancer of variegation 3-9	862.9810072	-0.267379916
FBgn0260386	mind the gap	240.6116948	-0.199486015

FBgn0260388	CG42514 gene product from transcrip	610.9376788	0.098318391	0.991146172	610.9376788	0.223674734	0.827267758	610.9376788
FBgn0260390	CG42516 gene product from transcrip	0			0			0
FBgn0260392	CG42518 gene product from transcrip	0			0			0
FBgn0260393	CG17147 gene product from transcrip	589.6660736	-1.184444589	2.84E-05	589.6660736	-0.802539723	0.019188862	589.6660736
FBgn0260396	CG42521 gene product from transcrip	0.07379547	0.108851729		0.07379547	0		0.07379547
FBgn0260397	Suppressor of variegation 3-3	513.3546696	0.035813605	0.994876798	513.3546696	-0.106517247	0.916455459	513.3546696
FBgn0260398	PSEA-binding protein 49kD	0			0			0
FBgn0260399	greatwall	297.6786201	0.03484181	0.99564268	297.6786201	-0.011627001	0.997246866	297.6786201
FBgn0260400	embryonic lethal abnormal vision	1288.375822	0.031663167	0.998322595	1288.375822	0.351742145	0.805797107	1288.375822
FBgn0260401	Mediator complex subunit 9	0			0			0
FBgn0260407	mitochondrial ribosomal protein S23	307.0798383	0.182776027	0.963501097	307.0798383	0.345738979	0.687453403	307.0798383
FBgn0260428	CG42523 gene product from transcrip	2.630783112	0.49635808	0.376545848	2.630783112	-0.059068423		2.630783112
FBgn0260429	CG42524 gene product from transcrip	196.6521108	0.485303629	0.656359093	196.6521108	0.283994339	0.839587254	196.6521108
FBgn0260430	CG42525 gene product from transcrip	22.73734751	0.836026633	0.399353831	22.73734751	0.88564153	0.332530839	22.73734751
FBgn0260431	CG42526 gene product from transcrip	43.23703958	0.156032211	0.985645036	43.23703958	-0.255739604	0.874361514	43.23703958
FBgn0260434	CG42529 gene product from transcrip	0			0			0
FBgn0260435	pseudo	15.20144635	-1.599531509	0.006218823	15.20144635	-0.603067805	0.630904556	15.20144635
FBgn0260436	CG42531 gene product from transcrip	0			0			0
FBgn0260437	pseudo	0.684004978	-0.073174882		0.684004978	-0.21356962		0.684004978
FBgn0260486	Zizimin ortholog (H. sapiens)	531.8079645	0.085584065	0.994497358	531.8079645	-0.059125547	0.980691994	531.8079645
FBgn0260487	CG42534 gene product from transcrip	13.0324323	0.195095375	0.985645036	13.0324323	0.107606104	0.973807349	13.0324323
FBgn0260499	quiver	153.4193176	-0.126698988	0.988572405	153.4193176	0.124647175	0.94714921	153.4193176
FBgn0260632	dorsal	1295.837384	0.055082471	0.994497358	1295.837384	-0.028535706	0.986877529	1295.837384
FBgn0260634	eukaryotic translation initiation factor 4	1111.400927	0.224206382	0.956108275	1111.400927	0.313459384	0.796097411	1111.400927
FBgn0260635	thread	7082.282377	-0.31591914	0.820701281	7082.282377	-0.595825694	0.292131032	7082.282377
FBgn0260639	gamma-Tubulin at 23C	699.9209215	-0.110112684	0.972330876	699.9209215	-0.174324978	0.816599359	699.9209215
FBgn0260642	Antennapedia	710.0948093	0.09252958	0.985645036	710.0948093	0.218264004	0.764601941	710.0948093
FBgn0260643	CG42535 gene product from transcrip	0.07379547	0.108851729		0.07379547	0		0.07379547
FBgn0260644	CG42536 gene product from transcrip	0.221386409	0.173816599		0.221386409	0		0.221386409
FBgn0260645	CG42537 gene product from transcrip	0			0			0
FBgn0260646	CG42538 gene product from transcrip	1.00073038	0.076883911		1.00073038	0.008097313		1.00073038
FBgn0260648	CG31938 gene product from transcrip	395.7047096	0.018577417	0.998322595	395.7047096	-0.153234943	0.908976332	395.7047096
FBgn0260653	serpentine	9873.922238	0.586058484	0.235098752	9873.922238	1.01905229	0.003156697	9873.922238
FBgn0260655	lethal (3) 76BDm	663.2331064	-0.39799797	0.316901578	663.2331064	-0.183829698	0.803205896	663.2331064
FBgn0260657	CG42540 gene product from transcrip	92.44055027	-0.207710526	0.953941411	92.44055027	-0.217970297	0.869020803	92.44055027
FBgn0260658	CG42541 gene product from transcrip	78.44718657	0.503189514	0.714029775	78.44718657	0.222609299	0.914502763	78.44718657
FBgn0260659	CG42542 gene product from transcrip	1686.041447	0.199847152	0.88909528	1686.041447	-0.039752776	0.984061407	1686.041447
FBgn0260660	Multiplexin	1211.11983	0.251225845	0.861736263	1211.11983	-0.057018033	0.977209204	1211.11983
FBgn0260713	CG42544 gene product from transcrip	0.625848217	0.103850568		0.625848217	0.060884914		0.625848217
FBgn0260718	pseudo	0.130116168	0.082464995		0.130116168	0		0.130116168
FBgn0260719	pseudo	0			0			0
FBgn0260720	ncRNA	16.32430284	-0.407877807	0.892718858	16.32430284	-0.226369522	0.926252243	16.32430284
FBgn0260721	pseudo	0.144899044	0		0.144899044	0		0.144899044
FBgn0260722	ncRNA	847.2557469	-0.045666001	0.996902828	847.2557469	0.053034987	0.986877529	847.2557469
FBgn0260741	CG3281 gene product from transcript	259.8988873	-0.269014383	0.746276133	259.8988873	-0.150815907	0.879513184	259.8988873
FBgn0260742	CG12213 gene product from transcrip	1189.787607	-0.235875833	0.648838228	1189.787607	-0.195823653	0.700663372	1189.787607
FBgn0260743	Glutamate Carrier 1	1207.512417	-0.068295539	0.994497358	1207.512417	0.187434762	0.850061293	1207.512417
FBgn0260744	Transport and Golgi organization 9	4235.520192	-0.010255741	0.998322595	4235.520192	-0.207510769	0.798530085	4235.520192
FBgn0260745	midline fasciclin	3822.796551	0.27623984	0.495925879	3822.796551	0.209142928	0.663600791	3822.796551
FBgn0260746	Ectoderm-expressed 3	963.8844956	-0.079454709	0.994497358	963.8844956	0.199776548	0.868136717	963.8844956
FBgn0260747	CG5010 gene product from transcript	1918.169573	0.053699475	0.994497358	1918.169573	0.412581069	0.377036537	1918.169573
FBgn0260748	CG5004 gene product from transcript	1388.35194	0.088482025	0.987458415	1388.35194	0.017942033	0.993457973	1388.35194
FBgn0260749	CG5640 gene product from transcript	1345.11082	-0.180670496	0.943412521	1345.11082	-0.172335079	0.869625206	1345.11082
FBgn0260750	Multi-substrate lipid kinase	422.4806139	-0.084228579	0.994497358	422.4806139	0.139755202	0.933048047	422.4806139
FBgn0260753	Pigment-dispersing factor receptor	198.3046535	0.305393474	0.915289583	198.3046535	0.221920021	0.90616036	198.3046535
FBgn0260755	CG42553 gene product from transcrip	431.9848209	-0.369404896	0.416369183	431.9848209	-0.159671733	0.851566102	431.9848209
FBgn0260756	CG42554 gene product from transcrip	203.1053586	-0.380779594	0.858079153	203.1053586	-0.366643447	0.796573058	203.1053586
FBgn0260758	CG42556 gene product from transcrip	29.32838052	0.369711852	0.893753317	29.32838052	0.194909101	0.933048047	29.32838052
FBgn0260759	CG42557 gene product from transcrip	0			0			0
FBgn0260760	CG42558 gene product from transcrip	0			0			0
FBgn0260761	CG42559 gene product from transcrip	0			0			0
FBgn0260762	CG42560 gene product from transcrip	0			0			0
FBgn0260763	CG42561 gene product from transcrip	658.1974861	-1.084422822	0.031605294	658.1974861	-0.088377337	0.972380715	658.1974861

[illegible]

FBgn0261834	CG42766 gene product from transcrip	1.177377569	0.140594179		1.177377569	0.047722305
FBgn0261835	ncRNA	85.87574129	-0.575666986	0.713581746	85.87574129	-0.485779247
FBgn0261836	Muscle-specific protein 300 kDa	10868.77862	0.27520434	0.9379015	10868.77862	0.349911267
FBgn0261837	CG42769 gene product from transcrip	0			0	
FBgn0261838	CG42770 gene product from transcrip	0			0	
FBgn0261839	CG42771 gene product from transcrip	0			0	
FBgn0261840	CG42772 gene product from transcrip	0			0	
FBgn0261841	CG42773 gene product from transcrip	0			0	
FBgn0261842	CG42774 gene product from transcrip	0			0	
FBgn0261843	CG42775 gene product from transcrip	0			0	
FBgn0261844	CG42776 gene product from transcrip	0			0	
FBgn0261845	CG42777 gene product from transcrip	0			0	
FBgn0261846	CG42778 gene product from transcrip	0.221386409	0.173816599		0.221386409	0
FBgn0261847	CG42779 gene product from transcrip	749.7816823	3.531284011	1.15E-22	749.7816823	2.454667291
FBgn0261848	CG42780 gene product from transcrip	2.158306241	-0.019089834	0.998322595	2.158306241	-0.109410159
FBgn0261849	CG42781 gene product from transcrip	0.07379547	0.108851729		0.07379547	0
FBgn0261850	Xeroderma pigmentosum D ortholog (H	598.6787872	-0.100277171	0.985645036	598.6787872	-0.261007717
FBgn0261853	CG42782 gene product from transcrip	0.458760182	0		0.458760182	0
FBgn0261854	atypical protein kinase C	1643.091703	-0.224374559	0.809703489	1643.091703	0.005743988
FBgn0261856	pseudo	0.100708642	0		0.100708642	0
FBgn0261857	pseudo	1.425538341	-0.078970276		1.425538341	-0.12518338
FBgn0261858	CG42787 gene product from transcrip	0.957208949	0.139740158		0.957208949	0.069475873
FBgn0261859	CG42788 gene product from transcrip	501.7485379	-0.289407227	0.635378395	501.7485379	0.032435388
FBgn0261860	CG42789 gene product from transcrip	0.07379547	0.108851729		0.07379547	0
FBgn0261861	CG42790 gene product from transcrip	0.056320698	0		0.056320698	0
FBgn0261862	withered	3113.071692	0.221115999	0.933260463	3113.071692	-0.263612522
FBgn0261871	CG33507 gene product from transcrip	18.10093145	-0.308243664	0.950976319	18.10093145	-0.264100699
FBgn0261872	CG33522 gene product from transcrip	1658.45179	0.049628888	0.994497358	1658.45179	-0.109813408
FBgn0261873	stardust	2022.354808	0.246154134	0.811144636	2022.354808	0.300263739
FBgn0261881	lethal (2) 35Be	449.8876089	-0.033928069	0.996983104	449.8876089	0.034751071
FBgn0261885	CG7467 gene product from transcript	3577.660438	0.355992258	0.425680277	3577.660438	0.318670511
FBgn0261924	ncRNA	0			0	
FBgn0261925	CG42792 gene product from transcrip	1.52124357	-0.043888315		1.52124357	-0.122194215
FBgn0261927	ncRNA	33.69632021	-0.226613908	0.978973481	33.69632021	-0.306837043
FBgn0261928	CG42795 gene product from transcrip	49.0411717	0.429739187	0.758942416	49.0411717	0.000399574
FBgn0261929	5-hydroxytryptamine (serotonin) recep	11.38351187	-0.063115729	0.995584438	11.38351187	-0.511179217
FBgn0261930	ventral nervous system defective	62.99786718	-0.839691412	0.351156938	62.99786718	-1.091898923
FBgn0261931	CG42797 gene product from transcrip	699.1428855	0.009613091	0.998322595	699.1428855	0.187363188
FBgn0261932	CG42798 gene product from transcrip	1087.283009	0.350283322	0.890114416	1087.283009	0.289877428
FBgn0261933	Small ribonucleoprotein particle protei	593.0576644	0.12692904	0.953305045	593.0576644	0.285810592
FBgn0261934	CG42799 gene product from transcrip	1034.40002	0.308607508	0.879879899	1034.40002	0.278781249
FBgn0261938	mitochondrial RNA polymerase	323.8178604	-0.204930007	0.824841397	323.8178604	0.03935895
FBgn0261952	shrew	0.394207517	0.008768026		0.394207517	-0.038220412
FBgn0261953	Transcription factor AP-2	342.7942049	0.073070697	0.994497358	342.7942049	-0.106271542
FBgn0261954	enhanced adult sensory threshold	2140.03757	0.235268226	0.923470826	2140.03757	0.367314469
FBgn0261955	knockdown	16175.99757	0.370424154	0.803460791	16175.99757	0.614703749
FBgn0261963	midline	173.2407382	0.077044503	0.994497358	173.2407382	0.206816938
FBgn0261973	ncRNA	0			0	
FBgn0261974	ncRNA	0			0	
FBgn0261975	CG42806 gene product from transcrip	1195.245235	0.330879722	0.580354456	1195.245235	0.034587499
FBgn0261976	CG18013 gene product from transcrip	164.8535636	-0.140082105	0.969626359	164.8535636	0.0393885
FBgn0261983	lethal (2) giant discs 1	922.4563546	-0.167854034	0.954654825	922.4563546	-0.062129186
FBgn0261984	Inositol-requiring enzyme-1	1277.089427	-0.165902446	0.881480458	1277.089427	-0.122210578
FBgn0261985	CG1228 gene product from transcript	2612.166651	-0.216708165	0.93024912	2612.166651	-0.405864631
FBgn0261986	CG5053 gene product from transcript	752.6929128	0.027623467	0.997459641	752.6929128	0.006957394
FBgn0261987	Peroxinectin-like	4.461421392	-0.086943458	0.9945211	4.461421392	-0.017712073
FBgn0261988	G protein-coupled receptor kinase 2	1541.057601	0.090333611	0.987910537	1541.057601	-0.115132668
FBgn0261989	CG42807 gene product from transcrip	1523.154476	0.060853769	0.994497358	1523.154476	0.011729927
FBgn0261990	CG42808 gene product from transcrip	580.1038972	0.423246464	0.345443738	580.1038972	0.29685581
FBgn0261991	CG42809 gene product from transcrip	0			0	
FBgn0261992	CG42810 gene product from transcrip	1.240878691	0.252927096		1.240878691	-0.014376178
FBgn0261993	CG42811 gene product from transcrip	0.461280428	0		0.461280428	0.047040774
FBgn0261994	CG42812 gene product from transcrip	1.982196323	-0.259565617	0.946165413	1.982196323	0.050117236
FBgn0261995	CG42813 gene product from transcrip	1962.735696	0.247453013	0.832593675	1962.735696	0.121052791
FBgn0261996	CG42814 gene product from transcrip	0.147590939	0.153120924		0.147590939	0
FBgn0261997	CG42815 gene product from transcrip	0.364670766	0.190673221		0.364670766	0
FBgn0261998	CG42816 gene product from transcrip	4.35419196	0.991887339	0.124558231	4.35419196	0.285801255
FBgn0261999	CG42817 gene product from transcrip	486.9285358	0.208738879	0.932628168	486.9285358	0.468265686
FBgn0262000	CG42818 gene product from transcrip	3.232298883	0.00078846	0.999781966	3.232298883	0.136446525
FBgn0262001	CG42819 gene product from transcrip	1.049358161	0		1.049358161	0.134824589
FBgn0262002	CG42820 gene product from transcrip	2.079501178	0.438696061	0.460797731	2.079501178	-0.043104604
FBgn0262003	CG42821 gene product from transcrip	207.8262656	1.445087715	0.017854026	207.8262656	0.879069595
FBgn0262004	CG42822 gene product from transcrip	970.2258552	-0.243874941	0.95975044	970.2258552	-0.150659681
FBgn0262005	CG42823 gene product from transcrip	81.61527436	-0.151477093	0.99157593	81.61527436	-0.958376336
FBgn0262006	CG42824 gene product from transcrip	4.501785702	0.002604709	0.999629143	4.501785702	0.193745402
FBgn0262007	CG42825 gene product from transcrip	134.984301	-2.790762469	3.28E-11	134.984301	-1.434567877
FBgn0262008	CG42826 gene product from transcrip	1.505818132	0.000472942		1.505818132	-0.17313475
FBgn0262009	CG42827 gene product from transcrip	0.861639523	0.39165372		0.861639523	0
FBgn0262010	CG42828 gene product from transcrip	0.056320698	0		0.056320698	0
FBgn0262017	CG42830 gene product from transcrip	0.664159226	0.216058883		0.664159226	0
FBgn0262018	Cadherin-N2	61.52365298	0.319816469	0.893280346	61.52365298	-0.004691747
FBgn0262020	CG42831 gene product from transcrip	0.646549802	-0.022542475		0.646549802	0.115200669
FBgn0262021	CG42832 gene product from transcrip	42.59280181	2.113703561	3.63E-06	42.59280181	0.571769057
FBgn0262022	CG42833 gene product from transcrip	213.1385899	0.453488597	0.607392541	213.1385899	0.461823378
FBgn0262023	CG42834 gene product from transcrip	2026.424159	-0.30394249	0.933125958	2026.424159	-0.487591461
FBgn0262024	CG42835 gene product from transcrip	65.76113973	-0.408520481	0.89770139	65.76113973	-0.430303078
FBgn0262025	ncRNA	0.12877022	0		0.12877022	0
FBgn0262026	CG42837 gene product from transcrip	0			0	
FBgn0262027	pseudo	0.682091679	0.220905492		0.682091679	0.072009397
FBgn0262028	ncRNA	68.90877958	-0.658721566	0.468843814	68.90877958	-0.618530922
FBgn0262029	dachs	1858.145562	-0.087178614	0.988572405	1858.145562	-0.091093823
FBgn0262030	CG42841 gene product from transcrip	0.130116168	0.082464995		0.130116168	0
FBgn0262031	pseudo	0			0	
FBgn0262033	ncRNA	0.442772817	0.202930855		0.442772817	0
FBgn0262035	CG42846 gene product from transcrip	2718.595857	0.404598526	0.573114477	2718.595857	0.298151421
FBgn0262036	CG42847 gene product from transcrip	673.0830116	0.752195619	0.199504223	673.0830116	0.843224597
FBgn0262057	Serpin 77Ba	1558.125198	0.035936442	0.994497358	1558.125198	0.333221851
FBgn0262081	C-terminal Src kinase	1707.83608	-0.195193637	0.965607861	1707.83608	-0.536889661
FBgn0262095	ncRNA	6.831083691	0.176697937	0.988354972	6.831083691	-0.170014879
FBgn0262097	ncRNA	2.031323604	0.384160668	0.398752201	2.031323604	0
FBgn0262098	CG42851 gene product from transcrip	0.590363756	0.212547667		0.590363756	0
FBgn0262099	CG42852 gene product from transcrip	0.594196916	0		0.594196916	0.213090201
FBgn0262100	CG42853 gene product from transcrip	2.250556722	0.672584673	0.124233273	2.250556722	0
FBgn0262101	CG42854 gene product from transcrip	4.99805094	0.029919499	0.998322595	4.99805094	-0.212149852
FBgn0262102	CG42855 gene product from transcrip	2.034631454	0.333234353	0.835962233	2.034631454	-0.180667962
FBgn0262103	Salt-inducible kinase 3	1417.939204	0.145855399	0.972945531	1417.939204	-0.176437425
FBgn0262104	CG42857 gene product from transcrip	3.295204065	0.640493528	0.209434326	3.295204065	0.058920559
FBgn0262105	ncRNA	0.295181878	0.186873145		0.295181878	0
FBgn0262106	ncRNA	1.07757312	0.265134692		1.07757312	0.094882907
FBgn0262107	ncRNA	48.49837551	0.472005093	0.826430371	48.49837551	0.4195979
FBgn0262108	ncRNA	121.9512631	-0.44622233	0.717821646	121.9512631	-0.332370264
FBgn0262109	ncRNA	42659.87875	0.013793854	0.998322595	42659.87875	-0.21213643
FBgn0262111	forked	121.5192096	-0.085681228	0.994497358	121.5192096	0.148901935
FBgn0262112	shroud	1166.106087	0.259141951	0.90604688	1166.106087	0.356920721
FBgn0262114	Ran-binding protein M	6302.440504	-0.113844397	0.975999817	6302.440504	-0.362142886
FBgn0262115	CG17683 gene product from transcrip	1420.272963	-0.122784683	0.956325514	1420.272963	-0.119265865
FBgn0262116	CG40127 gene product from transcrip	2442.803398	0.127896394	0.979231897	2442.803398	0.447241679
FBgn0262117	Integrator 3	829.9929827	-0.049249163	0.995120986	829.9929827	0.126180351
FBgn0262123	lethal (2) 41Ab	140.8148057	-0.580962742	0.346311943	140.8148057	-0.125798627
FBgn0262124	unextended	1712.952135	-0.072521698	0.994497358	1712.952135	0.362333075
FBgn0262125	Sec23 ortholog (S. cerevisiae)	7162.848245	0.193637214	0.954886241	7162.848245	0.20231653
FBgn0262126	Sec24CD ortholog (H. sapiens)	2794.160248	0.34588158	0.652274434	2794.160248	0.577497897
FBgn0262127	kibra ortholog	776.768075	0.126838283	0.987458415	776.768075	0.107239278
FBgn0262139	trachealess	410.8782852	0.364480235	0.57599282	410.8782852	0.130732033
FBgn0262140	CG42866 gene product from transcrip	0.313114331	0.087768108		0.313114331	0.097751115
FBgn0262141	CG42867 gene product from transcrip	60.58103629	-0.499523142	0.764963416	60.58103629	-0.842427826
FBgn0262142	ncRNA	10.24469829	-0.602733619	0.702643548	10.24469829	-0.27842947
FBgn0262143	CG42869 gene product from transcrip	0			0	
FBgn0262144	CG42870 gene product from transcrip	0.07379547	0.108851729		0.07379547	0
FBgn0262146	Metallothionein E	927.528405	-2.014461952	1.37E-05	927.528405	-1.867747185
FBgn0262148	ncRNA	387.9337128	-0.362685013	0.54676524	387.9337128	-0.617729141
FBgn0262149	ncRNA	13.38008333	0.821958465	0.417179338	13.38008333	0.869514784
FBgn0262150	CG42876 gene product from transcrip	2.063208827	0.411871382	0.72893315	2.063208827	-0.131790353
FBgn0262160	CG9932 gene product from transcript	4817.392298	0.509666993	0.376005157	4817.392298	0.428467231
FBgn0262163	pseudo	0.852492285	0.096164704		0.852492285	0.319220202
FBgn0262166	CG8445 gene product from transcript	286.4380842	-0.007977763	0.998322595	286.4380842	-0.155736677
FBgn0262167	anastral spindle 1	422.258676	0.037376299	0.995120986	422.258676	-0.078541232
FBgn0262169	CG2264 gene product from transcript	1459.116726	-0.173191806	0.963043532	1459.116726	-0.073167451
FBgn0262170	CG42878 gene product from transcrip	0.221010999	-0.098542318		0.221010999	-0.096216254
FBgn0262172	mir-307b stem loop					
FBgn0262174	mir-993 stem loop					
FBgn0262177	mir-375 stem loop					
FBgn0262178	mir-981 stem loop					
FBgn0262180	mir-2283 stem loop					
FBgn0262181	mir-972 stem loop					
FBgn0262182	mir-957 stem loop					
FBgn0262185	mir-995 stem loop					
FBgn0262187	mir-970 stem loop					
FBgn0262189	mir-2500 stem loop					
FBgn0262191	mir-2497 stem loop					
FBgn0262194	mir-2489 stem loop					
FBgn0262200	mir-962 stem loop					
FBgn0262201	mir-973 stem loop					
FBgn0262203	mir-967 stem loop					
FBgn0262204	mir-2279 stem loop					
FBgn0262205	mir-932 stem loop					
FBgn0262210	mir-927 stem loop					
FBgn0262211	mir-2498 stem loop					
FBgn0262213	mir-2495 stem loop					
FBgn0262214	mir-929 stem loop					
FBgn0262215	mir-989 stem loop					
FBgn0262216	mir-974 stem loop					
FBgn0262220	mir-987 stem loop					
FBgn0262222	mir-971 stem loop					
FBgn0262223	mir-994 stem loop					
FBgn0262227	mir-991 stem loop					
FBgn0262228	mir-975 stem loop					
FBgn0262233	mir-2494 stem loop					
FBgn0262235	mir-983-1 stem loop					
FBgn0262237	mir-2491 stem loop					
FBgn0262239	mir-961 stem loop					
FBgn0262243	mir-964 stem loop					
FBgn0262249	mir-958 stem loop					
FBgn0262251	mir-977 stem loop					
FBgn0262252	mir-965 stem loop					
FBgn0262256	mir-959 stem loop					
FBgn0262260	mir-2282 stem loop					
FBgn0262262	mir-997 stem loop					
FBgn0262263	mir-969 stem loop					
FBgn0262266	mir-980 stem loop					
FBgn0262269	mir-955 stem loop					
FBgn0262272	mir-1002 stem loop					
FBgn0262275	mir-2280 stem loop					
FBgn0262279	mir-983-2 stem loop					
FBgn0262282	mir-984 stem loop					
FBgn0262283	mir-985 stem loop					
FBgn0262286	mir-1001 stem loop					
FBgn0262290	mir-iab-8 stem loop					
FBgn0262291	mir-956 stem loop					
FBgn0262293	mir-2490 stem loop					
FBgn0262297	mir-2496 stem loop					
FBgn0262300	mir-988 stem loop					
FBgn0262301	mir-979 stem loop					
FBgn0262302	mir-954 stem loop					
FBgn0262307	mir-990 stem loop					
FBgn0262314	mir-978 stem loop					
FBgn0262315	mir-2493 stem loop					
FBgn0262316	mir-986 stem loop					
FBgn0262317	mir-966 stem loop					
FBgn0262319	mir-1000 stem loop					
FBgn0262321	mir-960 stem loop					
FBgn0262323	mir-252 stem loop					
FBgn0262327	mir-2281 stem loop					
FBgn0262330	mir-193 stem loop					
FBgn0262331	mir-999 stem loop					
FBgn0262332	mir-968 stem loop					
FBgn0262333	mir-963 stem loop					
FBgn0262334	mir-992 stem loop					
FBgn0262335	mir-2492 stem loop					
FBgn0262338	mir-2499 stem loop					
FBgn0262339	mir-982 stem loop					
FBgn0262341	mir-976 stem loop					
FBgn0262351	CG43049 gene product from transcrip	0.485449224	0.126597316		0.485449224	-0.025350364
FBgn0262352	CG43050 gene product from transcrip	3.732624584	0.696966925	0.343164434	3.732624584	0.050636021
FBgn0262353	ncRNA	445.4822667	-0.054293632	0.994876798	445.4822667	-0.168409983
FBgn0262354	CG43052 gene product from transcrip	1.464008634	0.198284062		1.464008634	-0.086922124
FBgn0262355	ncRNA	0.07379547	0.108851729		0.07379547	0
FBgn0262356	CG43054 gene product from transcrip	0.07379547	0.108851729		0.07379547	0
FBgn0262357	CG43055 gene product from transcrip	0.241472153	0.06444487		0.241472153	0.110367284
FBgn0262358	CG43056 gene product from transcrip	0			0	
FBgn0262359	CG43057 gene product from transcrip	0			0	
FBgn0262360	CG43058 gene product from transcrip	0.502923996	0.15336473		0.502923996	-0.025184644
FBgn0262361	CG43059 gene product from transcrip	0.516568287	0.208268862		0.516568287	0
FBgn0262362	CG43060 gene product from transcrip	556.7365857	-0.906127137	0.235098752	556.7365857	-0.530516305
FBgn0262363	CG43061 gene product from transcrip	3.440898145	-0.683659744	0.438006735	3.440898145	-0.515869142
FBgn0262364	CG43062 gene product from transcrip	8.057137369	0.463222857	0.84105523	8.057137369	0.308329507
FBgn0262365	CG43063 gene product from transcrip	0.100708642	0		0.100708642	0
FBgn0262366	CG43064 gene product from transcrip	300.5177171	-0.395495028	0.699729767	300.5177171	-0.232668748
FBgn0262367	mir-13b-1 stem loop					
FBgn0262368	mir-282 stem loop					
FBgn0262369	mir-31b stem loop					
FBgn0262370	mir-7 stem loop					
FBgn0262371	mir-263b stem loop					
FBgn0262372	mir-3 stem loop					
FBgn0262373	mir-9a stem loop					
FBgn0262374	mir-2b-1 stem loop					
FBgn0262375	mir-1005 stem loop					
FBgn0262376	mir-219 stem loop					
FBgn0262377	mir-2a-1 stem loop					
FBgn0262378	mir-317 stem loop					
FBgn0262379	mir-1012 stem loop					
FBgn0262380	mir-309 stem loop					
FBgn0262381	mir-276b stem loop					
FBgn0262382	mir-1010 stem loop					
FBgn0262383	mir-284 stem loop					
FBgn0262384	mir-1014 stem loop					
FBgn0262385	mir-307a stem loop					
FBgn0262386	mir-306 stem loop					
FBgn0262387	mir-190 stem loop					
FBgn0262388	mir-92b stem loop					
FBgn0262389	mir-1017 stem loop					
FBgn0262390	mir-303 stem loop					
FBgn0262391	mir-184 stem loop					
FBgn0262392	mir-1004 stem loop					
FBgn0262393	mir-33 stem loop					
FBgn0262394	mir-281-1 stem loop					
FBgn0262395	mir-12 stem loop					
FBgn0262396	mir-314 stem loop					
FBgn0262397	mir-2c stem loop					
FBgn0262398	mir-124 stem loop					
FBgn0262400	mir-287 stem loop					
FBgn0262401	mir-285 stem loop					
FBgn0262402	mir-311 stem loop					
FBgn0262403	mir-278 stem loop					
FBgn0262404	mir-996 stem loop					
FBgn0262405	mir-998 stem loop					
FBgn0262406	ncRNA					
FBgn0262407	mir-308 stem loop					
FBgn0262408	mir-5 stem loop					
FBgn0262409	mir-304 stem loop					
FBgn0262410	mir-6-2 stem loop					
FBgn0262411	mir-92a stem loop					
FBgn0262412	mir-1008 stem loop					
FBgn0262413	mir-9b stem loop					
FBgn0262414	mir-4 stem loop					
FBgn0262415	mir-289 stem loop					
FBgn0262416	mir-31a stem loop					
FBgn0262417	mir-316 stem loop					
FBgn0262418	mir-6-3 stem loop					
FBgn0262419	mir-277 stem loop					
FBgn0262420	mir-318 stem loop					
FBgn0262421	mir-13b-2 stem loop					
FBgn0262422	mir-1011 stem loop					
FBgn0262423	mir-281-2 stem loop					
FBgn0262424	mir-10 stem loop					
FBgn0262425	mir-288 stem loop					
FBgn0262426	mir-283 stem loop					
FBgn0262427	mir-1016 stem loop					
FBgn0262428	mir-87 stem loop					
FBgn0262429	mir-13a stem loop					
FBgn0262430	mir-9c stem loop					
FBgn0262431	mir-1003 stem loop					
FBgn0262432	mir-8 stem loop					
FBgn0262433	mir-210 stem loop					
FBgn0262434	mir-11 stem loop					
FBgn0262435	mir-1009 stem loop					
FBgn0262436	mir-310 stem loop					
FBgn0262437	mir-274 stem loop					
FBgn0262438	mir-1006 stem loop					
FBgn0262440	mir-280 stem loop					
FBgn0262441	mir-313 stem loop					
FBgn0262442	mir-2501 stem loop					
FBgn0262443	mir-286 stem loop					
FBgn0262444	mir-1013 stem loop					
FBgn0262445	mir-133 stem loop					
FBgn0262446	mir-137 stem loop					
FBgn0262447	mir-14 stem loop					
FBgn0262448	mir-279 stem loop					
FBgn0262449	mir-2b-2 stem loop					
FBgn0262450	mir-312 stem loop					
FBgn0262451	bantam					
FBgn0262452	mir-79 stem loop					
FBgn0262453	mir-275 stem loop					
FBgn0262454	mir-1007 stem loop					
FBgn0262455	mir-1 stem loop					
FBgn0262456	mir-125 stem loop					
FBgn0262457	mir-1015 stem loop					
FBgn0262458	mir-305 stem loop					
FBgn0262459	mir-34 stem loop					
FBgn0262460	mir-2a-2 stem loop					
FBgn0262461	mir-315 stem loop					
FBgn0262462	mir-iab-4 stem loop					
FBgn0262463	mir-100 stem loop					
FBgn0262464	mir-6-1 stem loop					
FBgn0262467	Synthesis of cytochrome c oxidase	1011.253433	0.179645352	0.892718858	1011.253433	0.409981955
FBgn0262473	Toll	1180.599687	0.046266931	0.994497358	1180.599687	0.244460363
FBgn0262475	bruno-2	708.0369769	-0.239777085	0.886446899	708.0369769	-0.191679866
FBgn0262476	CG43066 gene product from transcrip	128.5286436	0.001966715	0.999629143	128.5286436	-0.126067372
FBgn0262477	Forkhead box P	61.47190285	-0.144762879	0.984673682	61.47190285	-0.620512188
FBgn0262478	CG43068 gene product from transcrip	0.663783816	0.101095794		0.663783816	-0.040112414
FBgn0262479	CG43069 gene product from transcrip	1.003286247	0.229986838		1.003286247	0.060525294
FBgn0262480	CG43070 gene product from transcrip	0.060151179	-0.097832662		0.060151179	-0.093256207
FBgn0262481	CG43071 gene product from transcrip	333.4983956	0.50659566	0.493147605	333.4983956	0.481153612
FBgn0262482	CG43072 gene product from transcrip	1.052199751	-0.075358474		1.052199751	0.168400167
FBgn0262483	RIM-binding protein	142.7204752	0.286201101	0.804678243	142.7204752	0.021254011
FBgn0262484	CG43074 gene product from transcrip	1.483987818	0.117164949		1.483987818	0.233904207
FBgn0262485	CG43075 gene product from transcrip	0.664159226	0.216058883		0.664159226	0
FBgn0262486	CG43076 gene product from transcrip	1.132169393	0.234996534		1.132169393	0.026774024
FBgn0262487	CG43077 gene product from transcrip	1.753616498	0.367522294	0.406820555	1.753616498	0
FBgn0262508	CG43078 gene product from transcrip	4708.488944	0.40397866	0.688566959	4708.488944	0.504500764
FBgn0262509	neuromusculin	1588.512093	0.221318505	0.961442281	1588.512093	0.496224085
FBgn0262510	pseudo	0.360969511	0		0.360969511	0
FBgn0262511	Vacuolar H[+] ATPase 44kD subunit	5566.706923	0.02019803	0.998322595	5566.706923	0.320838427
FBgn0262512	Vacuolar H[+] ATPase 14kD subunit 1	6736.100476	0.01134655	0.998322595	6736.100476	0.140807297
FBgn0262513	Vacuolar H[+] ATPase 16kD subunit 4	0.203911637	0.121748477		0.203911637	0
FBgn0262514	Vacuolar H[+] ATPase PPA1 subunit 2	1.040450217	0.433703918		1.040450217	0
FBgn0262515	Vacuolar H[+] ATPase AC45 accessory s	10987.54908	0.202529106	0.824841397	10987.54908	0.315259989
FBgn0262516	Transient receptor potential cation cha	4.033736472	0.145836529	0.975861786	4.033736472	-0.003417247
FBgn0262517	CG32210 gene product from transcrip	1159.669591	-0.389295381	0.339396406	1159.669591	-0.222015594
FBgn0262518	CG8287 gene product from transcript	2303.130915	-0.04940248	0.994497358	2303.130915	0.040214514
FBgn0262519	CG8103 gene product from transcript	5913.331989	0.044240992	0.994497358	5913.331989	-0.009004174
FBgn0262524	verrocchio	97.47748543	-0.116932604	0.989856236	97.47748543	-0.102710808
FBgn0262527	non-specific lethal 1	2028.735229	0.109938024	0.968528723	2028.735229	0.048935928
FBgn0262528	CG43082 gene product from transcrip	0.997055392	0.090148368		0.997055392	0
FBgn0262529	CG43083 gene product from transcrip	491.0638577	0.332150632	0.703936445	491.0638577	0.213767808
FBgn0262530	CG43084 gene product from transcrip	457.346869	0.141497749	0.985645036	457.346869	0.401036095
FBgn0262531	CG43085 gene product from transcrip	5787.417328	-0.113223705	0.994497358	5787.417328	-0.369688865
FBgn0262532	pseudo	3.197910085	-0.608587211	0.50079657	3.197910085	-0.028651523
FBgn0262533	pseudo	0.525490855	-0.106761776		0.525490855	-0.062976746
FBgn0262534	CG43088 gene product from transcrip	0.221386409	0.173816599		0.221386409	0
FBgn0262535	CG43089 gene product from transcrip	0.221386409	0.173816599		0.221386409	0
FBgn0262536	CG43090 gene product from transcrip	0.26792183	-0.090266931		0.26792183	-0.08803755
FBgn0262537	CG43091 gene product from transcrip	1.40952898	-0.123105022		1.40952898	-0.073013898
FBgn0262538	CG43092 gene product from transcrip	1.048037677	0.089174739		1.048037677	0.341234448
FBgn0262539	CG43093 gene product from transcrip	0.933746478	-0.026637334		0.933746478	-0.087350377
FBgn0262540	CG43094 gene product from transcrip	0.401977276	0.076010367		0.401977276	0.097395221
FBgn0262541	CG43095 gene product from transcrip	0.186436866	0.066048487		0.186436866	0
FBgn0262542	pseudo	0			0	
FBgn0262543	ncRNA	0.618919771	0.09470003		0.618919771	-0.084377944
FBgn0262544	CG43098 gene product from transcrip	0.811750165	0.221487775		0.811750165	0
FBgn0262546	pseudo	0.664159226	0.216058883		0.664159226	0
FBgn0262547	CG43101 gene product from transcrip	0.549568215	0.113998686		0.549568215	0.076962298
FBgn0262559	Malate dehydrogenase 2	10865.12138	0.277203338	0.893280346	10865.12138	0.552028882
FBgn0262560	wicked	366.3373117	-0.014445241	0.998322595	366.3373117	0.025795947
FBgn0262562	CG43102 gene product from transcrip	369.973615	0.134176331	0.979231897	369.973615	0.343430173
FBgn0262563	CG43103 gene product from transcrip	554.6565272	0.206383321	0.944197659	554.6565272	0.232744685
FBgn0262564	CG43104 gene product from transcrip	0			0	
FBgn0262565	pseudo	5.674356901	0.262469746	0.948611894	5.674356901	-0.166803564
FBgn0262566	CG43106 gene product from transcrip	0.720479924	0.199628844		0.720479924	0
FBgn0262567	CG43107 gene product from transcrip	1.315074257	-0.372898805		1.315074257	-0.420983415
FBgn0262568	CG43108 gene product from transcrip	2.22176903	0.391552599	0.6828669	2.22176903	0.080212051
FBgn0262569	CG43109 gene product from transcrip	23.95670805	-0.728497112	0.519319479	23.95670805	-0.726760619

[illegible]

FBgn0262690	ncRNA	1.124828181	-0.020134722		1.124828181	-0.067649181		1.124828181	-0.092945843	
FBgn0262699	retina aberrant in pattern	1243.919306	-0.030388207	0.995584438	1243.919306	-0.012573109	0.995619712	1243.919306	0.072408536	0.895781869
FBgn0262714	Sin3A-associated protein 130	1125.383522	0.002656209	0.998322595	1125.383522	-0.172275825	0.737394194	1125.383522	0.141093245	0.672295054
FBgn0262716	Actin-related protein 3	4067.222179	-0.195959687	0.825494693	4067.222179	-0.083967054	0.93637756	4067.222179	0.18393216	0.629480234
FBgn0262717	CG43161 gene product from transcrip	2154.535831	0.4245481	0.690358538	2154.535831	0.610005112	0.342305154	2154.535831	-1.318840621	0.000448456
FBgn0262719	CG43163 gene product from transcrip	821.7378824	0.405481541	0.443425647	821.7378824	0.427242309	0.388607729	821.7378824	0.017893041	0.982926525
FBgn0262721	CG43165 gene product from transcrip	200.0279489	-0.231054817	0.946165413	200.0279489	-0.01666226	0.997037674	200.0279489	0.156883517	0.842253687
FBgn0262722	CG43166 gene product from transcrip	817.1349046	-2.066766051	6.34E-05	817.1349046	-1.078860053	0.151504118	817.1349046	0.616161512	0.447537966
FBgn0262728	Peptidyl-alpha-hydroxyglycine-alpha-ar	435.5417942	-0.131143302	0.988572405	435.5417942	-0.261494943	0.848588436	435.5417942	0.318633021	0.642182307
FBgn0262730	CG14446 gene product from transcrip	732.0818517	0.423087593	0.77169846	732.0818517	0.3864121	0.750344828	732.0818517	0.014639736	0.990956456
FBgn0262731	putative noncoding RNA 013:4	219.0994817	-0.184946958	0.949952217	219.0994817	0.340750784	0.634145882	219.0994817	0.335902901	0.471543372
FBgn0262732	multiprotein bridging factor 1	2923.779744	0.092179565	0.985645036	2923.779744	0.328220972	0.511201392	2923.779744	-0.306238724	0.398902182
FBgn0262733	Src oncogene at 64B	610.7834876	0.013934002	0.998322595	610.7834876	0.193176519	0.90480014	610.7834876	0.273392554	0.690100351
FBgn0262734	RNA-binding protein 2	10251.4452	0.37711992	0.805580095	10251.4452	0.570418211	0.466625824	10251.4452	-0.544448663	0.348248651
FBgn0262735	IGF-II mRNA-binding protein	2055.805438	0.507630101	0.749926685	2055.805438	0.710821037	0.455161017	2055.805438	-0.638974514	0.378526863
FBgn0262736	Vacuolar H[+] ATPase 16kD subunit 1	55526.79831	0.074627144	0.993966728	55526.79831	0.334173937	0.58226435	55526.79831	-0.364565886	0.353601801
FBgn0262737	mushroom-body expressed	4563.129455	0.202270828	0.966088307	4563.129455	-0.066273726	0.980691994	4563.129455	0.359156329	0.584319416
FBgn0262738	no receptor potential A	627.883973	0.194207898	0.952645803	627.883973	0.195569261	0.870965316	627.883973	0.045410014	0.958924384
FBgn0262739	Argonaute-1	4107.537248	0.302095461	0.876824427	4107.537248	0.382098074	0.686729636	4107.537248	-0.047635916	0.964377123
FBgn0262740	Ecotropic viral integration site 5 orthol	1241.23938	0.165214227	0.97064214	1241.23938	0.209603378	0.864997596	1241.23938	-0.023880897	0.98040309
FBgn0262741	mRNA-like ncRNA in embryogenesis 23	84.15957137	-0.122507317	0.994497358	84.15957137	-0.180901291	0.94426666	84.15957137	0.394479831	0.672491806
FBgn0262742	Fasciclin 1	1852.068109	-0.170594866	0.960160242	1852.068109	-0.075091424	0.966097907	1852.068109	0.231206996	0.676699921
FBgn0262743	Female sterile (2) Ketel	2731.849427	0.07223812	0.9945211	2731.849427	0.256926674	0.868136717	2731.849427	0.178435961	0.842632645
FBgn0262782	Malate dehydrogenase 1	25114.28688	0.067399857	0.994497358	25114.28688	0.180400694	0.91558304	25114.28688	-0.650641451	0.182352016
FBgn0262786	CG43167 gene product from transcrip	0.824609967	0.113188482		0.824609967	-0.04854742		0.824609967	-0.172111819	
FBgn0262787	CG43168 gene product from transcrip	1.367164378	0.271984378		1.367164378	0		1.367164378	-0.272697169	0.387151488
FBgn0262788	CG43169 gene product from transcrip	5.915146841	0.305815654	0.954378726	5.915146841	-0.211304913		5.915146841	-0.209690784	0.855940948
FBgn0262789	pseudo	0.621210393	0.201751174		0.621210393	0.118441311		0.621210393	-0.203495892	
FBgn0262790	CG43171 gene product from transcrip	0			0			0		
FBgn0262791	CG43172 gene product from transcrip	0			0			0		
FBgn0262794	CG43175 gene product from transcrip	30.43646282	0.269164802	0.969590861	30.43646282	0.380516953	0.832797896	30.43646282	-0.590161483	0.489571658
FBgn0262795	CG43176 gene product from transcrip	0			0			0		
FBgn0262801	twisted bristles roughened eye	2076.589894	0.458676027	0.320238253	2076.589894	0.500761167	0.232526928	2076.589894	-0.614667738	0.049438545
FBgn0262806	CG43177 gene product from transcrip	0			0			0		
FBgn0262807	CG43178 gene product from transcrip	0			0			0		
FBgn0262808	CG43179 gene product from transcrip	186.7960861	0.291080415	0.936128798	186.7960861	0.536586887	0.592565499	186.7960861	-0.165650349	0.869064744
FBgn0262810	CG43181 gene product from transcrip	1.44261215	0.12703576		1.44261215	0.53669366		1.44261215	-0.125095196	0.888121316
FBgn0262811	CG43182 gene product from transcrip	509.2508607	0.846901219	0.32394952	509.2508607	0.71087056	0.468891997	509.2508607	-0.107207769	0.931948905
FBgn0262812	CG43183 gene product from transcrip	0.442772817	0.202930855		0.442772817	0		0.442772817	-0.203783346	
FBgn0262813	CG43184 gene product from transcrip	0.602912419	0.050780626		0.602912419	0.057935597		0.602912419	0.055774023	
FBgn0262814	CG43185 gene product from transcrip	0.100708642	0		0.100708642	0		0.100708642	0.114352451	
FBgn0262815	pseudo	146.063165	-0.157144224	0.99157593	146.063165	-0.059541968	0.9872372	146.063165	-1.099883726	0.073361606
FBgn0262816	CG43187 gene product from transcrip	112.1955976	-0.383946586	0.859393667	112.1955976	-0.276596772	0.873100502	112.1955976	0.197109051	0.842632645
FBgn0262817	CG43188 gene product from transcrip	1.757439481	0.27262526	0.888834551	1.757439481	0.103088829		1.757439481	-0.380947435	0.495804321
FBgn0262818	CG43189 gene product from transcrip	0.441042613	-0.065209358		0.441042613	-0.024329561		0.441042613	-0.050873415	
FBgn0262819	CG43190 gene product from transcrip	0			0			0		
FBgn0262820	CG43191 gene product from transcrip	8.862590295	0.314119702	0.954886241	8.862590295	0.142820979	0.961378425	8.862590295	-0.26881062	0.814576098
FBgn0262821	CG43192 gene product from transcrip	2.529594475	0.28712214	0.9379015	2.529594475	0.444176536		2.529594475	0.105425844	0.924761946
FBgn0262822	CG43193 gene product from transcrip	0.07379547	0.108851729		0.07379547	0		0.07379547	-0.111773044	
FBgn0262823	CG43194 gene product from transcrip	0			0			0		
FBgn0262824	CG43195 gene product from transcrip	0			0			0		
FBgn0262825	ncRNA	42.85631909	0.145692243	0.99157593	42.85631909	0.277102219	0.879749855	42.85631909	-0.365319163	0.675741447
FBgn0262827	CG43198 gene product from transcrip	0			0			0		
FBgn0262828	ncRNA	0.536253799	0.069360308		0.536253799	0.031174834		0.536253799	0.008445823	
FBgn0262836	CG43200 gene product from transcrip	0			0			0		
FBgn0262837	CG43201 gene product from transcrip	0			0			0		
FBgn0262838	CG43202 gene product from transcrip	0.147590939	0.153120924		0.147590939	0		0.147590939	-0.15413487	
FBgn0262839	CG43203 gene product from transcrip	0.07379547	0.108851729		0.07379547	0		0.07379547	-0.111773044	
FBgn0262840	CG43204 gene product from transcrip	6.97190309	0.396181761	0.901320232	6.97190309	0.477533887	0.758075487	6.97190309	-0.099819698	0.941276973
FBgn0262842	CG43206 gene product from transcrip	1.291695918	-0.187897558		1.291695918	-0.265296225		1.291695918	-0.07223734	0.930086818
FBgn0262843	CG43207 gene product from transcrip	1.032378436	-0.156305171		1.032378436	-0.154636094		1.032378436	0.125251267	
FBgn0262844	CG43208 gene product from transcrip	26.48734105	-0.766144162	0.482619801	26.48734105	-0.500021575	0.737574848	26.48734105	-0.117749775	0.930225895
FBgn0262845	CG43209 gene product from transcrip	2.869893077	0.63626955	0.375419261	2.869893077	-0.076230221		2.869893077	-0.822553146	0.083980589
FBgn0262846	CG43210 gene product from transcrip	0			0			0		
FBgn0262847	pseudo	0			0			0		
FBgn0262848	pseudo	2.126017661	-0.238008581	0.958499814	2.126017661	-0.157643254		2.126017661	0.227171887	0.79288764
FBgn0262849	pseudo	0			0			0		
FBgn0262850	pseudo	0			0			0		
FBgn0262851	pseudo	0			0			0		
FBgn0262852	pseudo	0			0			0		
FBgn0262853	pseudo	0			0			0		
FBgn0262854	CG43218 gene product from transcrip	8.950546535	-0.292998846	0.961435662	8.950546535	-0.473334778	0.750869987	8.950546535	0.548055531	0.521659975
FBgn0262855	CG43219 gene product from transcrip	0.33133591	0.044145862		0.33133591	0		0.33133591	0.03827177	
FBgn0262856	CG43220 gene product from transcrip	0.78289801	0.143782941		0.78289801	0		0.78289801	0.210431605	
FBgn0262857	CG43221 gene product from transcrip	0			0			0		
FBgn0262858	CG43222 gene product from transcrip	117.0767353	0.303793292	0.901284647	117.0767353	-0.131735812	0.949698854	117.0767353	0.440574464	0.49222473
FBgn0262866	Ribosomal protein S6 kinase II	417.7635044	-0.205419166	0.918956451	417.7635044	-0.233846816	0.799313864	417.7635044	0.233059465	0.658160897

[illegible]

FBgn0050187	CG30187 gene product from transcrip	579.3592526	0.298808005	0.829467893	579.3592526	0.32218998	0.717389414
FBgn0034833	CG13539 gene product from transcrip	0.94424512	0.161852579		0.94424512	0.04076142	
FBgn0034835	CG3092 gene product from transcript	1.254522982	0.231031993		1.254522982	0	
FBgn0034839	CG13540 gene product from transcrip	2.34947599	0.634595407	0.370644185	2.34947599	-0.078837555	
FBgn0034841	CG13541 gene product from transcrip	0.87740251	0.130389193		0.87740251	-0.038281008	
FBgn0034844	CG9861 gene product from transcript	4.176455098	0.873394678	0.097895691	4.176455098	-0.129934367	
FBgn0034846	CG9863 gene product from transcript	1.814479739	0.453694292	0.660414426	1.814479739	-0.06800369	
FBgn0050409	CG30409 gene product from transcrip	1.294826733	0.278381979		1.294826733	0.10018503	
FBgn0050410	Ribose-5-phosphate isomerase	4970.266732	0.227117341	0.966782845	4970.266732	0.262292469	0.874179323
FBgn0034850	CG9875 gene product from transcript	2.479121487	0.787397137	0.123844676	2.479121487	-0.059565384	
FBgn0034853	Interacts with the C terminus of ELL 1	294.3941324	0.090132928	0.994497358	294.3941324	0.192047284	0.892373033
FBgn0041712	CG9889 gene product from transcript	1477.176256	0.352066444	0.648838228	1477.176256	0.551910206	0.222256944
FBgn0034856	CG9891 gene product from transcript	11863.73604	-0.174836124	0.975532686	11863.73604	-0.23835212	0.861074999
FBgn0034858	CG10315 gene product from transcrip	2030.395941	-0.014553519	0.998322595	2030.395941	-0.083363099	0.94426666
FBgn0050411	CG30411 gene product from transcrip	790.2404482	-0.421626457	0.744801712	790.2404482	-0.791177403	0.187832154
FBgn0034867	CG13557 gene product from transcrip	0			0		
FBgn0034866	Odorant receptor 59c	0			0		
FBgn0034865	Odorant receptor 59b	0.072449522	0		0.072449522	0	
FBgn0034863	CG12227 gene product from transcrip	1.547551569	0.479850054		1.547551569	0	
FBgn0026384	Odorant receptor 59a	0.145437648	0.126400319		0.145437648	0	
FBgn0034861	CG9815 gene product from transcript	156.0243344	0.283277998	0.886446899	156.0243344	0.489366391	0.509997464
FBgn0034860	CG9812 gene product from transcript	4203.397355	-0.441305781	0.642151225	4203.397355	-0.2190369	0.873100502
FBgn0034859	CG3520 gene product from transcript	379.3366428	0.257767206	0.823048271	379.3366428	0.245095542	0.772799368
FBgn0028497	CG3530 gene product from transcript	777.3326807	-0.328053988	0.439442335	777.3326807	-0.408467963	0.235578129
FBgn0050414	CG30414 gene product from transcrip	1499.345909	-0.345839115	0.877859811	1499.345909	-0.235020601	0.894890529
FBgn0040660	CG13551 gene product from transcrip	17239.3296	-0.128254421	0.988354972	17239.3296	-0.267668629	0.831801071
FBgn0034854	Golgin-245 ortholog (H. sapiens)	2331.22085	-0.048762402	0.994497358	2331.22085	-0.029723875	0.988525421
FBgn0003977	virilizer	695.3563788	0.093424544	0.993127879	695.3563788	0.108690912	0.94714921
FBgn0034849	CG3500 gene product from transcript	507.1352031	-0.06428799	0.994497358	507.1352031	-0.124334671	0.915954217
FBgn0046253	CG3502 gene product from transcript	370.0955614	0.895968926	0.226284119	370.0955614	0.212585796	0.920127339
FBgn0050413	CG30413 gene product from transcrip	9.590048514	-1.308981647	0.052506905	9.590048514	-0.876684206	0.338780504
FBgn0050417	CG30417 gene product from transcrip	1.31084368	0.285775952		1.31084368	0	
FBgn0050416	CG30416 gene product from transcrip	1.429027094	0.279667746		1.429027094	0	
FBgn0050412	CG30412 gene product from transcrip	1.106932043	0.228582052		1.106932043	0	
FBgn0034842	Proteasome beta5 subunit-related 1	2.361455025	0.360233366	0.399353831	2.361455025	0	
FBgn0034840	CG3124 gene product from transcript	6.328935609	0.245185846	0.596449531	6.328935609	0	1
FBgn0003009	orientation disruptor	4.145130733	0.233703385	0.965741401	4.145130733	0.104188848	
FBgn0034838	CG12782 gene product from transcrip	2.719934572	0.655315995	0.269030267	2.719934572	-0.001883185	
FBgn0034837	Ribosomal protein L22-like	7.498619802	0.792738007	0.363470291	7.498619802	-0.01557274	0.997246866
FBgn0040063	yippee interacting protein 3	4.157715848	0.839021996	0.270539215	4.157715848	0.470778914	
FBgn0034834	Large Subunit 2	2.367713495	0.649894717	0.399353831	2.367713495	-0.038179037	
FBgn0034869	CG13558 gene product from transcrip	0.221386409	0.173816599		0.221386409	0	
FBgn0034870	CG13559 gene product from transcrip	29.71767627	0.598491133	0.638948409	29.71767627	0.884246698	0.257308201
FBgn0041234	Gustatory receptor 59f	4.08804337	0.021061245	0.998322595	4.08804337	0.176505977	
FBgn0050185	CG30185 gene product from transcrip	1219.203059	0.103190803	0.993966728	1219.203059	0.020879151	0.995619712
FBgn0034877	CG17280 gene product from transcrip	6147.265157	-0.001660825	0.999629143	6147.265157	0.126321358	0.951701663
FBgn0010501	Death caspase-1	549.004301	-0.452297006	0.250522657	549.004301	-0.52394763	0.13784923
FBgn0034880	Integrin alphaPS5 subunit	125.1411813	0.380673814	0.751650588	125.1411813	0.272119209	0.825700404
FBgn0015277	Phosphotidylinositol 3 kinase 59F	1062.085385	-0.24353858	0.924674154	1062.085385	-0.500642001	0.478603551
FBgn0050184	CG30184 gene product from transcrip	10.3223378	0.043509457	0.998322595	10.3223378	-0.197484131	0.939825929
FBgn0015903	apontic	571.7701863	-0.080002394	0.994497358	571.7701863	-0.085748994	0.961113238
FBgn0034882	CG5398 gene product from transcript	3.12648781	0.503835695	0.678655521	3.12648781	0.056267912	
FBgn0004795	retained	343.1472305	0.059403208	0.994497358	343.1472305	-0.01468815	0.997246866
FBgn0034887	Sulfotransferase 1	25.30644364	0.172469704	0.989856236	25.30644364	0.183399986	0.944894567

[illegible]

FBgn0263090	ncRNA	0			0			0
FBgn0263091	pseudo	0.060151179	-0.097832662		0.060151179	-0.093256207		0.060151179
FBgn0263092	pseudo	0.287010221	0.020450848		0.287010221	-0.078793369		0.287010221
FBgn0263093	pseudo	1.970634254	-0.332861717	0.805580095	1.970634254	-0.25454805		1.970634254
FBgn0263094	CG43362 gene product from transcrip	0			0			0
FBgn0263095	ncRNA	2.1433291	-0.044294048	0.995584438	2.1433291	0.191548116		2.1433291
FBgn0263096	pseudo	96.18368214	-0.168541448	0.985645036	96.18368214	-0.080262933	0.980691994	96.18368214
FBgn0263102	pipsqueak	4022.34379	0.145197346	0.926338353	4022.34379	0.170833872	0.797974284	4022.34379
FBgn0263106	DnaJ-like-1	3682.285223	0.768989863	0.390560468	3682.285223	0.5226306	0.658774601	3682.285223
FBgn0263107	bereft							
FBgn0263108	BTB-protein-VII	2459.966093	0.039163835	0.994497358	2459.966093	-0.076122198	0.936711754	2459.966093
FBgn0263109	CG43366 gene product from transcrip	90.98251498	0.442532966	0.746692563	90.98251498	0.339584868	0.799970993	90.98251498
FBgn0263110	CG43367 gene product from transcrip	509.8752958	0.029394202	0.996983104	509.8752958	0.010101405	0.997246866	509.8752958
FBgn0263111	cacophony	151.7846696	0.374253125	0.864873127	151.7846696	0.169661124	0.939574224	151.7846696
FBgn0263112	CG43369 gene product from transcrip	1311.21968	-0.218569458	0.840126208	1311.21968	-0.078030055	0.950965358	1311.21968
FBgn0263113	CG43370 gene product from transcrip	8.992353392	1.246864492	0.077063847	8.992353392	0.547576069	0.684745433	8.992353392
FBgn0263114	CG43371 gene product from transcrip	3.100526532	-0.089153613	0.994497358	3.100526532	0.086378105		3.100526532
FBgn0263116	5-hydroxytryptamine (serotonin) recep	38.37933854	0.224903257	0.970065778	38.37933854	-0.017995453	0.997246866	38.37933854
FBgn0263117	CG34377 gene product from transcrip	313.6350439	-0.014793131	0.998322595	313.6350439	-0.166818086	0.922400604	313.6350439
FBgn0263118	taxi	287.3186637	-0.012766668	0.998322595	287.3186637	0.418561383	0.450480352	287.3186637
FBgn0263120	Acyl-CoA synthetase long-chain	4371.415325	-0.049320916	0.994876798	4371.415325	0.299536092	0.709061417	4371.415325
FBgn0263121	Proteasome alpha1 subunit	22.9344893	0.080944514	0.9945211	22.9344893	0.021550876	0.997037674	22.9344893
FBgn0263130	ncRNA	0			0			0
FBgn0263131	CG43373 gene product from transcrip	46.95731373	0.239291915	0.963243134	46.95731373	0.112476177	0.964539632	46.95731373
FBgn0263132	CG43374 gene product from transcrip	2507.744214	0.499323165	0.648884221	2507.744214	0.780232859	0.22366388	2507.744214
FBgn0263133	iconoclast	333.2880337	0.085868393	0.993127879	333.2880337	0.546944432	0.246480896	333.2880337
FBgn0263143	vreteno	136.3522104	0.147261938	0.969626359	136.3522104	0.105277395	0.94426666	136.3522104
FBgn0263144	bicoid-interacting protein 3	1850.213464	0.293579908	0.622234365	1850.213464	0.360983649	0.414703432	1850.213464
FBgn0263197	CG33130 gene product from transcrip	2685.562808	0.006161701	0.998322595	2685.562808	0.018909234	0.991444605	2685.562808
FBgn0263198	Acinus	3719.329961	-0.064179784	0.979231897	3719.329961	-0.152474495	0.731018526	3719.329961
FBgn0263199	Galactokinase	2967.036149	-0.340588254	0.303717744	2967.036149	-0.234447758	0.605450378	2967.036149
FBgn0263200	Galactose-1-phosphate uridylyltransfe	2395.713309	0.093447626	0.993127879	2395.713309	0.365825547	0.634145882	2395.713309
FBgn0263201	small Cajal body-specific RNA : MeU1b	0.342180795	0.047629538		0.342180795	0.087151039		0.342180795
FBgn0263206	CG43376 gene product from transcrip	1.386189067	0.202857418		1.386189067	0.163595443		1.386189067
FBgn0263207	pseudo	0			0			0
FBgn0263208	pseudo	0			0			0
FBgn0263209	pseudo	0			0			0
FBgn0263210	pseudo	0			0			0
FBgn0263211	pseudo	0.060151179	-0.097832662		0.060151179	-0.093256207		0.060151179
FBgn0263212	pseudo	0			0			0
FBgn0263213	pseudo	0			0			0
FBgn0263214	pseudo	0			0			0
FBgn0263215	pseudo	0.7039322	-0.019091649		0.7039322	-0.051525335		0.7039322
FBgn0263216	CG43386 gene product from transcrip	100.1986129	0.287932876	0.953941411	100.1986129	0.535798567	0.651249204	100.1986129

[illegible]

FBgn0263829	CG34334 gene product from transcrip	22.8553453	1.156718297	0.112929597	22.8553453	1.920188647	0.000545642
FBgn0263830	CG40486 gene product from transcrip	647.7394554	0.117726051	0.991146172	647.7394554	0.160771138	0.925047855
FBgn0263831	XPG-like endonuclease	134.0200582	-0.374633046	0.795603346	134.0200582	-0.010511037	0.998705293
FBgn0263832	Reduction in Cnn dots 5	825.0150291	-0.078643677	0.993966728	825.0150291	-0.15901103	0.889349896
FBgn0263834	pseudo	0.056320698	0		0.056320698	0	
FBgn0263835	pseudo	0			0		
FBgn0263838	ncRNA	2.400721765	-0.146578745	0.987910537	2.400721765	-0.468110649	
FBgn0263839	ncRNA	23.94083282	1.188462452	0.080092058	23.94083282	-0.283067076	0.897821035
FBgn0263841	CG43702 gene product from transcrip	1.275563189	0.32709476		1.275563189	0.067223486	
FBgn0263842	CG43703 gene product from transcrip	0			0		
FBgn0263843	ncRNA	11.59982335	-0.367920272	0.924867928	11.59982335	-0.255077704	0.915890998
FBgn0263844	ncRNA	0			0		
FBgn0263845	ncRNA	0.07379547	0.108851729		0.07379547	0	
FBgn0263846	CG43707 gene product from transcrip	82.16652807	-0.205575493	0.954378726	82.16652807	-0.029093015	0.991444605
FBgn0263847	ncRNA	3.230030035	-0.253848708	0.95630182	3.230030035	-0.258275258	
FBgn0263848	CG43709 gene product from transcrip	3.080487174	0.040090971	0.99764662	3.080487174	0.159530913	
FBgn0263849	CG43710 gene product from transcrip	0.756691022	0.086382417		0.756691022	0	
FBgn0263850	CG43711 gene product from transcrip	0.167676683	0		0.167676683	0.129953448	
FBgn0263851	ncRNA	10.19260305	0.21605429	0.979231897	10.19260305	0.387292951	0.828048693
FBgn0263852	lethal (2) k12914	1050.420582	0.259585504	0.753828842	1050.420582	0.394846948	0.388426415
FBgn0263855	Bub1-related kinase	438.9354452	0.02944998	0.99764662	438.9354452	0.066265293	0.973524774
FBgn0263863	mitochondrial ribosomal protein L45	1097.473262	-0.023034935	0.998322595	1097.473262	0.075202336	0.961113238
FBgn0263864	Apaf-1-related-killer	273.9165742	-0.131561518	0.99157593	273.9165742	-0.357025021	0.792524256
FBgn0263866	ncRNA	0.139419282	-0.122978845		0.139419282	-0.119403538	
FBgn0263867	ncRNA	0			0		
FBgn0263868	ncRNA	0			0		
FBgn0263869	ncRNA	3.333468147	0.120667691	0.994497358	3.333468147	0.39157501	
FBgn0263870	ncRNA	2.212829336	-0.018501528	0.998322595	2.212829336	-0.271277123	
FBgn0263871	ncRNA	0.784087099	0.262838675		0.784087099	0.03056338	
FBgn0263872	ncRNA	0.701339196	0.018895096		0.701339196	0.070381374	
FBgn0263873	sickie	713.456089	0.201143273	0.893490961	713.456089	-0.053849269	0.973807349
FBgn0263874	ncRNA	0.351502576	0.161356617		0.351502576	0	
FBgn0263911	Cytochrome c oxidase subunit VIII	4659.943543	0.275142806	0.900180886	4659.943543	0.318924457	0.770169252
FBgn0263916	Equilibrative nucleoside transporter 2	1880.746294	0.151705374	0.961442281	1880.746294	0.42276351	0.397713242
FBgn0263929	javelin-like	1744.021885	0.038059122	0.995584438	1744.021885	0.09494588	0.951640396
FBgn0263930	division abnormally delayed	2421.341772	0.139557805	0.978641661	2421.341772	0.173195395	0.900999788
FBgn0263933	CG4063 gene product from transcript	1505.629341	-0.053035323	0.994497358	1505.629341	-0.054228689	0.971833087
FBgn0263934	espinas	795.7586191	0.251804859	0.822926382	795.7586191	-0.132462724	0.91558304
FBgn0263967	ncRNA	44.45361601	-0.653327808	0.415593704	44.45361601	-0.446535551	0.673843121
FBgn0263968	no-on-and-no-off transient C	1154.229465	0.166660521	0.956414448	1154.229465	0.225597667	0.806020939
FBgn0263970	ncRNA	3.892040006	-0.020050417	0.998322595	3.892040006	-0.16491875	
FBgn0263971	CG43725 gene product from transcrip	0.744308097	-0.074666325		0.744308097	0.018639788	
FBgn0263973	javelin	703.7800883	0.180311117	0.973639317	703.7800883	-0.273015951	0.829178937
FBgn0263974	CG43726 gene product from transcrip	24.01518723	0.121602029	0.994497358	24.01518723	0.578756869	0.651795237

[illegible]

FBgn0264833	ncRNA	2.064397277	0.246466043	0.953941411	2.064397277	0.565868426		2.064397277	-0.038858192	0.974417377
FBgn0264834	ncRNA	124.1929857	-0.431028613	0.844755814	124.1929857	0.017278437	0.997246866	124.1929857	0.076930907	0.953962511
FBgn0264835	ncRNA	9.319146349	0.722192589	0.534831227	9.319146349	0.528393323	0.70192988	9.319146349	-0.43763204	0.64632587
FBgn0264836	CG44044 gene product from transcrip	0			0			0		
FBgn0264837	ncRNA	0			0			0		
FBgn0264838	ncRNA	11.03854696	-0.057090078	0.997003712	11.03854696	-0.153252105	0.958001614	11.03854696	0.509070982	0.57224608
FBgn0264839	ncRNA	8.928028268	0.396089668	0.902357407	8.928028268	0.560647309	0.672894434	8.928028268	-0.613055162	0.46179582
FBgn0264840	ncRNA	0.221386409	0.173816599		0.221386409	0		0.221386409	-0.174835842	
FBgn0264841	ncRNA	0			0			0		
FBgn0264842	ncRNA	0.060151179	-0.097832662		0.060151179	-0.093256207		0.060151179	0	
FBgn0264843	ncRNA	0			0			0		
FBgn0264844	ncRNA	0			0			0		
FBgn0264845	Thioester-containing protein 5	7.859705013	-0.118828198	0.994497358	7.859705013	-0.48484329	0.728422782	7.859705013	-0.018570358	0.990821201
FBgn0264848	vihar	725.1340847	-0.101311518	0.979231897	725.1340847	-0.04809042	0.974305506	725.1340847	0.368057581	0.273164913
FBgn0264855	Adaptor Protein complex 2, alpha subu	3307.466966	-0.154336347	0.944197659	3307.466966	0.074365597	0.951640396	3307.466966	0.256775201	0.504688754
FBgn0264857	iab-8 non-coding RNA	61.47380564	0.059393386	0.995584438	61.47380564	0.183768189	0.933048047	61.47380564	0.197787249	0.840711831
FBgn0264862	ncRNA	0.413570335	-0.031376036		0.413570335	0.108508797		0.413570335	0	
FBgn0264863	ncRNA	5.300666024	0.237670596	0.963501097	5.300666024	0.249294758		5.300666024	-0.296913822	0.724611683
FBgn0264864	ncRNA	1.07772956	0.218668719		1.07772956	0.091447826		1.07772956	-0.245915463	
FBgn0264865	ncRNA	1.060097671	0.082377043		1.060097671	-0.05778308		1.060097671	-0.17761687	
FBgn0264866	ncRNA	0			0			0		
FBgn0264867	ncRNA	0.112641396	0		0.112641396	0		0.112641396	0	
FBgn0264868	ncRNA	0.221386409	0.173816599		0.221386409	0		0.221386409	-0.174835842	
FBgn0264869	ncRNA	0.213214751	-0.032560156		0.213214751	-0.096443753		0.213214751	-0.068579174	
FBgn0264870	ncRNA	1.170360389	0.228679122		1.170360389	-0.009508248		1.170360389	-0.176475683	
FBgn0264871	ncRNA	1.375745206	0.104910127		1.375745206	-0.191942636		1.375745206	-0.201294775	0.747534126
FBgn0264872	ncRNA	0.116471877	-0.071974357		0.116471877	-0.068468588		0.116471877	0	
FBgn0264873	ncRNA	0.145437648	0.126400319		0.145437648	0		0.145437648	-0.128839	
FBgn0264874	ncRNA	15.32096974	-0.089059142	0.9945211	15.32096974	0.430881863	0.797044857	15.32096974	0.227475516	0.844125968
FBgn0264875	ncRNA	1.121333341	0.018897408		1.121333341	0.299532167		1.121333341	-0.04999271	
FBgn0264876	ncRNA	3.056632486	0.011414042	0.998322595	3.056632486	0.281923864		3.056632486	-0.289012191	0.700424932
FBgn0264877	ncRNA	2.172444358	-0.206450657	0.96419728	2.172444358	-0.304517047		2.172444358	0.09091834	0.925558106
FBgn0264878	ncRNA	18.03716443	0.163679409	0.991146172	18.03716443	0.232272934	0.918961524	18.03716443	0.279928414	0.79058447
FBgn0264879	ncRNA	4.275705218	-0.223094831	0.969626359	4.275705218	-0.372000453		4.275705218	0.007203656	0.995115004
FBgn0264880	ncRNA	0.07379547	0.108851729		0.07379547	0		0.07379547	-0.111773044	
FBgn0264881	CG44072 gene product from transcrip	0.514509874	-0.063283585		0.514509874	-0.105227972		0.514509874	-0.047902048	
FBgn0264882	ncRNA	0.221386409	0.173816599		0.221386409	0		0.221386409	-0.174835842	
FBgn0264883	ncRNA	0.198065639	0		0.198065639	0.166537523		0.198065639	0	
FBgn0264884	ncRNA	0.646412029	0.087043202		0.646412029	0.084815109		0.646412029	-0.131748707	
FBgn0264885	ncRNA	0.07379547	0.108851729		0.07379547	0		0.07379547	-0.111773044	
FBgn0264886	ncRNA	0.060151179	-0.097832662		0.060151179	-0.093256207		0.060151179	0	
FBgn0264887	ncRNA	1.478766587	0.167135832		1.478766587	-0.122612982		1.478766587	-0.301038211	0.661591938
FBgn0264888	pseudo	0.6114982	0.049259734		0.6114982	0.104415116		0.6114982	-0.049977303	
FBgn0264889	ncRNA	0			0			0		
FBgn0264890	ncRNA	0.370893927	0.043549098		0.370893927	0.155346787		0.370893927	-0.044466432	
FBgn0264891	ncRNA	0.232979467	0.009382506		0.232979467	0.025091505		0.232979467	-0.059447097	
FBgn0264892	ncRNA	0.252060678	-0.086908936		0.252060678	-0.084641062		0.252060678	0	
FBgn0264893	ncRNA	0			0			0		
FBgn0264894	CG44085 gene product from transcrip	5535.293654	-0.251847827	0.921703858	5535.293654	-0.288407085	0.798682004	5535.293654	-0.069284374	0.939512066
FBgn0264895	Rap GTPase activating protein 1	923.2130695	0.118149301	0.987862946	923.2130695	0.160790352	0.91558304	923.2130695	0.166114379	0.81448245
FBgn0264896	ncRNA	0			0			0		
FBgn0264897	CG44088 gene product from transcrip	1.638854136	-0.158662537	0.978259505	1.638854136	-0.20353813		1.638854136	-0.136599276	0.870502422
FBgn0264898	ncRNA	0			0			0		
FBgn0264899	CG44090 gene product from transcrip	0.147590939	0.153120924		0.147590939	0		0.147590939	-0.15413487	
FBgn0264900	ncRNA	0.813952059	0.096718025		0.813952059	0.067331561		0.813952059	-0.170716845	
FBgn0264901	ncRNA	5.735903821	0.008215571	0.998322595	5.735903821	0.08765299		5.735903821	-0.329844674	0.706917934
FBgn0264902	ncRNA	5.99992551	0.130421704	0.994497358	5.99992551	-0.113583559		5.99992551	-0.122776121	0.926433687
FBgn0264903	CG44094 gene product from transcrip	8.119977926	-1.208829762	0.082693937	8.119977926	-1.075526451	0.151843689	8.119977926	1.006002424	0.124644942
FBgn0264904	ncRNA	12.82158237	0.564451322	0.738603464	12.82158237	1.060632876	0.178513556	12.82158237	-0.742992277	0.334482065
FBgn0264905	ncRNA	1.002561969	-0.257955062		1.002561969	-0.251165995		1.002561969	0.173217956	

[illegible]

FBgn0265537	ncRNA	0			0			0
FBgn0265538	ncRNA	0			0			0
FBgn0265539	ncRNA	0			0			0
FBgn0265540	ncRNA	2.456764736	0.043132178	0.995584438	2.456764736	-0.038101703		2.456764736
FBgn0265541	pseudo	1.408318863	0.099035666		1.408318863	0.117482292		1.408318863
FBgn0265542	ncRNA	3.441588732	-0.067987172	0.995120986	3.441588732	0.155537857		3.441588732
FBgn0265543	ncRNA	0.295181878	0.186873145		0.295181878	0		0.295181878
FBgn0265544	pseudo	0.07379547	0.108851729		0.07379547	0		0.07379547
FBgn0265545	ncRNA	0.882880914	-0.015340955		0.882880914	0.046943625		0.882880914
FBgn0265546	ncRNA	2.829394589	-0.008994596	0.998322595	2.829394589	-0.008812623		2.829394589
FBgn0265547	ncRNA	0.221386409	0.173816599		0.221386409	0		0.221386409
FBgn0265548	ncRNA	0.147590939	0.153120924		0.147590939	0		0.147590939
FBgn0265549	ncRNA	0			0			0
FBgn0265550	ncRNA	0			0			0
FBgn0265551	ncRNA	0			0			0
FBgn0265574	Cell division cycle 5 ortholog	660.3727548	0.080850097	0.99157593	660.3727548	0.269837272	0.684990219	660.3727548
FBgn0265575	CG44402 gene product from transcrip	2258.237007	0.499004387	0.68934315	2258.237007	0.126055345	0.956526474	2258.237007
FBgn0265576	CG44403 gene product from transcrip	4.316899834	0.384033004	0.822406497	4.316899834	0.397315044		4.316899834
FBgn0265577	ncRNA	181.9797408	0.565512244	0.67926883	181.9797408	-0.210358823	0.925047855	181.9797408
FBgn0265578	CG44405 gene product from transcrip	2.309389868	0.100056795	0.994497358	2.309389868	-0.011134072		2.309389868
FBgn0265579	CG44406 gene product from transcrip	0			0			0
FBgn0265580	ncRNA	78.11227064	-0.174071197	0.987458415	78.11227064	0.340847436	0.835840792	78.11227064
FBgn0265581	ncRNA	0			0			0
FBgn0265582	ncRNA	0.056320698	0		0.056320698	0		0.056320698
FBgn0265583	ncRNA	0			0			0
FBgn0265584	ncRNA	0.394178984	0.07821582		0.394178984	-0.029430995		0.394178984
FBgn0265585	ncRNA	0.440109455	0.071715129		0.440109455	0		0.440109455
FBgn0265586	ncRNA	0.139419282	-0.122978845		0.139419282	-0.119403538		0.139419282
FBgn0265587	ncRNA	3.029387569	-0.483818425	0.47698125	3.029387569	-0.504584614		3.029387569
FBgn0265588	ncRNA	0			0			0
FBgn0265589	ncRNA	0			0			0
FBgn0265590	ncRNA	253.7574177	0.495462356	0.373755736	253.7574177	0.270330616	0.769448057	253.7574177
FBgn0265591	ncRNA	0.147590939	0.153120924		0.147590939	0		0.147590939
FBgn0265592	ncRNA	0.260232335	0.102358155		0.260232335	0		0.260232335
FBgn0265593	ncRNA	0.905631379	0.183469702		0.905631379	0.042877736		0.905631379
FBgn0265594	ncRNA	0.936431245	-0.125836165		0.936431245	-0.28737839		0.936431245
FBgn0265595	CG44422 gene product from transcrip	85.32441335	0.135610539	0.989856236	85.32441335	0.149804611	0.942284238	85.32441335
FBgn0265596	ncRNA	12.84661191	-0.566413046	0.736375381	12.84661191	-0.185642023	0.944894567	12.84661191
FBgn0265597	radish	97.65982561	0.32467026	0.874992083	97.65982561	0.329857362	0.792524256	97.65982561
FBgn0265598	Beadex	426.4227659	0.239166986	0.953305045	426.4227659	0.192415237	0.91558304	426.4227659
FBgn0265605	Ras-related protein interacting with calı	1124.739923	-0.141331917	0.929739221	1124.739923	-0.038414627	0.978973038	1124.739923
FBgn0265606	Proteasome alpha4 subunit, Testis-spe	1.383293202	0.354952094		1.383293202	0		1.383293202
FBgn0265607	CG12621 gene product from transcrip	0			0			0
FBgn0265608	CG44426 gene product from transcrip	0.060151179	-0.097832662		0.060151179	-0.093256207		0.060151179
FBgn0265609	ncRNA	0.060151179	-0.097832662		0.060151179	-0.093256207		0.060151179
FBgn0265610	CG44428 gene product from transcrip	2.267219679	0.291930872	0.929926159	2.267219679	-0.091938794		2.267219679
FBgn0265611	ncRNA	56.81916295	-0.019143763	0.998322595	56.81916295	0.071445009	0.98515	56.81916295
FBgn0265612	ncRNA	0.241472153	0.06444487		0.241472153	0.110367284		0.241472153
FBgn0265613	ncRNA	2.097770285	0.081585338	0.994497358	2.097770285	-0.106282611		2.097770285
FBgn0265614	ncRNA	0			0			0
FBgn0265623	Suppressor of zeste 2	468.287062	-0.119241075	0.979231897	468.287062	0.077939956	0.961567894	468.287062
FBgn0265625	CG44433 gene product from transcrip	0.850596092	0.1907967		0.850596092	0		0.850596092
FBgn0265626	CG44434 gene product from transcrip	0			0			0
FBgn0265627	CG44435 gene product from transcrip	0			0			0
FBgn0265629	CG44437 gene product from transcrip	6.004959782	0.014825291	0.998322595	6.004959782	-0.077777789		6.004959782
FBgn0265630	strawberry notch	703.4168012	-0.053138673	0.994497358	703.4168012	-0.041161652	0.984900211	703.4168012
FBgn0265631	CG44438 gene product from transcrip	7.28090649	0.321059331	0.949623474	7.28090649	0.445684904	0.770169252	7.28090649
FBgn0265632	CG44439 gene product from transcrip	0.908334279	-0.057844787		0.908334279	-6.98E-05		0.908334279
FBgn0265633	ncRNA	11.72916377	0.007211903	0.998322595	11.72916377	0.724968473	0.510233305	11.72916377
FBgn0265634	ncRNA	6.320035359	0.284634426	0.956325514	6.320035359	0.360255228	0.825115861	6.320035359
FBgn0265635	ncRNA	9.19229041	0.465775876	0.846500794	9.19229041	0.472924489	0.764601941	9.19229041
FBgn0265636	ncRNA	22.52523826	-0.385221136	0.899317399	22.52523826	-0.026679189	0.996076098	22.52523826
FBgn0265637	ncRNA	1.885634076	-0.315388882	0.902357407	1.885634076	-0.38339886		1.885634076
FBgn0265638	ncRNA	1.64749513	0.083324226	0.994497358	1.64749513	-0.179935061		1.64749513
FBgn0265639	ncRNA	0			0			0
FBgn0265640	ncRNA	0.289798087	0		0.289798087	0		0.289798087
FBgn0265641	ncRNA	0.313923393	-0.027739727		0.313923393	-0.074289342		0.313923393
FBgn0265642	ncRNA	0			0			0
FBgn0265643	ncRNA	0			0			0
FBgn0265644	ncRNA	5.157721483	0.249356785	0.967901085	5.157721483	0.205480839		5.157721483
FBgn0265645	ncRNA	0			0			0
FBgn0265646	ncRNA	0.736152415	-0.035011407		0.736152415	0.012625494		0.736152415
FBgn0265648	ncRNA	1.402113921	0.233025405		1.402113921	0		1.402113921
FBgn0265649	ncRNA	0.056320698	0		0.056320698	0		0.056320698
FBgn0265650	ncRNA	0.244470467	0.097680939		0.244470467	0.07908414		0.244470467
FBgn0265651	ncRNA	5.333156899	0.000829162	0.999781966	5.333156899	0.425379516		5.333156899
FBgn0265652	ncRNA	0			0			0
FBgn0265653	ncRNA	0.07379547	0.108851729		0.07379547	0		0.07379547
FBgn0265654	ncRNA	0.325856147	-0.026744466		0.325856147	-0.070509499		0.325856147
FBgn0265655	ncRNA	0			0			0
FBgn0265656	ncRNA	0.12877022	0		0.12877022	0		0.12877022
FBgn0265657	ncRNA	0			0			0
FBgn0265658	ncRNA	3.958192876	-0.081567758	0.9945211	3.958192876	0.310975448		3.958192876
FBgn0265659	ncRNA	11.74134856	1.362649676	0.040571248	11.74134856	1.227909079	0.08551634	11.74134856
FBgn0265660	ncRNA	2.291809279	0.129584669	0.992706654	2.291809279	0.406895039		2.291809279
FBgn0265661	ncRNA	48.85945134	0.007503793	0.998322595	48.85945134	0.324855536	0.845624469	48.85945134
FBgn0265662	ncRNA	3.46381633	-0.45215654	0.805580095	3.46381633	0.040088183		3.46381633
FBgn0265663	ncRNA	0			0			0
FBgn0265664	ncRNA	0.370242373	0.043205374		0.370242373	0.080524446		0.370242373
[illegible]	[illegible]	[illegible]	[illegible]	[illegible]	[illegible]	[illegible]	[illegible]	[illegible]

FBgn0265982	ncRNA	3.36679045	0.235526514	0.953755212	3.36679045	-0.067859701		3.36679045
FBgn0265983	ncRNA	0.488088731	-0.039819533		0.488088731	0.050079058		0.488088731
FBgn0265984	ncRNA	1.742177168	-0.104750986	0.994497358	1.742177168	-0.027752076		1.742177168
FBgn0265985	ncRNA	6.002327374	-0.129446988	0.994497358	6.002327374	-0.098332954		6.002327374
FBgn0265986	ncRNA	0			0			0
FBgn0265987	ncRNA	4.436478524	0.107830099	0.994497358	4.436478524	-0.069221538		4.436478524
FBgn0265988	mauve	1646.688424	-0.362921349	0.498035621	1646.688424	-0.45828891	0.27078092	1646.688424
FBgn0265991	Z band alternatively spliced PDZ-motif p	14412.96384	0.232131847	0.931901041	14412.96384	0.4693574	0.511201392	14412.96384
FBgn0265992	ncRNA	0.07379547	0.108851729		0.07379547	0		0.07379547
FBgn0265993	ncRNA	0.349377819	0.078090042		0.349377819	0		0.349377819
FBgn0265994	ncRNA	1.296846258	0.385732743		1.296846258	-0.036545901		1.296846258
FBgn0265995	ncRNA	0			0			0
FBgn0265996	ncRNA	0.100708642	0		0.100708642	0		0.100708642
FBgn0265997	ncRNA	1.268727014	0.374318844		1.268727014	0.09873072		1.268727014
FBgn0265998	Darkener of apricot	2657.654846	-0.196624151	0.953305045	2657.654846	-0.292251916	0.75802194	2657.654846
FBgn0265999	ncRNA	0.36080569	0.054947807		0.36080569	-0.067252893		0.36080569
FBgn0266000	CG44774 gene product from transcrip	1563.870705	0.679589556	0.511717831	1563.870705	0.793123909	0.351397063	1563.870705
FBgn0266001	ncRNA	1.667979765	0.248346194	0.921622016	1.667979765	-0.001575974		1.667979765
FBgn0266002	ncRNA	8.081035032	-0.616249013	0.666450455	8.081035032	-0.439929	0.780244249	8.081035032
FBgn0266003	ncRNA	0.355333057	0.12412121		0.355333057	-0.033176389		0.355333057
FBgn0266004	ncRNA	0.130116168	0.082464995		0.130116168	0		0.130116168
FBgn0266005	ncRNA	375.922824	-0.100774724	0.993908998	375.922824	-0.261531068	0.828254838	375.922824
FBgn0266007	ncRNA	0			0			0
FBgn0266008	ncRNA	0.167676683	0		0.167676683	0.129953448		0.167676683
FBgn0266009	ncRNA	0.147590939	0.153120924		0.147590939	0		0.147590939
FBgn0266010	ncRNA	0.07379547	0.108851729		0.07379547	0		0.07379547
FBgn0266011	ncRNA	21.88784571	-0.407148564	0.886446899	21.88784571	-0.327389901	0.861850001	21.88784571
FBgn0266019	CG43154 gene product from transcrip	5718.254492	0.130436279	0.987458415	5718.254492	0.257066248	0.832797896	5718.254492
FBgn0266020	ncRNA	5.550172725	0.158483225	0.99157593	5.550172725	0.228380503		5.550172725
FBgn0266021	ncRNA	1.147272018	-0.131972691		1.147272018	-0.130986305		1.147272018
FBgn0266022	ncRNA	0			0			0
FBgn0266023	ncRNA	0			0			0
FBgn0266024	ncRNA	0			0			0
FBgn0266025	ncRNA	0.056320698	0		0.056320698	0		0.056320698
FBgn0266026	ncRNA	0.147619473	0		0.147619473	0		0.147619473
FBgn0266027	ncRNA	0.513163926	-0.106743675		0.513163926	-0.10377085		0.513163926
FBgn0266028	ncRNA	0.848893447	-0.065373031		0.848893447	-0.107211251		0.848893447
FBgn0266029	ncRNA	0.060151179	-0.097832662		0.060151179	-0.093256207		0.060151179
FBgn0266030	ncRNA	0.554833486	-0.049963412		0.554833486	0.026687275		0.554833486
FBgn0266031	ncRNA	0.205588827	0.047195087		0.205588827	-0.055327512		0.205588827
FBgn0266032	ncRNA	0.174504111	0.073112477		0.174504111	0		0.174504111
FBgn0266033	ncRNA	0.269535449	-0.029361293		0.269535449	-0.080898063		0.269535449
FBgn0266034	ncRNA	0			0			0
FBgn0266035	ncRNA	0			0			0
FBgn0266036	ncRNA	0.308381376	-0.075773916		0.308381376	-0.073698995		0.308381376
FBgn0266037	ncRNA	0.120302357	-0.141479015		0.120302357	-0.1379702		0.120302357
FBgn0266038	ncRNA	0.337650923	-0.070882919		0.337650923	-0.068938159		0.337650923
FBgn0266039	ncRNA	0.252060678	-0.086908936		0.252060678	-0.084641062		0.252060678
FBgn0266040	ncRNA	0			0			0
FBgn0266041	ncRNA	21.43859599	-0.143179258	0.994497358	21.43859599	-0.35772606	0.85011603	21.43859599
FBgn0266042	ncRNA	53.20777415	0.081671013	0.994497358	53.20777415	0.58187411	0.536148288	53.20777415
FBgn0266043	ncRNA	0.246652292	0		0.246652292	0.078781854		0.246652292
FBgn0266044	ncRNA	10.93414732	0.281330488	0.966088307	10.93414732	0.358136908	0.850061293	10.93414732
FBgn0266045	ncRNA	2.417870152	0.012970529	0.998322595	2.417870152	0.108730881		2.417870152
FBgn0266046	ncRNA	220.0851869	0.011118332	0.998322595	220.0851869	-0.287695674	0.815824306	220.0851869
FBgn0266047	CG44812 gene product from transcrip	4.452138381	0.315166682	0.949623474	4.452138381	0.400784236		4.452138381
FBgn0266048	ncRNA	1.551828491	-0.117026842		1.551828491	0.091615649		1.551828491
FBgn0266049	ncRNA	1.158897264	0.136229755		1.158897264	0.271930108		1.158897264
FBgn0266050	CG44815 gene product from transcrip	0.320412047	-0.039506658		0.320412047	-0.037444411		0.320412047
FBgn0266051	ncRNA	4.233465152	0.198201311	0.975999817	4.233465152	0.234732751		4.233465152
FBgn0266052	ncRNA	1.930622182	0.227038757	0.953941411	1.930622182	-0.188720134		1.930622182
FBgn0266053	Protein associated with topo II related -	1195.084509	0.044126143	0.995120986	1195.084509	0.274197928	0.770169252	1195.084509
FBgn0266064	Glycogen synthase	5994.342138	0.305296334	0.871091663	5994.342138	0.524060137	0.479376545	5994.342138
FBgn0266065	ncRNA	0			0			0
FBgn0266066	ncRNA	0.056320698	0		0.056320698	0		0.056320698
FBgn0266067	ncRNA	0			0			0
FBgn0266068	ncRNA	0			0			0
FBgn0266069	ncRNA	0.056320698	0		0.056320698	0		0.056320698
FBgn0266070	ncRNA	0.281537587	0.10133235		0.281537587	-0.039571732		0.281537587
FBgn0266071	ncRNA	0			0			0
FBgn0266072	ncRNA	13.6068044	0.362704449	0.929926159	13.6068044	-0.374676153	0.836795357	13.6068044
FBgn0266073	ribosomal RNA	4.581109549	0.27251133	0.961442281	4.581109549	0.66402564		4.581109549
FBgn0266074	ncRNA	0.112641396	0		0.112641396	0		0.112641396
FBgn0266076	mir-276a stem loop							
FBgn0266083	over compensating males	576.1399603	0.155004153	0.963501097	576.1399603	0.067769571	0.968777026	576.1399603
FBgn0266084	Formin homology 2 domain containing	2294.438309	0.045989508	0.994497358	2294.438309	-0.024602639	0.990025502	2294.438309
FBgn0266086	ncRNA	2.037410677	0.180238207	0.975861786	2.037410677	0.236604874		2.037410677
FBgn0266087	ncRNA	0.423738843	0		0.423738843	0.14870878		0.423738843
FBgn0266088	ncRNA	1.991329937	0.28617399	0.917640938	1.991329937	0.057816548		1.991329937
FBgn0266094	ncRNA	7.949035619	-0.401251706	0.898864856	7.949035619	-0.001600088	1	7.949035619
FBgn0266095	ncRNA	59.51145876	0.169780238	0.985645036	59.51145876	-0.530132335	0.638266824	59.51145876
FBgn0266096	ncRNA	4.059236761	0.183536512	0.987458415	4.059236761	-0.168186095		4.059236761
FBgn0266097	ncRNA	1.806085449	0.07329822	0.994497358	1.806085449	0.025087508		1.806085449
FBgn0266098	rugose	11.87607748	0.485427054	0.825494693	11.87607748	0.24652292	0.917887901	11.87607748
FBgn0266099	CG44836 gene product from transcrip	8.833468674	0.243005607	0.975861786	8.833468674	-0.372901186	0.83583436	8.833468674
FBgn0266100	CG44837 gene product from transcrip	161.154874	-0.228424992	0.953305045	161.154874	-0.358504847	0.735815428	161.154874
FBgn0266101	CG44838 gene product from transcrip	788.9069282	0.107116975	0.989856236	788.9069282	-0.033198354	0.989503484	788.9069282
FBgn0266102	pseudo	0			0			0
FBgn0266111	anastral spindle 3	211.1165489	0.067438561	0.994497358	211.1165489	0.072651108	0.960705837	211.1165489
FBgn0266114	ncRNA	0			0			0
FBgn0266115	ncRNA	3.859608875	0.185404892	0.985645036	3.859608875	0.030347275		3.859608875
FBgn0266116	ncRNA	0.983861235	-0.059049907		0.983861235	0.02342198		0.983861235
FBgn0266117	ncRNA	4.744358492	-0.154272331	0.988354972	4.744358492	-0.092021833		4.744358492
FBgn0266124	ghiberti	513.0849119	0.022300036	0.998322595	513.0849119	0.253302865	0.83316704	513.0849119
FBgn0266129	jim lovell	75.21840744	-0.397003789	0.726605599	75.21840744	-0.415899268	0.64241103	75.21840744
FBgn0266136	Guanylyl cyclase at 76C	4.330138495	0.084177095	0.994497358	4.330138495	0.037448185		4.330138495
FBgn0266137	Dopamine 1-like receptor 2	41.06118757	0.203354615	0.975044255	41.06118757	0.190953564	0.922400604	41.06118757
FBgn0266138	ncRNA	0.147590939	0.153120924		0.147590939	0		0.147590939
FBgn0266139	ncRNA	7.290591141	0.066656498	0.995584438	7.290591141	-0.072773901	0.985851007	7.290591141
FBgn0266140	ncRNA	0.072449522	0		0.072449522	0		0.072449522
FBgn0266141	ncRNA	0.561442446	0.061469627		0.561442446	0.0698585		0.561442446
FBgn0266142	ncRNA	0.944476704	-0.099628396		0.944476704	0.027905291		0.944476704
FBgn0266143	ncRNA	7.832412767	0.159012522	0.99157593	7.832412767	0.225604759	0.922352014	7.832412767
FBgn0266144	ncRNA	0.503381678	0.097806839		0.503381678	0.056653597		0.503381678
FBgn0266145	ncRNA	16.04844697	0.77787001	0.457940355	16.04844697	0.302656245	0.884956001	16.04844697
FBgn0266146	ncRNA	0.221414942	0.066564265		0.221414942	0		0.221414942
FBgn0266147	ncRNA	0.174504111	0.073112477		0.174504111	0		0.174504111
FBgn0266148	ncRNA	5.171727119	-0.342932337	0.936128798	5.171727119	-0.073478959		5.171727119
FBgn0266149	ncRNA	8.051551202	-0.184092208	0.988216049	8.051551202	-0.106354237	0.973807349	8.051551202
FBgn0266150	ncRNA	0.07379547	0.108851729		0.07379547	0		0.07379547
FBgn0266151	ncRNA	0			0			0
FBgn0266152	ncRNA	0			0			0
FBgn0266153	ncRNA	0.198065639	0		0.198065639	0.166537523		0.198065639
FBgn0266154	ncRNA	0.139419282	-0.122978845		0.139419282	-0.119403538		0.139419282
FBgn0266155	ncRNA	0.07379547	0.108851729		0.07379547	0		0.07379547
FBgn0266156	ncRNA	0.272019982	-0.126251713		0.272019982	-0.124035946		0.272019982
FBgn0266157	ncRNA	0.218694513	0.060854751		0.218694513	0		0.218694513
FBgn0266158	ncRNA	0.221386409	0.173816599		0.221386409	0		0.221386409
FBgn0266159	ncRNA	0			0			0
FBgn0266160	ncRNA	0.28031808	0		0.28031808	0.098202706		0.28031808
FBgn0266161	ncRNA	0.317691619	-0.036669891		0.317691619	-0.034694826		0.317691619
FBgn0266162	ncRNA	1.114910945	0.133527368		1.114910945	-0.187913308		1.114910945
FBgn0266163	CG44869 gene product from transcrip	0.144899044	0		0.144899044	0		0.144899044
FBgn0266164	ncRNA	0.147619473	0		0.147619473	0		0.147619473
FBgn0266165	ncRNA	0.07379547	0.108851729		0.07379547	0		0.07379547
FBgn0266166	ncRNA	0			0			0
FBgn0266167	ncRNA	41.83777192	0.389300241	0.892718858	41.83777192	0.4046983	0.797974284	41.83777192
FBgn0266168	ncRNA	34.17315958	-0.878718674	0.315050392	34.17315958	-0.487466769	0.729635084	34.17315958
FBgn0266169	ncRNA	0.44921427	0.074737253		0.44921427	0.040406314		0.44921427
FBgn0266170	CG44876 gene product from transcrip	0			0			0
FBgn0266171	CG44877 gene product from transcrip	0			0			0
FBgn0266172	CG44878 gene product from transcrip	0.099032819	0		0.099032819	0.123176119		0.099032819
FBgn0266173	CG44879 gene product from transcrip	0			0			0
FBgn0266174	CG44880 gene product from transcrip	0			0			0
FBgn0266175	CG44881 gene product from transcrip	0			0			0
FBgn0266176	CG44882 gene product from transcrip	0			0			0
FBgn0266177	CG44883 gene product from transcrip	0			0			0
FBgn0266178	CG44884 gene product from transcrip	0.357249636	-0.03620483		0.357249636	0.122238003		0.357249636
FBgn0266179	ncRNA	0.725201738	-0.089208309		0.725201738	-0.177865962		0.725201738
FBgn0266180	ncRNA	5.169508614	-0.319521722	0.936128798	5.169508614	-0.035211763		5.169508614
FBgn0266181	CASK regulatory gene	0			0			0
FBgn0266182	ncRNA	24.55760688	0.360013035	0.927059603	24.55760688	-0.053266585	0.990025502	24.55760688
FBgn0266183	ncRNA	41.51933716	0.125777552	0.993127879	41.51933716	-0.063689185	0.985624614	41.51933716
FBgn0266186	Vesicle-associated membrane protein 7	2504.899451	-0.335162915	0.660844341	2504.899451	-0.334258903	0.611478474	2504.899451
FBgn0266195	Trimethylguanosine synthase 1 ortholc	0			0			0
FBgn0266196	ncRNA	0			0			0
FBgn0266197	ncRNA	17.84885048	-0.235671773	0.975999817	17.84885048	0.11844615	0.971833087	17.84885048
FBgn0266198	ncRNA	1.351042775	-0.142573237		1.351042775	-0.233889632		1.351042775
FBgn0266199	ncRNA	8.595791145	-0.222321625	0.979231897	8.595791145	-0.28830381	0.895445286	8.595791145
FBgn0266200	ncRNA	9.95978981	-0.162259242	0.99157593	9.95978981	-0.225676623	0.926735231	9.95978981
FBgn0266201	ncRNA	0			0			0
FBgn0266202	ncRNA	0.815231823	0.010638421		0.815231823	-0.046701243		0.815231823
FBgn0266203	ncRNA	0			0			0
FBgn0266204	ncRNA	7.604233689	0.338194989	0.944197659	7.604233689	-0.062837962	0.986877529	7.604233689
FBgn0266205	ncRNA	0.757031542	-0.065695808		0.757031542	-0.093779792		0.757031542
FBgn0266206	ncRNA	0.702522989	0.024084405		0.702522989	-0.121604658		0.702522989
FBgn0266207	ncRNA	0.355094979	0		0.355094979	0.10810916		0.355094979
FBgn0266208	ncRNA	2.017435546	0.051306698	0.995584438	2.017435546	0.129253597		2.017435546
FBgn0266209	ncRNA	1.618292622	0.206038291	0.965076657	1.618292622	-0.114048016		1.618292622
FBgn0266210	ncRNA	1.391832196	-0.162456835		1.391832196	-0.254208268		1.391832196
FBgn0266211	pseudo	26.81321061	-0.742040025	0.42048989	26.81321061	-0.319269242	0.852772987	26.81321061
FBgn0266212	ncRNA	3.70030287	-0.229033	0.966157244	3.70030287	-0.297360105		3.70030287
FBgn0266213	ncRNA	0.112641396	0		0.112641396	0		0.112641396
FBgn0266214	ncRNA	0.639249431	-0.026064599		0.639249431	-0.011778251		0.639249431
FBgn0266215	ncRNA	0			0			0
FBgn0266216	ncRNA	11.19670098	0.142485826	0.994497358	11.19670098	-0.036002281	0.994479311	11.19670098
FBgn0266217	ncRNA	5.744353242	0.174031738	0.989856236	5.744353242	-0.284049327		5.744353242
FBgn0266218	ncRNA	0.537996509	0.04044733		0.537996509	0		0.537996509
FBgn0266219	ncRNA	3.653718876	-0.330481072	0.926642056	3.653718876	-0.445590597		3.653718876
FBgn0266220	ncRNA	0			0			0
FBgn0266221	ncRNA	0			0			0
FBgn0266222	ncRNA	17.73706889	-0.088393526	0.9945211	17.73706889	-0.202878359	0.938496787	17.73706889
FBgn0266223	ncRNA	314.2343152	-1.159014121	0.020853534	314.2343152	-0.365334608	0.771825396	314.2343152
FBgn0266224	ncRNA	1.554345151	-0.312927598		1.554345151	-0.152349416		1.554345151
FBgn0266225	ncRNA	6.898180357	0.640448348	0.650200631	6.898180357	0.024957963	0.996947062	6.898180357
FBgn0266226	ncRNA	0.07379547	0.108851729		0.07379547	0		0.07379547
FBgn0266227	ncRNA	23.20992642	0.723180059	0.479465274	23.20992642	1.026401203	0.157803898	23.20992642
FBgn0266228	ncRNA	16.49522297	0.776544656	0.469259474	16.49522297	-0.068242946	0.986877529	16.49522297
FBgn0266229	ncRNA	0.060151179	-0.097832662		0.060151179	-0.093256207		0.060151179
FBgn0266230	ncRNA	0			0			0
FBgn0266231	ncRNA	0.07379547	0.108851729		0.07379547	0		0.07379547
FBgn0266232	ncRNA	0.289798087	0		0.289798087	0		0.289798087
FBgn0266233	ncRNA	0.281566121	0.008801825		0.281566121	-0.041534417		0.281566121
FBgn0266234	ncRNA	2.743123537	-0.319291237	0.898864856	2.743123537	-0.269841412		2.743123537
FBgn0266235	ncRNA	1.247835196	0.246314727		1.247835196	0		1.247835196
FBgn0266236	ncRNA	0			0			0
FBgn0266237	ncRNA	22.03892487	0.06325823	0.995584438	22.03892487	0.089317809	0.980691994	22.03892487
FBgn0266238	ncRNA	3.869193114	-0.08477351	0.994497358	3.869193114	0.235246241		3.869193114
FBgn0266239	ncRNA	0.07379547	0.108851729		0.07379547	0		0.07379547
FBgn0266240	ncRNA	0			0			0
FBgn0266241	ncRNA	3.704052722	-0.084541723	0.994497358	3.704052722	-0.395584396		3.704052722
FBgn0266242	ncRNA	0			0			0
FBgn0266243	ncRNA	0.569545161	-0.049752609		0.569545161	-0.016156118		0.569545161
FBgn0266244	ncRNA	0.190267346	0.010068336		0.190267346	-0.050960481		0.190267346
FBgn0266245	ncRNA	0			0			0
FBgn0266246	ncRNA	0.07379547	0.108851729		0.07379547	0		0.07379547
FBgn0266247	ncRNA	0			0			0
FBgn0266248	ncRNA	0.207742118	0.067675148		0.207742118	-0.049276071		0.207742118
FBgn0266249	ncRNA	0.221386409	0.173816599		0.221386409	0		0.221386409
FBgn0266250	ncRNA	2.442597875	-0.04886488	0.995904623	2.442597875	0.172306664		2.442597875
FBgn0266251	ncRNA	1.066004634	-0.18874186		1.066004634	-0.171371764		1.066004634
FBgn0266252	ncRNA	20.86734918	-0.09418079	0.994497358	20.86734918	0.133200975	0.965276384	20.86734918
FBgn0266253	ncRNA	5.12613369	0.753809169	0.237258737	5.12613369	0.071216258		5.12613369
FBgn0266254	ncRNA	0			0			0
FBgn0266255	ncRNA	0.221386409	0.173816599		0.221386409	0		0.221386409
FBgn0266256	ncRNA	0.617224539	0		0.617224539	0.113335586		0.617224539
FBgn0266257	ncRNA	0			0			0
FBgn0266258	ncRNA	10.7845036	-0.279223516	0.966157244	10.7845036	-0.662953119	0.584053378	10.7845036
FBgn0266259	CG44954 gene product from transcrip	0.056320698	0		0.056320698	0		0.056320698
FBgn0266260	ncRNA	4.938170211	0.525787925	0.734424276	4.938170211	0.090225946		4.938170211
FBgn0266261	CG44956 gene product from transcrip	3129.312343	0.851613558	0.024833986	3129.312343	0.462207068	0.455161017	3129.312343
FBgn0266262	ncRNA	6.658187711	-0.04283513	0.998322595	6.658187711	-0.272972966	0.90480014	6.658187711
FBgn0266263	ncRNA	0.060151179	-0.097832662		0.060151179	-0.093256207		0.060151179
FBgn0266264	ncRNA	0.07379547	0.108851729		0.07379547	0		0.07379547
FBgn0266268	Ferrochelatase	953.4407633	0.087364389	0.99157593	953.4407633	0.06323104	0.971833087	953.4407633
FBgn0266275	ncRNA	2.466167844	0.203037445	0.954654825	2.466167844	0.057794394		2.466167844
FBgn0266276	ncRNA	1.231681333	0.073254137		1.231681333	0.159439084		1.231681333
FBgn0266277	ncRNA	0.883773606	-0.147510571		0.883773606	-0.211393754		0.883773606
FBgn0266278	ncRNA	0			0			0
FBgn0266279	ncRNA	16.68062298	-0.419276577	0.880088833	16.68062298	-0.019423684	0.997246866	16.68062298
FBgn0266280	ncRNA	1.084264529	-0.057618738		1.084264529	0.103498471		1.084264529
FBgn0266281	ncRNA	1.094399973	-0.087789664		1.094399973	-0.083711615		1.094399973
FBgn0266282	java no jive	354.0577913	-0.084157912	0.994497358	354.0577913	-0.091721727	0.95955656	354.0577913
FBgn0266284	Nucleostemin ortholog (H. sapiens) 3	702.5354208	0.10161196	0.979231897	702.5354208	0.113448431	0.920269865	702.5354208
FBgn0266302	ncRNA	0.130116168	0.082464995		0.130116168	0		0.130116168
FBgn0266303	ncRNA	1.390250906	0.050769584		1.390250906	0.166352816		1.390250906
FBgn0266304	ncRNA	0.167676683	0		0.167676683	0.129953448		0.167676683
FBgn0266305	ncRNA	2.56683065	-0.207720673	0.973928666	2.56683065	-0.001077822		2.56683065
FBgn0266306	ncRNA	0.412820873	-0.061912805		0.412820873	-0.060130029		0.412820873
FBgn0266307	ncRNA	0			0			0
FBgn0266308	ncRNA	2.86752566	0.120818641	0.993509441	2.86752566	0.14709892		2.86752566
FBgn0266309	ncRNA	9.913710897	0.479134	0.822406497	9.913710897	0.530796163	0.689849402	9.913710897
FBgn0266310	ncRNA	11.18474027	-0.26093002	0.971565969	11.18474027	0.147358177	0.959619247	11.18474027
FBgn0266311	ncRNA	0.618482784	0.035986512		0.618482784	0.021944835		0.618482784
FBgn0266312	ncRNA	19.04768103	0.309192365	0.956325514	19.04768103	0.052023556	0.990025502	19.04768103
FBgn0266313	ncRNA	22.62922508	-0.196927832	0.979742185	22.62922508	-0.068224207	0.98613036	22.62922508
FBgn0266314	ncRNA	0.345685111	0		0.345685111	0.116013275		0.345685111
FBgn0266315	ncRNA	4.636378769	0.301569231	0.944197659	4.636378769	0.248205718		4.636378769
FBgn0266316	ncRNA	40.26969112	0.174029463	0.979841869	40.26969112	0.484578423	0.653469512	40.26969112
FBgn0266317	ncRNA	0			0			0
FBgn0266318	ncRNA	0.499049347	-0.031385368		0.499049347	-0.099583157		0.499049347
FBgn0266319	ncRNA	3.188108927	-0.247124786	0.949774689	3.188108927	0.222260257		3.188108927
FBgn0266320	ncRNA	0			0			0
FBgn0266321	ncRNA	0			0			0
FBgn0266322	ncRNA	99.81155551	-0.190050403	0.975999817	99.81155551	-0.257734332	0.871207708	99.81155551
FBgn0266323	ncRNA	0			0			0
FBgn0266324	ncRNA	0			0			0
FBgn0266325	ncRNA	0			0			0
FBgn0266326	ncRNA	1.350874454	0.123161293		1.350874454	-0.058685924		1.350874454
FBgn0266327	ncRNA	18.06144944	0.459878861	0.846956878	18.06144944	0.040853751	0.993170566	18.06144944
FBgn0266328	ncRNA	59.94047766	-0.192010541	0.979231897	59.94047766	0.332694651	0.824735368	59.94047766
FBgn0266329	ncRNA	1.461897731	0.23077391		1.461897731	0.586582065		1.461897731
FBgn0266330	ncRNA	0			0			0
FBgn0266331	ncRNA	0.056320698	0		0.056320698	0		0.056320698
FBgn0266346	Cyclic nucleotide-gated ion channel sul	233.064865	-0.200442041	0.9379015	233.064865	-0.150072685	0.914502763	233.064865
FBgn0266347	nicotinic Acetylcholine Receptor alpha4	97.40373164	-0.214046544	0.971565969	97.40373164	0.293772503	0.849387814	97.40373164
FBgn0266348	ncRNA	2.20126437	0.269280292	0.925140457	2.20126437	0.257168022		2.20126437
FBgn0266349	ncRNA	69.15308926	-0.631836618	0.400863957	69.15308926	-0.655158044	0.362563542	69.15308926
FBgn0266350	ncRNA	2.62530596	0.259026344	0.946165413	2.62530596	0.264175612		2.62530596
FBgn0266351	ncRNA	3.767374622	-0.027058195	0.998322595	3.767374622	0.678934558		3.767374622
FBgn0266352	ncRNA	0			0			0
FBgn0266354	CG45002 gene product from transcrip	66.56383995	0.198171489	0.971565969	66.56383995	0.082870298	0.973807349	66.56383995
FBgn0266355	ncRNA	0			0			0
FBgn0266356	ncRNA	5.48823055	0.199613965	0.985645036	5.48823055	-0.385582414		5.48823055
FBgn0266357	ncRNA	0			0			0
FBgn0266358	ncRNA	0.295181878	0.186873145		0.295181878	0		0.295181878
FBgn0266359	ncRNA	6.094032132	0.070563939	0.995584438	6.094032132	-0.251696423		6.094032132
FBgn0266360	ncRNA	0.213214751	-0.032560156		0.213214751	-0.096443753		0.213214751
FBgn0266361	ncRNA	1.979002233	0.112285735	0.988478465	1.979002233	0.285151049		1.979002233
FBgn0266362	ncRNA	0			0			0
FBgn0266363	CG45011 gene product from transcrip	0.174504111	0.073112477		0.174504111	0		0.174504111
FBgn0266364	CG45012 gene product from transcrip	0.147619473	0		0.147619473	0		0.147619473
FBgn0266365	ncRNA	0			0			0
FBgn0266366	ncRNA	0.139419282	-0.122978845		0.139419282	-0.119403538		0.139419282
FBgn0266369	Microsomal triacylglycerol transfer pro	3415.804018	0.569884465	0.045145774	3415.804018	0.797923479	0.000931665	3415.804018
FBgn0266371	ncRNA	0.07379547	0.108851729		0.07379547	0		0.07379547
FBgn0266372	ncRNA	0.357249636	-0.03620483		0.357249636	0.122238003		0.357249636
FBgn0266376	ncRNA	419.4741487	-0.002094801	0.999629143	419.4741487	-0.246497906	0.886645978	419.4741487
FBgn0266377	Phosphodiesterase 8	1324.479254	0.133335177	0.911691477	1324.479254	0.275906879	0.437690716	1324.479254
FBgn0266378	ncRNA	0.056320698	0		0.056320698	0		0.056320698
FBgn0266379	ncRNA	0.788887077	0.273186852		0.788887077	0.260457122		0.788887077
FBgn0266380	ncRNA	0			0			0
FBgn0266381	ncRNA	0.360969511	0		0.360969511	0		0.360969511
FBgn0266382	ncRNA	0.897180283	-0.02874958		0.897180283	0.014127741		0.897180283
FBgn0266383	ncRNA	0.211868803	-0.099154722		0.211868803	-0.096714836		0.211868803
FBgn0266384	ncRNA	2.318380074	0.022768161	0.998322595	2.318380074	0.173378841		2.318380074
FBgn0266388	ncRNA	3.739410902	0.037255662	0.998322595	3.739410902	-0.142361396		3.739410902
FBgn0266389	ncRNA	0.893635768	-0.021658901		0.893635768	0.075264946		0.893635768
FBgn0266390	ncRNA	1.20033382	0.168053233		1.20033382	-0.047096514		1.20033382
FBgn0266391	ncRNA	0.100708642	0		0.100708642	0		0.100708642
FBgn0266392	ncRNA	0.248299581	0.109758614		0.248299581	0		0.248299581
FBgn0266393	pseudo	0			0			0
FBgn0266394	ncRNA	3.9186174	0.274751715	0.956083993	3.9186174	0.149665587		3.9186174
FBgn0266395	ncRNA	1.653433456	-0.292610908	0.825494693	1.653433456	-0.210106098		1.653433456
FBgn0266396	ncRNA	34.1203114	-0.048220406	0.997459641	34.1203114	0.06006235	0.9872372	34.1203114
FBgn0266397	ncRNA	0.850508433	-0.093063615		0.850508433	-0.105359066		0.850508433
FBgn0266398	ncRNA	0.481279048	0.077091587		0.481279048	0.018907875		0.481279048
FBgn0266399	ncRNA	4.06955854	-0.086737036	0.994497358	4.06955854	-0.032141482		4.06955854
FBgn0266400	ncRNA	0			0			0
FBgn0266401	ncRNA	0			0			0
FBgn0266402	ncRNA	0			0			0
FBgn0266403	ncRNA	2.33950825	-0.178623718	0.967338745	2.33950825	-0.298173386		2.33950825
FBgn0266404	ncRNA	0.112641396	0		0.112641396	0		0.112641396
FBgn0266405	ncRNA	89.34093263	1.6310977	0.005950645	89.34093263	0.242416875	0.918961524	89.34093263
FBgn0266406	ncRNA	0			0			0
FBgn0266407	ncRNA	2.424753175	-0.171718512	0.975999817	2.424753175	0.015750697		2.424753175
FBgn0266408	ncRNA	43.53231782	-0.740191216	0.400863957	43.53231782	-0.812322665	0.308092284	43.53231782
FBgn0266409	CG45049 gene product from transcrip	994.2569088	0.051810716	0.995584438	994.2569088	0.201754519	0.899670936	994.2569088
FBgn0266410	CG45050 gene product from transcrip	9867.258346	0.12458176	0.994151247	9867.258346	0.184109688	0.933048047	9867.258346
FBgn0266411	similar	3277.434988	0.126670104	0.978331272	3277.434988	0.093363201	0.950220403	3277.434988
FBgn0266412	ncRNA	1.917860104	-0.02621932	0.998322595	1.917860104	-0.159568821		1.917860104
FBgn0266413	ncRNA	1.979532958	0.003067423	0.999472035	1.979532958	0.153267697		1.979532958
FBgn0266414	ncRNA	32.36153486	0.418762983	0.871091663	32.36153486	0.42949735	0.781979744	32.36153486
FBgn0266415	ncRNA	0.198065639	0		0.198065639	0.166537523		0.198065639
FBgn0266416	ncRNA	0.221414942	0.066564265		0.221414942	0		0.221414942
FBgn0266417	CG45057 gene product from transcrip	1773.897126	-0.263044767	0.690730055	1773.897126	-0.50362503	0.115617793	1773.897126
FBgn0266418	wide awake	1186.755113	-0.184957887	0.961031911	1186.755113	-0.22250771	0.848588436	1186.755113
FBgn0266419	ncRNA	0			0			0
FBgn0266420	Otefin	457.1052681	-0.0137821	0.998322595	457.1052681	0.01562683	0.995108225	457.1052681
FBgn0266421	Replication protein A3	793.1744745	-0.187436069	0.949774689	793.1744745	-0.070819344	0.96950421	793.1744745
FBgn0266428	ncRNA	0			0			0
FBgn0266429	Allatostatin A receptor 1	42.92105255	-0.090126463	0.994497358	42.92105255	-0.098558201	0.973524774	42.92105255
FBgn0266430	CG45060 gene product from transcrip	29.27157286	-0.367861243	0.901320232	29.27157286	-0.504043207	0.692875202	29.27157286
FBgn0266431	CG45061 gene product from transcrip	9.011922243	-0.386049619	0.911043162	9.011922243	-0.566965753	0.666389573	9.011922243
FBgn0266432	CG45062 gene product from transcrip	0.419452047	0.109213798		0.419452047	0.095667579		0.419452047
FBgn0266433	CG45063 gene product from transcrip	0.293028587	0.182888499		0.293028587	0		0.293028587
FBgn0266434	CG45064 gene product from transcrip	0			0			0
FBgn0266435	CG45065 gene product from transcrip	0			0			0
FBgn0266436	CG45066 gene product from transcrip	0.981850844	0.216161631		0.981850844	-0.021537739		0.981850844
FBgn0266437	CG45067 gene product from transcrip	2.296060517	-0.263550494	0.933260463	2.296060517	-0.11594367		2.296060517
FBgn0266438	CG45068 gene product from transcrip	0			0			0
FBgn0266439	CG45069 gene product from transcrip	0			0			0
FBgn0266441	CG45071 gene product from transcrip	463.4228043	0.040563993	0.995120986	463.4228043	-0.171665625	0.877559024	463.4228043
FBgn0266442	CG45072 gene product from transcrip	0.374074571	-0.064488993		0.374074571	-0.109585345		0.374074571
FBgn0266443	CG45073 gene product from transcrip	0.219233118	0.166466733		0.219233118	0		0.219233118
FBgn0266445	CG45075 gene product from transcrip	0			0			0
FBgn0266446	CG45076 gene product from transcrip	14543.52428	0.046730294	0.994497358	14543.52428	0.265253271	0.666389573	14543.52428
FBgn0266448	CG45078 gene product from transcrip	1946.96664	0.883948464	0.086659627	1946.96664	0.91790006	0.06940836	1946.96664
FBgn0266449	Kruppel homolog 2	2627.729482	-0.028720884	0.995584438	2627.729482	0.261409822	0.62956189	2627.729482
FBgn0266450	Kruppel homolog 1	313.9160848	0.290499497	0.901320232	313.9160848	-0.033489545	0.991444605	313.9160848
FBgn0266451	CG45077 gene product from transcrip	11.42584774	-0.016550396	0.998322595	11.42584774	-0.010305325	0.999078753	11.42584774
FBgn0266452	CTP synthase	1035.996934	-0.120806604	0.954654825	1035.996934	0.00161179	1	1035.996934
FBgn0266454	CG45079 gene product from transcrip	0.972979616	-0.049018598		0.972979616	0.019220827		0.972979616
FBgn0266455	CG45080 gene product from transcrip	0			0			0
FBgn0266456	CG45081 gene product from transcrip	39.40876768	-0.765113583	0.416464882	39.40876768	-0.820372146	0.338183264	39.40876768
FBgn0266457	ncRNA	3.697189532	0.11409277	0.994497358	3.697189532	-0.379466117		3.697189532
FBgn0266459	CG45084 gene product from transcrip	0			0			0
FBgn0266464	N-ethylmaleimide-sensitive factor 2	1795.426212	-0.193439738	0.872786274	1795.426212	-0.011971319	0.996470448	1795.426212
FBgn0266465	Germinal centre kinase III	1329.371272	-0.08553301	0.969368572	1329.371272	-0.133034949	0.805951663	1329.371272
FBgn0266486	CG45085 gene product from transcrip	0			0			0
FBgn0266487	CG45086 gene product from transcrip	0			0			0
FBgn0266488	CG45087 gene product from transcrip	0			0			0
FBgn0266489	CG45088 gene product from transcrip	0			0			0
FBgn0266490	CG45089 gene product from transcrip	0			0			0
FBgn0266491	Sarcolamban A	0			0			0
FBgn0266492	Sarcolamban B	66.832066	0.308065327	0.920213485	66.832066	0.473842302	0.66111643	66.832066
FBgn0266518	DNA polymerase interacting tpr contair	1407.324732	-0.175781435	0.961442281	1407.324732	-0.23124843	0.825788686	1407.324732
FBgn0266521	stathmin	4082.708576	-0.512180289	0.383821342	4082.708576	-0.471647337	0.447521962	4082.708576
FBgn0266525	CG45092 gene product from transcrip	0			0			0
FBgn0266526	CG45093 gene product from transcrip	0			0			0
FBgn0266534	CG45095 gene product from transcrip	40.26678339	0.083938304	0.9945211	40.26678339	0.189528242	0.934529508	40.26678339
FBgn0266535	ncRNA	0			0			0
FBgn0266536	ncRNA	0			0			0
FBgn0266537	ncRNA	0			0			0
FBgn0266538	CG45099 gene product from transcrip	0			0			0
FBgn0266539	CG45100 gene product from transcrip	0			0			0
FBgn0266540	CG45101 gene product from transcrip	0			0			0
FBgn0266549	ncRNA	465.9340993	0.364714218	0.892718858	465.9340993	0.814687048	0.29594872	465.9340993
FBgn0266557	kismet	6373.530992	0.166874524	0.978641661	6373.530992	0.10904013	0.959037694	6373.530992
FBgn0266564	ncRNA	0.664187759	0.169546281		0.664187759	0		0.664187759
FBgn0266565	ncRNA	0.543244748	0.054998633		0.543244748	0.117053603		0.543244748
FBgn0266566	CG45105 gene product from transcrip	62.44105014	0.110797948	0.993127879	62.44105014	-0.293476781	0.816599359	62.44105014
FBgn0266568	yellow-achaete intergenic RNA	4.019748464	-0.053352863	0.996957673	4.019748464	-0.015835054		4.019748464
FBgn0266569	CG42259 gene product from transcrip	1210.73898	0.215756384	0.971565969	1210.73898	0.314887886	0.831996423	1210.73898
FBgn0266570	CG2982 gene product from transcript (	952.3659552	-0.139150977	0.97064214	952.3659552	-0.042703326	0.984488479	952.3659552
FBgn0266572	ellipsoid body open	1082.04902	-0.247873292	0.755584388	1082.04902	-0.067170634	0.95955656	1082.04902
FBgn0266573	transfer RNA:CR45106	0			0			0
FBgn0266574	transfer RNA:CR45107	0.739977485	-0.210140558		0.739977485	-0.207706246		0.739977485
FBgn0266577	ncRNA	6.074631546	-0.591055664	0.703955165	6.074631546	-0.579129858		6.074631546
FBgn0266578	ncRNA	0			0			0
FBgn0266579	CG45110 gene product from transcrip	425.7581351	0.164591795	0.953941411	425.7581351	0.562998062	0.156114036	425.7581351
FBgn0266580	Gp210 ortholog (H. sapiens)	1590.823798	-0.051171993	0.994497358	1590.823798	0.305021636	0.645558524	1590.823798

FBgn0266581	pitchoune	1617.562988	0.126549745	0.961435662	1617.562988	0.110009205	0.91558304	1617.562988	-0.204886771	0.598618523
FBgn0266582	NADH dehydrogenase (ubiquinone) Fe	5733.924422	0.065019256	0.994876798	5733.924422	0.210799085	0.902565728	5733.924422	-0.724615885	0.156902005
FBgn0266585	transfer RNA:CR32456	0.07379547	0.108851729		0.07379547	0		0.07379547	-0.111773044	
FBgn0266586	ncRNA	3.665791644	0.120680472	0.994497358	3.665791644	-0.180759404		3.665791644	-0.470350436	0.55742689
FBgn0266587	ncRNA	2.024891398	-0.032541341	0.998322595	2.024891398	-0.003957884		2.024891398	0.174654233	0.851042447
FBgn0266588	ncRNA	0			0			0		
FBgn0266589	ncRNA	0			0			0		
FBgn0266590	ncRNA	0.354957647	-0.058919079		0.354957647	-0.098661968		0.354957647	0.009889388	
FBgn0266591	CG45116 gene product from transcript	56.61681621	1.264110485	0.023158471	56.61681621	1.024594364	0.115617793	56.61681621	-1.92888521	2.67E-05
FBgn0266592	ncRNA	0.275212753	0.054051185		0.275212753	0		0.275212753	0.074308479	
FBgn0266593	ncRNA	1.076666991	-0.026665161		1.076666991	-0.009503066		1.076666991	0.253868444	
FBgn0266594	ncRNA	0.922216133	0.006299121		0.922216133	-0.080463945		0.922216133	0.028898051	
FBgn0266595	ncRNA	0.246623758	0.110226517		0.246623758	0.072763218		0.246623758	-0.111232335	
FBgn0266596	ncRNA	1.484707494	-0.014760409		1.484707494	0.058739577		1.484707494	0.229857449	0.768313548
FBgn0266598	kinetochore Mis12-Ndc80 network cor	48.17266525	0.153871286	0.988216049	48.17266525	-0.164115062	0.941279144	48.17266525	0.091647773	0.933349818
FBgn0266599	Heat shock protein cognate 4	80906.94444	0.252001613	0.47538892	80906.94444	0.096191778	0.89404045	80906.94444	0.104920801	0.766026327
FBgn0266615	CG45122 gene product from transcript	1.323518566	0.258763087		1.323518566	0		1.323518566	0.227987849	0.736101497
FBgn0266616	ncRNA	0.677652237	0.049001208		0.677652237	0		0.677652237	0.219475977	
FBgn0266617	ncRNA	1.240721597	0.015229349		1.240721597	-0.183706359		1.240721597	0.071905924	
FBgn0266618	ncRNA	10.2992549	0.04130837	0.998322595	10.2992549	0.19413062	0.941360428	10.2992549	0.096162638	0.943201255
FBgn0266619	ncRNA	25.23601771	-0.323834191	0.946165413	25.23601771	-0.266841713	0.90480014	25.23601771	0.427578567	0.633645925
FBgn0266620	ncRNA	2.578265057	0.127986976	0.989856236	2.578265057	-0.063312478		2.578265057	-0.025226	0.983168695
FBgn0266621	ncRNA	6.24354614	-0.264661316	0.969368572	6.24354614	-0.404073567	0.808155744	6.24354614	-0.533786927	0.534122995
FBgn0266622	ncRNA	4.110024292	0.223097945	0.975532686	4.110024292	-0.113120516		4.110024292	-0.180066811	0.870502422
FBgn0266623	ncRNA	0.07379547	0.108851729		0.07379547	0		0.07379547	-0.111773044	
FBgn0266624	ncRNA	13.21656977	-0.959603551	0.269030267	13.21656977	-0.597355377	0.645025488	13.21656977	0.444339087	0.638787682
FBgn0266625	ncRNA	14.62745222	0.248553934	0.974745767	14.62745222	-0.354765102	0.851257465	14.62745222	-0.242376559	0.830781882
FBgn0266626	ncRNA	2.128423969	0.060457017	0.995120986	2.128423969	0.106065798		2.128423969	-0.083858277	0.936208179
FBgn0266627	ncRNA	6.885456486	-0.237559876	0.974745767	6.885456486	0.000863822	1	6.885456486	0.148940192	0.906210568
FBgn0266628	ncRNA	2.449186225	0.142629371	0.990713915	2.449186225	-0.183107401		2.449186225	0.019497903	0.989633106
FBgn0266629	ncRNA	4.77162354	0.018439662	0.998322595	4.77162354	-0.463922081		4.77162354	-0.102091412	0.930086818
FBgn0266630	ncRNA	8.940265899	0.172110244	0.988572405	8.940265899	0.136380672	0.960705837	8.940265899	0.051697452	0.971803745
FBgn0266631	ncRNA	27.88015923	0.530897086	0.766559852	27.88015923	0.885285199	0.324338644	27.88015923	-0.247661367	0.826551735
FBgn0266632	ncRNA	6.280406264	0.029060639	0.998322595	6.280406264	0.102517045	0.950220403	6.280406264	-0.064023345	0.937036523
FBgn0266633	ncRNA	12.76875963	-0.643281885	0.64547863	12.76875963	-0.709125154	0.522753428	12.76875963	0.228507782	0.843090043
FBgn0266634	ncRNA	1.253889708	0.271451646		1.253889708	0.129580436		1.253889708	-0.121880835	
FBgn0266635	ncRNA	0.568637886	0		0.568637886	0.193087183		0.568637886	0.194012577	
FBgn0266636	ncRNA	38.5107532	0.261050861	0.962850925	38.5107532	0.359263918	0.816599359	38.5107532	-0.472456172	0.546215671
FBgn0266637	ncRNA	6.071110101	-0.30608267	0.954654825	6.071110101	-0.346915045		6.071110101	-0.082318778	0.953118278
FBgn0266638	ncRNA	0.976998529	0.085652805		0.976998529	-0.12340249		0.976998529	-0.138296434	
FBgn0266639	ncRNA	23.86463433	-0.032345741	0.998322595	23.86463433	-0.226046262	0.920916018	23.86463433	0.236614191	0.82792366
FBgn0266640	ncRNA	0.599393078	0.015212148		0.599393078	-0.010770686		0.599393078	-0.031084698	
FBgn0266641	ncRNA	0.099032819	0		0.099032819	0.123176119		0.099032819	0	
FBgn0266642	ncRNA	1.630629465	0.137073559	0.985645036	1.630629465	-0.131413903		1.630629465	-0.033641163	0.9757426

[illegible]

FBgn0267022	ncRNA	1.824531637	0.278545793	0.917248088	1.824531637	-0.063629144		1.824531637	-0.317461291	0.656615749	1.824531637	-0.038923938		1.824531637	0.018495062
FBgn0267023	ncRNA	6.591855624	-0.076908443	0.994876798	6.591855624	-0.215124305	0.920826993	6.591855624	0.341987047	0.714739064	6.591855624	0.265086016	0.937707552	6.591855624	0.154274955
FBgn0267024	ncRNA	0.778464952	-0.2437248		0.778464952	-0.237154743		0.778464952	0.113505253		0.778464952	-0.130215729		0.778464952	0.156588297
FBgn0267025	ncRNA	3.091293734	0.078794424	0.9945211	3.091293734	-0.108933909		3.091293734	-0.219575491	0.823519204	3.091293734	-0.140786729		3.091293734	0.138882398
FBgn0267026	pseudo	0.702716867	-0.026767222		0.702716867	0.038896085		0.702716867	0.149953062		0.702716867	0.123190606		0.702716867	-0.014883731
FBgn0267027	ncRNA	0.266709503	0		0.266709503	0.176133145		0.266709503	0		0.266709503	0		0.266709503	-0.17606399
FBgn0267028	ncRNA	4.63532614	0.204998144	0.979231897	4.63532614	0.078782236		4.63532614	0.324354171	0.755220395	4.63532614	0.529358275		4.63532614	-0.028892712
FBgn0267029	ncRNA	105.3864413	-0.217320456	0.945699553	105.3864413	-0.027557162	0.992131234	105.3864413	0.552288382	0.222706596	105.3864413	0.334969947	0.757814553	105.3864413	-0.039644695
FBgn0267030	ncRNA	5.645425514	0.183441751	0.988216049	5.645425514	-0.118497193		5.645425514	-0.140779684	0.915895163	5.645425514	0.042659361	1	5.645425514	0.082554235
FBgn0267031	ncRNA	0.412147151	0.07097141		0.412147151	0.128811869		0.412147151	-0.071880625		0.412147151	0		0.412147151	-0.12871756
FBgn0267032	ncRNA	0.056320698	0		0.056320698	0		0.056320698	0		0.056320698	0		0.056320698	0.091014032
FBgn0267033	maternal gene required for meiosis	1658.873343	0.598153505	0.530023602	1658.873343	0.795252293	0.251608855	1658.873343	-0.81043337	0.146099897	1658.873343	-0.21228581	0.944620965	1658.873343	-0.674994118
FBgn0267034	ncRNA	0			0			0			0			0	
FBgn0267035	ncRNA	7.361615745	0.212792687	0.979231897	7.361615745	0.262221609	0.910666561	7.361615745	-0.362588676	0.714852105	7.361615745	-0.149803495	0.974390852	7.361615745	-0.445087992
FBgn0267036	ncRNA	49.523821	-0.017953742	0.998322595	49.523821	-0.074494161	0.985591316	49.523821	0.527091881	0.538504967	49.523821	0.509144279	0.774777902	49.523821	0.073274027
FBgn0267037	ncRNA	2.808323977	0.258710339	0.945911599	2.808323977	0.380427374		2.808323977	0.133127272	0.8934216	2.808323977	0.391840997		2.808323977	-0.344542298
FBgn0267038	ncRNA	0.278838563	-0.173996697		0.278838563	-0.17168468		0.278838563	0		0.278838563	-0.176158346		0.278838563	0
FBgn0267039	ncRNA	0			0			0			0			0	
FBgn0267040	ncRNA	1.024377476	0.175480903		1.024377476	0		1.024377476	0.001399781		1.024377476	0.176880726		1.024377476	0.245443634
FBgn0267041	ncRNA	29.3392923	0.166647272	0.987458415	29.3392923	-0.158349827	0.94728821	29.3392923	-0.423678084	0.596371881	29.3392923	-0.257034323	0.933211758	29.3392923	-0.200979945
FBgn0267042	CG45486 gene product from transcrip	2.567424033	-0.075958734	0.994497358	2.567424033	0.264890588		2.567424033	-0.191426488	0.822631341	2.567424033	-0.267391446		2.567424033	-0.432669203
FBgn0267043	CG45487 gene product from transcrip	0			0			0			0			0	
FBgn0267044	CG45488 gene product from transcrip	0			0			0			0			0	
FBgn0267045	CG45489 gene product from transcrip	0.221386409	0.173816599		0.221386409	0		0.221386409	-0.174835842		0.221386409	0		0.221386409	0
FBgn0267046	CG45490 gene product from transcrip	0.099032819	0		0.099032819	0.123176119		0.099032819	0		0.099032819	0		0.099032819	-0.122843617
FBgn0267047	CG45491 gene product from transcrip	0			0			0			0			0	
FBgn0267048	CG45492 gene product from transcrip	0			0			0			0			0	
FBgn0267049	CG45493 gene product from transcrip	0			0			0			0			0	
FBgn0267050	CG45494 gene product from transcrip	0.246623758	0.110226517		0.246623758	0.072763218		0.246623758	-0.111232335		0.246623758	0		0.246623758	-0.072685092
FBgn0267051	pseudo	0.19573998	-0.103274018		0.19573998	-0.100786204		0.19573998	0		0.19573998	-0.10557425		0.19573998	0.053925479
FBgn0267052	pseudo	0.180453536	-0.163802206		0.180453536	-0.161348312		0.180453536	0		0.180453536	-0.166076918		0.180453536	0
FBgn0267053	pseudo	0			0			0			0			0	
FBgn0267054	pseudo	0			0			0			0			0	
FBgn0267055	pseudo	0			0			0			0			0	
FBgn0267056	ncRNA	0.056320698	0		0.056320698	0		0.056320698	0		0.056320698	0		0.056320698	0.091014032
FBgn0267057	ncRNA	7.858355508	-0.250041355	0.969626359	7.858355508	-0.169595135	0.946634373	7.858355508	-0.15057377	0.903291524	7.858355508	-0.400619182	0.863150128	7.858355508	0.273378852
FBgn0267058	ncRNA	1.855928102	0.271938927	0.921905921	1.855928102	0.016489843		1.855928102	-0.327444837	0.635162715	1.855928102	-0.055515091		1.855928102	0.078276077
FBgn0267059	ncRNA	0.280220173	-0.0438418		0.280220173	-0.041660055		0.280220173	0.049952169		0.280220173	0.006112043		0.280220173	0.082678078
FBgn0267060	ncRNA	0			0			0			0			0	
FBgn0267061	ncRNA	0			0			0			0			0	
FBgn0267062	ncRNA	1.571561797	0.178789202		1.571561797	0.040326937		1.571561797	0.058174735	0.942325187	1.571561797	0.236965537		1.571561797	-0.073107993
FBgn0267063	ncRNA	0			0			0			0			0	
FBgn0267064	ncRNA	0.056320698	0		0.056320698	0		0.056320698	0		0.056320698	0		0.056320698	0.091014032
FBgn0267065	ncRNA	0			0			0			0			0	
FBgn0267066	ncRNA	0.056320698	0		0.056320698	0		0.056320698	0		0.056320698	0		0.056320698	0.091014032
FBgn0267067	ncRNA	0.952839049	0		0.952839049	0.366606522		0.952839049	0.052550965		0.952839049	0.05691198		0.952839049	-0.138568031
FBgn0267068	ncRNA	0			0			0			0			0	
FBgn0267069	ncRNA	0			0			0			0			0	
FBgn0267070	ncRNA	1.044376501	0.199439418		1.044376501	0.039317374		1.044376501	-0.261174244		1.044376501	-0.061743258		1.044376501	0.271310353
FBgn0267071	ncRNA	0			0			0			0			0	
FBgn0267072	ncRNA	0.100708642	0		0.100708642	0		0.100708642	0.114352451		0.100708642	0.116098517		0.100708642	0
FBgn0267073	ncRNA	53.49239949	0.110798698	0.994497358	53.49239949	0.580973304	0.579373297	53.49239949	-0.295585563	0.751701679	53.49239949	-0.184789502	0.957236635	53.49239949	-0.662405408
FBgn0267074	ncRNA	0			0			0			0			0	
FBgn0267075	ncRNA	0.133946648	0.011640396		0.133946648	-0.065113325		0.133946648	-0.082465381		0.133946648	-0.070827708		0.133946648	0
FBgn0267076	ncRNA	1.005431084	0.05716701		1.005431084	0.043482276		1.005431084	0.043778933		1.005431084	0.100947254		1.005431084	-0.042858337
FBgn0267077	ncRNA	0.056320698	0		0.056320698	0		0.056320698	0		0.056320698	0		0.056320698	0.091014032
FBgn0267078	ncRNA	0.930744963	-0.037141048		0.930744963	0.016896192		0.930744963	-0.057735491		0.930744963	-0.094878491		0.930744963	-0.045428487
FBgn0267079	ncRNA	0.147590939	0.153120924		0.147590939	0		0.147590939	-0.15413487		0.147590939	0		0.147590939	0
FBgn0267080	ncRNA	0.229148987	0.059032177		0.229148987	0.074836564		0.229148987	-0.060046196		0.229148987	0		0.229148987	-0.027679651
FBgn0267081	ncRNA	6.1492319	-0.425913842	0.872095585	6.1492319	-0.364148233	0.836520481	6.1492319	0.39185716	0.686181535	6.1492319	-0.03404845	1	6.1492319	0.119472791
FBgn0267082	ncRNA	0			0			0			0			0	
FBgn0267083	ncRNA	0.702722524	0.045816076		0.702722524	0.021323158		0.702722524	-0.029782645		0.702722524	0.016032467		0.702722524	0.076685951
FBgn0267084	ncRNA	1.758765444	-0.156290524	0.978661819	1.758765444	0.170242544		1.758765444	-0.101149325	0.912851833	1.758765444	-0.257443204		1.758765444	-0.446148387
FBgn0267085	ncRNA	0			0			0			0			0	
FBgn0267086	ncRNA	11.27883313	0.143080811	0.994497358	11.27883313	0.309775277	0.881045512	11.27883313	0.422121299	0.665000897	11.27883313	0.565208173	0.730439214	11.27883313	-0.405600777
FBgn0267087	ncRNA	15.27603373	0.544375957	0.754340177	15.27603373	-0.032002202	0.995305272	15.27603373	0.012798198	0.993231313	15.27603373	0.557174269	0.731467978	15.27603373	0.322363044
FBgn0267088	ncRNA	0			0			0			0			0	
FBgn0267089	ncRNA	0.728188121	-0.019003373		0.728188121	-0.023833708		0.728188121	-0.04168706		0.728188121	-0.060691763		0.728188121	-0.016978437
FBgn0267090	ncRNA	0.07379547	0.108851729		0.07379547	0		0.07379547	-0.111773044		0.07379547	0		0.07379547	0
FBgn0267091	ncRNA	0.765302094	0.259596687		0.765302094	0.137949631		0.765302094	-0.208797297		0.765302094	0.050792784		0.765302094	-0.093935498
FBgn0267096	ncRNA	0			0			0			0			0	
FBgn0267097	ncRNA	1.706902463	-0.01564371	0.998322595	1.706902463	0.12803857		1.706902463	0.141607557	0.792631765	1.706902463	0.125968441		1.706902463	-0.086167896
FBgn0267098	ncRNA	0			0			0			0			0	
FBgn0267099	ncRNA	0.241472153	0.06444487		0.241472153	0.110367284		0.241472153	-0.065471921		0.241472153	0		0.241472153	-0.110302494
FBgn0267100	ncRNA	1.232177418	-0.02680527		1.232177418	0.241435147		1.232177418	-0.070711286		1.232177418	-0.097519045		1.232177418	0.005642461
FBgn0267101	ncRNA	0.273501217	-0.041754955		0.273501217	-0.039628719		0.273501217	0.061592761		0.273501217	0.019839859		0.273501217	0.07622326
FBgn0267102	ncRNA	3.597036883	0.197180702	0.975999817	3.597036883	0.349332366		3.597036883	-0.045477753	0.972091932	3.597036883	0.151701777		3.597036883	-0.24673374
FBgn0267103	ncRNA	1.479108889	0.4099092		1.479108889	0.274120723		1.479108889	-0.348057475	0.516558554	1.479108889	0.061841161		1.479108889	-0.19076955
FBgn0267104	CG45544 gene product from transcrip	1.083026319	0.088784259		1.083026319	0.169854657		1.083026319	-0.021217819		1.083026319	-0.06756578		1.083026319	-0.102070724
FBgn0267105	CG45545 gene product from transcrip	0.139419282	-0.122978845		0.139419282	-0.119403538		0.139419282	0		0.139419282	-0.127144101		0.139419282	0
FBgn0267106	CG45546 gene product from transcrip	0.314400184	-0.098911827		0.314400184	-0.142618533		0.314400184	-0.047618647		0.314400184	-0.146532115		0.314400184	0
FBgn0267107	ncRNA	0.854300441	0.050819588		0.854300441	0.225326651		0.854300441	0.124344613		0.854300441	0.175168236		0.854300441	-0.186182435
FBgn0267108	pseudo	0.12877022	0		0.12877022	0		0.12877022	0.078096705		0.12877022	0.079487931		0.12877022	0.062960077
FBgn0267109	ncRNA	3.530418687	-0.453769483	0.806327386	3.530418687	-0.771304824		3.530418687	-0.274205138	0.776855535	3.530418687	-0.727982019		3.530418687	0.217490298
FBgn0267110	ncRNA	0			0			0			0			0	
FBgn0267111	ncRNA	80.23595635	0.371216432	0.872095585	80.23595635	-0.060091135	0.986599042	80.23595635	0.160911951	0.877727005	80.23595635	0.532129463	0.651750248	80.23595635	-0.193295336
FBgn0267112	ncRNA	4.263252506	-0.074596109	0.994876798	4.263252506	0.132159334		4.263252506	0.149882504	0.895743089	4.263252506	0.075290292		4.263252506	-0.11233447
FBgn0267113	ncRNA	9.458979863	0.097759106	0.994497358	9.458979863	0.238598732	0.918961524	9.458979863	0.24475368	0.829684959	9.458979863	0.342517243	0.911771836	9.458979863	-0.197520974
FBgn0267114	ncRNA	0.297098458	0		0.297098458	0.186453258		0.297098458	0		0.297098458	0		0.297098458	-0.186369208
FBgn0267115	ncRNA	0.287038754	-0.086452148		0.287038754	-0.084191522		0.287038754	0		0.287038754	-0.088571072		0.287038754	0.087428992
FBgn0267116	ncRNA	0.144899044	0		0.144899044	0		0.144899044	0.148757697		0.144899044	0.150113718		0.144899044	0
FBgn0267117	ncRNA	1.398501166	-0.010413177		1.398501166	0.172152077		1.398501166	-0.007380039	0.993367502	1.398501166	-0.017793449		1.398501166	-0.270703094
FBgn0267118	ncRNA	0.245607685	0		0.245607685	0		0.245607685	0.174171351		0.245607685	0.175306785		0.245607685	0
FBgn0267119	ncRNA	70.58729281	-0.420091961	0.703955165	70.58729281	-0.240836026	0.860381325	70.58729281	0.512192914	0.342507605	70.58729281	0.092103422	0.981145999	70.58729281	0.245142785
FBgn0267120	ncRNA	0.112641396	0		0.112641396	0		0.112641396	0		0.112641396	0		0.112641396	0.135141552
FBgn0267121	ncRNA	0			0			0			0			0	
FBgn0267122	ncRNA	0.590363756	0.212547667		0.590363756	0		0.590363756	-0.213293314		0.590363756	0		0.590363756	0
FBgn0267123	ncRNA	0.368977348	0.196066888		0.368977348	0		0.368977348	-0.196979264		0.368977348	0		0.368977348	0
FBgn0267124	ncRNA	4.110858618	0.351104666	0.917248088	4.110858618	0.046898711		4.110858618	0.138245177	0.913461381	4.110858618	0.489352391		4.110858618	-0.099704675
FBgn0267125	ncRNA	1.431491238	0.066530701		1.431491238	-0.076435414		1.431491238	0.003745798	0.997322959	1.431491238	0.070276566		1.431491238	-0.135416256
FBgn0267126	ncRNA	14.45747391	-0.146039695	0.993966728	14.45747391	-0.183345157	0.944894567	14.45747391	0.43495539	0.639934973	14.45747391	0.288921396	0.932912729	14.45747391	0.436071174
FBgn0267127	ncRNA	3.489317718	-0.098419413	0.994497358	3.489317718	0.068488434		3.489317718	0.191177617	0.843359506	3.489317718	0.092763092		3.489317718	-0.296026364
FBgn0267128	ncRNA	0.966895504	0.228577967		0.966895504	0.250541163		0.966895504	-0.23178589		0.966895504	0		0.966895504	-0.124297387
FBgn0267129	ncRNA	0.171482341	0		0.171482341	0.091530081		0.171482341	0.069298968		0.171482341	0.070592924		0.171482341	-0.091486164
FBgn0267130	ncRNA	41.84621126	0.933458461	0.269030267	41.84621126	0.173641313	0.948432738	41.84621126	2.359804534	2.89E-07	41.84621126	3.293283975	1.03E-13	41.84621126	2.832102701
FBgn0267131	ncRNA	0.060151179	-0.097832662		0.060151179	-0.093256207		0.060151179	0		0.060151179	-0.102528014		0.060151179	0
FBgn0267132	ncRNA	0.12877022	0		0.12877022	0		0.12877022	0.078096705		0.12877022	0.079487931		0.12877022	0.062960077
FBgn0267133	ncRNA	0.072449522	0		0.072449522	0		0.072449522	0.101824093		0.072449522	0.103598231		0.072449522	0
FBgn0267134	ncRNA	0			0			0			0			0	
FBgn0267135	ncRNA	0.07379547	0.108851729		0.07379547	0		0.07379547	-0.111773044		0.07379547	0		0.07379547	0
FBgn0267136	ncRNA	0.281566121	0.008801825		0.281566121	-0.041534417		0.281566121	-0.054562021		0.281566121	-0.045762023		0.281566121	0.082485599
FBgn0267137	ncRNA	0.647952565	0.075721271		0.647952565	-0.063251927		0.647952565	-0.240304185		0.647952565	-0.164950536		0.647952565	-0.039297521
FBgn0267138	ncRNA	0.586480261	0.01318483		0.586480261	-0.031276396		0.586480261	-0.16214583		0.586480261	-0.148966282		0.586480261	-0.115065232
FBgn0267139	ncRNA	0.293028587	0.182888499		0.293028587	0		0.293028587	-0.18387959		0.293028587	0		0.293028587	0
FBgn0267140	ncRNA	0.060151179	-0.097832662		0.060151179	-0.093256207		0.060151179	0		0.060151179	-0.102528014		0.060151179	0
FBgn0267141	ncRNA	1.59556727	0.057615532		1.59556727	0.226714734		1.59556727	-0.069892019	0.917793618	1.59556727	-0.012278846		1.59556727	-0.210759058
FBgn0267142	ncRNA	5.990782282	0.059216151	0.995584438	5.990782282	0.094403961		5.990782282	0.313445585	0.755590939	5.990782282	0.372668212	0.883230548	5.990782282	-0.16546226
FBgn0267143	ncRNA	0.099032819	0		0.099032819	0.123176119		0.099032819	0		0.099032819	0		0.099032819	-0.122843617
FBgn0267144	ncRNA	1.418082665	-0.150345113		1.418082665	-0.209920054		1.418082665	-0.007479749	0.993336681	1.418082665	-0.157825096		1.418082665	0.091856849
FBgn0267145	ncRNA	1.784314157	0.085885364	0.994497358	1.784314157	-0.015256183		1.784314157	0.057840214	0.954690984	1.784314157	0.143727231		1.784314157	0.28252482
FBgn0267146	ncRNA	0.201758346	0.107303578		0.201758346	0		0.201758346	-0.108333141		0.201758346	0		0.201758346	0.053294381
FBgn0267147	ncRNA	0.213214751	-0.032560156		0.213214751	-0.096443753		0.213214751	-0.068579174		0.213214751	-0.10114162		0.213214751	0

FBgn0267484	3.479744912	0.097500616	0.994497358	3.479744912	0.266522599		3.479744912	-0.401315584	0.611010045
FBgn0267485	1.783342393	-0.328768295	0.845308204	1.783342393	0.200041592		1.783342393	-0.080901478	0.931414712
FBgn0267486	579.5712579	-0.120294748	0.979231897	579.5712579	-0.427383456	0.479376545	579.5712579	0.115076069	0.866524525
FBgn0267487	1815.078413	0.024531073	0.996542676	1815.078413	-0.13477981	0.870965316	1815.078413	0.230089671	0.516488754
FBgn0267488	1953.486531	-0.135624793	0.982804959	1953.486531	0.095233801	0.959953166	1953.486531	0.400660801	0.461040122
FBgn0267489	0.07379547	0.108851729		0.07379547	0		0.07379547	-0.111773044	
FBgn0267490	0			0			0		
FBgn0267491	0			0			0		
FBgn0267492	0.221386409	0.173816599		0.221386409	0		0.221386409	-0.174835842	
FBgn0267493	0			0			0		
FBgn0267494	0			0			0		
FBgn0267495	220.2593674	0.206789751	0.975532686	220.2593674	0.619101524	0.490270746	220.2593674	-0.15278552	0.883739236
FBgn0267513	160.3202573	0.514700025	0.787351793	160.3202573	0.352676639	0.851257465	160.3202573	-0.678918877	0.389473558
FBgn0267514	168.6034696	0.157192306	0.992706654	168.6034696	1.348355566	0.042911314	168.6034696	-0.537802427	0.541181887
FBgn0267515	142393.3866	0.151065812	0.989856236	142393.3866	1.06764277	0.081817951	142393.3866	-0.339093927	0.693177463
FBgn0267516	1104.221671	0.557574715	0.516034745	1104.221671	0.436091141	0.656953918	1104.221671	-0.523533842	0.379976361
FBgn0267517	13.74791519	0.006210905	0.998322595	13.74791519	-0.192768589	0.908103495	13.74791519	-0.074469776	0.937711632
FBgn0267518	0.099032819	0		0.099032819	0.123176119		0.099032819	0	
FBgn0267519	479207.5888	0.371535748	0.886446899	479207.5888	0.393756658	0.785649499	479207.5888	-0.61414026	0.382507375
FBgn0267520	70436.0858	0.039755243	0.998322595	70436.0858	0.323253558	0.869020803	70436.0858	-0.170795006	0.891643921
FBgn0267521	0			0			0		
FBgn0267522	0			0			0		
FBgn0267523	364.390765	0.583626441	0.716029477	364.390765	0.233835904	0.920812352	364.390765	-0.99445427	0.134812462
FBgn0267524	5.698684772	-0.329710092	0.946165413	5.698684772	-0.32062491		5.698684772	-0.346779548	0.728620611
FBgn0267525	0			0			0		
FBgn0267526	0.39589052	0.14917845		0.39589052	0		0.39589052	-0.099287381	
FBgn0267527	0.280191639	0.055197709		0.280191639	-0.038840333		0.280191639	-0.049409595	
FBgn0267528	1.777110384	0.143327186	0.985645036	1.777110384	0.123206243		1.777110384	-0.060742867	0.953972895
FBgn0267529	0			0			0		
FBgn0267530	0.708716499	0.040854985		0.708716499	0.030408672		0.708716499	-0.094898925	
FBgn0267531	0			0			0		
FBgn0267532	0			0			0		
FBgn0267533	1.183952016	-0.06785133		1.183952016	-0.22357971		1.183952016	0.047640364	
FBgn0267534	18.57837821	-0.265107827	0.968528723	18.57837821	-0.211118178	0.933048047	18.57837821	0.233286139	0.835459744
FBgn0267535	0			0			0		
FBgn0267536	0.648390177	0.194700908		0.648390177	-0.032980892		0.648390177	-0.234643346	
FBgn0267537	0			0			0		
FBgn0267538	0			0			0		
FBgn0267539	0.871901343	0.188817372		0.871901343	-0.015832909		0.871901343	-0.206879059	
FBgn0267540	0			0			0		
FBgn0267541	0.582307	0.046472537		0.582307	0.162064634		0.582307	0.051243919	
FBgn0267542	0			0			0		
FBgn0267543	0			0			0		
FBgn0267544	0.060151179	-0.097832662		0.060151179	-0.093256207		0.060151179	0	

[illegible]

FBgn0267933	0			0			0		
FBgn0267934	0.822815084	-0.050107229		0.822815084	-0.149123209		0.822815084	0.114857621	
FBgn0267935	0.147590939	0.153120924		0.147590939	0		0.147590939	-0.15413487	
FBgn0267936	0.279384296	0.090973343		0.279384296	-0.042526045		0.279384296	-0.137770454	
FBgn0267937	0			0			0		
FBgn0267938	0.099032819	0		0.099032819	0.123176119		0.099032819	0	
FBgn0267939	0.201417284	0		0.201417284	0		0.201417284	0.161275553	
FBgn0267940	0.139419282	-0.122978845		0.139419282	-0.119403538		0.139419282	0	
FBgn0267941	0.471734902	0		0.471734902	0.086278813		0.471734902	0.094412236	
FBgn0267944	0			0			0		
FBgn0267945	0			0			0		
FBgn0267946	0			0			0		
FBgn0267947	0.468002987	0.053118364		0.468002987	-0.035437596		0.468002987	-0.094364731	
FBgn0267948	0.186436866	0.066048487		0.186436866	0		0.186436866	-0.06707759	
FBgn0267949	0			0			0		
FBgn0267950	0.243931863	0		0.243931863	0.073126577		0.243931863	0.106271572	
FBgn0267951	0.144899044	0		0.144899044	0		0.144899044	0.148757697	
FBgn0267952	0.345656578	0.086868499		0.345656578	0.110389119		0.345656578	-0.087797162	
FBgn0267953	0.07379547	0.108851729		0.07379547	0		0.07379547	-0.111773044	
FBgn0267954	15.26878784	0.595766185	0.694459829	15.26878784	0.739393162	0.480784815	15.26878784	-0.863709728	0.22287223
FBgn0267955	0			0			0		
FBgn0267956	0.147619473	0		0.147619473	0		0.147619473	0	
FBgn0267957	0.07379547	0.108851729		0.07379547	0		0.07379547	-0.111773044	
FBgn0267958	0			0			0		
FBgn0267959	0.393668287	-0.116476885		0.393668287	-0.15496952		0.393668287	-0.042114613	
FBgn0267960	0.056320698	0		0.056320698	0		0.056320698	0	
FBgn0267961	0.100708642	0		0.100708642	0		0.100708642	0.114352451	
FBgn0267962	0			0			0		
FBgn0267963	0			0			0		
FBgn0267964	0			0			0		
FBgn0267965	0.446193541	-0.07941586		0.446193541	-0.139316021		0.446193541	-0.063264189	
FBgn0267966	0.537828929	0.223302757		0.537828929	0		0.537828929	-0.102287998	
FBgn0267967	20.62254628	0.598877889	0.694459829	20.62254628	-0.016358593	0.997538821	20.62254628	-0.017454385	0.991009778
FBgn0267969	0.201219742	0		0.201219742	0		0.201219742	0.117621883	
FBgn0267970	4.331041766	-0.074879199	0.994876798	4.331041766	0.472145037		4.331041766	0.007868447	0.995035614
FBgn0267972	2187.295433	0.063354274	0.99157593	2187.295433	0.379155613	0.258830207	2187.295433	-0.124702448	0.771119459
FBgn0267975	3367.353556	-0.017663049	0.998322595	3367.353556	0.053997105	0.984041268	3367.353556	-0.227689585	0.735297813
FBgn0267976	2486.787164	0.015895282	0.998322595	2486.787164	0.133016008	0.939574224	2486.787164	-0.050408286	0.958924384
FBgn0267977	92.85909035	-0.111284377	0.99157593	92.85909035	-0.079155363	0.972335724	92.85909035	0.296424513	0.649750877
FBgn0267978	1012.241526	-0.166464784	0.957372756	1012.241526	0.086504713	0.952755155	1012.241526	0.399004991	0.341568997
FBgn0267982	3.777360521	0.04251545	0.997208689	3.777360521	-0.190782013		3.777360521	-0.381521727	0.584409141
FBgn0267983	4.196792974	-0.038298329	0.998322595	4.196792974	-0.161587256		4.196792974	-0.005190076	0.997071933
FBgn0267984	0.666462747	0		0.666462747	0.101209201		0.666462747	0.084742743	
FBgn0267985	1.030502431	0.010752785		1.030502431	0.140075562		1.030502431	-0.024551001	
FBgn0267986	1.861855052	0.258442766	0.858079153	1.861855052	0.399013761		1.861855052	-0.261227077	0.630552224

[illegible]

FBgn0035444	CG12012 gene product from transcrip	911.4130077	-0.288328168	0.536722837	911.4130077	-0.353820792	[illegible]
FBgn0035445	CG12014 gene product from transcrip	51.21275357	-0.223199851	0.971565969	51.21275357	-0.663160489	[illegible]
FBgn0027616	CG12076 gene product from transcrip	3570.583186	-0.005126112	0.998322595	3570.583186	-0.172794096	[illegible]
FBgn0052268	Drosomycin-like 6	0			0		[illegible]
FBgn0052274	Drosomycin-like 1	1.891662383	-0.539193433	0.413211179	1.891662383	-0.472925973	[illegible]
FBgn0035440	CG14969 gene product from transcrip	940.1674018	-0.14934692	0.917248088	940.1674018	-0.065951687	[illegible]
FBgn0035437	CG11526 gene product from transcrip	1698.253713	0.000518677	0.999781966	1698.253713	-0.191314275	[illegible]
FBgn0035435	Phosphatidylinositol glycan anchor bic	62.20893617	0.126770428	0.988572405	62.20893617	0.217504043	[illegible]
FBgn0035431	CG14968 gene product from transcrip	518.2731832	-0.995919876	0.005950645	518.2731832	-0.844141903	[illegible]
FBgn0035425	CG17746 gene product from transcrip	1607.240635	-0.184439211	0.909660755	1607.240635	-0.344331967	[illegible]
FBgn0035422	Ribosomal protein L28	16738.7227	0.127720692	0.975999817	16738.7227	0.058635893	[illegible]
FBgn0035420	CG14967 gene product from transcrip	777.9283536	0.146660187	0.97064214	777.9283536	0.150975847	[illegible]
FBgn0053160	CG33160 gene product from transcrip	0.747279545	-0.087588811		0.747279545	-0.175314885	[illegible]
FBgn0052277	CG32277 gene product from transcrip	1.616250237	0.055774716	0.994876798	1.616250237	-0.158427649	[illegible]
FBgn0053159	CG33159 gene product from transcrip	0.657281127	-0.028743722		0.657281127	0.018226186	[illegible]
FBgn0052271	CG32271 gene product from transcrip	3.642349559	0.114343049	0.994497358	3.642349559	-0.604316071	[illegible]
FBgn0047135	CG32276 gene product from transcrip	14068.84143	0.16002846	0.91488891	14068.84143	0.557199919	[illegible]
FBgn0035415	CG14966 gene product from transcrip	508.2633983	0.028231399	0.995584438	508.2633983	0.158707256	[illegible]
FBgn0035414	CG14965 gene product from transcrip	129.3008329	-0.08118501	0.994497358	129.3008329	0.113635286	[illegible]
FBgn0035410	CG14964 gene product from transcrip	690.5764032	0.475747789	0.688566959	690.5764032	0.80702901	[illegible]
FBgn0035409	CG14963 gene product from transcrip	240.5108679	-0.435168877	0.733331754	240.5108679	-1.463774815	[illegible]
FBgn0035407	ASCIZ homolog	92.99061646	-0.176951084	0.97064214	92.99061646	-0.288943714	[illegible]
FBgn0035402	CG12082 gene product from transcrip	3229.98828	-0.105208346	0.988354972	3229.98828	-0.138607182	[illegible]
FBgn0035400	CG11537 gene product from transcrip	2361.085025	-0.1415577	0.844755814	2361.085025	0.037040474	[illegible]
FBgn0026189	CG7740 gene product from transcript	2908.991211	-0.076330836	0.994497358	2908.991211	-0.096499765	[illegible]
FBgn0052273	transfer RNA:CR32273	0			0		[illegible]
FBgn0035398	CG1869 gene product from transcript	2685.515936	0.075864562	0.994497358	2685.515936	0.376725881	[illegible]
FBgn0052272	transfer RNA:CR32272	0			0		[illegible]
FBgn0005640	Ecdysone-induced protein 63E	1619.696749	-0.04814826	0.995120986	1619.696749	-0.059754881	[illegible]
FBgn0035452	CG10359 gene product from transcrip	1688.5791	0.185356097	0.846035137	1688.5791	0.251463755	[illegible]
FBgn0035453	CG10357 gene product from transcrip	0.796470107	-0.133353901		0.796470107	-0.026131271	[illegible]
FBgn0035455	CG10862 gene product from transcrip	3.359973298	0.93949313	0.040100402	3.359973298	-0.019058196	[illegible]
FBgn0040694	CG14974 gene product from transcrip	1.435059468	0.327654685		1.435059468	0.040244889	[illegible]
FBgn0001254	Ecdysone-inducible gene E2	7252.983891	-0.343390168	0.912603335	7252.983891	-0.569501119	[illegible]
FBgn0052267	CG32267 gene product from transcrip	461.775035	-0.150053994	0.975999817	461.775035	0.465405341	[illegible]
FBgn0035449	CG14971 gene product from transcrip	1720.68564	-0.204013603	0.929926159	1720.68564	-0.274008702	[illegible]
FBgn0026259	CG10840 gene product from transcrip	6126.072023	0.122829776	0.975999817	6126.072023	0.163907191	[illegible]
FBgn0014388	sprouty	1611.379481	0.274106317	0.856137457	1611.379481	0.353292035	[illegible]
FBgn0052266	CG32266 gene product from transcrip	26.68335246	0.828775853	0.412272658	26.68335246	1.145596518	[illegible]
FBgn0035462	Integrator 10	182.8903827	0.049521208	0.994876798	182.8903827	0.108697031	[illegible]
FBgn0035464	CG12006 gene product from transcrip	892.1977053	0.072581447	0.989856236	892.1977053	0.203485564	[illegible]
FBgn0004888	Succinyl coenzyme A synthetase alpha	9591.832108	0.076751129	0.994497358	9591.832108	0.172416053	[illegible]
FBgn0004875	encore	1979.362608	0.235356217	0.832566727	1979.362608	0.295840517	[illegible]
FBgn0064227	CG14975 gene product from transcrip	5.953823019	0.336158621	0.944197659	5.953823019	0.568086975	[illegible]
FBgn0013751	Arrowhead	100.848857	-0.303250634	0.844755814	100.848857	0.024437644	[illegible]
FBgn0026592	Fire exit	42.99101944	0.420066048	0.799062687	42.99101944	-0.026173571	[illegible]
FBgn0035469	CG14977 gene product from transcrip	922.1130621	0.006555563	0.998322595	922.1130621	0.032563049	[illegible]
FBgn0035471	CG10849 gene product from transcrip	1412.714227	0.148254563	0.839263768	1412.714227	0.296414324	[illegible]
FBgn0041147	imaginal discs arrested	172.5228738	-0.081744619	0.994497358	172.5228738	-0.06332994	[illegible]
FBgn0004910	Ecdysone-induced protein 63F 1	2087.313245	0.033629709	0.99723561	2087.313245	-0.200750217	[illegible]
FBgn0035473	maggie	1513.881464	0.127850592	0.973453438	1513.881464	0.312596808	[illegible]
FBgn0035470	Ccz1 ortholog	431.6435666	-0.078975335	0.988216049	431.6435666	-0.224499403	[illegible]
FBgn0035468	Gustatory receptor 63a	1.044367789	-0.092524381		1.044367789	0.051268628	[illegible]
FBgn0015585	Accessory gland protein 63F	9.329927735	0.835635245	0.40244538	9.329927735	0.005516893	[illegible]
FBgn0052263	CG32263 gene product from transcrip	0.440619526	0.2012941		0.440619526	0	[illegible]
FBgn0052262	CG32262 gene product from transcrip	83.92257956	0.216488584	0.953941411	83.92257956	-0.028669034	[illegible]
FBgn0035461	nutcracker	114.5316797	0.255969025	0.8856311	114.5316797	-0.077824225	[illegible]
FBgn0052264	CG32264 gene product from transcrip	2754.521355	0.018493707	0.998322595	2754.521355	0.116329714	[illegible]
FBgn0035458	pickpocket 27	1.164545699	-0.11342488		1.164545699	0.035536697	[illegible]
FBgn0035490	CG1136 gene product from transcript	259.7136511	0.346812153	0.816948048	259.7136511	0.442894619	[illegible]
FBgn0042179	CG18869 gene product from transcrip	148.0587976	-0.42872489	0.738212573	148.0587976	-0.163312472	[illegible]
FBgn0000449	disembodied	47.90916305	0.220173329	0.972479043	47.90916305	0.146601463	[illegible]
FBgn0052259	CG32259 gene product from transcrip	5.690852972	0.754075407	0.346206788	5.690852972	0.057788273	[illegible]
FBgn0035495	CG14989 gene product from transcrip	1977.709323	-1.062343302	7.88E-05	1977.709323	-1.207733138	[illegible]
FBgn0035496	CG14990 gene product from transcrip	1965.307453	0.461944485	0.310743837	1965.307453	0.145620267	[illegible]
FBgn0035498	Fermitin 1	2685.459636	-0.10676356	0.975861786	2685.459636	-0.002429521	[illegible]
FBgn0028484	Activated Cdc42 kinase	1268.528284	-0.281141373	0.759515508	1268.528284	-0.247765273	[illegible]
FBgn0003206	Ras oncogene at 64B	3483.229763	-0.053105938	0.99365579	3483.229763	0.014014907	[illegible]
FBgn0004552	Adipokinetic hormone	67.45711374	0.500517058	0.68788912	67.45711374	-0.259053787	[illegible]
FBgn0052260	CG32260 gene product from transcrip	14.76603243	0.356969839	0.924143028	14.76603243	-0.340144472	[illegible]
FBgn0035501	CG1299 gene product from transcript	2305.265968	0.004347511	0.998322595	2305.265968	-0.166742707	[illegible]
FBgn0040696	CG18675 gene product from transcrip	0.814329135	0.18657078		0.814329135	0	[illegible]
FBgn0003710	temperature-induced paralytic E	64.26091837	0.085558113	0.994497358	64.26091837	0.003803173	[illegible]
FBgn0035513	Cuticular protein 64Ad	13.47733121	-0.055965472	0.997117822	13.47733121	0.233964615	[illegible]
FBgn0035515	CG14997 gene product from transcrip	1483.877889	-0.141500727	0.950893746	1483.877889	-0.031026666	[illegible]
FBgn0011692	pavarotti	976.4599621	-0.132089656	0.956414448	976.4599621	-0.048399465	[illegible]
FBgn0035517	CG1265 gene product from transcript	1968.676142	-0.281850768	0.89316218	1968.676142	-0.413495593	[illegible]
FBgn0035519	CG1309 gene product from transcript	710.4013234	-0.126531087	0.949623474	710.4013234	0.027913228	[illegible]
FBgn0035521	Vacuolar H[+] ATPase M9.7 subunit a	67.58967457	0.098153852	0.994497358	67.58967457	0.168664714	[illegible]
FBgn0035522	CG1273 gene product from transcript	193.1209588	-0.022992475	0.998322595	193.1209588	-0.301066874	[illegible]
FBgn0035523	CG1311 gene product from transcript	1217.118148	-0.065888629	0.994497358	1217.118148	0.023977573	[illegible]
FBgn0035526	CG1316 gene product from transcript	2176.887383	0.083868066	0.988354972	2176.887383	-0.03886923	[illegible]
FBgn0015829	Transcription factor IIEbeta	338.6711482	0.249073497	0.755584388	338.6711482	0.081095075	[illegible]
FBgn0041630	Hexosaminidase 1	4560.591703	-0.128055341	0.979091207	4560.591703	-0.402207575	[illegible]
FBgn0035529	CG1319 gene product from transcript	1003.421438	0.087341356	0.989375441	1003.421438	0.16863213	[illegible]
FBgn0035528	CG15012 gene product from transcrip	3282.640528	-0.171900806	0.946165413	3282.640528	-0.453660627	[illegible]
FBgn0035524	CG11583 gene product from transcrip	558.4879822	-0.100830275	0.981438301	558.4879822	0.028957268	[illegible]
FBgn0035520	CG11586 gene product from transcrip	572.5508428	-0.080485875	0.985411701	572.5508428	-0.062131622	[illegible]
FBgn0035518	CG15011 gene product from transcrip	612.8855101	0.159872194	0.936128798	612.8855101	0.397285642	[illegible]
FBgn0041171	archipelago	3246.64533	-0.039761965	0.9945211	3246.64533	-0.089211882	[illegible]
FBgn0001257	Ecdysone-inducible gene L2	3574.215008	-0.395915469	0.563822344	3574.215008	-0.037061965	[illegible]
FBgn0052252	ncRNA	2.841207885	-0.008453496	0.998322595	2.841207885	-0.14123929	[illegible]
FBgn0035512	Cuticular protein 64Ac	2.901054778	0.094336715	0.994497358	2.901054778	-0.290805871	[illegible]
FBgn0035511	Cuticular protein 64Ab	1.157109284	-0.001948193		1.157109284	-0.106068161	[illegible]
FBgn0035510	Cuticular protein 64Aa	104.3402726	0.012738212	0.998322595	104.3402726	0.106033049	[illegible]
FBgn0035508	CG15005 gene product from transcrip	21.06041701	0.507784548	0.792520678	21.06041701	0.192845263	[illegible]
FBgn0028664	Vacuolar H[+] ATPase M9.7 subunit c	462.4556343	0.028436408	0.995584438	462.4556343	0.064147418	[illegible]
FBgn0040697	tipE homolog 3	11.37335268	0.074176815	0.995120986	11.37335268	0.124794359	[illegible]
FBgn0035505	tipE homolog 2	85.23406347	-0.143072625	0.988354972	85.23406347	-0.194718787	[illegible]
FBgn0035504	tipE homolog 4	42.71792035	0.228929077	0.968528723	42.71792035	-0.005344872	[illegible]
FBgn0011653	masquerade	327.5120986	-0.149528193	0.974005319	327.5120986	-0.071310939	[illegible]
FBgn0004574	Ras opposite	3578.107163	-0.172665187	0.963243134	3578.107163	-0.161411543	[illegible]
FBgn0035499	CG14996 gene product from transcrip	12682.95077	-0.451404545	0.18228871	12682.95077	-0.426436993	[illegible]
FBgn0004516	Glutamic acid decarboxylase 1	233.7855396	-0.06295647	0.994497358	233.7855396	-0.108110101	[illegible]
FBgn0016013	Fumarylacetoacetase	181.1462758	0.484794556	0.688182473	181.1462758	0.928308165	[illegible]
FBgn0024179	wishful thinking	715.336296	-0.239042203	0.74052988	715.336296	-0.19367954	[illegible]
FBgn0035491	Dpy-30-like 2	3.271179729	0.470374706	0.500897942	3.271179729	0.017926707	[illegible]
FBgn0035488	CG11593 gene product from transcrip	690.6176299	-0.304008987	0.728029857	690.6176299	-0.194978092	[illegible]
FBgn0052251	CG32251 gene product from transcrip	475.8832328	-0.086246678	0.994497358	475.8832328	0.091714221	[illegible]
FBgn0052250	CG32250 gene product from transcrip	932.8641168	-0.423613922	0.423255665	932.8641168	-0.217699182	[illegible]
FBgn0035534	mitochondrial ribosomal protein S6	276.5917658	-0.38553379	0.803460791	276.5917658	0.083080183	[illegible]
FBgn0035539	slowdown	646.8656661	-0.630419789	0.495571928	646.8656661	-1.001880984	[illegible]
FBgn0035540	Syntaxin 17	826.9690499	-0.230526151	0.753828842	826.9690499	-0.47770757	[illegible]
FBgn0035542	CG11347 gene product from transcrip	17418.79715	0.22991232	0.929926159	17418.79715	0.368392807	[illegible]
FBgn0035543	CG15020 gene product from transcrip	0.352641161	0.047012041		0.352641161	-0.032328	[illegible]
FBgn0000038	nicotinic Acetylcholine Receptor beta1	112.4468557	0.206528518	0.944197659	112.4468557	0.098960026	[illegible]
FBgn0035544	CG15021 gene product from transcrip	386.7049326	0.500323428	0.719948026	386.7049326	0.390326772	[illegible]
FBgn0035545	CG12607 gene product from transcrip	954.0519399	1.254816872	0.068908222	954.0519399	1.179412675	[illegible]
FBgn0052249	CG32249 gene product from transcrip	41297.8699	0.315944117	0.85659012	41297.8699	0.305976142	[illegible]
FBgn0052248	CG32248 gene product from transcrip	155.9458363	1.833788318	0.000927066	155.9458363	2.298171362	[illegible]
FBgn0035550	CG11349 gene product from transcrip	2526.803853	-0.068072574	0.992711351	2526.803853	0.319613009	[illegible]
FBgn0035551	CG7465 gene product from transcript	3308.583638	0.503147882	0.688847553	3308.583638	1.041480456	[illegible]
FBgn0035552	CG11350 gene product from transcrip	16244.21451	0.682941656	0.518399313	16244.21451	0.918976138	[illegible]
FBgn0035557	CG11353 gene product from transcrip	39.00876364	0.88097804	0.304148555	39.00876364	0.491168689	[illegible]
FBgn0035558	CG11357 gene product from transcrip	1627.528706	0.012518763	0.998322595	1627.528706	-0.084582502	[illegible]
FBgn0052246	CG32246 gene product from transcrip	2.457124031	0.233110962	0.960742862	2.457124031	0.045852388	[illegible]
FBgn0015805	CG7471 gene product from transcript	3253.321356	0.181673564	0.805580095	3253.321356	0.045194754	[illegible]
FBgn0035563	CG13716 gene product from transcrip	4.947823413	-0.169601406	0.988216049	4.947823413	-0.06551075	[illegible]
FBgn0052243	CG32243 gene product from transcrip	751.3017669	0.168218497	0.944197659	751.3017669	0.191650632	[illegible]
FBgn0014073	Tie-like receptor tyrosine kinase	308.5787905	0.328818425	0.753828842	308.5787905	0.329129089	[illegible]
FBgn0035555	CG13720 gene product from transcrip	0.662835455	-0.189104959		0.662835455	-0.185737953	[illegible]
FBgn0035554	CG13721 gene product from transcrip	22.26388303	-0.084171195	0.994876798	22.26388303	0.174997429	[illegible]
FBgn0035553	CG13722 gene product from transcrip	1810.543776	0.107563541	0.994497358	1810.543776	-0.215318162	[illegible]
FBgn0052241	CG32241 gene product from transcrip	2928.430227	0.735392303	0.521677008	2928.430227	0.757979426	[illegible]
FBgn0040699	CG15024 gene product from transcrip	0.281566121	0.008801825		0.281566121	-0.041534417	[illegible]
FBgn0035548	CG15023 gene product from transcrip	0.710187003	-0.053258957		0.710187003	0.067453147	[illegible]
FBgn0035547	CG15022 gene product from transcrip	0			0		[illegible]
FBgn0035546	CG11345 gene product from transcrip	377.3543978	0.910570032	0.1429836	377.3543978	1.058100423	[illegible]
FBgn0035541	CG15019 gene product from transcrip	1709.876475	0.000150033	0.999994709	1709.876475	-0.190974203	[illegible]
FBgn0052240	CG32240 gene product from transcrip	2.974271275	0.29163639	0.892718858	2.974271275	-0.224819233	[illegible]
FBgn0035538	Dopamine/Ecdysteroid receptor	167.4896401	-0.661540032	0.173111853	167.4896401	-0.689903174	[illegible]
FBgn0035537	CG11342 gene product from transcrip	58.87568894	-0.247794678	0.968528723	58.87568894	0.098372872	[illegible]
FBgn0035533	Cdc42-interacting protein 4	3290.816707	-0.114461867	0.959208615	3290.816707	-0.249294404	[illegible]
FBgn0035532	CG15014 gene product from transcrip	465.1459834	0.042144214	0.995120986	465.1459834	-0.034792441	[illegible]
FBgn0066365	dusky-like	17.73591122	0.00086041	0.999781966	17.73591122	-0.076065748	[illegible]
FBgn0052238	CG32238 gene product from transcrip	3.009357298	0.360006434	0.72893315	3.009357298	-0.083526377	[illegible]
FBgn0035574	Rho guanine nucleotide exchange facto	1715.582172	0.181773972	0.956414448	1715.582172	0.058903782	[illegible]
FBgn0042199	CG13713 gene product from transcrip	1.829559356	0.052969416	0.994876798	1.829559356	-0.019526476	[illegible]
FBgn0035572	CG13711 gene product from transcrip	0.493709725	0.063803795		0.493709725	0	[illegible]
FBgn0027085	Leucyl-tRNA synthetase	476.3291129	-0.042647492	0.994497358	476.3291129	0.117989154	[illegible]
FBgn0035581	CG17150 gene product from transcrip	5.331194466	0.167997627	0.989856236	5.331194466	0.035060196	[illegible]
FBgn0035582	CG13705 gene product from transcrip	2572.004234	0.469124728	0.760039945	2572.004234	0.721969684	[illegible]
FBgn0035583	CG13704 gene product from transcrip	941.8655513	-0.573229262	0.17364741	941.8655513	-0.740682849	[illegible]
FBgn0028699	CG7499 gene product from transcript	466.2464695	0.461652991	0.727537036	466.2464695	1.004344439	[illegible]
FBgn0047330	CG32235 gene product from transcrip	0.139419282	-0.122978845		0.139419282	-0.119403538	[illegible]
FBgn0052237	CG32237 gene product from transcrip	11.02024105	-0.08634295	0.994876798	11.02024105	-0.11102446	[illegible]
FBgn0035578	CG13707 gene product from transcrip	263.3417568	-0.465011982	0.536514704	263.3417568	-0.638656817	[illegible]
FBgn0035577	CG13708 gene product from transcrip	15.24195118	0.057001527	0.997003712	15.24195118	-0.236938362	[illegible]
FBgn0042131	CG18808 gene product from transcrip	2.578188331	0.305838047	0.925140457	2.578188331	0.046404339	[illegible]
FBgn0035575	CG7509 gene product from transcript	5.807451241	0.281509571	0.963043532	5.807451241	0.162005484	[illegible]
FBgn0035571	CG12493 gene product from transcrip	12.82207722	0.880957411	0.339396406	12.82207722	-0.010567379	[illegible]
FBgn0035570	CG13712 gene product from transcrip	1.083146094	0.039985711		1.083146094	0.204842576	[illegible]
FBgn0035569	CG15876 gene product from transcrip	3.239466918	0.543886549	0.59839604	3.239466918	0.077088065	[illegible]
FBgn0035568	CG18418 gene product from transcrip	1.54970486	0.237156884		1.54970486	0	[illegible]
FBgn0035567	CG7514 gene product from transcript	0.07379547	0.108851729		0.07379547	0	[illegible]
FBgn0046793	CG32236 gene product from transcrip	1.707401451	0.295595046	0.738212573	1.707401451	0.017352635	[illegible]
FBgn0035585	CG12027 gene product from transcrip	2.329078858	0.36376336	0.569019066	2.329078858	-0.032856582	[illegible]
FBgn0052232	CG32232 gene product from transcrip	0.147590939	0.153120924		0.147590939	0	[illegible]
FBgn0035584	CG17030 gene product from transcrip	0.516568287	0.208268862		0.516568287	0	[illegible]
FBgn0005775	Connectin	303.299899	0.174571319	0.975999817	303.299899	0.112930379	[illegible]
FBgn0030234	CG15211 gene product from transcrip	1280.211223	-0.097501726	0.994497358	1280.211223	0.333867084	[illegible]
FBgn0040850	CG15210 gene product from transcrip	977.4033192	-0.421698842	0.639712927	977.4033192	-0.807896125	[illegible]
FBgn0030237	CG15209 gene product from transcrip	1818.962986	-0.430110689	0.717990485	1818.962986	-0.901927029	[illegible]
FBgn0030239	CG17333 gene product from transcrip	1423.712637	0.040907906	0.995120986	1423.712637	0.343761427	[illegible]
FBgn0030240	CG2202 gene product from transcript	882.2977481	-0.065659841	0.993466746	882.2977481	0.094671528	[illegible]
FBgn0030243	CG2186 gene product from transcript	1898.053117	0.115814593	0.949774689	1898.053117	0.101311484	[illegible]
FBgn0030244	CG2157 gene product from transcript	430.9482747	0.395986774	0.717821646	430.9482747	0.327822994	[illegible]
FBgn0030247	CG15208 gene product from transcrip	3.597169203	0.771460405	0.188311913	3.597169203	-0.077073981	[illegible]
FBgn0003965	vermilion	816.8569161	0.194470988	0.956414448	816.8569161	0.09588489	[illegible]
FBgn0030251	CG2145 gene product from transcript	6112.311349	0.214613101	0.946165413	6112.311349	0.263231777	[illegible]
FBgn0030257	CG11160 gene product from transcrip	376.028453	0.293599045	0.89770139	376.028453	0.333290143	[illegible]
FBgn0030261	CG15203 gene product from transcrip	1185.969949	0.042930671	0.995584438	1185.969949	0.132074026	[illegible]
FBgn0030260	CG1537 gene product from transcript	96.89002846	0.174481061	0.971565969	96.89002846	-0.14774465	[illegible]
FBgn0030259	CG1545 gene product from transcript	28.56148435	-0.028338068	0.998322595	28.56148435	-0.322728398	[illegible]
FBgn0030258	CG1552 gene product from transcript	74.14458243	0.16218812	0.987458415	74.14458243	0.035161299	[illegible]
FBgn0021742	CG1567 gene product from transcript	7.697508257	0.421860991	0.88370699	7.697508257	0.681038202	[illegible]
FBgn0052670	Rab at 9Fb	0.952138266	0.157678329		0.952138266	0.010890496	[illegible]
FBgn0052671	Rab at 9Fa	0.147590939	0.153120924		0.147590939	0	[illegible]
FBgn0030246	CG1582 gene product from transcript	1164.492867	0.001805838	0.999550539	1164.492867	0.027404225	[illegible]
FBgn0017561	Open rectifier K[+] channel 1	214.0439566	-0.057344369	0.994876798	214.0439566	-0.495308965	[illegible]
FBgn0030245	CG1637 gene product from transcript	3540.504767	-0.245044549	0.72740743	3540.504767	-0.352126898	[illegible]
FBgn0030242	sister of feo	204.5125858	0.138237104	0.975999817	204.5125858	-0.103569658	[illegible]
FBgn0030241	fascetto	1023.963165	-0.047748125	0.994876798	1023.963165	-0.157801268	[illegible]
FBgn0003321	small bristles	1301.735385	-0.082356531	0.985645036	1301.735385	-0.102058774	[illegible]
FBgn0052669	CG32669 gene product from transcrip	407.824278	0.299877106	0.815846652	407.824278	0.111994416	[illegible]
FBgn0003360	stress-sensitive B	43485.45843	0.202046913	0.946165413	43485.45843	0.507713814	[illegible]
FBgn0025111	Adenine nucleotide translocase 2	63.37311311	0.510667428	0.610840834	63.37311311	-0.031728224	[illegible]
FBgn0030263	CG2076 gene product from transcript	0			0		[illegible]
FBgn0027498	CG2061 gene product from transcript	0			0		[illegible]
FBgn0015245	Heat shock protein 60	11837.80584	0.294611257	0.832566727	11837.80584	0.51855511	[illegible]
FBgn0028686	Regulatory particle triple-A ATPase 3	7623.140321	-0.168174275	0.969626359	7623.140321	-0.434992095	[illegible]
FBgn0030269	CG18292 gene product from transcrip	734.2024178	-0.104970136	0.989856236	734.2024178	-0.098483554	[illegible]
FBgn0030271	CG15202 gene product from transcrip	31.74687384	0.051944164	0.997117822	31.74687384	0.029857121	[illegible]
FBgn0030272	CG15201 gene product from transcrip	202.0794355	0.299616012	0.882516171	202.0794355	0.167070134	[illegible]
FBgn0030274	l(3)mbt interacting protein 1	2012.430515	-0.394207942	0.587634051	2012.430515	-0.71986208	[illegible]
FBgn0030276	Dynein light intermediate chain	3634.206965	-0.185595245	0.89770139	3634.206965	0.077445444	[illegible]
FBgn0030278	CG15200 gene product from transcrip	2.048798376	0.415986927	0.341648199	2.048798376	0	[illegible]
FBgn0030280	CG11106 gene product from transcrip	4.114930064	0.548312321	0.245942131	4.114930064	0	[illegible]
FBgn0030283	CG15198 gene product from transcrip	1.475909391	0.233889205		1.475909391	0	[illegible]
FBgn0030286	CG1657 gene product from transcript	1721.742746	-0.157291353	0.910739253	1721.742746	-0.198549436	[illegible]
FBgn0030290	CG11756 gene product from transcrip	7.279831269	-0.324286029	0.952645803	7.279831269	-0.694092299	[illegible]
FBgn0030291	CG1738 gene product from transcript	197.4043373	-0.056432462	0.994497358	197.4043373	-0.024341274	[illegible]
FBgn0030292	CG11752 gene product from transcrip	858.959692	-0.033700208	0.998322595	858.959692	0.111481338	[illegible]
FBgn0030293	CG1737 gene product from transcript	1938.268607	0.120131639	0.954378726	1938.268607	0.119326791	[illegible]
FBgn0000499	dishevelled	1190.71009	0.091787196	0.981763935	1190.71009	-0.104428022	[illegible]
FBgn0030294	PTIP associated 1	1088.168049	-0.310169368	0.798887624	1088.168049	-0.221253127	[illegible]
FBgn0004864	hopscotch	874.7211648	0.035924271	0.995120986	874.7211648	0.11960301	[illegible]
FBgn0030289	CG1597 gene product from transcript	1483.041893	0.066925774	0.994497358	1483.041893	0.042294858	[illegible]
FBgn0028421	Kinesin associated protein 3	1146.129052	-0.051133478	0.9945211	1146.129052	-0.226911599	[illegible]
FBgn0001491	lethal (1) 10Bb	559.1444622	-0.194606888	0.923470826	559.1444622	0.046049309	[illegible]
FBgn0003411	sisterless A	17.96810288	0.792368001	0.442952877	17.96810288	0.165984835	[illegible]
FBgn0030277	CG1394 gene product from transcript	8.403953503	1.015022892	0.040455391	8.403953503	0.026672342	[illegible]
FBgn0026268	CG1386 gene product from transcript	464.2868483	0.053628709	0.996902828	464.2868483	-0.288933328	[illegible]
FBgn0052668	CG32668 gene product from transcrip	11.95747229	0.300203533	0.961031911	11.95747229	0.088520473	[illegible]
FBgn0020255	CG1404 gene product from transcript	13135.91966	0.207319483	0.784469732	13135.91966	0.224599097	[illegible]
FBgn0030270	CG15199 gene product from transcrip	0			0		[illegible]
FBgn0052667	short spindle 7	0.97934183	-0.039565599		0.97934183	-0.088395115	[illegible]
FBgn0030268	Kinesin-like protein at 10A	2546.672386	0.079247268	0.989699975	2546.672386	0.009552482	[illegible]
FBgn0010391	GTP-binding protein	7252.169288	0.11178708	0.988216049	7252.169288	0.261766727	[illegible]
FBgn0030266	CG11122 gene product from transcrip	1142.353525	0.521360072	0.323802335	1142.353525	0.488014358	[illegible]
FBgn0027359	CG1728 gene product from transcript	745.2850766	0.216840842	0.89770139	745.2850766	0.253628937	[illegible]
FBgn0030296	CG15196 gene product from transcrip	1.808871819	0.518141858	0.567039952	1.808871819	-0.124254802	[illegible]
FBgn0001145	Glutamine synthetase 2	17158.34665	-0.111726306	0.988572405	17158.34665	-0.394884686	[illegible]
FBgn0030300	Sphingosine kinase 1	726.7854567	-0.557307749	0.138343653	726.7854567	-0.764900903	[illegible]
FBgn0030301	Heterochromatin protein 5	940.8894642	-0.05944023	0.994497358	940.8894642	-0.120156713	[illegible]
FBgn0030304	CG11715 gene product from transcrip	436.7987028	0.733323155	0.124558231	436.7987028	0.168096689	[illegible]
FBgn0030305	CG1749 gene product from transcript	2796.826197	0.092573316	0.990957786	2796.826197	0.163772729	[illegible]
FBgn0030306	Spase 25-subunit	6100.676081	0.286075829	0.642151225	6100.676081	0.320545643	[illegible]
FBgn0052666	Death-associated protein kinase relatec	2342.194679	0.0725614	0.994497358	2342.194679	0.060398003	[illegible]
FBgn0030310	Peptidoglycan recognition protein SA	541.5610434	0.143699619	0.950976319	541.5610434	0.049012764	[illegible]
FBgn0030311	CG11699 gene product from transcrip	0			0		[illegible]
FBgn0030313	CG11697 gene product from transcrip	2.508373374	0.782890642	0.225604726	2.508373374	0.146714132	[illegible]
FBgn0030314	CG11696 gene product from transcrip	486.550898	0.015637888	0.998322595	486.550898	-0.011911444	[illegible]
FBgn0030316	CG11695 gene product from transcrip	319.9214172	0.01022529	0.998322595	319.9214172	-0.170900227	[illegible]
FBgn0019660	RNA on the X 2	18.78977458	-0.345061612	0.920136002	18.78977458	-1.202923513	[illegible]
FBgn0002948	no distributive disjunction	202.8730939	0.127928942	0.985645036	202.8730939	0.104011558	[illegible]
FBgn0030318	rhomboid-4	1430.337303	-0.135156759	0.967901085	1430.337303	-0.271220456	[illegible]
FBgn0030321	CG1703 gene product from transcript	4456.43518	-0.054289025	0.994497358	4456.43518	0.081112191	[illegible]
FBgn0002723	Methoprene-tolerant	838.8258898	0.437172894	0.516270252	838.8258898	0.35398932	[illegible]
FBgn0004370	Protein tyrosine phosphatase 10D	2226.073513	0.092005997	0.991865251	2226.073513	0.338936064	[illegible]
FBgn0014133	bifocal	4016.239567	-0.175730098	0.938366404	4016.239567	-0.374599472	[illegible]
FBgn0030326	CG2444 gene product from transcript	15198.34232	0.249321551	0.967901085	15198.34232	-0.938583741	[illegible]
FBgn0030323	CG2371 gene product from transcript	287.6171448	-0.104614665	0.989066282	287.6171448	0.047940923	[illegible]
FBgn0030320	CG2247 gene product from transcript	911.3624662	-0.053060206	0.994497358	911.3624662	-0.063004286	[illegible]
FBgn0030319	CG2533 gene product from transcript	8.579099582	0.396588668	0.901320232	8.579099582	-0.085844277	[illegible]
FBgn0030317	CG1561 gene product from transcript	8.323918266	0.007662099	0.998322595	8.323918266	0.226417911	[illegible]
FBgn0000618	enhancer of yellow 2	681.3544152	0.077777144	0.994151247	681.3544152	0.133459651	[illegible]
FBgn0027259	kinetochore Mis12-Ndc80 network coi	143.9276766	0.062718181	0.994497358	143.9276766	-0.132001153	[illegible]
FBgn0003277	RNA polymerase II 215kD subunit	2387.934281	0.166472486	0.952412688	2387.934281	0.255030505	[illegible]
FBgn0030309	CG1572 gene product from transcript	3627.673982	0.103253819	0.9917657	3627.673982	0.360594951	[illegible]
FBgn0030307	CG33235 gene product from transcrip	2.900246759	0.382304639	0.756888805	2.900246759	-0.215354825	[illegible]
FBgn0030298	Odorant receptor 10a	0			0		[illegible]
FBgn0045502	Gustatory receptor 10a	0			0		[illegible]
FBgn0030297	Gustatory receptor 10b	0.060151179	-0.097832662		0.060151179	-0.093256207	[illegible]
FBgn0030329	pretaporter	5733.182158	-0.43040398	0.518399313	5733.182158	-0.572491309	[illegible]
FBgn0030330	Transport and Golgi organization 10	1154.185058	0.058310043	0.99157593	1154.185058	0.023487789	[illegible]
FBgn0030331	CG15221 gene product from transcrip	418.6255195	0.199519226	0.975999817	418.6255195	-0.028920163	[illegible]
FBgn0002577	miniature	67.01256529	-0.187276256	0.985645036	67.01256529	-0.538870386	[illegible]
FBgn0030334	CG4139 gene product from transcript	320.054017	0.470658643	0.518399313	320.054017	0.487498263	[illegible]
FBgn0030339	CG1895 gene product from transcript	19.54746337	-0.311326767	0.953941411	19.54746337	-0.258791945	[illegible]
FBgn0030343	CG1886 gene product from transcript	1375.034309	-0.119655862	0.977442266	1375.034309	0.049342686	[illegible]
FBgn0030345	CG1847 gene product from transcript	342.6081023	-0.263272674	0.850551179	342.6081023	-0.267528218	[illegible]
FBgn0030347	CG15739 gene product from transcrip	191.3711744	-0.812568558	0.146318368	191.3711744	-1.115580257	[illegible]
FBgn0030350	Selenoprotein G	2568.698024	0.002578769	0.999621204	2568.698024	-0.223924293	[illegible]
FBgn0030351	CG1840 gene product from transcript	525.3363121	0.2866929	0.78070356	525.3363121	0.353851331	[illegible]
FBgn0030352	severe impairment of CI with lengthene	467.7547053	-0.162911952	0.966157244	467.7547053	-0.025780363	[illegible]
FBgn0030360	CG1806 gene product from transcript	335.1917356	-0.007198145	0.998322595	335.1917356	-0.0513551	[illegible]
FBgn0030362	CG1803 gene product from transcript	48952.50394	0.027062583	0.998322595	48952.50394	-0.053077844	[illegible]
FBgn0030361	CG1492 gene product from transcript	76.48008829	-0.222079002	0.956325514	76.48008829	-0.335099189	[illegible]
FBgn0001083	furrowed	1509.049375	-0.097851963	0.987458415	1509.049375	-0.002738555	[illegible]
FBgn0030359	CG18130 gene product from transcrip	17.66674034	0.279603107	0.963501097	17.66674034	0.018374864	[illegible]
FBgn0003865	twisted gastrulation	0			0		[illegible]
FBgn0000808	gastrulation-defective	21.51625148	0.265868202	0.966157244	21.51625148	-0.001999335	[illegible]
FBgn0030358	CG10362 gene product from transcrip	71.63637315	-0.260804401	0.937350359	71.63637315	-0.284050455	[illegible]
FBgn0030357	CG2471 gene product from transcript	5351.786755	-0.3822824	0.886446899	5351.786755	-1.053233239	[illegible]
FBgn0052660	ncRNA	0			0		[illegible]
FBgn0030356	ncRNA	1.421331786	0.448289299		1.421331786	0	[illegible]
FBgn0030354	CG1559 gene product from transcript	1793.813128	0.047229872	0.994497358	1793.813128	0.022325838	[illegible]
FBgn0030349	CG10353 gene product from transcrip	2151.912054	0.04238257	0.994497358	2151.912054	0.197359492	[illegible]
FBgn0030348	CG10352 gene product from transcrip	3.805062505	0.045675917	0.997459641	3.805062505	0.433593686	[illegible]
FBgn0030346	CG11802 gene product from transcrip	518.1221128	-0.228820952	0.949623474	518.1221128	-0.301828663	[illegible]
FBgn0030344	CG2025 gene product from transcript	829.9511589	0.127697888	0.975532686	829.9511589	0.235358092	[illegible]
FBgn0030342	CG10347 gene product from transcrip	690.9976717	-0.103245486	0.978641661	690.9976717	0.337485378	[illegible]
FBgn0030341	p24-related-1	2573.160623	0.201751694	0.826123977	2573.160623	0.166943316	[illegible]
FBgn0030340	CG15740 gene product from transcrip	2.473990585	0.469426593	0.718153804	2.473990585	0.279239926	[illegible]
FBgn0030338	CG15741 gene product from transcrip	127.329025	1.2625901	0.057107882	127.329025	0.898729045	[illegible]
FBgn0001218	Heat shock 70-kDa protein cognate 3	45176.52753	0.477584326	0.363267165	45176.52753	0.652168458	[illegible]
FBgn0004511	dusky	22.39940802	-0.21008902	0.979231897	22.39940802	0.137575153	[illegible]
FBgn0030332	CG9360 gene product from transcript	90.8153211	0.055144265	0.995584438	90.8153211	0.526810529	[illegible]
FBgn0030328	CG2446 gene product from transcript	7692.14724	0.165651426	0.93196206	7692.14724	0.016830408	[illegible]
FBgn0030327	alpha1,6-fucosyltransferase	1768.416448	0.084168792	0.994497358	1768.416448	0.072547671	[illegible]
FBgn0030364	CG15735 gene product from transcrip	2327.451682	-0.022248599	0.99564268	2327.451682	-0.086367928	[illegible]
FBgn0030365	Transport and Golgi organization 4	1305.895462	-0.146227916	0.984438423	1305.895462	-0.123457301	[illegible]
FBgn0030369	CG1786 gene product from transcript	43.05020223	-0.273875424	0.953305045	43.05020223	0.205819978	[illegible]
FBgn0052657	ncRNA	1.368431122	0.240461555		1.368431122	-0.040199485	[illegible]
FBgn0040859	ncRNA	5.661964509	1.062775107	0.087960346	5.661964509	0.07796935	[illegible]
FBgn0030376	CG2750 gene product from transcript	1.687547892	0.333076351	0.556956355	1.687547892	-0.015447516	[illegible]
FBgn0052655	CG32655 gene product from transcrip	4.659554136	0.740268596	0.308402171	4.659554136	0.312684272	[illegible]
FBgn0030377	CG1924 gene product from transcript	16.41350664	-0.058911573	0.996902828	16.41350664	-0.143764338	[illegible]
FBgn0030375	CG11356 gene product from transcrip	0.167676683	0		0.167676683	0.129953448	[illegible]
FBgn0030374	CG15734 gene product from transcrip	0.516568287	0.208268862		0.516568287	0	[illegible]
FBgn0030373	CG12721 gene product from transcrip	5.439195755	0.163594212	0.990065596	5.439195755	0.213462723	[illegible]
FBgn0030370	Uch-L5-related	0.571335674	0.059497124		0.571335674	-0.021693759	[illegible]
FBgn0030367	CG1488 gene product from transcript	352.7472403	-0.691377193	0.548513921	352.7472403	-0.89461082	[illegible]
FBgn0030366	Ubiquitin-specific protease 7	3700.070881	0.071857003	0.994497358	3700.070881	0.167258982	[illegible]
FBgn0043001	Chromatin accessibility complex 16kD	335.3865392	-0.10664519	0.994497358	335.3865392	-0.060849272	[illegible]
FBgn0030384	CG2577 gene product from transcript	0.174504111	0.073112477		0.174504111	0	[illegible]
FBgn0030386	CG2574 gene product from transcript	1.258247717	0.291975763		1.258247717	0.040755615	[illegible]
FBgn0030389	CG15927 gene product from transcrip	2.831442537	0.088970715	0.994497358	2.831442537	0.027860925	[illegible]
FBgn0030394	Cuticular protein 11A	5701.75627	0.547854151	0.746692563	5701.75627	0.433002606	[illegible]
FBgn0002562	Larval serum protein 1 alpha	462156.0046	0.623826967	0.435744391	462156.0046	0.883141366	[illegible]
FBgn0030396	CG2556 gene product from transcript	2020.858816	-0.517302616	0.281813143	2020.858816	-0.651625345	[illegible]
FBgn0030398	Cuticular protein 11B	705.9318087	0.163879405	0.971565969	705.9318087	0.336104965	[illegible]
FBgn0052654	Sec16 ortholog (S. cerevisiae)	2786.182669	0.393850682	0.683671744	2786.182669	0.569472967	[illegible]
FBgn0030407	CG2543 gene product from transcript	67.03466568	-0.366373182	0.811144636	67.03466568	-0.221238852	[illegible]
FBgn0030408	CG11085 gene product from transcrip	14.77759896	-0.312650691	0.953755212	14.77759896	-0.433379784	[illegible]
FBgn0030406	CG1463 gene product from transcript	470.960779	0.156825347	0.979153974	470.960779	0.213764933	[illegible]
FBgn0030403	CG1824 gene product from transcript	1501.171476	0.007557021	0.998322595	1501.171476	0.049741242	[illegible]
FBgn0030400	CG11138 gene product from transcrip	1470.366277	0.101054719	0.985645036	1470.366277	0.061739534	[illegible]
FBgn0030395	Meckel syndrome, type 1 homologue	0.669381172	0.076534142		0.669381172	0.102411543	[illegible]
FBgn0030391	CG1900 gene product from transcript	435.1547402	0.182428508	0.916467039	435.1547402	0.286630747	[illegible]
FBgn0030390	CG15731 gene product from transcrip	4.416459766	0.394991676	0.886446899	4.416459766	0.141541809	[illegible]
FBgn0052651	CG32651 gene product from transcrip	1.633711727	0.348655013	0.576250825	1.633711727	-0.040281454	[illegible]
FBgn0030385	Ionotropic receptor 11a	0.308479293	-0.041135639		0.308479293	-0.039026594	[illegible]
FBgn0030409	CG15728 gene product from transcrip	2.709092839	0.660499647	0.400863957	2.709092839	-0.149643637	[illegible]
FBgn0030411	CG2540 gene product from transcript	199.5482041	-0.191900592	0.956325514	199.5482041	-0.324915538	[illegible]
FBgn0030412	Tomosyn ortholog	1675.858994	0.181198863	0.872095585	1675.858994	0.167853008	[illegible]
FBgn0015024	Casein kinase Ialpha	9596.51878	-0.007707632	0.998322595	9596.51878	0.110441542	[illegible]
FBgn0030417	CG15725 gene product from transcrip	65.4463626	0.683378111	0.567187746	65.4463626	0.549580885	[illegible]
FBgn0030418	CG4004 gene product from transcript	756.54368	0.024075058	0.998322595	756.54368	-0.082489218	[illegible]
FBgn0030421	CG3812 gene product from transcript	136.475226	-0.503636752	0.68934315	136.475226	-0.253656266	[illegible]
FBgn0030425	CG3775 gene product from transcript	1174.677089	0.209718626	0.966157244	1174.677089	0.157928736	[illegible]
FBgn0030433	mitochondrial ribosomal protein L49	619.139847	0.029480492	0.998322595	619.139847	0.001736155	[illegible]
FBgn0030435	CG4645 gene product from transcript	1169.096261	0.315699136	0.554131028	1169.096261	0.389648554	[illegible]
FBgn0030434	BRMS1 ortholog	828.2351946	-0.055200048	0.995120986	828.2351946	-0.185147602	[illegible]
FBgn0030432	CG4404 gene product from transcript	47.91741116	-0.762236165	0.425298471	47.91741116	-0.316674433	[illegible]
FBgn0030431	CG4407 gene product from transcript	412.5845124	-0.20684322	0.830887413	412.5845124	0.037862185	[illegible]
FBgn0052649	CG32649 gene product from transcrip	1048.114437	-0.027880852	0.997459641	1048.114437	0.214266051	[illegible]
FBgn0052650	CG32650 gene product from transcrip	29.28223319	0.201698281	0.985010871	29.28223319	-0.238531028	[illegible]
FBgn0030429	CG4661 gene product from transcript	2.636217172	0.63266185	0.391747037	2.636217172	-0.17570098	[illegible]
FBgn0052647	CG32647 gene product from transcrip	157.8000602	0.440213365	0.798529748	157.8000602	0.359517492	[illegible]
FBgn0030420	CG12717 gene product from transcrip	237.0566132	-0.011639764	0.998322595	237.0566132	0.084576827	[illegible]
FBgn0020513	CG3989 gene product from transcript	13243.68121	0.153276838	0.956325514	13243.68121	0.028297219	[illegible]
FBgn0030410	Aven ortholog	180.6052047	-0.012734457	0.998322595	180.6052047	-0.099042823	[illegible]
FBgn0052642	CG32642 gene product from transcrip	0			0		[illegible]
FBgn0052644	Mucin related 11Da	0.201758346	0.107303578		0.201758346	0	[illegible]
FBgn0030438	CG15721 gene product from transcrip	0.407823274	0.143000271		0.407823274	0	[illegible]
FBgn0052643	CG32643 gene product from transcrip	0.733648113	0.243777752		0.733648113	0	[illegible]
FBgn0030441	CG2209 gene product from transcript	0.130116168	0.082464995		0.130116168	0	[illegible]
FBgn0030447	CG2200 gene product from transcript	1351.672647	-0.169076404	0.979231897	1351.672647	-0.150786614	[illegible]
FBgn0030451	CG15717 gene product from transcrip	216.0850525	0.283660579	0.902424733	216.0850525	0.893254686	[illegible]
FBgn0030452	Major Facilitator Superfamily Transport	39.56540434	-0.292051111	0.954378726	39.56540434	-0.174631999	[illegible]
FBgn0052645	CG32645 gene product from transcrip	85.69462351	0.207399741	0.960742862	85.69462351	0.285313407	[illegible]
FBgn0030456	CG4332 gene product from transcript	914.2193548	-0.065582655	0.994497358	914.2193548	0.069121859	[illegible]
FBgn0030457	CG12096 gene product from transcrip	578.067016	-0.12009541	0.974745767	578.067016	0.101426372	[illegible]
FBgn0052640	CG32640 gene product from transcrip	60.34991181	-0.38637286	0.908816464	60.34991181	-0.284035305	[illegible]
FBgn0052641	CG32641 gene product from transcrip	56.22137537	-0.062052649	0.995584438	56.22137537	-0.261584504	[illegible]
FBgn0029133	CG1591 gene product from transcript	3232.197152	-0.138866957	0.945699553	3232.197152	0.284879912	[illegible]
FBgn0004456	multiple edematous wings	1309.15275	0.142941203	0.953305045	1309.15275	0.274370468	[illegible]
FBgn0030462	CG15742 gene product from transcrip	0.905631379	0.183469702		0.905631379	0.042877736	[illegible]
FBgn0052639	CG32639 gene product from transcrip	1.846738286	0.220350307	0.958499814	1.846738286	-0.086392645	[illegible]
FBgn0030460	CG2453 gene product from transcript	450.6332605	0.031629908	0.998322595	450.6332605	0.072071162	[illegible]
FBgn0030459	CG12723 gene product from transcrip	1.169765605	0.156845271		1.169765605	0.325265139	[illegible]
FBgn0025463	Brahma associated protein 60kD	3946.905952	-0.041668067	0.9945211	3946.905952	-0.174041122	[illegible]
FBgn0030455	CG4318 gene product from transcript	1.002017511	0.257263602		1.002017511	-0.019662676	[illegible]
FBgn0052638	CG32638 gene product from transcrip	1776.961388	0.202536596	0.838180007	1776.961388	0.121314113	[illegible]
FBgn0030449	Ferritin 3 heavy chain homologue	2.108387013	0.286505455	0.865273684	2.108387013	0.085125438	[illegible]
FBgn0010303	hemipterous	2151.718293	0.183026365	0.91488891	2151.718293	0.167644814	[illegible]
FBgn0030443	CG12715 gene product from transcrip	5765.915527	0.652154117	0.103106829	5765.915527	0.589593354	[illegible]
FBgn0030440	CG15719 gene product from transcrip	0.985177619	0.134475156		0.985177619	0.14095326	[illegible]
FBgn0030439	CG12716 gene product from transcrip	0.881865168	0		0.881865168	0.33230769	[illegible]
FBgn0030437	hector	12.68688129	-0.208484513	0.982540663	12.68688129	-0.201692085	[illegible]

FBgn0000346	comatose	254.4228021	-0.04857786	0.994876798	254.4228021	0.022556434	0.993214629
FBgn0030465	CG15743 gene product from transcrip	812.5786405	0.177921312	0.856571339	812.5786405	0.063564571	0.95188184
FBgn0030466	CG15744 gene product from transcrip	605.3119096	0.026517975	0.998322595	605.3119096	-0.00221801	1
FBgn0030468	CG1622 gene product from transcript	1179.389045	-0.085928479	0.993127879	1179.389045	-0.225650578	0.820753979
FBgn0040309	thioredoxin peroxidase 1	5142.471147	0.109041939	0.989760074	5142.471147	0.279197551	0.775844637
FBgn0052636		11.42181621	0.050079915	0.99764662	11.42181621	0.238075657	0.920127339
FBgn0030474	CG15747 gene product from transcrip	584.8391717	-0.180489695	0.880088833	584.8391717	-0.314349495	0.490134099
FBgn0030477	doublesex-Mab related 11E	6.755969368	-0.044529361	0.998322595	6.755969368	0.475897537	0.74624482
FBgn0030478	CG1640 gene product from transcript	6967.53274	0.116811926	0.979231897	6967.53274	0.298335541	0.688211539
FBgn0030480	CG1660 gene product from transcript	266.8771417	0.108157162	0.993127879	266.8771417	0.392624195	0.677282764
FBgn0030481	CG1662 gene product from transcript	780.1127111	-0.038875998	0.995584438	780.1127111	0.160618797	0.904406238
FBgn0030482	CG1673 gene product from transcript	2958.742247	0.206963562	0.971565969	2958.742247	0.555778917	0.53448937
FBgn0030484	Glutathione S transferase T4	1965.968262	-0.319214479	0.556956355	1965.968262	-0.142772831	0.876929065
FBgn0030486	CG1716 gene product from transcript	985.7463519	0.042070619	0.9945211	985.7463519	-0.057834446	0.968777026
FBgn0030492	CG15754 gene product from transcrip	244.8662424	0.129967753	0.994497358	244.8662424	0.314657726	0.854980937
FBgn0030493	CG15756 gene product from transcrip	348.1061482	-0.371940833	0.912494615	348.1061482	-1.008267157	0.194197573
FBgn0030494	Cuticular protein 12A	1725.233872	0.836425791	0.23573308	1725.233872	0.726309602	0.35654367
FBgn0030495	CG11816 gene product from transcrip	3.38548552	-0.055430479	0.995584438	3.38548552	-0.248435127	
FBgn0030485	CG1998 gene product from transcript	447.0515967	-0.11584614	0.966782845	447.0515967	0.128691981	0.882181224
FBgn0030483	CG12725 gene product from transcrip	1.756713091	0.321538611	0.815249619	1.756713091	-0.136539419	
FBgn0030479	CG1987 gene product from transcript	1966.024223	-0.084608071	0.985645036	1966.024223	-0.197615841	0.773294804
FBgn0010198	Ribosomal protein S15Aa	30855.62151	-0.016126654	0.998322595	30855.62151	-0.013309288	0.996076098
FBgn0030467	CG1764 gene product from transcript	271.1366155	-0.168086791	0.97064214	271.1366155	-0.050506036	0.984795555
FBgn0052631	CG32631 gene product from transcrip	11.66384946	0.946249019	0.281813143	11.66384946	0.53332826	0.696528331
FBgn0052633	CG32633 gene product from transcrip	3156.785691	-0.382035887	0.862079032	3156.785691	-0.57879774	0.564931665
FBgn0030499	CG11178 gene product from transcrip	716.7885391	-0.373447528	0.466307248	716.7885391	-0.492942401	0.208714782
FBgn0030501	BthD selenoprotein	253.8539207	0.17428077	0.924674154	253.8539207	0.042910125	0.981228863
FBgn0030503	Transport and Golgi organization 2	1320.188572	-0.35436381	0.652274434	1320.188572	-0.287630718	0.71658922
FBgn0030505	NFAT homolog	1709.859367	0.22256613	0.939583701	1709.859367	0.229706925	0.848588436
FBgn0030507	CG11164 gene product from transcrip	270.058073	0.039865066	0.994876798	270.058073	-0.096001737	0.939574224
FBgn0030508	CG15760 gene product from transcrip	91.69115286	-0.006892176	0.998322595	91.69115286	-0.238717343	0.861074999
FBgn0030509	CG11162 gene product from transcrip	341.0128036	-1.657916349	0.002988383	341.0128036	-1.364341095	0.030964771
FBgn0030511	CG11158 gene product from transcrip	423.6559609	0.115175675	0.993509441	423.6559609	0.227401472	0.894246428
FBgn0002878	mutagen-sensitive 101	332.5546864	0.1632732	0.959208615	332.5546864	0.315265138	0.654632002
FBgn0030518	CG11134 gene product from transcrip	1717.935524	-0.063713569	0.9945211	1717.935524	0.034912696	0.990025502
FBgn0030519	CG11151 gene product from transcrip	13391.35273	-0.122296132	0.979231897	13391.35273	0.02470466	0.991444605
FBgn0020909	CG11130 gene product from transcrip	368.0715766	-0.087008299	0.994497358	368.0715766	-0.020253893	0.995108225
FBgn0030528	CG11095 gene product from transcrip	263.3585214	0.24436216	0.876449859	263.3585214	0.180483329	0.876744307
FBgn0027537	Nucleoporin 93kD-1	1392.441028	-0.059662461	0.993127879	1392.441028	-0.018693857	0.99009081
FBgn0052626	AMP deaminase	6087.369687	-0.201198707	0.817348474	6087.369687	-0.330481712	0.421896183
FBgn0030530	Ajuba LIM protein	1882.386212	-0.094482656	0.979231897	1882.386212	-0.203925987	0.77393101
FBgn0030529	Chloride intracellular channel	2844.490972	-0.039752577	0.995120986	2844.490972	0.140709981	0.906537259
FBgn0030525	CG10996 gene product from transcrip	2.806357569	0.361904824	0.862118921	2.806357569	0.253596546	
FBgn0030524	CG10993 gene product from transcrip	1.909326079	0.518677868	0.341648199	1.909326079	0.016744335	
[illegible]	[illegible]	[illegible]	[illegible]	[illegible]	[illegible]	[illegible]	[illegible]

FBgn0030895	CG7135 gene product from transcript	18.59341787	0.489311562	0.810559204	18.59341787	0.475120359	0.750777293	18.59341787	-0.751620244	0.311895685	18.59341787
FBgn0052553	CG32553 gene product from transcrip	0.264062816	0.057107491		0.264062816	-0.040397821		0.264062816	-0.101672424		0.264062816
FBgn0030894	CG7192 gene product from transcript	690.5732066	-0.295901485	0.635933989	690.5732066	-0.089917256	0.939842065	690.5732066	0.472145053	0.114375594	690.5732066
FBgn0030890	CG7536 gene product from transcript	1481.152565	-0.356198154	0.443425647	1481.152565	-0.021230888	0.991000388	1481.152565	0.236023324	0.553746085	1481.152565
FBgn0030886	CG12672 gene product from transcrip	8.097963662	0.225258661	0.978233016	8.097963662	-0.111644126	0.971833087	8.097963662	-0.045108191	0.976641222	8.097963662
FBgn0030883	CG7772 gene product from transcript	228.4643317	-0.053926271	0.9945211	228.4643317	0.211815156	0.851340756	228.4643317	-0.272752076	0.627777117	228.4643317
FBgn0030877	Actin-related protein 8	441.8159097	-0.147177007	0.990262271	441.8159097	-0.22937318	0.910666561	441.8159097	0.042170825	0.974483245	441.8159097
FBgn0083228	Frequenin 2	96.23680549	3.34E-05	0.999994709	96.23680549	0.401497016	0.776273613	96.23680549	0.240778342	0.811182614	96.23680549
FBgn0030900	Holes in muscle	37.2945203	0.376104018	0.855884878	37.2945203	0.17646324	0.934188786	37.2945203	0.106759234	0.92280061	37.2945203
FBgn0030911	ncRNA	0.642800959	0.01456241		0.642800959	-0.033275944		0.642800959	-0.175916999		0.642800959
FBgn0030914	CG6106 gene product from transcript	558.3835361	0.246460246	0.961031911	558.3835361	0.417581372	0.730802877	558.3835361	-1.23137832	0.00922995	558.3835361
FBgn0030915	CG6179 gene product from transcript	257.5041305	-0.00976039	0.998322595	257.5041305	-0.062586051	0.984488479	257.5041305	0.14225289	0.88049888	257.5041305
FBgn0022355	Transferrin 1	16622.69209	0.982523436	2.99E-05	16622.69209	0.693313965	0.013703446	16622.69209	-1.210113808	5.68E-08	16622.69209
FBgn0030918	CG15056 gene product from transcrip	2.111891769	0.611773725	0.28390629	2.111891769	0.019754872		2.111891769	-0.645168317	0.141137886	2.111891769
FBgn0030921	CG6290 gene product from transcript	0			0			0			0
FBgn0052551	CG32551 gene product from transcrip	0			0			0			0
FBgn0052548	CG32548 gene product from transcrip	6.061658136	0.961097706	0.04433664	6.061658136	0.088308947		6.061658136	-0.878926738	0.048359994	6.061658136
FBgn0052549	CG32549 gene product from transcrip	3942.372737	0.194058922	0.901320232	3942.372737	-0.140616445	0.897840331	3942.372737	0.183007388	0.709793513	3942.372737
FBgn0008635	Coat Protein (coatomer) beta	7047.031176	0.212744486	0.926642056	7047.031176	0.271389223	0.770169252	7047.031176	-0.366144716	0.449248338	7047.031176
FBgn0004957	porcupine	597.8506841	-0.13658712	0.978641661	597.8506841	-0.262818669	0.792524256	597.8506841	0.172635421	0.793183147	597.8506841
FBgn0030913	CG6123 gene product from transcript	36.36619306	0.335972196	0.945847297	36.36619306	0.28950466	0.895202908	36.36619306	-0.139741586	0.916179736	36.36619306
FBgn0030912	CG6023 gene product from transcript	90.21876438	-0.397932816	0.764963416	90.21876438	-0.183155758	0.915954217	90.21876438	0.3737203	0.573618717	90.21876438
FBgn0004956	outstretched	53.5571268	0.21651835	0.966988857	53.5571268	-0.009045585	0.999024272	53.5571268	-0.06486422	0.953962511	53.5571268
FBgn0030905	CG15059 gene product from transcrip	0.650514935	0.171513143		0.650514935	-0.020350363		0.650514935	-0.194565846		0.650514935
FBgn0030904	unpaired 2	5.508601849	0.09603603	0.994497358	5.508601849	-0.397170174		5.508601849	-0.143035678	0.913812954	5.508601849
FBgn0030899	HES-related	0.174504111	0.073112477		0.174504111	0		0.174504111	0.014071434		0.174504111
FBgn0030931	CG6318 gene product from transcript	6.739342494	-0.118457927	0.994497358	6.739342494	0.007350042	1	6.739342494	0.125821044	0.92312512	6.739342494
FBgn0027087	Histidyl-tRNA synthetase	3511.075839	0.102348779	0.991146172	3511.075839	0.10838891	0.94714921	3511.075839	-0.378449083	0.446236813	3511.075839
FBgn0030938	CG15047 gene product from transcrip	4.854298765	0.217000962	0.975999817	4.854298765	0.228278085		4.854298765	-0.48152191	0.558621001	4.854298765
FBgn0052546	transfer RNA:CR32546	0			0			0			0
FBgn0030943	CG6540 gene product from transcript	589.5754512	0.111122252	0.985645036	589.5754512	0.154940376	0.904246991	589.5754512	-0.039102019	0.963350754	589.5754512
FBgn0001079	fused	378.3467481	-0.12578432	0.979231897	378.3467481	-0.02871044	0.990025502	378.3467481	0.283643978	0.57763798	378.3467481
FBgn0004959	phantom	932.4851072	0.28533746	0.738624522	932.4851072	0.075980572	0.959037694	932.4851072	-0.363241512	0.35680648	932.4851072
FBgn0030949	CG6585 gene product from transcript	1.037480818	0.209404851		1.037480818	0.06618405		1.037480818	-0.064755728		1.037480818
FBgn0030952	CG12609 gene product from transcrip	0.499093515	0.181996391		0.499093515	0		0.499093515	-0.182795121		0.499093515
FBgn0030951	CG6873 gene product from transcript	1.180727513	0.229873428		1.180727513	0		1.180727513	-0.230355641		1.180727513
FBgn0010383	Cytochrome P450-18a1	425.7738341	0.503330274	0.718153804	425.7738341	0.06686512	0.985332517	425.7738341	0.011605024	0.992842751	425.7738341
FBgn0030947	CG6696 gene product from transcript	5.375747891	0.226230731	0.976370706	5.375747891	0.368505497		5.375747891	-0.229016113	0.838202313	5.375747891
FBgn0030946	CG6659 gene product from transcript	1740.94254	-0.055468223	0.99157593	1740.94254	-0.072253392	0.933048047	1740.94254	0.065075172	0.873683125	1740.94254
FBgn0030945	CG6632 gene product from transcript	257.4941098	0.038884123	0.998322595	257.4941098	-0.018235895	0.997246866	257.4941098	0.034651508	0.979863971	257.4941098
FBgn0030944	CG6617 gene product from transcript	2417.290774	-0.205378916	0.832566727	2417.290774	-0.186820793	0.796384175	2417.290774	0.229394666	0.547981807	2417.290774
FBgn0027335	Rab11 interacting protein	4136.999916	0.105570518	0.985645036	4136.999916	-0.049487797	0.980691994	4136.999916	0.080897691	0.912140543	4136.999916
FBgn0030941	wengen	917.0215603	-0.21696672	0.764963416	917.0215603	-0.327575376	0.423277688	917.0215603	0.122848302	0.778154953	917.0215603
FBgn0030940	CG15040 gene product from transcrip	6.217069486	0.957241849	0.118363619	6.217069486	0.12010589	0.95955656	6.217069486	-0.962068708	0.068154475	6.217069486
FBgn0030937	CG15042 gene product from transcrip	0.527145273	0.090446677		0.527145273	-0.032909123		0.527145273	0.007415767		0.527145273

[illegible]

FBgn0036756	CG5582 gene product from transcript	501.5998806	-0.143149472	0.972945531	501.5998806
FBgn0052191	CG32191 gene product from transcrip	893.4133318	-0.41369977	0.316901578	893.4133318
FBgn0052187	CG32187 gene product from transcrip	146.9300395	0.22270542	0.954378726	146.9300395
FBgn0036754	CG5589 gene product from transcript	215.7419143	0.085543861	0.993466746	215.7419143
FBgn0052190	CG32190 gene product from transcrip	8261.142976	0.027693673	0.995120986	8261.142976
FBgn0036732	Organic anion transporting polypeptide	3282.925959	-0.163382846	0.897904102	3282.925959
FBgn0052185	elevated during infection	37.23665611	1.044928722	0.191663663	37.23665611
FBgn0036734	CG7564 gene product from transcript	1394.776219	-0.126461744	0.945699553	1394.776219
FBgn0036738	CG7542 gene product from transcript	1.529770067	-0.08457707		1.529770067
FBgn0036741	CG7510 gene product from transcript	696.0259869	-0.414001111	0.694459829	696.0259869
FBgn0036742	CG7497 gene product from transcript	139.9720358	-0.150651386	0.979231897	139.9720358
FBgn0036745	CG7484 gene product from transcript	821.5426163	0.117829318	0.987458415	821.5426163
FBgn0052183	CG32183 gene product from transcrip	53.29104267	-0.222311998	0.971542212	53.29104267
FBgn0036749	CG7460 gene product from transcript	172.3925716	0.494609518	0.625787581	172.3925716
FBgn0052182	CG32182 gene product from transcrip	19.40152061	-0.503333	0.791227483	19.40152061
FBgn0052181	CG32181 gene product from transcrip	7.756097013	0.091109505	0.9945211	7.756097013
FBgn0036752	Adenosine deaminase-related growth fa	630.631346	-0.573787465	0.290861962	630.631346
FBgn0043025	Male-specific insect derived growth fac	11.14061773	0.421346974	0.886446899	11.14061773
FBgn0036751	Adenosine deaminase-related growth fa	0.590363756	0.212547667		0.590363756
FBgn0036750	CG6034 gene product from transcript	53.8452191	-0.114351271	0.994497358	53.8452191
FBgn0036747	CG6052 gene product from transcript	0.489279705	0.099554575		0.489279705
FBgn0036746	CREB-regulated transcription coactivato	1331.595477	0.178528162	0.967901085	1331.595477
FBgn0052179	Keren	219.9381456	-0.244797773	0.931455573	219.9381456
FBgn0004401	Protein on ecdysone puffs	13187.67091	-0.0917842	0.979231897	13187.67091
FBgn0036740	CG6259 gene product from transcript	2655.369387	0.036626448	0.994876798	2655.369387
FBgn0023197	Jonah 74E	5554.466396	-4.917798824	9.14E-64	5554.466396
FBgn0036735	Enhancer of decapping 3	1007.704477	-0.007267745	0.998322595	1007.704477
FBgn0036733	U4-U6 small nuclear riboprotein factor	767.8419806	-0.028467891	0.996983104	767.8419806
FBgn0052177	Nedd4 family interacting protein	660.2361627	-0.21106496	0.923470826	660.2361627
FBgn0036703	CG7707 gene product from transcript	1.005847992	0.167643754		1.005847992
FBgn0036705	CG13723 gene product from transcrip	0.287010221	0.020450848		0.287010221
FBgn0036707	CG13724 gene product from transcrip	0			0
FBgn0036714	CG7692 gene product from transcript	347.2056766	-0.164215057	0.919902729	347.2056766
FBgn0015550	target of Poxn	137.9773555	0.504267002	0.297967072	137.9773555
FBgn0040793	CG7630 gene product from transcript	5233.080149	-0.028036879	0.998322595	5233.080149
FBgn0036725	CG18265 gene product from transcrip	48.98460005	0.042539095	0.99723561	48.98460005
FBgn0036726	CG7603 gene product from transcript	1858.971714	-0.011551704	0.998322595	1858.971714
FBgn0036727	CG7589 gene product from transcript	451.730719	-0.890026851	0.035141769	451.730719
FBgn0036728	CG7580 gene product from transcript	2374.582386	0.281998162	0.8856311	2374.582386
FBgn0036730	quijote	8.962932437	0.644764285	0.649039738	8.962932437
FBgn0036731	CG6333 gene product from transcript	2.798468154	0.060714551	0.995120986	2.798468154
FBgn0036729	CG13733 gene product from transcrip	9.956927806	0.733485347	0.52480889	9.956927806
FBgn0036723	CG12229 gene product from transcrip	22.10118295	0.609306959	0.68934315	22.10118295
FBgn0052174	CG32174 gene product from transcrip	2.431228467	0.09626794	0.994497358	2.431228467
FBgn0042641	fringe connection	0			0
FBgn0053052	CG33052 gene product from transcrip	400.974955	0.08854519	0.989856236	400.974955
FBgn0053051	CG33051 gene product from transcrip	438.0090449	0.052643303	0.994497358	438.0090449
FBgn0027660	bloated tubules	1901.793624	0.182757134	0.819945851	1901.793624
FBgn0026197	CG32172 gene product from transcrip	1088.595962	-0.374172984	0.892718858	1088.595962
FBgn0036717	CG13731 gene product from transcrip	18003.30669	0.371621073	0.792866354	18003.30669
FBgn0036716	CG13728 gene product from transcrip	2.648890616	0.194885266	0.978641661	2.648890616
FBgn0036715	Cadherin 74A	815.493208	-0.416511717	0.516103614	815.493208
FBgn0036712	brivido-2	13.08025153	1.69931723	0.001494306	13.08025153
FBgn0036711	CG13727 gene product from transcrip	2.79999227	0.216669378	0.968718701	2.79999227
FBgn0052173	transfer RNA:CR32173	0.765954064	-0.038941524		0.765954064
FBgn0036710	CG6479 gene product from transcript	297.9983909	-0.189055888	0.907005324	297.9983909
FBgn0036709	Odorant receptor 74a	0.313921675	0.050517759		0.313921675
FBgn0036708	CG13725 gene product from transcrip	0.368977348	0.196066888		0.368977348
FBgn0036706	CG6485 gene product from transcript	2.048798376	0.415986927	0.341648199	2.048798376
FBgn0036704	CG6497 gene product from transcript	0.526006688	0.145690189		0.526006688
FBgn0036702	CG6512 gene product from transcript	4207.838318	0.272553529	0.614907394	4207.838318
FBgn0052170	CG32170 gene product from transcrip	283.499212	-0.001481409	0.999629143	283.499212
FBgn0036676	CG13028 gene product from transcrip	54.72103677	0.252631008	0.963043532	54.72103677
FBgn0010352	Neural conserved at 73EF	9688.367026	-0.119401343	0.966865338	9688.367026
FBgn0036686	CG7728 gene product from transcript	977.8745324	0.0871385	0.988354972	977.8745324
FBgn0036688	Fermitin 2	488.0210332	0.205193392	0.847707545	488.0210332
FBgn0036691	bad egg	330.18429	-0.288352732	0.950391106	330.18429
FBgn0036695	PAPS transporter 2	778.4014568	0.109005168	0.974005319	778.4014568
FBgn0036697	CG7725 gene product from transcript	762.0620573	-0.381811733	0.433061354	762.0620573
FBgn0036698	CG7724 gene product from transcript	199.0356808	0.170930029	0.975044255	199.0356808
FBgn0036696	CG14057 gene product from transcrip	112.2437219	-0.107061962	0.991865251	112.2437219
FBgn0036690	Insulin-like peptide 8	6.016935602	-0.678383684	0.444310276	6.016935602
FBgn0011293	antennal protein 10	2.002613974	0.098183594	0.994497358	2.002613974
FBgn0036687	CG6652 gene product from transcript	6.348853297	0.979975826	0.097479833	6.348853297
FBgn0036684	CG3764 gene product from transcript	1261.298389	-0.066080002	0.994497358	1261.298389
FBgn0036679	CG13022 gene product from transcrip	0.198065639	0		0.198065639
FBgn0036678	CG11905 gene product from transcrip	277.2208809	-0.341767649	0.849417619	277.2208809
FBgn0036677	CG13023 gene product from transcrip	122.6326791	0.723287813	0.316901578	122.6326791
FBgn0028693	Regulatory particle non-ATPase 12	5680.195708	-0.22167075	0.767778996	5680.195708
FBgn0052163	CG32163 gene product from transcrip	21.70808727	-0.170887495	0.989856236	21.70808727
FBgn0042178	CG32165 gene product from transcrip	148.6246342	0.259069841	0.926642056	148.6246342
FBgn0042177	CG32164 gene product from transcrip	497.7008701	0.029385428	0.998322595	497.7008701
FBgn0025558	CG4101 gene product from transcript	29.94566006	-0.059338528	0.995682185	29.94566006
FBgn0041721	ncRNA	25.80355752	0.273411247	0.966865338	25.80355752
FBgn0036652	CG13032 gene product from transcrip	3.9080857	1.072420426	0.036814888	3.9080857
FBgn0036654	CG9692 gene product from transcript	8.580174668	0.384611543	0.891773597	8.580174668
FBgn0000414	Disabled	1236.871241	0.195018607	0.887161992	1236.871241
FBgn0036655	CG13031 gene product from transcrip	0			0
FBgn0043577	CG9697 gene product from transcript	55.7914058	-0.144733681	0.969368572	55.7914058
FBgn0036659	CG9701 gene product from transcript	2417.612427	0.298705198	0.750684567	2417.612427
FBgn0004108	Neurotactin	1376.956885	-0.381868627	0.739076299	1376.956885
FBgn0036661	CG9705 gene product from transcript	7565.22381	-0.022010984	0.998322595	7565.22381
FBgn0036662	CG9706 gene product from transcript	705.023481	0.19882427	0.886089446	705.023481
FBgn0021768	CG9710 gene product from transcript	1848.867346	-0.178451816	0.917248088	1848.867346
FBgn0036666	Tumor susceptibility gene 101 ortholog	2075.800953	0.005491845	0.998322595	2075.800953
FBgn0036668	CG9715 gene product from transcript	1294.96823	-0.099695558	0.99157593	1294.96823
FBgn0052161	CG32161 gene product from transcrip	1.3329402	0.567069159		1.3329402
FBgn0003410	seven in absentia	1406.251838	0.244217647	0.878994604	1406.251838
FBgn0036670	CG13029 gene product from transcrip	5.464534013	0.330148173	0.945056035	5.464534013
FBgn0036671	CG9951 gene product from transcript	803.1395633	-0.115757498	0.954378726	803.1395633
FBgn0003250	Rhodopsin 4	6.1719002	-0.287876313	0.961442281	6.1719002
FBgn0036667	CG9669 gene product from transcript	982.0806401	0.289319085	0.706612155	982.0806401
FBgn0036665	CG13024 gene product from transcrip	337.0495086	-0.23512588	0.966157244	337.0495086
FBgn0036663	CG9674 gene product from transcript	6720.256514	0.148485999	0.943722455	6720.256514
FBgn0025582	Int6 homologue	6447.584347	-0.051109124	0.994497358	6447.584347
FBgn0036660	CG13025 gene product from transcrip	286.8940991	-0.128729832	0.963501097	286.8940991
FBgn0004556	Dead box protein 73D	242.3428624	0.013599666	0.998322595	242.3428624
FBgn0043578	CG9681 gene product from transcript	370.6729347	0.25494745	0.950976319	370.6729347
FBgn0036656	CG13026 gene product from transcrip	0.978315807	-0.062159703		0.978315807
FBgn0063485	CG3849 gene product from transcript	0			0
FBgn0040512	Coat Protein (coatomer) zeta	2396.382105	0.165362762	0.966062204	2396.382105
FBgn0010223	G protein alpha f subunit	346.770665	-0.489649244	0.235453186	346.770665
FBgn0000017	Abl tyrosine kinase	1893.38962	0.137790664	0.972330876	1893.38962
FBgn0017572	CG4083 gene product from transcript	2272.819535	-0.036880367	0.9945211	2272.819535
FBgn0004465	Suppressor of ref(2)P sterility	1198.248447	-0.139526642	0.985645036	1198.248447
FBgn0002284	Proteasome beta6 subunit	6012.449427	-0.241593453	0.767292414	6012.449427
FBgn0036648	CG4098 gene product from transcript	165.2501573	-0.350587625	0.667694616	165.2501573
FBgn0027500	spindle defective 2	496.7595432	0.168683704	0.936159146	496.7595432
FBgn0036643	Syntaxin 8	680.9467864	-0.47677563	0.44762312	680.9467864
FBgn0036601	CG13063 gene product from transcrip	479.5477428	0.836018158	0.283716365	479.5477428
FBgn0052160	ncRNA	0.481675811	0.039476416		0.481675811
FBgn0042201	Neuropeptide-like precursor 3	1.199423383	0.055594008		1.199423383
FBgn0036606	CG13060 gene product from transcrip	25.91715399	0.208104353	0.979231897	25.91715399
FBgn0036610	CG13058 gene product from transcrip	2.376655843	-0.091675543	0.994497358	2.376655843
FBgn0053061	CG33061 gene product from transcrip	2.349127041	0.377708458	0.774842704	2.349127041
FBgn0053060	CG33060 gene product from transcrip	1.326888112	0.210301657		1.326888112
FBgn0040074	CG13057 gene product from transcrip	0.474889005	0		0.474889005
FBgn0040794	CG13056 gene product from transcrip	0.305393275	-0.074425199		0.305393275
FBgn0036612	CG4998 gene product from transcript	4.278215132	-0.062582973	0.995584438	4.278215132
FBgn0053257	CG33257 gene product from transcrip	0			0
FBgn0260460	mitochondrial ribosomal protein S34	417.9144765	0.168819503	0.918245666	417.9144765
FBgn0052357	transfer RNA:CR32357	0.416004798	0		0.416004798
FBgn0036617	Cuticular protein 72Ea	1.907250169	0.17291277	0.975999817	1.907250169
FBgn0036618	Cuticular protein 72Eb	1.981696357	-0.227360289	0.953305045	1.981696357
FBgn0036620	CG4842 gene product from transcript	356.9273527	0.344366689	0.843363315	356.9273527
FBgn0042137	CG18814 gene product from transcrip	68.14587288	0.416042171	0.846500794	68.14587288
FBgn0036624	RING-associated factor 2	583.7079854	0.104919823	0.988216049	583.7079854
FBgn0036627	Gag related protein	201.6302812	0.024120766	0.998322595	201.6302812
FBgn0028399	CG4672 gene product from transcript	6163.998652	-0.235349941	0.857339354	6163.998652
FBgn0053158	CG33158 gene product from transcrip	581.9322194	0.068579269	0.994497358	581.9322194
FBgn0003515	scarlet	324.2191019	0.313028103	0.910739253	324.2191019
FBgn0036639	CG4229 gene product from transcript	0.48594262	0.150100589		0.48594262
FBgn0036641	survival motor neuron	679.9637425	-0.141680468	0.984185333	679.9637425
FBgn0026061	Multiple inositol polyphosphate phosp	594.5937355	-0.772413068	0.09488849	594.5937355
FBgn0036638	CG13033 gene product from transcrip	52.07689251	1.132011019	0.108868641	52.07689251
FBgn0004569	argos	204.387387	-0.174558474	0.953538331	204.387387
FBgn0027080	Tyrosyl-tRNA synthetase	3322.04148	0.12902375	0.963043532	3322.04148
FBgn0036629	CG4573 gene product from transcript	79.27633446	-0.008724104	0.998322595	79.27633446
FBgn0036623	CG4729 gene product from transcript	2910.569359	0.036581072	0.994876798	2910.569359
FBgn0036622	CG4753 gene product from transcript	993.9213705	-0.526403149	0.72893315	993.9213705
FBgn0036621	roquin	2483.057274	0.183780893	0.967901085	2483.057274
FBgn0036619	Cuticular protein 72Ec	5.627768298	-0.015880464	0.998322595	5.627768298
FBgn0011693	Photoreceptor dehydrogenase	1.710556799	-0.136381577	0.988354972	1.710556799
FBgn0036614	CG4925 gene product from transcript	496.7796109	-0.193220802	0.917248088	496.7796109
FBgn0036608	CG13040 gene product from transcrip	0.521631789	-0.037179627		0.521631789
FBgn0036605	CG13041 gene product from transcrip	1.772530761	-0.129271626	0.979231897	1.772530761
FBgn0036602	CG13042 gene product from transcrip	8.051155499	0.282570189	0.962738886	8.051155499
FBgn0036600	CG13043 gene product from transcrip	585.8881094	0.818224537	0.336432827	585.8881094
FBgn0044028	CG13076 gene product from transcrip	130.3208654	0.100685008	0.990957786	130.3208654
FBgn0036560	CG5895 gene product from transcript	64.47279939	0.057522511	0.995584438	64.47279939
FBgn0005536	Myosin binding subunit	2681.760051	0.042652527	0.994497358	2681.760051
FBgn0036563	CG13075 gene product from transcrip	1158.28726	1.771637015	1.13E-15	1158.28726
FBgn0036566	Chloride channel-c	3404.097814	-0.207358988	0.823279465	3404.097814
FBgn0036568	male sterile (3) 72Dt	8.713720176	1.42232965	0.00224673	8.713720176
FBgn0036569	CG5414 gene product from transcript	190.9044763	0.129664063	0.977300259	190.9044763
FBgn0010280	TBP-associated factor 4	2753.607988	0.039480939	0.994876798	2753.607988
FBgn0036571	CG12272 gene product from transcrip	994.0172208	0.070554188	0.994497358	994.0172208
FBgn0011016	Signal sequence receptor beta	5243.957786	0.35318197	0.669321246	5243.957786
FBgn0052153	transfer RNA:CR32153	0			0
FBgn0036579	CG5027 gene product from transcript	512.6413767	-0.199877536	0.872095585	512.6413767
FBgn0036581	Mediator complex subunit 10	970.9244879	0.036257708	0.995120986	970.9244879
FBgn0036585	CG13071 gene product from transcrip	0.430896145	0		0.430896145
FBgn0036586	CG13070 gene product from transcrip	0			0
FBgn0040798	CG13069 gene product from transcrip	6.216901387	0.711869712	0.542487958	6.216901387
FBgn0036588	CG13068 gene product from transcrip	15.32103023	0.771919518	0.469339745	15.32103023
FBgn0036589	CG13067 gene product from transcrip	700.7806001	0.556705673	0.660849346	700.7806001
FBgn0040797	CG13066 gene product from transcrip	0.411682288	0.052770824		0.411682288
FBgn0036590	CG13065 gene product from transcrip	0.761993891	0.05013006		0.761993891
FBgn0036598	CG4982 gene product from transcript	20.47165736	1.083370568	0.159386848	20.47165736
FBgn0036597	CG4962 gene product from transcript	1524.978222	0.734191334	0.438394678	1524.978222
FBgn0036596	CG13045 gene product from transcrip	38.19592503	0.501824625	0.792866354	38.19592503
FBgn0036595	CG13046 gene product from transcrip	168.4190084	0.038749579	0.997459641	168.4190084
FBgn0036594	CG13047 gene product from transcrip	21.73105552	0.949199751	0.273000848	21.73105552
FBgn0036593	CG13048 gene product from transcrip	0.229148987	0.059032177		0.229148987
FBgn0036592	CG13049 gene product from transcrip	0.980729553	0.432123456		0.980729553
FBgn0036591	CG13050 gene product from transcrip	0.646469963	0.013386691		0.646469963
FBgn0036587	CG4950 gene product from transcript	571.9205441	0.464843675	0.778638906	571.9205441
FBgn0040801	CG13053 gene product from transcrip	16.67007402	0.047924136	0.998322595	16.67007402
FBgn0036584	CG13054 gene product from transcrip	1.024153886	0.097655065		1.024153886
FBgn0036583	CG13055 gene product from transcrip	7.432469061	0.082478556	0.994876798	7.432469061
FBgn0036580	Programmed Cell Death 5	1459.708333	0.030129781	0.997459641	1459.708333
FBgn0036577	CG13073 gene product from transcrip	20.9475116	-0.367828878	0.915289583	20.9475116
FBgn0036576	CG5151 gene product from transcript	1390.625938	0.184816563	0.967485396	1390.625938
FBgn0052152	CG32152 gene product from transcrip	0			0
FBgn0036575	CG5157 gene product from transcript	181.3934595	-0.388114651	0.898912603	181.3934595
FBgn0003076	phosphoglucose mutase	13425.25274	0.048974607	0.995120986	13425.25274
FBgn0036570	Integrator 9	368.5819305	-0.043866813	0.994497358	368.5819305
FBgn0036567	CG13074 gene product from transcrip	2.964470828	-0.061026766	0.995584438	2.964470828
FBgn0036565	CG5235 gene product from transcript	250.3027664	0.110391847	0.979231897	250.3027664
FBgn0036531	CG6244 gene product from transcript	2.71727922	-0.06314867	0.995584438	2.71727922
FBgn0036532	CG13445 gene product from transcrip	59.09022506	1.937397173	0.000164241	59.09022506
FBgn0015025	CKII-alpha subunit interactor-1	95.04183779	-0.591932696	0.337177289	95.04183779
FBgn0040230	diablo	941.5364424	-0.011234365	0.998322595	941.5364424
FBgn0036536	CG12713 gene product from transcrip	119.4366222	0.032058028	0.99764662	119.4366222
FBgn0036545	CG17035 gene product from transcrip	597.749326	-0.134601508	0.988216049	597.749326
FBgn0036547	CG17032 gene product from transcrip	4927.425425	0.251007821	0.785231468	4927.425425
FBgn0036549	CG10516 gene product from transcrip	892.8462973	-0.065843138	0.994497358	892.8462973
FBgn0036550	CG17026 gene product from transcrip	1197.032288	-0.038816039	0.995584438	1197.032288
FBgn0036551	CG17029 gene product from transcrip	3791.390999	0.43457053	0.693891724	3791.390999
FBgn0036552	CG17028 gene product from transcrip	59.08878709	0.633160494	0.520647053	59.08878709
FBgn0036553	CG17027 gene product from transcrip	3742.753358	0.096445262	0.979231897	3742.753358
FBgn0000115	ADP ribosylation factor-like 1	2135.090627	0.229380948	0.949623474	2135.090627
FBgn0036557	mitochondrial ribosomal protein S31	709.1632294	0.318368257	0.829597852	709.1632294
FBgn0036556	CG5830 gene product from transcript	1315.374163	-0.106936904	0.988354972	1315.374163
FBgn0000212	brahma	3332.587801	-0.047960334	0.994497358	3332.587801
FBgn0000489	cAMP-dependent protein kinase 3	2296.952905	0.518186216	0.40244538	2296.952905
FBgn0052150	CG32150 gene product from transcrip	28.52623865	-0.050231739	0.99723561	28.52623865
FBgn0036538	CG15715 gene product from transcrip	531.9555629	-0.391006392	0.46766751	531.9555629
FBgn0036537	CG18081 gene product from transcrip	1014.185906	-0.584488984	0.388088666	1014.185906
FBgn0036498	CG13455 gene product from transcrip	3.009593965	0.633702937	0.411467005	3.009593965
FBgn0036499	CG7276 gene product from transcript	0.351502576	0.161356617		0.351502576
FBgn0036500	CG7275 gene product from transcript	504.8387944	0.06353509	0.994497358	504.8387944
FBgn0036501	CG7272 gene product from transcript	2152.662166	0.213123902	0.944197659	2152.662166
FBgn0000565	Ecdysone-induced protein 28/29kD	5461.739218	0.166482094	0.979231897	5461.739218
FBgn0036504	CG7463 gene product from transcript	0.268385325	0		0.268385325
FBgn0004396	Cyclic-AMP response element binding p	2116.04675	0.215979997	0.969626359	2116.04675
FBgn0036510	CG7427 gene product from transcript	916.1461722	0.153507268	0.963243134	916.1461722
FBgn0036512	CG16979 gene product from transcrip	727.3969474	-0.051527383	0.994497358	727.3969474
FBgn0036514	CG12301 gene product from transcrip	353.488527	-0.121881668	0.979231897	353.488527
FBgn0036515	CG12304 gene product from transcrip	1846.014197	0.144071729	0.975999817	1846.014197
FBgn0036518	Rho GTPase activating protein at 71E	1169.648703	-0.09603613	0.979231897	1169.648703
FBgn0036522	CG7372 gene product from transcript	1255.235548	-0.107790549	0.979231897	1255.235548
FBgn0004589	Ecdysone-induced gene 71Eb	30.28345806	0.458802059	0.840842244	30.28345806
FBgn0004591	Ecdysone-induced gene 71Ed	4.665325715	-0.038667038	0.996902828	4.665325715
FBgn0004594	Ecdysone-induced gene 71Eg	60.97324545	-0.351147454	0.938791798	60.97324545
FBgn0014849	CG7327 gene product from transcript	0.273866805	0		0.273866805
FBgn0014851	CG7325 gene product from transcript	0.168962095	0		0.168962095
FBgn0036527	CG7304 gene product from transcript	0			0
FBgn0036529	polypeptide GalNAc transferase 8	197.1300511	0.61633684	0.212375767	197.1300511
FBgn0010105	commissureless	395.7203374	0.260180141	0.72893315	395.7203374
FBgn0041160	CG7554 gene product from transcript	412.4131778	-0.111943632	0.982540663	412.4131778
FBgn0036528	CG7579 gene product from transcript	0.060151179	-0.097832662		0.060151179
FBgn0014850	CG7588 gene product from transcript	0			0
FBgn0014848	CG7594 gene product from transcript	0			0
FBgn0004593	Ecdysone-induced gene 71Ef	2.102974691	-0.175342415	0.956083993	2.102974691
FBgn0004592	Ecdysone-induced gene 71Ee	48045.40719	0.218594361	0.924143028	48045.40719
FBgn0004590	Ecdysone-induced gene 71Ec	3.824802683	-0.039353087	0.995584438	3.824802683
FBgn0004588	Ecdysone-induced gene 71Ea	78.91310224	-0.084275574	0.994876798	78.91310224
FBgn0036520	CG13449 gene product from transcrip	15.37706391	0.063980317	0.995584438	15.37706391
FBgn0036519	CG7650 gene product from transcript	468.3955479	0.000648519	0.999781966	468.3955479
FBgn0036516	CG7656 gene product from transcript	4677.768104	0.065614545	0.994497358	4677.768104
FBgn0036509	CG7739 gene product from transcript	1637.193534	0.261500677	0.732714402	1637.193534
FBgn0036505	CG7945 gene product from transcript	1412.083375	-0.120977016	0.934308488	1412.083375
FBgn0004228	midgut expression 1	880.9782357	-1.335441431	1.39E-05	880.9782357
FBgn0036503	CG13454 gene product from transcrip	4.82755288	-0.08467478	0.9945211	4.82755288
FBgn0036502	CG7841 gene product from transcript	358.9253657	0.513760725	0.532891895	358.9253657
FBgn0026738	CG7857 gene product from transcript	783.552115	-0.179907494	0.925130854	783.552115
FBgn0040805	CG12355 gene product from transcrip	34.42886562	0.108799322	0.994497358	34.42886562
FBgn0029114	CG6890 gene product from transcript	783.7761967	0.005147787	0.998322595	783.7761967
FBgn0036491	Bestrophin 4	0.744424682	0.125004307		0.744424682
FBgn0036494	CG7250 gene product from transcript	74.86120862	-0.244459103	0.938791798	74.86120862
FBgn0036495	CG33259 gene product from transcrip	0			0
FBgn0036497	CG7815 gene product from transcript	1.106932043	0.228582052		1.106932043
FBgn0036496	CG7804 gene product from transcript	6.344380501	0.863420241	0.068908222	6.344380501
FBgn0036492	Bestrophin 3	3.56328854	0.419046479	0.728029857	3.56328854
FBgn0026320	Twin of m4	702.5617374	0.283184947	0.900680408	702.5617374
FBgn0000216	Bearded	119.8142005	0.687956203	0.235098752	119.8142005
FBgn0040296	CG3396 gene product from transcript	70.2619139	-0.004956853	0.998322595	70.2619139
FBgn0036459	CG3349 gene product from transcript	1.175167462	0.058104837		1.175167462
FBgn0036460	CG5114 gene product from transcript	789.2099556	0.033019695	0.995120986	789.2099556
FBgn0002778	minidiscs	1020.026508	-0.036707188	0.995584438	1020.026508
FBgn0036461	Zinc/iron regulated transporter-related	1320.723699	-0.240482623	0.798887624	1320.723699
FBgn0023174	Proteasome beta2 subunit	6507.738047	-0.215999554	0.835894303	6507.738047
FBgn0047338	CG32148 gene product from transcrip	1.031790626	0.217650935		1.031790626
FBgn0036470	CG13463 gene product from transcrip	2.206648506	0.690422689	0.308402171	2.206648506
FBgn0027088	Glycyl-tRNA synthetase	2249.425053	0.115838922	0.963501097	2249.425053
FBgn0036479	CG13458 gene product from transcrip	34.35853876	0.298464569	0.950644306	34.35853876
FBgn0036480	CG17081 gene product from transcrip	514.5802652	0.114894601	0.969626359	514.5802652
FBgn0052847	CG32847 gene product from transcrip	8.788001517	-0.211969873	0.979231897	8.788001517
FBgn0036484	Peroxin 3	1313.420347	-0.138611833	0.900680408	1313.420347
FBgn0036485	CG6869 gene product from transcript	146.1909996	0.742159605	0.120522238	146.1909996
FBgn0036487	CG6876 gene product from transcript	1088.294309	-0.311311562	0.530023602	1088.294309
FBgn0036488	CG6878 gene product from transcript	824.8691942	0.437895514	0.737553542	824.8691942
FBgn0036490	CG6888 gene product from transcript	0.959341104	0.225496619		0.959341104
FBgn0036489	CG7011 gene product from transcript	790.0638724	0.348597782	0.479113758	790.0638724
FBgn0036486	CG7003 gene product from transcript	252.3693142	-0.019223001	0.998322595	252.3693142
FBgn0047178	CG32147 gene product from transcrip	357.1359066	0.052675554	0.995120986	357.1359066
FBgn0014002	Protein disulfide isomerase	46962.52254	0.512025691	0.216579251	46962.52254
FBgn0036483	CG12316 gene product from transcrip	445.7388428	0.106620685	0.99157593	445.7388428
FBgn0036482	CG13457 gene product from transcrip	2.841488366	0.714595757	0.270356652	2.841488366
FBgn0036481	CG16959 gene product from transcrip	328.9174128	-0.079713446	0.994497358	328.9174128
FBgn0036476	CG6945 gene product from transcript	254.7731196	-0.089438435	0.993059337	254.7731196
FBgn0036474	Odorant receptor 71a	2.854193224	0.122801074	0.99157593	2.854193224
FBgn0036471	CG13460 gene product from transcrip	15839.41064	0.731199392	0.135001564	15839.41064
FBgn0036469	CG18649 gene product from transcrip	1451.673261	1.091178588	0.015306372	1451.673261
FBgn0036468	CG13461 gene product from transcrip	632.1600245	-3.886970149	4.65E-24	632.1600245
FBgn0036467	CG12310 gene product from transcrip	33465.44517	0.459448949	0.363470291	33465.44517
FBgn0036466	CG18581 gene product from transcrip	89.36235326	0.749344254	0.3526135	89.36235326
FBgn0025776	intermediate neuroblasts defective	1.750135318	-0.191692114	0.972479043	1.750135318
FBgn0036465	Regulatory particle non-ATPase 12-rela	1.572401577	0.299827819		1.572401577
FBgn0036463	Reversion-inducing-cysteine-rich prote	308.6048445	-0.388824499	0.767292414	308.6048445
FBgn0036462	mitochondrial ribosomal protein L39	764.7800448	-0.177551643	0.963057925	764.7800448
FBgn0040487	Brother of Bearded A	0.514443529	0.156350424		0.514443529
FBgn0040809	CG13465 gene product from transcrip	0.155353517	0		0.155353517
FBgn0003388	shade	2168.609962	-0.285527174	0.72893315	2168.609962
FBgn0036433	CG9628 gene product from transcript	356.8775335	-0.056410064	0.995584438	356.8775335
FBgn0041604	dally-like	1347.721637	-0.303979608	0.750684567	1347.721637
FBgn0011928	transfer RNA:met3:70Fb	0.203940171	0		0.203940171
FBgn0036437	CG5048 gene product from transcript	6.631108505	0.764053893	0.377579161	6.631108505
FBgn0036438	CG13477 gene product from transcrip	0.819512743	0.219072865		0.819512743
FBgn0036441	CG13476 gene product from transcrip	1.699128176	0.24763875	0.870179008	1.699128176
FBgn0036442	CG13473 gene product from transcrip	2.184547406	-0.025275325	0.998322595	2.184547406
FBgn0036446	CG9384 gene product from transcript	582.3622267	-0.379258183	0.660849346	582.3622267
FBgn0036450	tudor domain containing 3 ortholog (H	1286.340012	0.059982697	0.994497358	1286.340012
FBgn0036451	CG9425 gene product from transcript	2431.357582	0.28524376	0.815846652	2431.357582
FBgn0036454	CG17839 gene product from transcrip	4298.920858	0.072326503	0.994497358	4298.920858
FBgn0001120	giant nuclei	7.841514727	-0.039561354	0.998322595	7.841514727
FBgn0029148	CG5258 gene product from transcript	746.6695663	0.12733137	0.963501097	746.6695663
FBgn0036449	brummer	1039.841919	-0.410701223	0.376545848	1039.841919
FBgn0036448	myopic	2517.640787	-0.0917817	0.993466746	2517.640787
FBgn0036447	CG17173 gene product from transcrip	2.958709087	0.714721083	0.37562217	2.958709087
FBgn0053260	CG33260 gene product from transcrip	61.09419636	-0.064809873	0.995584438	61.09419636
FBgn0036443	CG13471 gene product from transcrip	1.24864127	0.34214192		1.24864127
FBgn0036440	CG17177 gene product from transcrip	0.863084659	-0.034730699		0.863084659
FBgn0036439	CG13474 gene product from transcrip	0.056320698	0		0.056320698
FBgn0036436	CG4914 gene product from transcript	573.0309572	-0.306342913	0.893280346	573.0309572
FBgn0011927	transfer RNA:met3:70Fa	0			0
FBgn0036423	CG3919 gene product from transcript	48.6214896	0.494512989	0.72893315	48.6214896
FBgn0036426	CG9592 gene product from transcript	0.431739499	0.045605491		0.431739499
FBgn0026409	Mitochondrial phosphate carrier prote	15545.60798	-0.218170497	0.882488949	15545.60798
FBgn0040318	CG13475 gene product from transcrip	41.71653432	0.195314222	0.978641661	41.71653432
FBgn0028418	Leucokinin	0			0
FBgn0036428	CG9238 gene product from transcript	1933.189583	-0.139271968	0.979231897	1933.189583
FBgn0036427	CG4613 gene product from transcript	2.542931485	0.014559564	0.998322595	2.542931485
FBgn0003459	stonewall	225.508055	-0.059448259	0.994497358	225.508055
FBgn0036422	CG3868 gene product from transcript	5792.266987	-1.094410061	0.076752946	5792.266987
FBgn0036421	CG13481 gene product from transcrip	1.451401547	0.309643055		1.451401547
FBgn0052141	CG32141 gene product from transcrip	3.996371585	0.987013024	0.126263195	3.996371585
FBgn0036415	CG7768 gene product from transcript	37.76045875	0.817111227	0.42048989	37.76045875
FBgn0036416	CG7924 gene product from transcript	1312.712684	-0.488275575	0.556956355	1312.712684
FBgn0036417	CG7906 gene product from transcript	2200.318465	-0.669225354	0.380412928	2200.318465
FBgn0001085	frizzled	657.4291262	0.094590455	0.988572405	657.4291262
FBgn0036419	CG13482 gene product from transcrip	1234.960543	0.244090591	0.875497214	1234.960543
FBgn0000411	Dichaete	420.4597878	-0.001740459	0.999629143	420.4597878
FBgn0042630	CG32139 gene product from transcrip	152.1346599	0.149603731	0.975861786	152.1346599
FBgn0036411	CG7345 gene product from transcript	50.2865761	-1.30924136	0.008662193	50.2865761
FBgn0000639	Fat body protein 1	561296.0299	0.11409557	0.972176266	561296.0299
FBgn0043550	Tetraspanin 68C	13.46599742	-0.28507739	0.965076657	13.46599742
FBgn0029167	Hemolectin	3615.854762	0.734339238	0.3397435	3615.854762
FBgn0036382	CG13737 gene product from transcrip	36.83803139	0.43444592	0.83696011	36.83803139
FBgn0001108	Glued	2431.550691	-0.024739812	0.996957673	2431.550691
FBgn0052137	CG32137 gene product from transcrip	1973.792426	0.12709663	0.968985781	1973.792426
FBgn0036389	short spindle 2	0			0
FBgn0001216	Heat shock protein cognate 1	7.347259123	0.015548054	0.998322595	7.347259123
FBgn0036393	CG17362 gene product from transcrip	1193.501991	0.385803193	0.790236611	1193.501991
FBgn0036394	CG9040 gene product from transcript	3193.780783	0.55057559	0.399353831	3193.780783
FBgn0036395	CG17361 gene product from transcrip	272.5185162	-0.021089586	0.998322595	272.5185162
FBgn0036398	CG9007 gene product from transcript	1269.916759	0.199744096	0.963501097	1269.916759
FBgn0036402	CG6650 gene product from transcript	1135.812038	0.013607224	0.998322595	1135.812038
FBgn0036403	CG6661 gene product from transcript	0			0
FBgn0026418	CG6603 gene product from transcript	7165.610539	-0.026118009	0.9945211	7165.610539
FBgn0036405	CG6833 gene product from transcript	368.1087424	-0.018305135	0.998322595	368.1087424
FBgn0013563	Peroxin 1	540.4330852	-0.295100632	0.686455458	540.4330852
FBgn0036410	CG8100 gene product from transcript	4477.299519	0.00347506	0.998322595	4477.299519
FBgn0036406	CG13484 gene product from transcrip	171.6225684	-0.080229166	0.994497358	171.6225684
FBgn0036397	CG8783 gene product from transcript	196.241708	-0.317597721	0.660849346	196.241708
FBgn0036396	CG17359 gene product from transcrip	114.483069	0.080897952	0.994497358	114.483069
FBgn0036391	CG17364 gene product from transcrip	68.80045871	0.836188416	0.123492275	68.80045871
FBgn0036390	CG13738 gene product from transcrip	0.797185605	0.105675119		0.797185605
FBgn0025874	Meiotic central spindle	239.5578125	-0.046049802	0.9945211	239.5578125
FBgn0036386	CG8833 gene product from transcript	292.8358267	-0.133049982	0.985645036	292.8358267
FBgn0026376	Ral guanine nucleotide dissociation stir	956.1919662	-0.041651047	0.994876798	956.1919662
FBgn0036381	CG8745 gene product from transcript	8298.949639	-0.250975728	0.822495995	8298.949639
FBgn0040812	CG8750 gene product from transcript	0.07379547	0.108851729		0.07379547
FBgn0036380	CG8757 gene product from transcript	0.100708642	0		0.100708642
FBgn0036368	CG10738 gene product from transcrip	63.65490938	-0.544467884	0.56367707	63.65490938
FBgn0036373	Tondu-domain-containing Growth Inhi	1649.372728	0.193166049	0.871091663	1649.372728
FBgn0036376	CG10743 gene product from transcrip	1245.625786	-0.11576885	0.969602255	1245.625786
FBgn0036377	CG10710 gene product from transcrip	172.6617291	0.188897143	0.979231897	172.6617291
FBgn0036372	Actin binding protein 1	2228.667197	-0.359068593	0.448219974	2228.667197
FBgn0036369	CG10089 gene product from transcrip	149.711325	0.188961383	0.959208615	149.711325
FBgn0036367	CG10116 gene product from transcrip	462.1844761	-0.69754876	0.42048989	462.1844761

FBgn0036366	CG10133 gene product from transcrip	294.5977494	-0.25950166	0.903436919	294.5977494	-0.101212845	0.95758415
FBgn0053262	CG33262 gene product from transcrip	0			0		
FBgn0052129	transfer RNA:CR32129	0.167676683	0		0.167676683	0.129953448	
FBgn0052128	transfer RNA:CR32128	0.060151179	-0.097832662		0.060151179	-0.093256207	
FBgn0052126	transfer RNA:CR32126	0.056320698	0		0.056320698	0	
FBgn0052127	transfer RNA:CR32127	0.314537515	-0.038553189		0.314537515	0.076752216	
FBgn0023095	capricious	1854.813105	0.450435921	0.459655876	1854.813105	0.377264853	0.589391688
FBgn0011844	transfer RNA:asp2:69F	0			0		
FBgn0003034	Sex Peptide	0			0		
FBgn0040814	CG14113 gene product from transcrip	2.755669723	0.497703866	0.247967756	2.755669723	0.027863174	
FBgn0036348	CG17687 gene product from transcrip	12.38143458	1.001317565	0.226503634	12.38143458	0.733737119	0.494860511
FBgn0040813	Neuropeptide-like precursor 2	23023.54861	0.495958006	0.818284402	23023.54861	0.349135975	0.855124824
FBgn0036349	SoxNeuro Co-Factor	0			0		
FBgn0036350	CG14111 gene product from transcrip	0.147590939	0.153120924		0.147590939	0	
FBgn0001256	Ecdysone-inducible gene L1	18.68942759	0.265123116	0.969626359	18.68942759	-0.109816403	0.973807349
FBgn0036352	CG14110 gene product from transcrip	3.426891259	0.252062686	0.966157244	3.426891259	-0.077246479	
FBgn0012010	transfer RNA:val4:70BCb	0.112641396	0		0.112641396	0	
FBgn0036364	CG14109 gene product from transcrip	2175.675156	0.145659599	0.978641661	2175.675156	-0.305514839	0.758333944
FBgn0036365	CG10732 gene product from transcrip	1447.535356	0.107486288	0.974005319	1447.535356	0.210777894	0.744650306
FBgn0036363	CG10140 gene product from transcrip	414.2863167	0.174355849	0.963501097	414.2863167	0.230706425	0.831996423
FBgn0036361	CG10154 gene product from transcrip	564.9209166	-0.302721315	0.943520261	564.9209166	-0.07553269	0.982932118
FBgn0036360	CG10713 gene product from transcrip	144.3476159	-0.67393632	0.484043024	144.3476159	-1.110066753	0.076374502
FBgn0012009	transfer RNA:val4:70BCa	0			0		
FBgn0036359	CG14105 gene product from transcrip	14.0988512	1.198263847	0.099832131	14.0988512	1.195311954	0.101734457
FBgn0260459	CG14106 gene product from transcrip	0.130116168	0.082464995		0.130116168	0	
FBgn0053263	CG33263 gene product from transcrip	1.295526338	0.035932371		1.295526338	0.019729385	
FBgn0052121	CG32121 gene product from transcrip	17.45865088	0.120856195	0.994497358	17.45865088	0.416852607	0.797974284
FBgn0036356	CG10222 gene product from transcrip	305.6045702	0.051396149	0.994876798	305.6045702	-0.046896644	0.984900211
FBgn0002573	senseless	153.0109527	0.371074938	0.727413193	153.0109527	0.233410932	0.848588436
FBgn0036354	Proteome of centrioles 1	333.6432405	-0.076961988	0.994497358	333.6432405	-0.219736455	0.883802014
FBgn0036353	CG10171 gene product from transcrip	1092.89373	-0.373706105	0.468371713	1092.89373	-0.122570386	0.91558304
FBgn0036351	CG14107 gene product from transcrip	5.076420151	0.227742769	0.975532686	5.076420151	-0.138290674	
FBgn0052119	CG32119 gene product from transcrip	11.29613421	1.222537024	0.034805189	11.29613421	0.140815625	0.951701663
FBgn0036321	CG14120 gene product from transcrip	476.0528037	-1.622831955	0.001644264	476.0528037	-1.916598157	0.000115067
FBgn0036323	CG14118 gene product from transcrip	219.0744106	0.521593954	0.487233923	219.0744106	0.280264025	0.815824306
FBgn0036328	CG10749 gene product from transcrip	0.516568287	0.208268862		0.516568287	0	
FBgn0036329	CG11262 gene product from transcrip	1.771091269	0.272706056	0.537066737	1.771091269	0	
FBgn0036330	CG11263 gene product from transcrip	3.08436973	-0.197858099	0.975861786	3.08436973	0.162472122	
FBgn0036331	CG14117 gene product from transcrip	130.1455815	0.104986973	0.99157593	130.1455815	0.170095282	0.911524876
FBgn0036334	CG11267 gene product from transcrip	1342.483019	0.154053827	0.967901085	1342.483019	0.377672629	0.567302715
FBgn0036336	isoprenylcysteine carboxylmethyltransf	101.9211083	0.003512542	0.998873611	101.9211083	0.095893128	0.962565277
FBgn0260441	Ribosomal protein S12	19918.36859	0.208269945	0.891131108	19918.36859	0.119094505	0.920127339
FBgn0036340	CG11274 gene product from transcrip	2826.477999	0.017490865	0.998322595	2826.477999	-0.132521061	0.861074999
FBgn0011284	Ribosomal protein S4	37468.05275	0.132482136	0.912494615	37468.05275	0.166520376	0.753572342
FBgn0036341	Syntaxin 13	2751.189173	-0.208117134	0.910739253	2751.189173	-0.329869962	0.634145882
[illegible]	[illegible]	[illegible]	[illegible]	[illegible]	[illegible]	[illegible]	[illegible]

FBgn0036004	Jumonji, AT rich interactive domain 2	1605.076691	0.375076228	0.658490808	1605.076691	0.318390786	0.692875202
FBgn0052037	CG32037 gene product from transcrip	9.043850145	0.115024937	0.994497358	9.043850145	-0.217541715	0.933048047
FBgn0052036	CG32036 gene product from transcrip	0.411682288	0.052770824		0.411682288	-0.035839188	
FBgn0036005	pallbearer	272.126427	-0.036892746	0.995120986	272.126427	-0.045517608	0.980691994
FBgn0011327	Ubiquitin carboxy-terminal hydrolase L	3166.584326	0.309526149	0.678655521	3166.584326	-0.013756691	0.99625321
FBgn0036000	CG3434 gene product from transcript	415.4427822	0.176194905	0.899899871	415.4427822	0.01934047	0.99193807
FBgn0035998	CG3437 gene product from transcript	46.8916309	-0.69927323	0.382757387	46.8916309	-0.354055223	0.796384175
FBgn0035997	pleiohomeotic like	1066.691597	-0.042621896	0.994497358	1066.691597	-0.14934953	0.850061293
FBgn0035996	CG3448 gene product from transcript	45.61580946	-0.063935527	0.995584438	45.61580946	-0.181674423	0.941623571
FBgn0005533	Ribosomal protein S17	34377.94644	-0.00624631	0.998322595	34377.94644	-0.156790074	0.897821035
FBgn0035989	CG3967 gene product from transcript	861.3393969	-0.287835778	0.77444208	861.3393969	-0.156823766	0.897821035
FBgn0035988	CG3982 gene product from transcript	18.49201709	0.765164643	0.481191376	18.49201709	-0.134947283	0.964908204
FBgn0035986	CG4022 gene product from transcript	626.9520736	-0.125249345	0.985645036	626.9520736	-0.046686774	0.985859045
FBgn0015218	Eukaryotic initiation factor 4E	6865.601526	0.103378864	0.988216049	6865.601526	0.353084487	0.603269348
FBgn0035983	CG4080 gene product from transcript	1740.98716	-0.018387614	0.998322595	1740.98716	-0.509312379	0.377578633
FBgn0001227	Heat shock gene 67Ba	205.2855893	-0.718056844	0.164421885	205.2855893	-0.648047806	0.247121583
FBgn0001225	Heat shock protein 26	4697.60676	0.75009769	0.442049599	4697.60676	0.157234568	0.950970297
FBgn0001229	Heat shock gene 67Bc	1346.608608	-0.95505136	0.123844676	1346.608608	-0.925511897	0.145257789
FBgn0011769	Ferredoxin	167.9919865	0.084087407	0.994497358	167.9919865	0.098121319	0.952826659
FBgn0004379	Kinesin-like protein at 67A	434.5385456	0.105007443	0.975861786	434.5385456	0.094279089	0.933048047
FBgn0035970	CG4483 gene product from transcript	0.809058269	0.179742622		0.809058269	0	
FBgn0035968	Slc45 ortholog 1	2818.021762	0.070966415	0.987910537	2818.021762	-0.023278478	0.9872372
FBgn0035993	Nuclear factor Y-box A	767.565067	-0.067536084	0.994497358	767.565067	-0.065151819	0.977405436
FBgn0052034	transfer RNA:thr:pseudogene	0			0		
FBgn0035936	Tetraspanin 66E	1107.152097	-0.317411779	0.680496943	1107.152097	0.214215881	0.805797107
FBgn0040321	Gram-negative bacteria binding protein	5173.694662	-0.495241403	0.054008323	5173.694662	-0.252599641	0.572501461
FBgn0011787	mitochondrial ribosomal protein L12	991.5650267	0.26477504	0.859393667	991.5650267	0.33825081	0.662580752
FBgn0035941	CG13313 gene product from transcrip	11.16704283	0.198598949	0.985645036	11.16704283	-0.072786827	0.98613036
FBgn0035944	CG5021 gene product from transcript	1331.861076	0.061689254	0.991146172	1331.861076	0.104672667	0.899556396
FBgn0035947	Signal recognition particle protein 68	2887.908955	0.133268503	0.960363267	2887.908955	0.200280561	0.777818501
FBgn0035949	CG13314 gene product from transcrip	18.48481675	-1.033563078	0.190847983	18.48481675	-0.424612738	0.796615544
FBgn0035951	CG5068 gene product from transcript	772.7271272	-0.160972705	0.968528723	772.7271272	-0.145394052	0.917910585
FBgn0035953	CG5087 gene product from transcript	638.9848132	0.003682149	0.998322595	638.9848132	0.005402818	0.998730566
FBgn0035954	Dorsocross3	91.41585098	-0.218682571	0.959208615	91.41585098	-0.131249347	0.946798903
FBgn0028789	Dorsocross1	107.2881295	0.091790557	0.994151247	107.2881295	-0.501701292	0.461886218
FBgn0035957	CG5144 gene product from transcript	18.34601688	1.260181767	0.072750638	18.34601688	0.589399963	0.652509677
FBgn0035960	CG4942 gene product from transcript	104.8159529	0.578465419	0.244029996	104.8159529	0.418818294	0.532682942
FBgn0023479	CG4821 gene product from transcript	4699.366277	0.541345885	0.068621386	4699.366277	0.468344358	0.15337121
FBgn0043806	CG32032 gene product from transcrip	653.1414851	0.207869341	0.886446899	653.1414851	0.016926839	0.994998875
FBgn0040827	CG13315 gene product from transcrip	8304.672514	-0.222486521	0.924143028	8304.672514	-0.394881974	0.590145587
FBgn0004244	Resistant to dieldrin	196.8125732	0.790740639	0.292528621	196.8125732	0.740401462	0.340840387
FBgn0035965	USE1 ortholog (S. cerevisiae)	209.6435073	0.154758225	0.960742862	209.6435073	0.060412119	0.971833087
FBgn0011206	boule	367.023135	0.347165472	0.85659012	367.023135	0.56668376	0.492734138
FBgn0035959	CG4911 gene product from transcript	2769.471652	0.178643233	0.972479043	2769.471652	0.034871191	0.990025502
FBgn0000116	Arginine kinase	[illegible]	[illegible]	[illegible]	[illegible]	[illegible]	[illegible]

[illegible]

FBgn0035619	CG10592 gene product from transcrip	721.1224569	-0.890192381	0.150073237	721.1224569	-0.382677866	0.763334728
FBgn0003486	spook	1.236055682	-0.006234852		1.236055682	-0.258047068	
FBgn0015766	CG10596 gene product from transcrip	20727.69752	0.058197045	0.994497358	20727.69752	0.081390173	0.941692091
FBgn0029118	CG10622 gene product from transcrip	6195.127972	0.093957607	0.993127879	6195.127972	0.272891707	0.783540904
FBgn0035612	CG10625 gene product from transcrip	25366.71488	0.388209643	0.726127249	25366.71488	0.934333131	0.031703771
FBgn0035611	CG13285 gene product from transcrip	282.886699	0.374755116	0.835962233	282.886699	0.526276364	0.579112715
FBgn0011986	transfer RNA:ser7:64D	0			0		
FBgn0052420	transfer RNA:CR32420	0.112641396	0		0.112641396	0	
FBgn0035600	CG4769 gene product from transcript	7810.076197	0.104749382	0.993127879	7810.076197	0.376685652	0.670617046
FBgn0035607	CG4835 gene product from transcript	135.570331	-1.831747252	0.001099303	135.570331	-1.248242862	0.078862013
FBgn0035610	Leucokinin receptor	146.6830598	-0.3979223	0.625787581	146.6830598	-0.415292201	0.555968073
FBgn0035608	CG10630 gene product from transcrip	28.45133209	1.196018033	0.100142771	28.45133209	-0.014335183	0.998730566
FBgn0035604	Ionotropic receptor 64a	0.514443529	0.156350424		0.514443529	0	
FBgn0035603	CG10635 gene product from transcrip	1527.181146	0.046349127	0.994876798	1527.181146	0.049903813	0.980691994
FBgn0035601	CG10640 gene product from transcrip	7143.392396	0.019932425	0.996536713	7143.392396	0.138823611	0.804006948
FBgn0004380	Kinesin-like protein at 64D	1318.450247	-0.165656479	0.88370699	1318.450247	-0.123721147	0.876744307
FBgn0052421	transfer RNA:CR32421	0			0		
FBgn0016031	lamina ancestor	2619.366864	-0.100172723	0.993966728	2619.366864	-0.162418548	0.918961524
FBgn0035594	CG4597 gene product from transcript	0.320419228	0.133417562		0.320419228	0.061047253	
FBgn0040842	CG15212 gene product from transcrip	91.86949045	0.374244925	0.807463547	91.86949045	0.91839948	0.078862013
FBgn0035593	CG4603 gene product from transcript	1413.226407	0.088645457	0.99157593	1413.226407	0.006788669	0.998730566
FBgn0035591	CG4611 gene product from transcript	245.7000491	-0.13694538	0.971762817	245.7000491	-0.000876622	1
FBgn0035589	Charged multivesicular body protein 2b	1256.014458	-0.197803631	0.918956451	1256.014458	-0.364730486	0.54592557
FBgn0035587	CG4623 gene product from transcript	486.3199606	0.056286414	0.994876798	486.3199606	0.208193679	0.888653195
FBgn0028962	mitochondrial alanyl-tRNA synthetase	658.6174544	-0.043124383	0.9945211	658.6174544	0.22847916	0.764380931
FBgn0010747	Signal recognition particle protein 54k	4368.62361	0.217384166	0.933260463	4368.62361	0.187303763	0.882312613
FBgn0035598	CG4669 gene product from transcript	9.161765506	0.775665965	0.307625378	9.161765506	0.001304815	1
FBgn0035586	CG10671 gene product from transcrip	544.9051645	0.011109959	0.998322595	544.9051645	0.146380621	0.889960653
FBgn0035588	CG10672 gene product from transcrip	1581.534518	-0.285062448	0.805580095	1581.534518	-0.096250753	0.950153589
FBgn0035590	p53-related protein kinase ortholog	302.6420841	-0.239148967	0.919514095	302.6420841	-0.348733177	0.689888554
FBgn0035592	CG10674 gene product from transcrip	11.81658742	-0.375132178	0.920136002	11.81658742	0.151269106	0.958832396
FBgn0040843	CG15213 gene product from transcrip	216.5648612	0.207151198	0.975999817	216.5648612	0.658132913	0.466732495
FBgn0052523	CG32523 gene product from transcrip	0.595434439	0.133698961		0.595434439	0.047973695	
FBgn0011954	transfer RNA:arg:19F	0			0		
FBgn0031169	CG1494 gene product from transcript	7.973959101	0.301889422	0.95868017	7.973959101	0.00434272	1
FBgn0020369	Regulatory particle triple-A ATPase 6	10930.9405	-0.220785799	0.940580989	10930.9405	-0.381230585	0.644307623
FBgn0031174	CG1486 gene product from transcript	4025.862181	0.111697399	0.985645036	4025.862181	0.037362058	0.986877529
FBgn0000709	flightless I	1669.002975	-0.114242686	0.975999817	1669.002975	-0.090100243	0.944894567
FBgn0015379	dodo	990.3080481	0.057201867	0.994497358	990.3080481	-0.036560345	0.986877529
FBgn0011710	Septin 1	2410.662133	-0.22810635	0.839295568	2410.662133	-0.375669688	0.456588334
FBgn0003423	sluggish A	3393.676839	-0.221714064	0.803861163	3393.676839	-0.028401036	0.986877529
FBgn0020272	misato	580.6909314	-0.264978252	0.830061197	580.6909314	-0.374805408	0.564767538
FBgn0031182	CG1644 gene product from transcript	593.7691979	-0.445534375	0.526670516	593.7691979	-0.457289769	0.484683967
FBgn0031181	Ionotropic receptor 20a	1.186538215	-0.220237108		1.186538215	-0.225407276	
FBgn0031178	CG10918 gene product from transcrip	6447.149779	0.448470484	0.767207106	6447.149779	-0.675269787	0.424499314

FBgn0031398	CG10880 gene product from transcrip	273.0499845	-0.343527152	0.448260876	273.0499845	-0.283665274	0.597449784	273.0499845	[illegible]
FBgn0010288	Ubiquitin carboxy-terminal hydrolase	3974.384791	-0.10869104	0.979231897	3974.384791	-0.266137439	0.699222128	3974.384791	[illegible]
FBgn0031397	CG15385 gene product from transcrip	506.9293707	0.232300946	0.952645803	506.9293707	0.36459235	0.728212704	506.9293707	[illegible]
FBgn0053124	CG33124 gene product from transcrip	31.97660524	0.436647096	0.872420581	31.97660524	0.511663554	0.728212704	31.97660524	[illegible]
FBgn0051668	CG31668 gene product from transcrip	18.05629621	0.716318658	0.540032514	18.05629621	0.672978628	0.564767538	18.05629621	[illegible]
FBgn0031395	CG10874 gene product from transcrip	493.7200926	0.112194878	0.979231897	493.7200926	-0.161794102	0.878786989	493.7200926	[illegible]
FBgn0000097	anterior open	1072.119105	-0.215870365	0.967901085	1072.119105	-0.464939865	0.651795237	1072.119105	[illegible]
FBgn0031391	CG11723 gene product from transcrip	545.673205	-0.215301149	0.817384807	545.673205	-0.278462251	0.607940701	545.673205	[illegible]
FBgn0031390	CG31671 gene product from transcrip	1753.64782	0.005862056	0.998322595	1753.64782	0.03606504	0.982864788	1753.64782	[illegible]
FBgn0031389	CG4259 gene product from transcript	475.4774231	0.395123842	0.633172703	475.4774231	0.296055894	0.748235094	475.4774231	[illegible]
FBgn0051669	transfer RNA:CR31669	0.07379547	0.108851729		0.07379547	0		0.07379547	[illegible]
FBgn0051940	transfer RNA:CR31940	0.116471877	-0.071974357		0.116471877	-0.068468588		0.116471877	[illegible]
FBgn0051939	transfer RNA:CR31939	0.578664578	0.006812677		0.578664578	0.104711426		0.578664578	[illegible]
FBgn0040718	CG15353 gene product from transcrip	88.33605117	0.01212881	0.998322595	88.33605117	0.312927278	0.824086124	88.33605117	[illegible]
FBgn0043539	Odorant-binding protein 22a	2.684595837	-0.071443896	0.994497358	2.684595837	0.151927733		2.684595837	[illegible]
FBgn0031377	CG15356 gene product from transcrip	438.9094956	0.200516789	0.791227483	438.9094956	-0.014820854	0.993865695	438.9094956	[illegible]
FBgn0031375	earmuff	91.59573592	-0.036257292	0.99723561	91.59573592	-0.231315783	0.867222024	91.59573592	[illegible]
FBgn0011859	transfer RNA:gly3:22BCa	0.415108826	-0.080156716		0.415108826	-0.115326819		0.415108826	[illegible]
FBgn0026398	Odorant receptor 22a	2.909946705	-0.07422461	0.9945211	2.909946705	-0.241219052		2.909946705	[illegible]
FBgn0026397	Odorant receptor 22b	2.475079262	0.055958932	0.995120986	2.475079262	-0.038365489		2.475079262	[illegible]
FBgn0031351	CG14352 gene product from transcrip	167.4700663	-0.274130914	0.910739253	167.4700663	-0.316595256	0.786500777	167.4700663	[illegible]
FBgn0011570	capping protein beta	3477.733178	0.084511666	0.972330876	3477.733178	0.023497481	0.98613036	3477.733178	[illegible]
FBgn0031357	mitochondrial ribosomal protein L48	270.6963995	0.201903113	0.967901085	270.6963995	0.118736357	0.95247517	270.6963995	[illegible]
FBgn0031360	CG31937 gene product from transcrip	1506.290383	-0.240942312	0.919789205	1506.290383	-0.095739678	0.95914272	1506.290383	[illegible]
FBgn0031364	CG17648 gene product from transcrip	0.167676683	0		0.167676683	0.129953448		0.167676683	[illegible]
FBgn0045497	Gustatory receptor 22e	0.281537587	0.10133235		0.281537587	-0.039571732		0.281537587	[illegible]
FBgn0031372	CG7295 gene product from transcript	2.611828341	0.598161291	0.250522657	2.611828341	0.078796753		2.611828341	[illegible]
FBgn0031373	CG15358 gene product from transcrip	34.66362236	0.693086421	0.536514704	34.66362236	0.190908679	0.940118668	34.66362236	[illegible]
FBgn0051663	CG31663 gene product from transcrip	578.812259	-0.26187847	0.755584388	578.812259	-0.235490123	0.74945674	578.812259	[illegible]
FBgn0031367	calcutta cup	2.55697943	0.441781651	0.642151225	2.55697943	0.129122101		2.55697943	[illegible]
FBgn0051664	CG31664 gene product from transcrip	304.940199	-0.205391895	0.979841869	304.940199	0.089913617	0.980691994	304.940199	[illegible]
FBgn0051933	CG31933 gene product from transcrip	1.475909391	0.233889205		1.475909391	0		1.475909391	[illegible]
FBgn0045501	Gustatory receptor 22a	0			0			0	[illegible]
FBgn0045498	Gustatory receptor 22d	0			0			0	[illegible]
FBgn0031365	CG17650 gene product from transcrip	17.43310591	0.565353663	0.736375381	17.43310591	0.044269951	0.991444605	17.43310591	[illegible]
FBgn0041249	Gustatory receptor 22f	0			0			0	[illegible]
FBgn0027597	CG17712 gene product from transcrip	771.9850409	-0.10026338	0.979231897	771.9850409	-0.139470301	0.889403736	771.9850409	[illegible]
FBgn0031361	CG17652 gene product from transcrip	211.1532232	0.014307262	0.998322595	211.1532232	0.103550895	0.936641868	211.1532232	[illegible]
FBgn0000579	Enolase	48321.55165	0.082706711	0.994497358	48321.55165	0.41122972	0.708289172	48321.55165	[illegible]
FBgn0031359	Rim2 ortholog (S. cerevisiae)	1332.971702	-0.16652695	0.938791798	1332.971702	-0.455469371	0.265714407	1332.971702	[illegible]
FBgn0031356	CG17660 gene product from transcrip	1426.392507	-0.182917223	0.910739253	1426.392507	-0.268015718	0.670182882	1426.392507	[illegible]
FBgn0021906	Rieske iron-sulfur protein	4565.488068	0.218914122	0.9379015	4565.488068	0.393366802	0.611315446	4565.488068	[illegible]
FBgn0051935	CG31935 gene product from transcrip	558.6178922	-0.120494725	0.975999817	558.6178922	-0.160658463	0.880071821	558.6178922	[illegible]
FBgn0031347	CG10869 gene product from transcrip	23.18815995	1.094357959	0.157146608	23.18815995	-0.08166644	0.984488479	23.18815995	[illegible]
FBgn0001174	CG7428 gene product from transcript	0.417154922	-0.02693038		0.417154922	-0.071357693		0.417154922	[illegible]
FBgn0031345	CG18132 gene product from transcrip	0.221386409	0.173816599		0.221386409	0		0.221386409	[illegible]
FBgn0031327	CG5397 gene product from transcript	2124.151892	0.0779314	0.993127879	2124.151892	-0.124509713	0.917910585	2124.151892	[illegible]
FBgn0041097	CG5423 gene product from transcript	276.0417922	-0.1056188	0.991146172	276.0417922	-0.273293586	0.796097411	276.0417922	[illegible]
FBgn0011294	antennal protein 5	0			0			0	[illegible]
FBgn0031331	CG5440 gene product from transcript	1.367164378	0.271984378		1.367164378	0		1.367164378	[illegible]
FBgn0026141	Cytoplasmic dynein light chain 2	6.709506015	0.958077448	0.026325924	6.709506015	0.016816656	0.996857156	6.709506015	[illegible]
FBgn0031337	CG14346 gene product from transcrip	2.269353369	0.474220884	0.495695917	2.269353369	0.184018658		2.269353369	[illegible]
FBgn0003926	small nuclear RNA U3 at 22A	0.060151179	-0.097832662		0.060151179	-0.093256207		0.060151179	[illegible]
FBgn0051928	CG31928 gene product from transcrip	0.380023996	-0.194424886		0.380023996	-0.192503627		0.380023996	[illegible]
FBgn0053128	CG33128 gene product from transcrip	14851.58261	-0.930204376	0.128426137	14851.58261	-0.878686424	0.172040892	14851.58261	[illegible]
FBgn0051926	CG31926 gene product from transcrip	10.00075663	-0.204449018	0.979231897	10.00075663	-0.469261505	0.741930728	10.00075663	[illegible]
FBgn0051661	CG31661 gene product from transcrip	0			0			0	[illegible]
FBgn0031344	CG7420 gene product from transcript	185.2525655	0.047912232	0.995584438	185.2525655	-0.148734051	0.939156908	185.2525655	[illegible]
FBgn0031343	CG18131 gene product from transcrip	20.1535422	1.227277004	0.087258785	20.1535422	0.680417476	0.562473024	20.1535422	[illegible]
FBgn0002543	leak	577.0383602	-0.050509719	0.995584438	577.0383602	-0.278017546	0.850061293	577.0383602	[illegible]
FBgn0053126	Neural Lazarillo	507.1957191	-0.337317027	0.940195249	507.1957191	-1.410664677	0.022898482	507.1957191	[illegible]
FBgn0051659	CG31659 gene product from transcrip	5.062389282	-0.082081019	0.994876798	5.062389282	-0.513161047		5.062389282	[illegible]
FBgn0031335	CG5565 gene product from transcript	2.304689534	0.501984693	0.365358959	2.304689534	-0.066774049		2.304689534	[illegible]
FBgn0051924	CG31924 gene product from transcrip	0.295181878	0.186873145		0.295181878	0		0.295181878	[illegible]
FBgn0031333	CG5561 gene product from transcript	1.129477497	0.252030884		1.129477497	0.033942212		1.129477497	[illegible]
FBgn0031332	CG5556 gene product from transcript	0.516568287	0.208268862		0.516568287	0		0.516568287	[illegible]
FBgn0028481	CG4341 gene product from transcript	268.7130002	0.035122028	0.995584438	268.7130002	-0.341498269	0.619371379	268.7130002	[illegible]
FBgn0031296	CG4415 gene product from transcript	11.31543571	1.290846301	0.04697587	11.31543571	1.168365983	0.092739499	11.31543571	[illegible]
FBgn0015905	asteroid	350.0597858	-0.02540883	0.996957673	350.0597858	-0.108313137	0.917910585	350.0597858	[illegible]
FBgn0031298	Autophagy-specific gene 4a	2916.59146	-0.227403368	0.77444208	2916.59146	-0.314756345	0.50068676	2916.59146	[illegible]
FBgn0031301	CG14339 gene product from transcrip	3.525899269	0.490378123	0.652274434	3.525899269	-0.116174912		3.525899269	[illegible]
FBgn0031302	CG14340 gene product from transcrip	4.743928463	-0.002916114	0.999629143	4.743928463	0.001259429		4.743928463	[illegible]
FBgn0031304	CG4552 gene product from transcript	1232.440291	-0.018663125	0.99723561	1232.440291	-0.057610344	0.951701663	1232.440291	[illegible]
FBgn0031306	CG4577 gene product from transcript	401.0181971	-0.410202637	0.48088494	401.0181971	-0.56621934	0.185133756	401.0181971	[illegible]
FBgn0031307	Major Facilitator Superfamily Transport	4936.064832	-0.032696462	0.998322595	4936.064832	0.22570637	0.872923767	4936.064832	[illegible]
FBgn0031310	Vacuolar protein sorting 29	590.3851667	-0.22807025	0.871091663	590.3851667	-0.183900571	0.852559484	590.3851667	[illegible]
FBgn0031312	Transport and Golgi organization 14	392.8303599	-0.016658184	0.998322595	392.8303599	-0.122769905	0.950970297	392.8303599	[illegible]
FBgn0031314	Integrator 14	491.749667	-0.140925109	0.960742862	491.749667	-0.235400008	0.739351375	491.749667	[illegible]
FBgn0031315	CG14341 gene product from transcrip	438.4209871	-0.197496531	0.917248088	438.4209871	-0.210981346	0.818935456	438.4209871	[illegible]
FBgn0051922	CG31922 gene product from transcrip	30.80618804	0.139943675	0.991865251	30.80618804	0.220531264	0.915954217	30.80618804	[illegible]
FBgn0031318	CG4887 gene product from transcript	1852.541608	-0.14501955	0.909440876	1852.541608	-0.283190074	0.475620641	1852.541608	[illegible]
FBgn0031319	CG4896 gene product from transcript	1189.166682	-0.201330607	0.817519408	1189.166682	-0.336061688	0.407302382	1189.166682	[illegible]
FBgn0031321	tRNA-guanine transglycosylase	232.1389854	-0.226040015	0.971565969	232.1389854	-0.198111641	0.926252243	232.1389854	[illegible]
FBgn0040723	CG5011 gene product from transcript	296.8119999	-1.001953107	0.017402645	296.8119999	-0.71327402	0.182223667	296.8119999	[illegible]
FBgn0031324	CG14342 gene product from transcrip	9.879228218	-0.343811776	0.944197659	9.879228218	-0.672368435	0.566092206	9.879228218	[illegible]
FBgn0031323	CG5139 gene product from transcript	1.610553111	0.378784928	0.569140102	1.610553111	0.183325338		1.610553111	[illegible]
FBgn0031320	CG5126 gene product from transcript	564.8324721	-0.14196474	0.979231897	564.8324721	-0.07151371	0.973213602	564.8324721	[illegible]
FBgn0031317	CG5118 gene product from transcript	600.7851059	-0.461434251	0.14363479	600.7851059	-0.589444686	0.031224252	600.7851059	[illegible]
FBgn0024314	Phospholipase A2 activator protein	4162.177432	-0.103252521	0.989375441	4162.177432	-0.192129958	0.864997596	4162.177432	[illegible]
FBgn0031313	CG5080 gene product from transcript	1993.974549	0.15528572	0.975999817	1993.974549	0.118764925	0.94714921	1993.974549	[illegible]
FBgn0031309	TFB4 ortholog (S. cerevisiae)	183.9526104	-0.140218176	0.969626359	183.9526104	0.03490846	0.986877529	183.9526104	[illegible]
FBgn0031305	CG4715 gene product from transcript	102.0175471	-1.543665261	0.000731719	102.0175471	-1.290401208	0.011453346	102.0175471	[illegible]
